# Effect of Electroacupuncture on Intestinal Mucosal Barrier in IBS-D Rats: Analysis Based on RNA-seq

**DOI:** 10.2174/0113862073395229250513074835

**Published:** 2025-05-27

**Authors:** Jingru Ruan, Jingwei Zhu, Kuiwu Li, Ziye Wang, Ting Wang, Xiaoyu Han, Xiaomin Li, Yucheng Fang, Xiaoge Song, Haoran Chu

**Affiliations:** 1 College of Traditional Chinese Medicine, Anhui University of Traditional Chinese Medicine, 350 Longzihu Road, Hefei 230012, Anhui, China;; 2 College of Second Clinical Medical, Anhui University of Traditional Chinese Medicine, 300 Shouchun Road, Hefei, 230061, Anhui, China;; 3 College of First Clinical Medical, Anhui University of Traditional Chinese Medicine, 350 Longzihu Road, Hefei, 230012, Anhui, China;; 4 Institute of Acupuncture and Meridians, Anhui University of Traditional Chinese Medicine, 103 Meishan Road, Hefei 230038, Anhui, China;; 5 Institute of Clinical Acupuncture and Moxibustion, Anhui Academy of Traditional Chinese Medicine, 103 Meishan Road, Hefei 230038, Anhui, China

**Keywords:** IBS-D, electroacupuncture, RNA-Seq, ceRNA, lncRNA-miRNA-mRNA, mast cell, intestinal mucosal barrier

## Abstract

**Objective:**

Transcriptome-level insights into electroacupuncture (EA)’s mechanisms for alleviating intestinal mucosal barrier damage in diarrhea-predominant irritable bowel syndrome (IBS-D) are limited. This study aimed to construct ceRNA networks and elucidate EA's role in restoring barrier integrity *via* lncRNA-miRNA-mRNA regulation in IBS-D rats.

**Methods:**

The IBS-D model was established by neonatal maternal separation (NMS), 4% acetic acid enema and restrain stress (RS). Rats were randomized into control, model, and EA groups. After 2-week EA treatment, colonic morphology was assessed by HE staining and TEM; intestinal barrier biomarkers were analyzed *via* ELISA and WB. RNA-seq identified differentially expressed RNAs (DE RNAs) to construct ceRNA networks. GO and KEGG analyzed EA-modulated DE mRNAs. RT-qPCR validated RNA-seq; WB and IF confirmed mast cell (MC) involvement in EA-regulated pathways.

**Results:**

RNA-seq identified 426 up-regulated and 429 down-regulated DE mRNAs, 342 up-regulated and 362 down-regulated DE lncRNAs, and 10 up-regulated and 48 down-regulated DE miRNAs following EA. Constructed ceRNA networks included 7 DE lncRNAs-miR-139-3p-*Bid* and -miR-378b-*Slc4a5*. GO analysis linked EA to defense response, hormone regulation, and cytokine function pathways. KEGG implicated antigen processing/presentation, neuroactive ligand-receptor interaction, PPAR signaling, and glutathione metabolism. RT-qPCR validated RNA-seq results.

**Conclusion:**

This RNA-seq study reveals EA mitigates IBS-D intestinal mucosal barrier damage by regulating genes and ceRNA networks, providing novel transcriptomic insights into its therapeutic mechanisms.

## 1 INTRODUCTION

Irritable bowel syndrome (IBS), classified as a functional gastrointestinal disorder, manifests as chronic abdominal pain associated with altered bowel habits and stool characteristics according to Rome IV diagnostic criteria [1]. With a global prevalence approximating 4.1% (Rome IV criteria) and 10.1% (Rome III criteria) based on epidemiological surveys, this condition imposes significant burdens on patients' quality of life through its chronic relapsing nature and therapeutic challenges [2, 3]. The Bristol Stool Form Scale facilitates IBS subtyping into four categories: diarrhea-predominant (IBS-D), constipation-predominant (IBS-C), mixed (IBS-M), and unclassified (IBS-U), among which IBS-D represents the most prevalent subtype under current diagnostic frameworks [4].

The pathophysiology of IBS-D involves multifactorial mechanisms, with emerging evidence highlighting four interrelated pathways: low-level intestinal inflammation, dysregulation of the gut-brain axis, gut microbiota alterations, and intestinal mucosal barrier dysfunction [5-7]. Recent scientific focus has increasingly centered on the pivotal role of intestinal barrier integrity in disease pathogenesis, particularly its interaction with other pathophysiological components. Studies have shown that patients with IBS have increased intestinal permeability [8, 9], which is closely related to mast cell (MC) activation and degranulation [10-12]. MCs are mainly distributed in the lamina propria and submucosa of the gastrointestinal tract [13]. In IBS-D, MCs activate degranulation and secrete large amounts of tryptase, which activates protease-activated receptor 2 (PAR-2) on the surface of intestinal epithelial cells, resulting in tight junction (TJ) rearrangement of epithelial cells, disrupting mucosal integrity and increasing permeability [14]. Furthermore, some pro-inflammatory factors secreted by MCs, such as tumor necrosis factor-α (TNF-α), interferon-γ (IFN-γ), and interleukin-1β (IL-1β), can also alter the expression of TJ proteins, leading to intestinal epithelial barrier dysfunction [15].

Currently, the treatment of IBS-D is mainly taking antidiarrheal drugs, antispasmodics, probiotics, and other drugs [16]. However, therapeutic efficacy is frequently compromised by adverse effects, including paradoxical constipation, nausea, and abdominal discomfort, compounded by the economic burden of long-term medication regimens [17, 18].

Acupuncture has a good effect on improving digestive system diseases, including IBS-D and functional dyspepsia [19, 20]. Acupuncture demonstrates the distinct advantages over conventional therapies: cost-effectiveness and safety. Clinical evidence confirms its efficacy in alleviating core IBS symptoms, including abdominal pain, bloating, and incomplete defecation, while enhancing patients' quality of life [21, 22]. Although the effectiveness of acupuncture in treating IBS-D has been recognised to some extent, the mechanism of action of acupuncture in treating IBS-D is, unfortunately, still unclear. Currently, studies of the mechanism of action of acupuncture in the treatment of IBS-D have mainly focused on the regulation of a single pathway or protein by acupuncture. This has resulted in a certain lack of systematisation and comprehensiveness that reflects the role of acupuncture itself in treating the disease by modulating multiple systems, levels, and targets.

There is increasing evidence that non-coding RNA, including long non-coding RNA (lncRNA) and microRNA (miRNA), plays an important role in the study of gastrointestinal diseases such as IBS and inflammatory bowel disease (IBD) [23-25]. The competitive endogenous RNA (ceRNA) hypothesis proposes that RNAs with miRNA target sites can bind miRNAs in competition with mRNAs, forming a coregulatory posttranscriptional network [26]. Numerous studies imply that lncRNAs can serve as ceRNAs to bind to miRNAs and thus affect the expression of downstream genes [27-29]. Nevertheless, it is rare to explore the mechanism of electroacupuncture (EA) in the treatment of IBS-D from the perspective of ceRNA.

To elucidate the mechanisms underlying EA treatment for IBS-D, we systematically investigated EA-induced alterations in colonic mucosal barrier function through histopathological and molecular analyses. Using high-throughput transcriptome sequencing, we identified differentially expressed mRNAs (DE mRNAs), long non-coding RNAs (DE lncRNAs), and miRNAs (DE miRNAs) in colonic tissues from IBS-D rat models before and after EA intervention. Through integrated bioinformatics approaches, we constructed a comprehensive ceRNA regulatory network potentially mediating EA's therapeutic effects on intestinal mucosal barrier repair. This network analysis, focusing on lncRNA-miRNA-mRNA interactions, revealed novel molecular pathways through which EA might ameliorate intestinal barrier dysfunction in IBS-D. Our findings provided multi-omics evidence supporting EA as a promising therapeutic strategy for IBS-D management while advancing our understanding of EA's regulatory mechanisms at the transcriptome level.

## 2 MATERIALS AND METHODS

### 2.1 Animals

Four Pregnant Sprague-Dawley rats (specific pathogen-free grade, gestational day 16 ± 2) were obtained from the Experimental Animal Center of Anhui University of Traditional Chinese Medicine [Animal Quality Certificate Number: SCXK (Liao) 2020-000^1^]. Animals were housed under controlled SPF conditions with a 12/12-hour light-dark cycle, ambient temperature maintained at 22-25°C, and relative humidity regulated between 45%-60%. All experimental procedures were conducted in strict accordance with the Guidelines for the Care and Use of Laboratory Animals and approved by the Animal Ethics Committee of Anhui University of Traditional Chinese Medicine (Ethical approval number: AHUCM-2020011).

### 2.2 Establishment and Evaluation of IBS-D Model

**Fig. (1):**
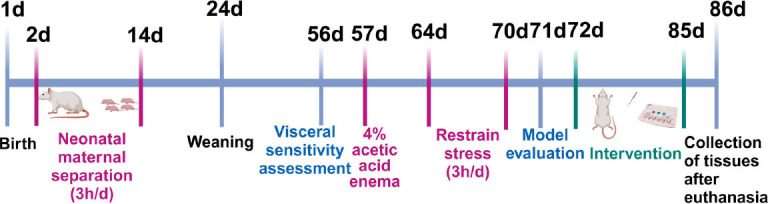
Timeline of the experimental procedures.

In this study, the IBS-D rat model was established by neonatal maternal separation (NMS), 4% acetic acid enema and restrain stress (RS) [30, 31] (Fig. **1**). From the second day after birth, the newborn rats were separated from the mother rats for 3 hours at 8:00 am every day for 14 days. To avoid the influence of the menstrual cycle of the female rats, only 25 male rats weighing 220±20g, were kept on normal chow until 56 days of age for the evaluation of visceral sensitivity. On day 57, after anaesthesia, 1 ml of 4% acetic acid was injected through a catheter (4-5 cm into the anus) and stimulation was maintained for 60 seconds and then rinsed. RS was performed for 7 consecutive days from the 64^th^ day (upper arm and chest were restrained from 8:00 to 11:00 every day). When the abdominal reflex (AWR) score of rats reached 3, the injected water volume decreased by 0.2-0.3 ml and the loose stool rate (LSR) is more than 60%, which were considered as the conditions for the successful preparation of the IBS-D rat model.

#### 2.2.1 Loose Stool Rate (LSR)

LSR was evaluated before modeling, after modeling and after intervention. The detailed recording method was that each rat was kept in a single cage with a stainless steel grid at the bottom, and a tray with filter paper was placed under the grid. The LSR of the rats in each group was observed within 6 hours. The difference between dry and loose faeces was determined by the presence of stains on the filter paper. LSR(%)= (number of loose stools/total number of faeces) ×100% [32].

#### 2.2.2 Evaluation of Minimum VolumeThreshold of AWR

Evaluation was performed before and after modeling and after intervention. The rats were fasted for 12 hours prior to the evaluation and allowed to drink only water. After the rats were anaesthetised with isoflurane, a paraffin oil-lubricated double-lumen catheter (2 mm in diameter and 31 cm in total length) was slowly inserted into the anus of the rats to a depth of approximately 4 cm from the anus. The catheter was secured to the tail of the rat, and the rat was then placed in a clear glass box to limit its rotation. After they were awake and calm, 0.9% sodium chloride solution at 26°C was slowly injected into the balloon for Colorectal balloon distention (CRD). CRD was maintained by water injection, and the amount of water injected into the rats when AWR reached 3 points was recorded, which was repeated three times for accurate measurement [33]. Each measurement was evaluated and recorded by two operators who did not know the grouping of rats.

### 2.3 Methods of Grouping and Intervention

In this study, rats were randomly divided into control, model and EA groups. During the modeling period, one rat died unexpectedly in both the model group and the EA group, so there were 11 rats in the model group and 12 rats in the EA group. The rats were bound to a rat fixator and then EA was performed on both sides of the Shangjuxu (ST 37, located on the outside of the hind limb, about 5 mm below the fibular head) and Tian Shu (ST 25, located 5mm beside navel). ST 25 was stabbed 3mm straight, while ST 37 was stabbed 5mm straight. Andi brand sterile acupuncture needle (diameter, 0.25mm; length, 13mm) and Huatuo brand SDZ-II electro-acupuncture instrument (Suzhou Medical Products Factory Co., Ltd.) were selected. The selection of electroacupuncture parameters was based on the records of relevant literature and the operation in related research [34, 35]. The acupoints on the same side were connected to the same pair of electrodes, and the density wave was 2Hz/15Hz and the current intensity was 1mA, so it was appropriate to shake the limbs of the rats slightly. EA once a day for 20 minutes each time for 14 days.

### 2.4 Tissue Hematoxylin-Eosin (HE) Staining

Colon tissue samples were fixed in 4% paraformaldehyde for 48 hours, dehydrated in gradient ethanol, cleared with xylene, soaked in wax, embedded in paraffin, sectioned, stained with HE, dehydrated again, cleared with neutral gum, and the morphology of the colon tissue was observed under a light microscope (CX41, OLYMPUS, Japan).

### 2.5 Transmission Electron Microscope (TEM)

1 mm^3^ of fresh colon tissue was fixed in 2.5% glutaraldehyde, treated with 1% osmic acid, rinsed with 0.1 mol L^-1^ phosphate buffer (PBS), dehydrated, embedded, sliced at 70 nm, stained and examined by transmission electron microscopy (JEM1400, JEOL, Japan), and the images were collected and analysed.

### 2.6 Enzyme-Linked Immunosorbent Assay (ELISA)

According to the instructions of the ELISA kit (Wuhan Colorful Gene Biological Technology Co., Ltd.), the standard was diluted and added sequentially, and the enzyme was added. After sealing the plate with a sealing film, it was incubated at 37°C for 30 min. After liquid preparation and washing, 50 μL of Developer A was added first, followed by 50 μL of Developer B for colour development and 50 μL of Stop Solution to stop the reaction in each well. Read the absorbance at 450 nm with an enzyme-labelled instrument and calculate the levels of D-lactic acid and Diamine oxidase (DAO) in rat serum, IL-1β, IL-6 and IFN-γ in serum and colon from the standard curve.

### 2.7 Western Blot (WB)

The colon was dissolved in radioimmunoprecipitation assay (RIPA) buffer and the protein concentration was determined with bicinchoninic acid (BCA) protein assay kit. 5 x sodium dodecyl sulfate–polyacrylamide gel electrophoresis (5X SDS-PAGE) protein loading buffer was added to the collected protein samples at a ratio of 1∶4, and the protein was denatured by heating in a water bath for 15 minutes. Subsequently, the isolated protein was transferred to polyvinylidene fluoride (PVDF) membrane and blocked with 5% skimmed milk powder for 2 h at room temperature. After that, the membrane was incubated overnight at 4 °C with the corresponding primary antibodies: Claudin 1 (Abcam,ab180158), Occludin (Bioss,bs-10011R), ZO-1 (Bioss,bs-1329R), tryptase (Bioss,bs-2725R), PAR-2 (Abcam,ab180953). The membrane was washed with PBST and incubated with relevant secondary antibodies, including HRP-labeled goat anti-rabbit IgG (1:20000) and HRP-labeled goat anti-mouse IgG (1:20000), for 1.2 h. The luminescent protein was detected with ECL (Thermo) kit. The gray value was measured with Image J software (NIH), and the relative expression was calculated. β-actin was used as an internal reference for calibration.

### 2.8 Immunofluorescence (IF)

The colon of the rat was embedded in paraffin wax and made into 5μm sections. After standardized dewaxing treatment, the sections were sealed with goat serum for 30 minutes. After standardized dewaxing procedures, the slices were repaired with goat serum for 30 minutes. Then, the slices were incubated overnight with the diluted primary antibody: tryptase (1:200, Abcam, ab90582), PAR-2 (1:100, Abcam, ab180953). Goat Anti-Rabbit IgG (FITC) and Goat anti-mouse IgG(CY3) antibodies were added. The slices were stained with DAPI at room temperature for 10 min to perform immunofluorescence studies. All the sections were immediately observed with digital slide scanners (Pannoramic MIDI, 3DHISTECH, Hungary).

### 2.9 RNA Sequencing (RNA-seq)

Total RNA was extracted from the colon tissues using TruSeq (Illumina, USA) according to the manufacturer’s instructions. The purity of the RNA samples was determined using a NanoDrop2000 (Thermo Fisher Scientific, USA). An Agilent 2100 Bioanalyzer (Agilent Technologies, USA) was used to test the integrity of the RNA. The library was sequenced on an Illumina HiSeq 2500 platform (Illumina, USA) using a 2×150 bp double-ended sequencing strategy. These programs were outsourced to Genesky Biotechnologies, Inc. (Shanghai, China). Finally, the lncRNA, miRNA, and mRNA sequencing data of 3 normal samples, 3 IBS-D colon samples and 3 samples after EA were obtained.

The quality of the original sequencing results was evaluated using FastQC software and R language (http://www.bioinformatics.babraham.ac.uk/projects/fastqc/). RIN ≥ 7 was detected by Agilent 2100 Bioanalyzer, which ensured the quality of the sequencing library. The raw reads were filtered using the TrimGalore method (http://www.bioinformatics.babraham.ac.uk/projects/trim_galore/) to obtain the clean reads for subsequent analysis. The filtered clean reads was aligned to the reference database annotation (rat genome selection RGSC6.0/rn6, Jul. version 2014) using the HISAT2 software (https://ccb.jhu.edu/soft-ware/hisat2/index.shtml). Differentially expressed genes were analyzed by DESeq2. Our criteria for screening DE mRNAs, DE lncRNAs and DE miRNAs were a |log2(fold change, FC) | > 1 and *P* < 0.05. And log2(fold change) > 1 was marked as an up-regulated gene, and log2 (fold change) < -1 was marked as a down-regulated gene.

### 2.10 Gene Ontology (GO) and Kyoto Encyclopedia of Genes and Genomes (KEGG)

Eventually, KEGG pathway and GO enrichment analyses were used to identify were conducted to investigate the functions of DE mRNAs. The “enrichplot” R package was used to visualize the results. GO and KEGG pathways with significant DE mRNA enrichment were screened at *P* < 0.05.

### 2.11 lncRNA-miRNA-mRNA ceRNA Network

To ensure the functions of lncRNAs, miRNAs, and mRNAs in the ceRNA network and further improve the reliability of the ceRNA network, we constructed a coexpression network of DE lncRNAs, DE miRNAs, and DE mRNAs. DE lncRNAs and DE miRNAs, DE mRNAs and DE miRNAs targets were predicted using miRanda software and RNA hybrid software, respectively, to analyze the target relationships between lncRNA-miRNA and mRNA-miRNA. The correlation between DE lncRNAs and DE mRNA expression was tested using the cor.test function in R language, and the correlation coefficient Cor > 0 and *P* < 0.05 were screened to obtain the lncRNA and mRNA correlation. Thereafter, the same miRNA was used as a bridge to construct a relationship pair of lncRNA-miRNA-mRNA, and the ceRNA network of lncRNA-miRNA-mRNA was obtained.

### 2.12 Real-Time Quantitative Reverse Transcription PCR (RT-qPCR)

The expression of DE RNAs in rat colon was determined by PCR. Frozen colon tissue was taken, weighed 50-100 mg, 1 ml Trizol was added for digestion, homogenised, and total RNA was extracted using a total RNA extraction kit. According to the instructions, RNA reverse transcription experiment was carried out and the cDNA product was equipped with qPCR reaction system in RNase-free centrifuge tube. All primers were designed using the NCBI database (Table **1**). Using β-actin as an internal reference, relative quantitative analysis of gene expression was performed by 2^-△△CT^.

### 2.13 Statistical Analysis

IBM SPSS23.0 and GraphPad Prism 8 software were used for statistical analysis and graph generation, respectively. All data are expressed as mean ± standard deviation 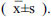 If the data conformed to normal distribution and homogeneity of variance, single-factor analysis of variance was used for multiple groups of data, and LSD was used for analysis of comparison between groups. If not, the data was tested by rank sum. *P*< 0.05 was considered to be statistically significant.

## 3 RESULTS

### 3.1 EA Alleviated Visceral Hypersensitivity and Diarrhoea-predominant Symptoms in IBS-D Rats

**Fig. (2):**
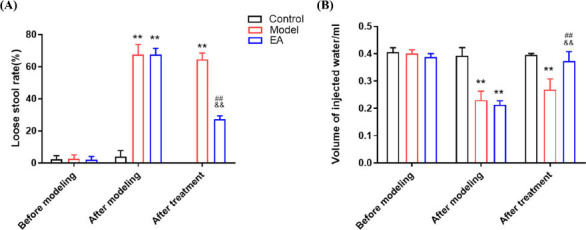
Improvement of symptoms of IBS-D rats by EA. **(A)** The LSR of rats in each group. X axis: the change of experimental timeline, Y axis: changes of the LSR in rats. **(B)** The minimum volume threshold of AWR in rats of each group. X axis: the change of experimental timeline, Y axis: changes of the minimum volume threshold of AWR in rats. Data are presented as mean ± SD. **P < 0.01, *vs.* control group; ^##^*P* < 0.01, *vs.* model group; *^&&^P* < 0.01, *vs.* the same group after modeling.

Before modelling, there were no differences between groups (*P* > 0.05) in the LSR and the amount of water injected (AWR score=3). After modeling, compared with the control group, the LSR in the model group and the EA group increased significantly, and the amount of water injected decreased (*P* < 0.01). After the end of EA, compared with the model group, the LSR in the EA group decreased, while the amount of water injected increased (*P* < 0.01) (Fig. **2A**, **B**).

### 3.2 EA Improved Low-grade Inflammatory Injury in the Colon of IBS-D Rats

**Fig. (3):**
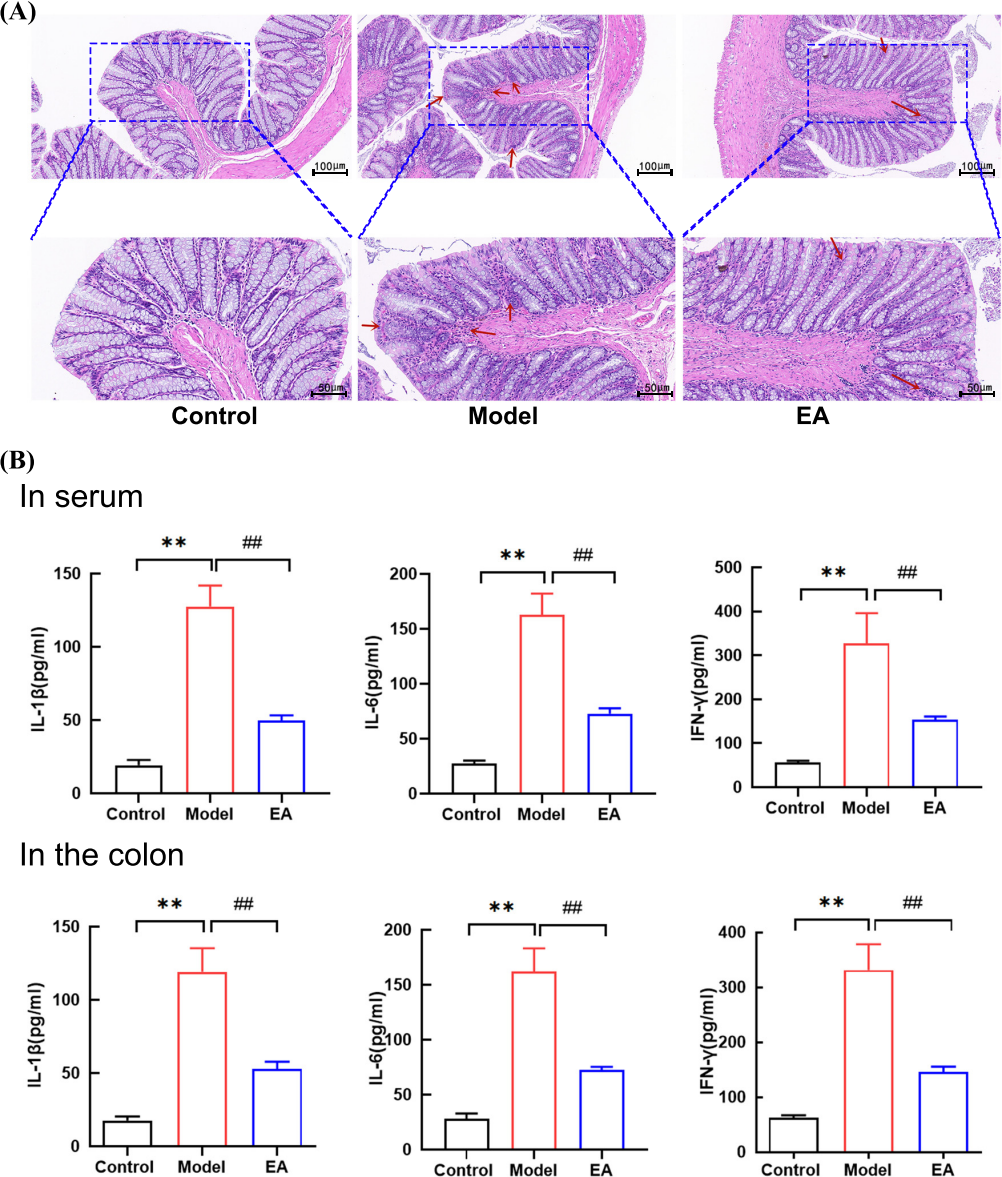
**Effect of EA on inflammatory injury of intestinal mucosa in IBS-D rats. (A)** Results of HE staining in the colon of rats in each group. **(B)** The levels of IL-1β, IL-6 and IFN-γ in serum and colon of three groups of rats. X axis: different groups of rats, Y axis: changes of IL-1β, IL-6 and IFN-γ levels in serum and colon in rats. Data are presented as mean ± SD. **P < 0.01, *vs.* control group; ^##^*P* < 0.01, *vs.* model group.

Histological examination through HE staining revealed that the colon epithelium, lamina propria, and muscularis mucosa in the control group maintained structural integrity, with normal crypt architecture, well-organized glandular arrangement, and evenly distributed goblet cells within the glands. In contrast, the model group exhibited significant pathological changes, characterized by extensive inflammatory cell infiltration in the colonic crypts, prominent nuclear staining, glandular atrophy, and disorganized distribution of goblet cells. Notably, compared to the model group, the EA group showed a reduction in inflammatory cell infiltration and a more organized glandular arrangement. These histological findings suggested that EA effectively improved colonic inflammatory injury in IBS-D rats (Fig. **3A**).

Furthermore, the inflammatory factors IL-1β, IL-6, and IFN-γ in the serum and colon of rats in each group were detected by ELISA. Compared with the control group, the levels of IL-1β, IL-6, and IFN-γ in the model group were significantly increased (*P* < 0.01). In contrast, the levels of IL-1β, IL-6, and IFN-γ in the serum and colon of rats in the EA group decreased compared to the model group (*P* < 0.01), indicating that EA could effectively inhibit the inflammatory response in IBS-D rats (Fig. **3B**).

### 3.3 EA Enhanced Tight Junction Integrity and Preserved the Intestinal Mucosal Barrier in IBS-D Rats

**Fig. (4):**
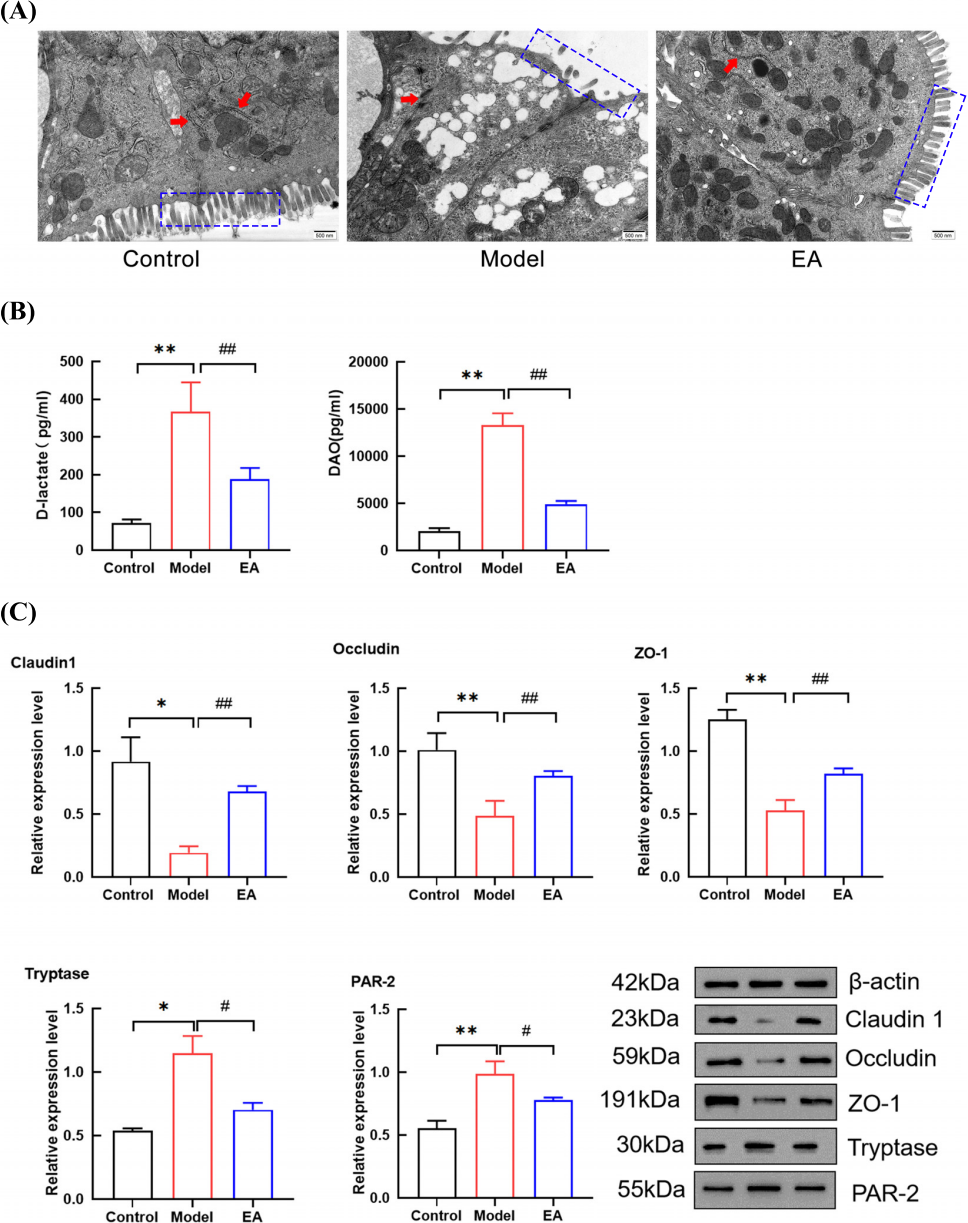
EA can improve the damage of the intestinal mucosal barrier in IBS-D rats. **(A)** Results of TEM in the colon of rats in each group. The red arrow indicated TJ structure and the blue box indicated intestinal villi. **(B)** The levels of DAO and D lactic acid in the serum of three groups of rats. X axis: different groups of rats, Y axis: changes of DAO and D-lactate contents in serum. **(C)** The levels of TJ protein, tryptase and PAR-2 in colon of rats in each group. X axis: different groups of rats, Y axis: changes of TJ protein, tryptase and PAR-2 contents in colon. Data are presented as mean ± SD. *P < 0.05, **P < 0.01, *vs.* control group; ^##^*P* < 0.01, ^#^*P* < 0.05, *vs*. model group.

TEM analysis demonstrated distinct morphological differences among the groups. In the control group, colonic mucosal microvilli exhibited uniform distribution, dense alignment, and structurally intact TJs between adjacent cells. In contrast, the model group displayed marked pathological features: microvilli were reduced in density, irregular in length, and disorganized in arrangement. Additionally, TJ structures were fragmented, gap junctions were abnormally widened, and chromatin vacuolation was prominent. Remarkably, EA restored near-normal microvilli morphology and significantly improved TJ continuity compared to the model group, suggesting a protective effect on intestinal barrier integrity (Fig. **4A**).

Serum D-lactate and DAO, biomarkers of intestinal mucosal barrier damage, were quantified by ELISA. Compared to the control group, the model group showed significantly increased serum levels of D-lactate and DAO (*P* < 0.01). Conversely, EA significantly reduced these biomarkers (*P* < 0.01) (Fig. **4B**), demonstrating its therapeutic potential in alleviating intestinal barrier damage.

To further investigate the molecular mechanism, WB was performed to assess colonic TJ proteins. The model group showed significantly reduced relative expression levels of claudin-1, occludin and zonule -1 (ZO-1) compared to the control group (*P* < 0.05, *P* < 0.01). In contrast, EA significantly upregulated the expression of these proteins compared to the model group (*P* < 0.01) (Fig. **4C**), suggesting that EA restored intestinal barrier integrity through modulation of TJ complexes.

### 3.4 RNA-seq Analysis of Protective Mechanism of EA on Intestinal Mucosal Barrier in IBS-D Rats

#### 3.4.1 Quality Assessment of RNA-seq Data

**Fig. (5):**
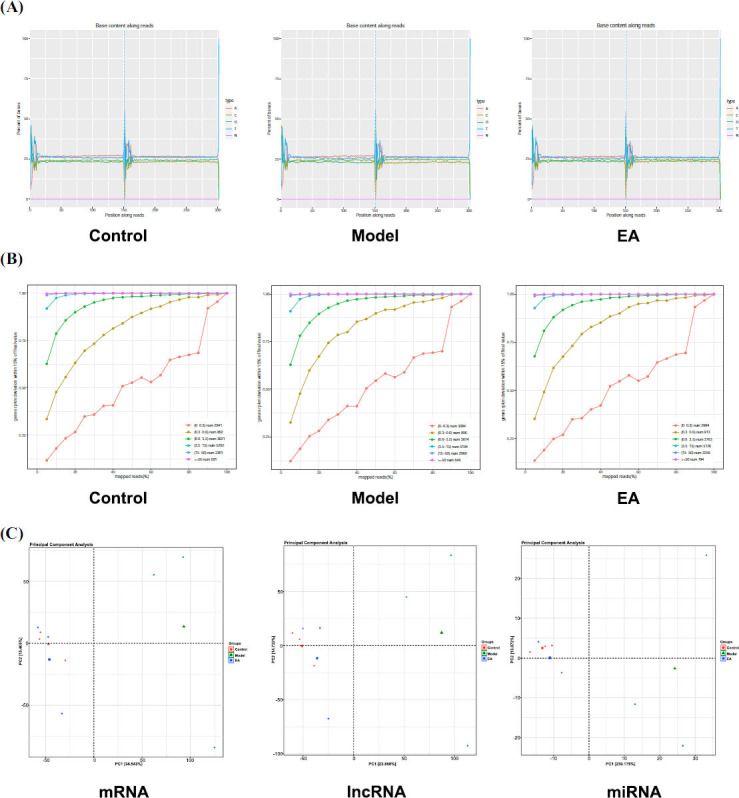
Quality assessment of RNA-seq data. **(A)** Composition distribution plots. The abscissa is the base position of reads, and the ordinate is the proportion of single base. Different colors represent different base types. **(B)** Sequencing saturation plots. The abscissa represents the percentage of effective comparison of reads, and the vertical axis represents the deviation ratio between the expression amount and the final value under this sampling condition. **(C)** PCA of lncRNA, miRNA and mRNA expression in three groups of samples.

Base composition distribution plots demonstrated nearly overlapping A-T and G-C curves, suggesting minimal sequencing bias. The sequencing data exhibited high saturation quality, with sufficient depth to comprehensively cover the majority of expressed genes. Principal component analysis (PCA) revealed distinct clustering patterns among the three groups (Fig. **5A**-**C**). Collectively, these quality control metrics confirm the reliability of the RNA-seq data for downstream bioinformatic analysis.

#### 3.4.2 Identification of Differentially Expressed lncRNA, miRNA, and mRNA

**Fig. (6):**
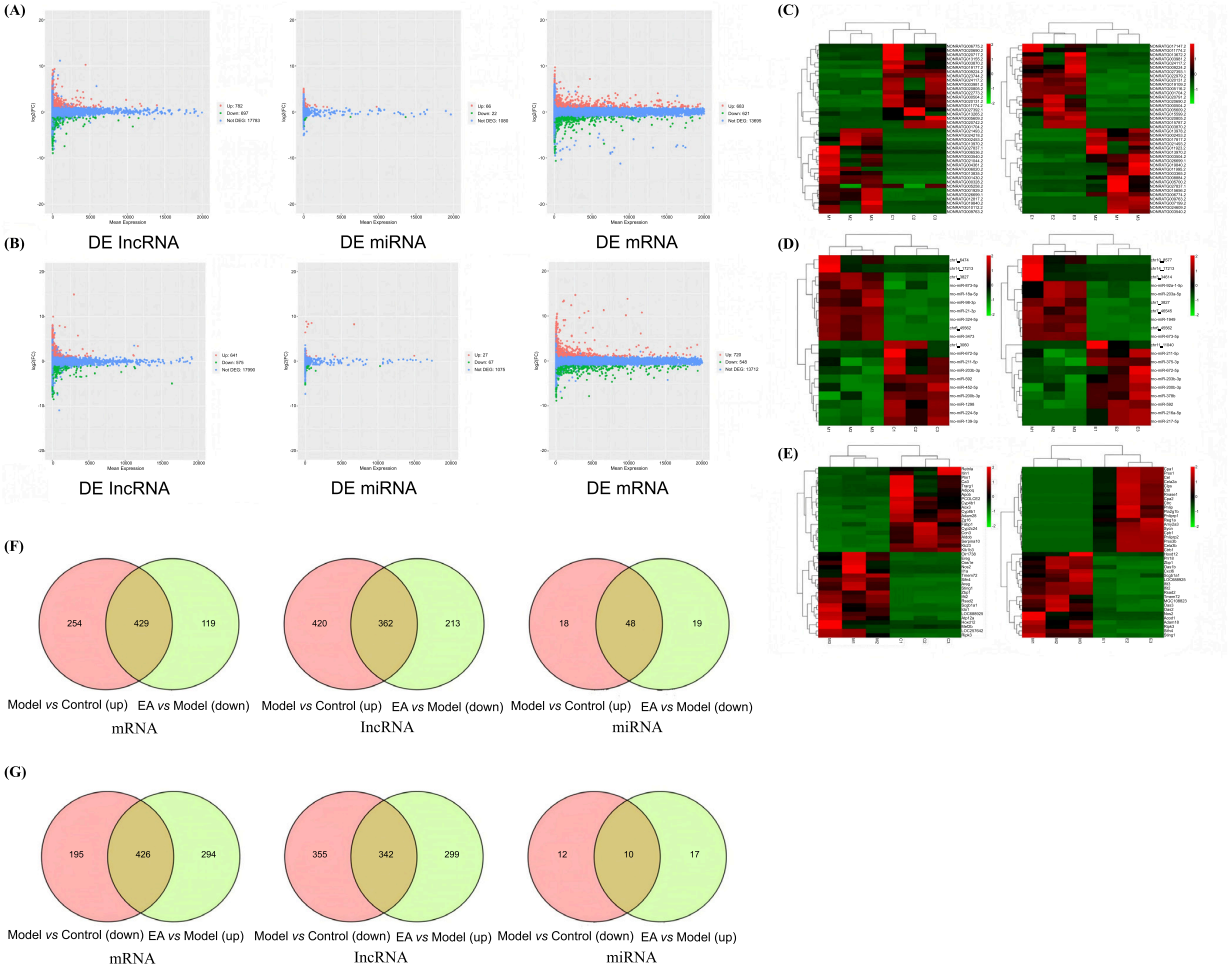
Identification of differentially expressed lncRNA, miRNA, and mRNA. **(A-E)** The valcano plot and heatmap of the DE lncRNAs, miRNAs, and mRNAs (*P* < 0.05, |logFC|>1). A:DE RNAs between model group and control group. B: DE RNAs between EA group and model group. C: The top 20 DE lncRNAs. D: The top 10 DE miRNAs. E: The top 20 DE mRNAs. **(F, G)** Quantity of DE RNAs regulated by EA. F: DE RNAs down-regulated by EA. G: DE RNAs up-regulated by EA.

Comparative transcriptomic analysis revealed extensive dysregulation of non-coding and coding RNAs in IBS-D pathogenesis. In the model group *versus* control, 1,479 statistically significant DE lncRNAs were identified (782 up-regulated, 697 down-regulated), accompanied by 1,304 DE mRNAs (683 up-regulated, 621 down-regulated) and 88 DE miRNAs (66 up-regulated, 22 down-regulated) (Fig. **6A**).

Importantly, EA substantially reversed these changes, with 1,216 lncRNAs (641 up-regulated, 575 down-regulated), 1,268 mRNAs (720 up-regulated, 548 down-regulated) and 94 miRNAs (27 up-regulated, 67 down-regulated) showing differential expression compared to the model group (Fig. **6B**). In addition, the top 20 DE lncRNAs and mRNAs and top 10 DE miRNAs were shown in heatmap (Fig. **6C**-**E**) (Supplementary Table **S1-S6**).

Among these DE RNAs, 429 mRNAs, 362 lncRNAs, and 48 miRNAs were down-regulated by EA (Fig. **6F**). Conversely, 426 mRNAs, 342 lncRNAs, and 10 miRNAs were up-regulated by EA (Fig. **6G**).

#### 3.4.3 Functional Enrichment Analysis of EA-Regulated mRNAs

**Fig. (7):**
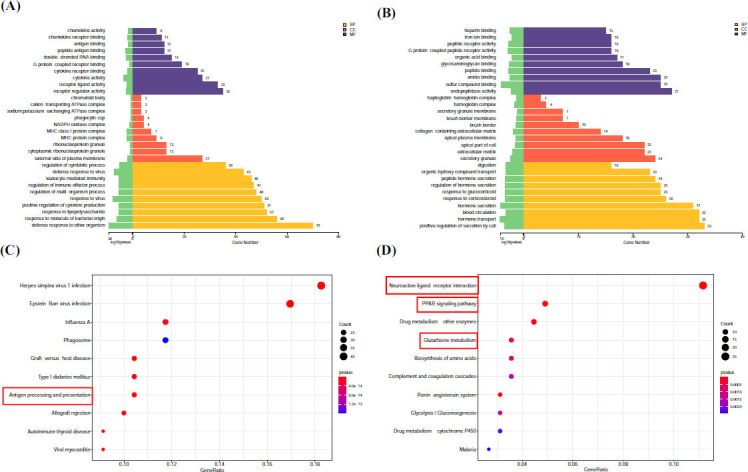
Functional enrichment analysis of EA-regulated mRNAs. **(A, C)** GO and KEGG enrichment analysis of 429 DE mRNAs down-regulated by EA. **(B, D)** GO and KEGG enrichment analysis of 426 DE mRNAs up-regulated by EA.

The function of 429 DE mRNAs down-regulated by EA was further analyzed (Supplementary Table **S7**). The results of GO analysis are shown in Fig. (**7A**). In terms of molecular function (MF), these genes down-regulated by EA were significantly associated with cytokine activity and peptide antigen binding. In terms of cell composition (CC), these genes down-regulated by EA were significantly related to MHC protein complex. In terms of biological processes (BP), these genes down-regulated by EA were involved in positive regulation of response to external stimulus (Fig. **7A**).

In addition, in the GO result analysis of 426 DE mRNAs up-regulated by EA (Supplementary Table **S8**), in terms of BP, these genes were involved in hormone transport and secretion. In terms of CC, these genes were significantly related to secretory granules and extracellular matrix. And In terms of MF, these genes were significantly associated with sulphur compound binding, G protein-coupled peptide receptor activity, and peptide receptor activity (Fig. **7B**).

Furthermore, KEGG pathway analysis revealed distinct mechanistic signatures for EA-mediated transcriptional regulation: 429 down-regulated DE mRNAs enriched in 247 pathway entries, of which 74 were significant (*P* < 0.05) (Supplementary Table **S9**). According to the ranking of P-values, the top 10 signaling pathways were displayed, among which Antigen processing and presentation (rno04612) was closely related to the biological function of MCs (Fig. **7C**). In addition, 426 up-regulated DE mRNAs were enriched in 250 pathway entries, of which 38 were significant (*P* < 0.05) (Supplementary Table **S10**). According to the ranking of P value, the top 10 signaling pathways were displayed, including the Neuroactive ligand-receptor interaction (rno04080), PPAR signaling pathway (rno03320) and Glutathione metabolism (rno00480). The activation of these signaling pathways was also related to the participation of MCs (Fig. **7D**).

#### 3.4.4 The ceRNA Analysis of DE RNAs Altered by EA

**Fig. (8):**
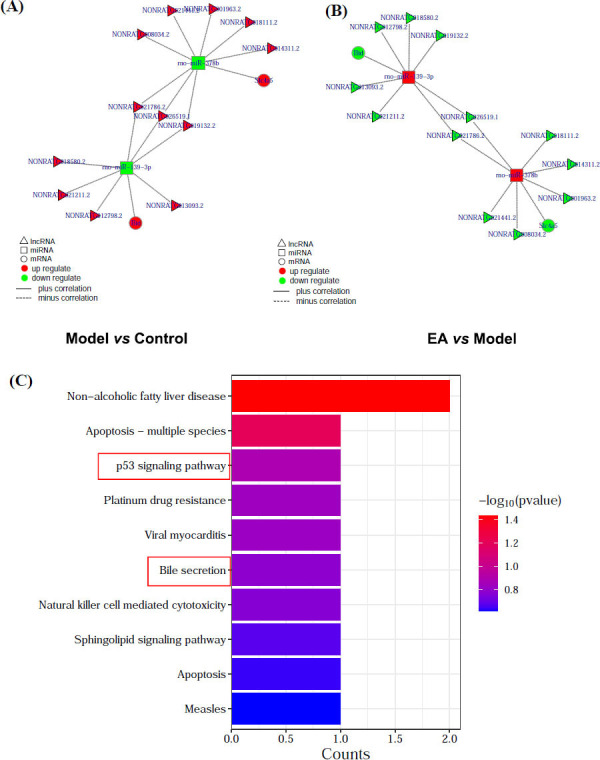
The ceRNA analysis of DE RNAs altered by EA and KEGG enrichment analysis of genes in ceRNA. **(A, B)** ceRNA network. A: Model *vs.* Control; B: EA *vs.* Model. **(C)** KEGG enrichment analysis of *Bid* and *Slc4a5 in* ceRNA.

DE RNAs meeting ceRNA screening criteria across all pairwise comparisons are shown in Table **2**. After the intersection of these ceRNAs, seven DE lncRNAs-miR-139-3p-*Bid* and seven DE lncRNAs-miR-378b-*Slc4a5* were identified (Supplementary Tables **S11**, **S12**). Specifically, EA could restore the damage of intestinal mucosal barrier in IBS-D rats by regulating the ceRNA networks of lncNONRATG013093.2/lncNONRATG018580.2/lncNONRATG026519.1/lncNONRATG021211.2/lncNONRATG019132.2/lncNONRATG021786.2/lncNONRATG012798.2-miR-139-3p-*Bid* and lncNONRATG018111.2/lncNONRATG001963.2/ lncNONRATG008034.2/lncNONRATG021786.2/lncNONR ATG026519.1/ lncNONRATG021441.2/ lncNONRATG014 311.2-miR-378b-*Slc4a5* (Fig. **8A**, **B**).

#### 3.4.5 KEGG Enrichment Analysis of Bid and Slc4a5 in ceRNA Regulated by EA

In the process of EA on intestinal mucosal barrier protection in IBS-D rats, the Bid and Slc4a5 in ceRNA networks of 7DE lncRNAs-miR-139-3p-*Bid* and 7 DE lncRNAs-miR-378b-*Slc4a5* were enriched, with 23 and 1 KEGG pathway respectively (Supplementary Table **S13**). Bid and Slc4a5 were enriched to the top 10 KEGG paths, which were shown in the barplot (Fig. **8C**). Among them, the p53 pathway and Bile secretion might have the participation of MCs in the biological process.

### 3.5 RT-qPCR Validation

**Fig. (9):**
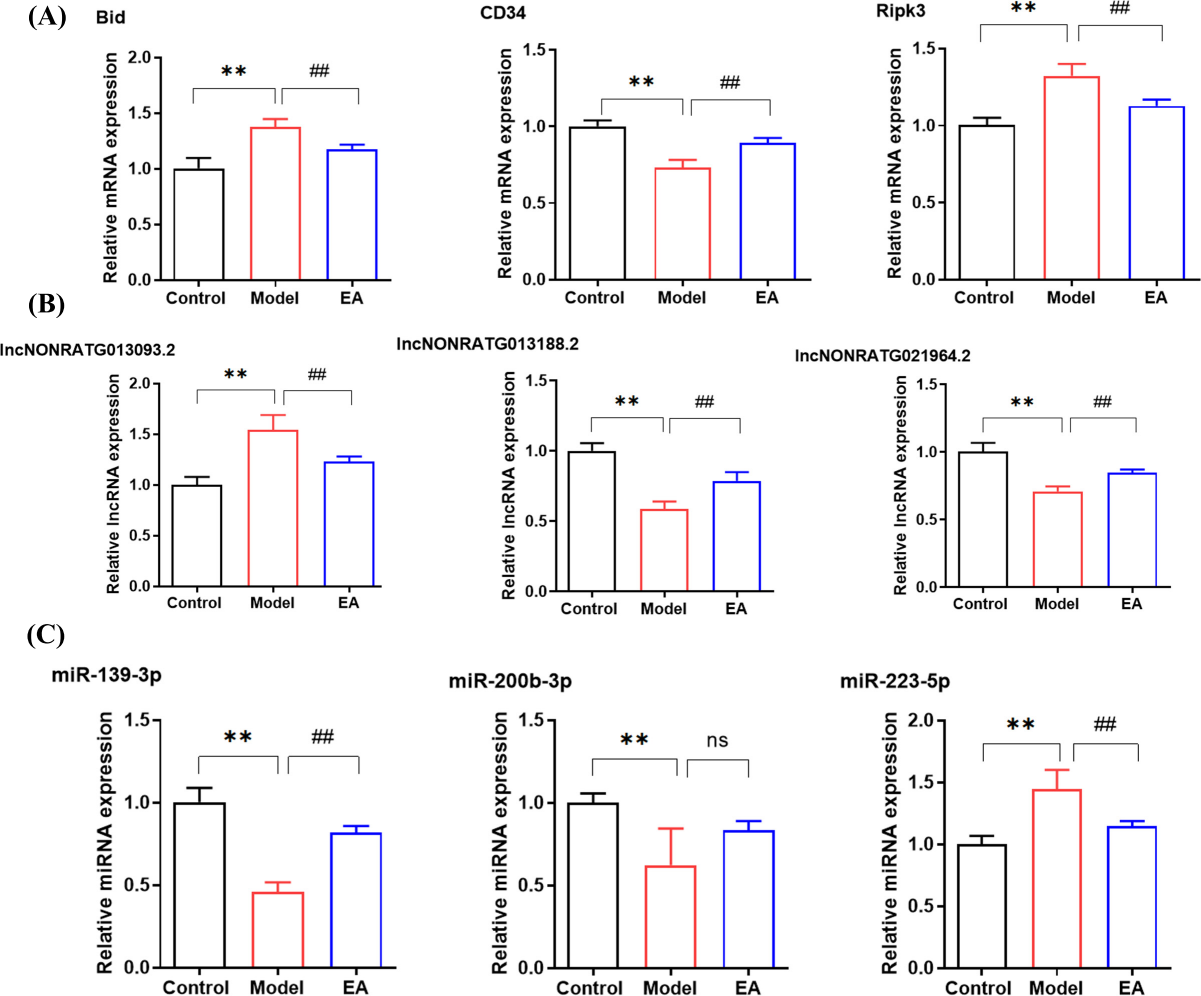
Results of RT-qPCR verification analysis. **(A)** mRNA content of DE mRNA in colon of rats in each group. **(B)** mRNA content of DE lncRNA in the colon of rats in each group. **(C)** miRNA content of DE miRNA in colon of rats in each group. X axis: different groups of rats, Y axis: changes of mRNA and miRNA content of DE RNA in colon. Data are presented as mean ± SD. **P < 0.01, *vs.* control group; ^##^*P* < 0.01, ns represents *P* > 0.05, *vs.* model group.

A subset of DE RNAs was randomly selected for mRNA and miRNA validation. Compared to the control group, the model group exhibited significantly elevated expression levels of *Bid*, *Ripk3*, lncNONRATG013093.2 (mRNAs), and miR-223-5p (miRNA) (*P* < 0.01), whereas *CD34*, lncNONRATG013188.2, lncNONRATG021964.2 (mRNAs), miR-139-3p, and miR-200b-3p (miRNAs) were down-regulated (*P* < 0.01). Following EA, the expression of *Bid*, *Ripk3*, lncNONRATG013093.2, and miR-223-5p was significantly reduced compared to the model group (*P* < 0.01), while *CD34*, lncNONRATG013188.2, lncNONRATG021964.2, miR-139-3p, and miR-200b-3p showed increased expression (*P* < 0.01, *P* > 0.05) (Fig. **9**). The concordant trends between RNA-Seq and RT-qPCR results validated the reliability of the transcriptomic data.

### 3.6 EA Reduced the Levels of Tryptase and PAR-2 in the Colon of IBS-D Rats

The change in tryptase levels reflects the activation state of the MCs. As a protease activation receptor, PAR-2 activation mainly depends on trypsin and tryptase released from MCs after activation. Compared with the control group, the levels of tryptase and PAR-2 in the model group increased significantly (*P* < 0.05, *P* < 0.01). Compared with the model group, the levels of tryptase and PAR-2 in the EA group decreased (*P* < 0.05) (Fig. **4C**).

**Fig. (10):**
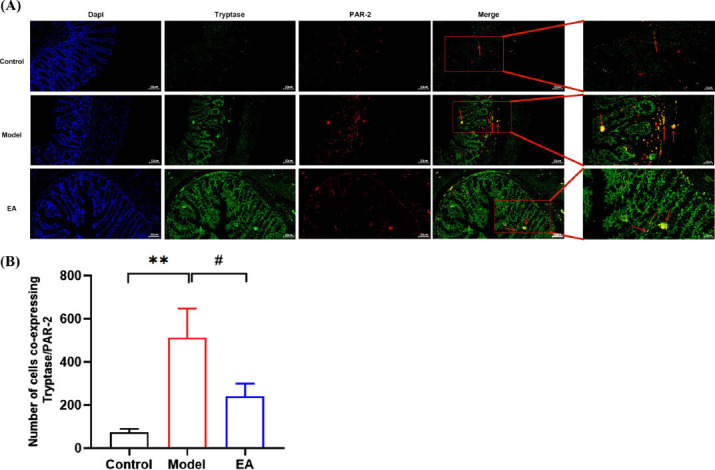
**(A)** Analysis of the results of tryptase /PAR-2 co-expression cells in three groups. **(B)** X axis: different groups of rats, Y axis: changes in the number of tryptase /PAR-2 co-expression cells in colon. Data are presented as mean ± SD.**P < 0.01, *vs.* control group; ^#^*P* < 0.05, *vs.* model group.

Furthermore, tryptase and PAR-2 in the colon tissues of rats were used in the immunofluorescence technique. PAR-2 was labelled with red fluorescence and tryptase with green fluorescence. Both were stained on the cell membrane, and the co-expressing cells showed orange fluorescence. Image J was used to count the co-expressed cells. Compared to the control group, the number of tryptase/PAR-2 co-expressed cells increased in the model group (*P* < 0.01). Compared with the model group, the number of tryptase/PAR-2 co-expression cells in the EA group decreased (*P* < 0.05) (Fig. **10A**, **B**). These results suggested that EA could inhibit MC activation.

## 4 DISCUSSION

As a therapeutic modality in traditional Chinese medicine, EA enhances conventional acupuncture by integrating continuous electrical stimulation at controlled intensities. This approach has sedative, analgesic and meridian-regulating effects and has been widely accepted in the clinical management of various pathological conditions [36-38]. While EA has demonstrated clinical efficacy in alleviating IBS-D symptoms [39], the molecular mechanisms underlying its therapeutic effects, particularly those mediated by ceRNA networks, remain poorly characterised. Therefore, we used RNA-seq to detect changes in the expression of lncRNA, miRNA and mRNA in the colonic tissues of IBS-D rats before and after EA, screened the ceRNA network, and investigated the protective mechanism of EA on the intestinal mucosal barrier at the transcriptome level. In this research, the LSR decreased and the minimum volume threshold of AWR increased after EA, indicating that EA could alleviate visceral hypersensitivity and diarrhoea symptoms in IBS-D rats, which was consistent with the clinical application of EA to improve abdominal pain and diarrhoea symptoms in IBS-D patients [40, 41].

Recently, an increasing number of studies have identified an important role for MCs in the progression of functional gastrointestinal diseases [11, 42, 43]. In the pathological process of IBS, activated MCs release tryptase through degranulation, which activates PAR-2 on epithelial cells [44, 45]. This subsequently activates NF-κB, leading to the release of inflammatory mediators. These mediators induce redistribution of TJ proteins on cell membranes, ultimately compromising the intestinal mucosal barrier [46]. Mucosal barrier dysfunction is mainly manifested by increased permeability of the intestinal mucosal epithelium, which can lead to intestinal bacteria and other antigenic substances crossing the epithelium and entering the mucosa, causing an enhanced immune response and triggering or exacerbating inflammation [47]. We found that compared with the control group, the levels of IL-1β, IL-6, and IFN-γ were increased in the colon and serum of rats in the model group, along with a large number of inflammatory cells infiltrated in the colonic crypts. However, after EA, the levels of pro-inflammatory factors were significantly reduced in serum and colon, and the inflammatory cells in colon were reduced and the glands were aligned. These results suggested that EA could improve low-grade inflammation in the colon of IBS-D rats. Our results were consistent with related studies [48, 49].

In addition, the TJ in the intestinal epithelial barrier, which separates the internal environment from the external environment of the intestine, is the key structure that regulates molecular transport between cells, and its integrity is one of the important factors in ensuring the function of the intestinal mucosal barrier [50]. TJs are composed of transmembrane proteins, including Claudin, Occludin and ZO-1 [51, 52]. In the study of IBS-D, the expressions of the proteins ZO-1, occludin and claudin-1 were found to be decreased in the colonic tissue of IBS-D patients [9], and animal experiments also confirmed that the damage to the intestinal mucosal barrier in rats was characterised by the decreased expression of these proteins [53]. DAO is a high-concentration enzyme found in the intestinal mucosa of human beings and other mammals. It is difficult for DAO to enter the systemic circulation when the intestinal mucosal barrier is intact, so the level of serum DAO can reflect the degree of intestinal mucosal injury [54]. D-lactic acid is produced by microbial metabolism in the intestine. When the upper layer of the intestinal mucosa is damaged and falls off, and the permeability of the intestine increases, D-lactic acid can enter the bloodstream through the damaged intestinal mucosa [55]. Our findings demonstrated that model group rats exhibited significantly elevated serum levels of DAO and D-lactic acid, accompanied by reduced colonic expression of Claudin-1, Occludin, and ZO-1. TEM further revealed structural compromise of TJs and disorganized intestinal villi. In contrast, EA effectively mitigated these pathological alterations by substantially decreasing serum DAO and D-lactic acid, upregulating the expression of key TJ proteins, and restoring the architectural integrity of intestinal epithelium. These findings suggested that EA could improve the intestinal mucosal barrier damage in IBS-D rats through modulation of TJ protein dynamics and structural stabilization.

The RNA-seq revealed that EA could down-regulate 429 mRNAs, 362 lncRNAs and 48 miRNAs. Concurrently, 426 mRNAs, 342 lncRNAs and 10 miRNAs were up-regulated by EA. Furthermore, GO functional enrichment analysis revealed that the regulatory effects of EA in IBS-D rats were mainly mediated through modulation of a series of defence responses (*e.g.*, against viruses and bacteria), involvement in the regulation of hormone secretion and transport, and influence on cytokine function. It is well known that the gut contains a large number of intestinal flora, such as *Bifidobacteria*, *Lactobacillus*, *Escherichia coli*, *Enterococcus* and so on. These changes in the flora affect the immune function of the gut [56]. The 16S rDNA technique was used to analyse the effect of acupuncture on the relative abundance and diversity of the intestinal flora in IBS-D rats. It was found that acupuncture could treat diarrhoea and alleviate visceral hypersensitivity by regulating the relative abundance of intestinal flora [57-59]. It was also reported that EA could improve the integrity of IBS-D intestinal mucosal barrier by regulating the composition of intestinal flora and increasing the expression of Occludin and ZO-1 [49]. Ruminococcus gnavus has been proven to induce and aggravate IBS-D by stimulating the production of peripheral 5-hydroxytryptamine (5-HT) [60]. However, some studies have confirmed that EA can improve the diarrhea symptoms of IBS-D rats by reducing the content of 5-HT in colon tissue and feces of IBS-D rats [61]. Furthermore, the influence of gut microbiota on intestinal function is often dependent on the action of hormones. There are at least 14 different gut hormones secreted by endocrine cells scattered between the epithelial cells facing the gut lumen [62]. Almost all of these hormones are peptides, which play a role in intestinal-brain communication [63]. Brain-gut peptides have dual functions as hormones and neurotransmitters, and studies related to IBS-D have found that the expression levels of 5-HT, vasoactive intestinal peptide (VIP) and surfactant proteins (SP) were increased due to activation of SCF/c-kit signaling pathway, which manifested as visceral pain sensitivity of IBS-D [64]. These reports provided evidence support for our GO analysis results.

Based on the analysis of the CC results obtained by GO, some researchers found that the major histocompatibility complex (MHC) was involved in antigen presentation and immune recognition [65]. Nicotinamide adenine dinucleotide phosphate hydride (NADPH) was an oxidoreductase that was involved in regulating many aspects of innate and adaptive immunity and also played an important role in the innate immune defense of the epithelial barrier [66]. NADPH oxidase 4 (NOX4) regulates the M1 polarization of intestinal macrophages through ROS, which leads to mucosal barrier injury to promote IBD progression [67]. The potential cytokine activity, peptide antigen, peptide receptor activity, and G protein-coupled peptide receptor activity of mRNAs altered by EA were significantly demonstrated in MF. The relationship between the function of these molecules and the intestinal mucosal barrier has also been studied. Protease-activated receptor (PAR) expressed in gastrointestinal tract is a unique G protein-coupled transmembrane receptor, especially PAR-1 and PAR-2, which regulate intestinal permeability and are the main factors affecting intestinal physiology and inflammatory response [44].

In the KEGG enrichment analysis of mRNAs changed by EA, the signal pathways such as antigen processing and presentation, neuroactive ligand-receptor interaction, PPAR signaling pathway and glutathione metabolism were all related to degranulation of MCs. Specifically, MCs have been shown to act as antigen-presenting cells, regulate both local and systemic inflammatory responses, and shape innate and adaptive immune responses [68]. As was mentioned previously, MC activation in the intestinal mucosa has been found in different categories of IBS. And 5-HT receptor is enriched in the neuroactive ligand receptor interaction pathway, which is related to intestinal dysfunction and visceral sensitivity of IBS-D [69, 70]. In addition, the expression of serotonin reuptake transporter is regulated by gut microbiota through mast cell-prostaglandin E2 [71]. Recent research has identified peroxisome proliferator-activated receptor-β/δ (PPARβ/δ) as a pivotal regulator of mast cell phenotype [72]. Concurrently, peroxisome proliferator-activated receptor γ (PPAR-γ) has been demonstrated to attenuate allergic responses by impeding mast cell degranulation [73]. Glutathione (GSH) is known to be a powerful antioxidant that can scavenge intracellular reactive oxygen species (ROS). Antigen stimulation raises reactive oxygen species (ROS) production from NADPH oxidases and mitochondria, leading to MCs degranulation [74]. In our previous study, it was confirmed that EA inhibited the activation of Tryptase/PAR-2/MLCK pathway by inhibiting MC degranulation, which reduced the phosphorylation level of MLC and the remodeling of tight junction protein, thus improving the integrity of the intestinal mucosal barrier in IBS-D rats [40]. Therefore, combined with the results of KEGG enrichment, we speculated that EA might inhibit MC degranulation by modulating these signalling pathways, which in turn exerted a protective effect on the intestinal mucosal barrier in IBS-D rats. To confirm this speculation, we observed the protein changes associated with MC degranulation and found that the protein levels of tryptase and PAR-2 were elevated and the number of tryptase/PAR-2 co-expressing cells increased in the model group. In contrast, the protein levels of tryptase and PAR-2 and the number of tryptase/PAR-2 co-expressing cells decreased in the colon of rats in the EA group compared with the model group.

The ceRNA theory of lncRNA-miRNA-mRNA is interesting as it suggests that certain lncRNAs have miRNA response elements (MREs) similar to those in mRNAs. Growing evidence supports that lncRNAs can act as ceRNAs or miRNA sponges, which could competitively bind to miRNAs and tend to regulate the expression levels of target mRNAs [75]. Finally, we found that EA may inhibit the activation of MCs and improve the inflammatory damage of the intestinal mucosal barrier in IBS-D rats by regulating the ceRNA networks of 7 DE lncRNAs-miR-139-3p-*Bid* and 7 DE lncRNAs-miR-378b-*Slc4a5*. MiR-139-3p involved in these ceRNA networks, is a key tumor suppressor gene which plays a role in regulating cell responses to various stresses and inhibiting carcinogenic signaling pathways [76]. MiR-378b is encoded by the miR-378 gene family, and miRNAs in this family are involved in the regulation of fat production [77]. In addition, miR-378 can regulate the cytotoxicity of natural killer cells, suggesting that miR-378 may be involved in mediating innate and adaptive immune processes [78]. BH3-interacting domain death agonist (Bid) is a pro-apoptotic member of the B-cell lymphoma 2 (Bcl-2) protein family. Its regulatory role in apoptosis is well known [79], but Bid is also key to inflammatory response and immune regulation [80]. Bid was enriched in the p53 signaling pathway, which may affect the integrity of the intestinal mucosal barrier by regulating the apoptosis of intestinal epithelial cells [81]. NBCe2(Slc4a5) is a co-transporter of Na, HCO3, which is currently considered to be related to arterial hypertension and metabolic acidosis [82]. Some researchers reported that metabolic acidosis is an important complication of diarrhea [83]. In other words, Slc4a5 may be associated with diarrhea symptoms in IBS-D. It had been demonstrated that Slc4a5 was implicated in Bile secretion pathway. Research has identified a role for MCs in regulating the catheter reaction and intestinal inflammation in cholestasis, with these effects being mediated through farnesol X receptor signal transduction [84]. In a study on IBS-D, it was also confirmed that bile acids induced visceral hypersensitivity through mucosal MCs to nociceptors, which involved the Farnesoid X receptor/nerve growth factor/transient receptor potential vanillin 1 axis [85].

In summary, EA ameliorated the damage of the intestinal mucosal barrier in IBS-D rats through multi-system and multi-target mechanisms. This therapeutic effect was mediated by modulating ceRNA networks-specifically, the 7DE lncRNAs-miR-139-3p-*Bid* and 7 DE lncRNAs-miR-378b-*Slc4a5* -which inhibited MCs degranulation, reduced pro-inflammatory cytokine production (IL-1β, IL-6, IFN-γ), downregulated serum DAO and D-lactic acid levels, and upregulated the expression of TJ proteins (Claudin1, Occludin, ZO-1). These findings underscore that EA achieved its protective effects on the intestinal mucosal barrier *via* systemic, integrative regulation. Our study provided valuable insights for further exploration of EA in IBS-D intervention and intestinal barrier protection.

## CONCLUSION

In conclusion, through RNA-seq, it was found that EA had a significant effect on gene expression in the colon of IBS-D rats, which may be involved in the inhibition of MC degranulation through the regulation of antigen processing and presentation, neuroactive ligand-receptor interaction, PPAR signaling pathway, glutathione metabolism and other signaling pathways. The ceRNA networks composed of 7 DE lncRNAs-miR-139-3p-*Bid* and 7 DE lncRNAs-miR-378b-*Slc4a5* was a potential target for EA to treat IBS-D rats and improved intestinal mucosal barrier damage. The treatment of IBS-D by EA highlighted its overall adjustment ability of multi-system, multi-level and multi-target, which was proved by RNA-seq. These findings provided a new perspective for further investigation and revealed the mechanism of EA.

## STUDY LIMITATIONS

While our results align with existing reports, certain limitations remain. These findings require further experimental validation to determine their universal applicability. In the future, we will verify the related pathways of EA regulation *in vivo* to determine their specific molecular regulation mechanism in the intervention of EA in IBS-D to protect intestinal mucosal barrier injury. And we will further clarify the targeted binding relationship between ceRNA. And adenovirus was used to transfect rats *in vivo* to elucidate how EA regulates a certain lncRNA-miRNA-mRNA axis in ceRNA with a clear targeted binding relationship to improve the intestinal mucosal barrier injury in rats, and to provide more experimental evidence for this study on the effect of EA on IBS-D rats based on RNA-seq.

## Figures and Tables

**Table 1:** Primer sequences.

**Gene**	**Amplicon Size (bp)**	**Forward Primer (5'→3')**	**Reverse Primer (5'→3')**
β-actin	150	CCCATCTATGAGGGTTACGC	TTTAATGTCACGCACGATTTC
U6	94	CTCGCTTCGGCAGCACA	AACGCTTCACGAATTTGCGT
Bid	171	AGGGACTTGGTTAGAAATGAGA	CACCTGGAAATAGGGAGACG
Ripk3	117	GAACGCACCAAATCCAATGA	TTCTTGGGAAAGGCAGTTCT
CD34	109	GATTGCATTGGTTACCTCGG	GTCTTCACCCAGCCTTTCT
miR-223-5p	67	ACACTCCAGCTGGGCCTCTGGGCCCTTCC	TGGTGTCGTGGAGTCG
miR-223-5p-RT		CTCAACTGGTGTCGTGGAGTCGGCAATTCAGTTGAGCAACTC	
miR-139-3p	66	ACACTCCAGCTGGGTGGAGACGCGGCCCTG	TGGTGTCGTGGAGTC
miR-139-3p-RT		CTCAACTGGTGTCGTGGAGTCGGCAATTCAGTTGAGCTCCAA	
miR-200b-3p	67	ACACTCCAGCTGGGTAATACTGCCTGGTAAT	TGGTGTCGTGGAGTC
miR-200b-3p-RT		CTCAACTGGTGTCGTGGAGTCGGCAATTCAGTTGAGGTCATC	
NONRATG013093.2	93	TTGAGGACTGGCCGTTTTAT	ACTGCACAGTGTCCCTTTTA
NONRATG013188.2	142	TGTTCAAGGGAAGAAATCAAGG	ATGTGGCTCAAGTAATCGCA
NONRATG021964.2	173	CTTTAGGATGGCTTTTGGGC	AGACTGCAACACCAACTGTA

**Table 2:** The results of ceRNA between three groups of pairwise comparison.

**Group**	**Up-Down-Up**	**Down-Up-Down**
Model group *vs.* Control group	63	485
EA group *vs.* Model group	182	296

## Data Availability

The data used and analyzed to support the findings of this study will be available from the corresponding author upon request.

## LIST OF ABBREVIATIONS

AWR: Abdominal Reflex
ceRNA: Competing Endogenous RNA
CRD: Colorectal Balloon Distention
DAO: Diamine Oxidase
DE RNAs: Differentially Expressed RNAs
EA: Electroacupuncture
IBS-D: Diarrhoea-predominant Irritable Bowel Syndrome
lncRNA: Long Non-coding RNAs
LSR: Loose Stool Rate
MC: Mast Cell
miRNA: microRNA
mRNA: Messenger RNA
NMS: Neonatal Maternal Separation
PAR-2: Protease-Activated Receptor 2
RNA-seq: RNA Sequencing
RS: Restrain Stress
TJ: Tight Junction
ZO-1: Zonule -1
