# Supplementary material for: Effect of Electroacupuncture on Intestinal Mucosal Barrier in IBS-D Rats: Analysis Based on RNA-seq
Source: Comb Chem High Throughput Screen. 2025 May 27;29(2):272–90. doi: 10.2174/0113862073395229250513074835 (PMC13223427; doi:10.2174/0113862073395229250513074835)
Supplement: Supplementary file 1 [file CCHTS-29-2-272_SD1.pdf]

Supplementary Material

Effect of Electroacupuncture on Intestinal Mucosal Barrier in IBS-D Rats: Analysis Based on RNA-seq

Jingru Ruan[1], Jingwei Zhu[2], Kuiwu Li[3], Ziye Wang[2], Ting Wang[2], Xiaoyu Han[3], Xiaomin Li[3], Yucheng Fang[2], Xiaoge Song[4] and Haoran Chu[2,5,*]

[1]College of Traditional Chinese Medicine, Anhui University of Traditional Chinese Medicine, 350 Longzihu Road, Hefei 230012, Anhui, China; [2]College of Second Clinical Medical, Anhui University of Traditional Chinese Medicine, 300 Shouchun Road, Hefei, 230061, Anhui, China; [3]College of First Clinical Medical, Anhui University of Traditional Chinese Medicine, 350 Longzihu Road, Hefei, 230012, Anhui, China;[4] Institute of Acupuncture and Meridians, Anhui University of Traditional Chinese Medicine, 103 Meishan Road, Hefei 230038, Anhui, China; [5]Institute of Clinical Acupuncture and Moxibustion, Anhui Academy of Traditional Chinese Medicine, 103 Meishan Road, Hefei 230038, Anhui, China

Support Table 1. The top 20 mRNAs with significant differential fold change among the up-regulated and down-regulated genes between the model group and the control group.

Gene	log2FoldChange	p value	type
Nos2	7.148723032	9.92191E-13	Up
Scgb1a1	5.483226963	5.01644E-12	Up
Ido1	5.107890301	7.77388E-34	Up
Hoxd12	4.958937163	6.1042E-12	Up
LOC257642	4.550263503	6.21839E-11	Up
Oas1e	4.549380012	9.98481E-08	Up
LOC688925	4.377995868	6.30039E-37	Up
Slfn4	4.249325785	1.51109E-80	Up
Tmem72	4.218908918	7.25546E-20	Up
Atp12a	4.173433249	0.001496951	Up
Zbp1	4.064124687	1.12843E-62	Up
Ripk3	4.043490708	1.27135E-36	Up
Ifit2	3.921436417	9.8185E-50	Up
Areg	3.835538663	2.81044E-37	Up
Il1a	3.790647468	4.33071E-06	Up
Olr1738	3.78616079	4.54282E-07	Up
Sting1	3.718104924	1.06219E-35	Up
Ereg	3.692221281	1.19991E-17	Up
Rsad2	3.69204865	1.33669E-38	Up
Mef2b	3.667694876	0.028012501	Up
Cyp2c24	-6.890354543	6.20952E-70	Down
Cyp8b1	-5.752979184	9.48086E-41	Down

Aldob	-4.76944061	4.56966E-07	Down
Retnla	-4.721766281	0.001007287	Down
Kb23	-4.643492698	3.31478E-15	Down
Ca3	-4.435901969	0.000140881	Down
Fabp1	-4.175755911	0.002632731	Down
Trarg1	-4.16507226	3.69278E-09	Down
Adipoq	-4.001436321	0.000313693	Down
Itln1	-3.988491214	0.001907972	Down
Klk1b3	-3.971301948	1.92822E-47	Down
Apob	-3.923559345	5.01541E-07	Down
Adam28	-3.846346281	1.55202E-22	Down
Serpina10	-3.798499458	2.25215E-12	Down
Cyp4b1	-3.720151449	8.6516E-25	Down
Plin1	-3.718252699	0.000254891	Down
Zg16	-3.623039191	1.33413E-38	Down
Aox3	-3.607397241	7.38379E-06	Down
PCOLCE2	-3.558388464	5.27113E-16	Down
Ccn3	-3.448089229	5.69823E-08	Down

After comparing the model group with the control group, the P value <0.05, and according to the difference type given by log2FoldChange, when log2FoldChange>1 is marked as UP, it means up-regulated gene. Log2FoldChange<-1 is marked as Down, which means down-regulated gene.

Support Table 2. The top 20 mRNAs with significant differential fold change among the up-regulated and down-regulated genes between the electroacupuncture group and the model group.

**Gene**	**log2FoldChange**	**p value**	**type**
Clps	12.38427912	7.27277E-44	Up
Cela3b	12.23862646	2.13118E-34	Up
Cpa1	12.01719922	4.49595E-65	Up
Prss3b	11.97643826	1.59951E-34	Up
Prss1	11.94509374	4.4885E-34	Up
Ctrb1	11.78002315	9.31255E-37	Up
Reg1a	11.76332728	7.97606E-46	Up
Cpb1	11.69912835	2.90035E-41	Up
Amy2a3	11.48030022	1.08117E-36	Up
Pla2g1b	11.47308045	7.56145E-38	Up
Sycn	11.47229462	2.88203E-52	Up
Pnliprp1	11.45688616	2.39619E-43	Up
Cpa2	11.44860271	2.9158E-35	Up
Cel	11.42843993	4.01476E-36	Up
Cela2a	11.3411983	1.82578E-56	Up
Pnlip	11.24820974	8.2653E-32	Up

Pnliprp2	11.20622377	1.90627E-93	Up
Rnase1	10.66979104	8.30889E-38	Up
Ctrl	10.28778586	1.59221E-95	Up
Ctrc	9.577665701	8.6262E-113	Up
Nos2	-7.823412004	6.55634E-14	Down
Acod1	-5.925546962	1.43757E-10	Down
Cxcl6	-5.396053395	2.04741E-21	Down
Zbp1	-4.650390733	1.43772E-20	Down
Ifit2	-4.448292252	8.91275E-31	Down
Slfn4	-4.409439147	1.09901E-05	Down
Oas3	-4.188460839	1.79939E-05	Down
LOC688925	-4.156359826	2.49651E-08	Down
Rsad2	-4.032208212	1.65974E-08	Down
Ifit3	-4.007450283	1.62098E-18	Down
Prr18	-3.921715091	0.000220148	Down
Hoxd12	-3.913360003	6.15468E-07	Down
Adam18	-3.898977108	1.73534E-06	Down
Oas2	-3.865971002	0.002350689	Down
Tmem72	-3.8252386	5.95412E-18	Down
Sting1	-3.818071868	1.63846E-22	Down
Ripk3	-3.745484479	4.05794E-27	Down
Scgb1a1	-3.419406086	0.004205099	Down
Oas1b	-3.3464701	0.002226576	Down
MGC108823	-3.337785653	9.55494E-15	Down

After comparing the electroacupuncture group with the model group, the P value <0.05, and according to the difference type given by log2FoldChange, when log2FoldChange>1 is marked as UP, it means up-regulated gene. Log2FoldChange<-1 is marked as Down, which means down-regulated gene.

Support Table 3. The top 20 lncRNAs with significant differential fold change among the up-regulated and down-regulated genes between the model group and the control group.

Gene	log2FoldChange	p value	type
NONRATG010112.2	10.22475741	2.31617E-10	Up
NONRATG001430.2	9.324684654	1.59732E-26	Up
NONRATG021493.2	8.645777365	1.69788E-11	Up
NONRATG027837.1	7.214332671	4.75228E-15	Up
NONRATG009763.2	6.981521263	3.9341E-06	Up
NONRATG006536.2	6.594388461	5.51497E-06	Up
NONRATG003540.2	5.995629177	3.00916E-06	Up
NONRATG024218.2	5.739601373	9.51197E-07	Up
NONRATG019840.2	5.711780139	3.61451E-07	Up
NONRATG021044.2	5.683185483	1.70944E-06	Up

NONRATG026699.1	5.53393845	3.89848E-42	Up
NONRATG000328.2	5.359169212	2.10583E-48	Up
NONRATG002453.2	5.330017925	2.60927E-19	Up
NONRATG004361.2	4.9335652	5.35548E-05	Up
NONRATG006020.2	4.89881096	5.33599E-08	Up
NONRATG013970.2	4.892085207	3.44598E-09	Up
NONRATG005258.2	4.761926669	3.18887E-12	Up
NONRATG013835.2	4.328604271	7.74099E-37	Up
NONRATG012817.2	4.124072659	1.14013E-14	Up
NONRATG001929.2	4.079861838	1.32508E-17	Up
NONRATG003981.2	-7.424129005	2.08975E-06	Down
NONRATG020742.2	-6.210352874	1.37833E-05	Down
NONRATG020805.2	-5.539815545	0.000142994	Down
NONRATG006775.2	-4.81051523	0.002133025	Down
NONRATG003870.2	-4.771405637	2.6315E-22	Down
NONRATG005609.2	-4.716728358	0.000274862	Down
NONRATG020690.2	-4.226481685	0.00094714	Down
NONRATG020131.2	-3.909782991	3.89447E-20	Down
NONRATG027392.1	-3.830491583	3.7371E-09	Down
NONRATG009224.2	-3.592405766	4.26639E-08	Down
NONRATG011774.2	-3.588721088	3.70317E-05	Down
NONRATG000504.2	-3.516908703	0.01055601	Down
NONRATG019177.2	-3.489681445	2.71281E-12	Down
NONRATG020717.2	-3.408845478	0.003205678	Down
NONRATG023744.2	-3.382500243	1.31091E-05	Down
NONRATG013155.2	-3.327533167	0.00193502	Down
NONRATG001704.2	-3.28161903	0.000309651	Down
NONRATG022773.2	-3.186722088	1.34503E-05	Down
NONRATG013265.2	-3.16295246	0.003504211	Down
NONRATG024117.2	-3.156903149	8.843E-05	Down

After comparing the model group with the control group, the P value <0.05, and according to the difference type given by log2FoldChange, when log2FoldChange>1 is marked as UP, it means up-regulated gene. Log2FoldChange<-1 is marked as Down, which means down-regulated gene.

Support Table 4. The top 20 lncRNAs with significant differential fold change among the up-regulated and down-regulated genes between the electroacupuncture group and the model group.

Gene	log2FoldChange	p value	type
NONRATG015599.2	7.944322596	5.33688E-22	Up
NONRATG003981.2	7.630371026	4.71852E-07	Up
NONRATG015787.2	5.938643425	5.67911E-05	Up
NONRATG005609.2	5.757825755	3.64663E-05	Up

NONRATG020805.2	5.587487779	6.51983E-05	Up
NONRATG020690.2	4.352094144	7.4969E-05	Up
NONRATG009224.2	4.331102558	4.37903E-10	Up
NONRATG003870.2	4.191225993	0.000237824	Up
NONRATG005116.2	4.164772316	5.75201E-19	Up
NONRATG001704.2	4.15259453	4.88146E-19	Up
NONRATG019109.2	3.957250181	2.27535E-28	Up
NONRATG020131.2	3.733341446	2.24511E-19	Up
NONRATG022979.2	3.536110761	1.36266E-24	Up
NONRATG000504.2	3.463529477	0.012993055	Up
NONRATG013672.2	3.437557456	0.005953133	Up
NONRATG024117.2	3.403532764	0.000104277	Up
NONRATG017147.2	3.380196103	0.010432214	Up
NONRATG027393.1	3.276757194	3.2527E-11	Up
NONRATG011774.2	3.255894699	0.000962928	Up
NONRATG020791.2	3.237217012	0.005921711	Up
NONRATG021493.2	-8.453128468	8.51254E-11	Down
NONRATG024609.2	-7.396517202	3.08051E-08	Down
NONRATG027837.1	-7.106994293	1.53733E-14	Down
NONRATG011995.2	-7.057115793	2.54316E-07	Down
NONRATG009763.2	-6.63883376	1.02933E-05	Down
NONRATG007199.2	-5.541924391	7.82256E-05	Down
NONRATG002453.2	-4.955026554	3.63794E-17	Down
NONRATG003504.2	-4.940614934	0.000323741	Down
NONRATG019840.2	-4.824667973	4.64453E-06	Down
NONRATG006774.2	-4.724354987	3.82409E-11	Down
NONRATG013978.2	-4.445967105	5.73487E-08	Down
NONRATG011923.2	-4.397309881	0.003146419	Down
NONRATG003540.2	-4.354029097	0.000737645	Down
NONRATG013970.2	-3.998308517	9.71983E-07	Down
NONRATG026699.1	-3.985312667	8.37133E-07	Down
NONRATG017817.2	-3.865878734	0.003797321	Down
NONRATG005700.2	-3.806378945	4.14894E-06	Down
NONRATG015656.2	-3.632790852	5.55263E-07	Down
NONRATG008884.2	-3.606677945	1.00719E-19	Down
NONRATG003365.2	-3.527355593	0.002147843	Down

After comparing the model group with the control group, the P value <0.05, and according to the difference type given by log2FoldChange, when log2FoldChange>1 is marked as UP, it means up-regulated gene. Log2FoldChange<-1 is marked as Down, which means down-regulated gene.

Support Table 5.The top 10 miRNAs with significant differential fold change among the up-regulated and down-regulated genes between the model group and the control group.

Gene	log2FoldChange	p value	type
chr1_6474	8.767997629	1.35524E-08	Up
chr6_45562	5.605005862	6.31803E-05	Up
chr14_17213	4.878508096	0.005252131	Up
chr1_3827	3.768589245	0.004361745	Up
rno-miR-324-5p	2.831065904	1.54962E-18	Up
rno-miR-21-3p	2.615917844	1.21968E-19	Up
rno-miR-18a-5p	2.117889629	1.03098E-13	Up
rno-miR-3473	2.068501436	7.13049E-11	Up
rno-miR-98-3p	1.958868421	7.28687E-09	Up
rno-miR-873-5p	1.886743865	0.047760482	Up
chr1_3060	-3.283005322	0.041515549	Down
rno-miR-1298	-3.110395358	1.43818E-08	Down
rno-miR-592	-2.623118915	0.011945416	Down
rno-miR-211-5p	-1.749087673	0.001424682	Down
rno-miR-224-5p	-1.623389842	8.80533E-11	Down
rno-miR-672-5p	-1.461598695	0.007816187	Down
rno-miR-139-3p	-1.417106987	7.831E-07	Down
rno-miR-452-5p	-1.2718133	0.01448594	Down
rno-miR-203b-3p	-1.261201485	0.00496864	Down
rno-miR-200b-3p	-1.246915731	1.72882E-06	Down

After comparing the model group with the control group, the P value <0.05, and according to the difference type given by log2FoldChange, when log2FoldChange>1 is marked as UP, it means up-regulated gene. Log2FoldChange<-1 is marked as Down, which means down-regulated gene.

Support Table 6. The top 20 miRNAs with significant differential fold change among the up-regulated and down-regulated genes betweenthe electroacupuncture group and the model group.

Gene	log2FoldChange	p value	type
rno-miR-216a-5p	8.507753356	5.64806E-47	Up
rno-miR-217-5p	8.248392848	1.39888E-26	Up
chr11_11040	4.685992519	0.001311338	Up
rno-miR-592	2.545433362	0.011439526	Up
rno-miR-672-5p	2.34856989	2.04283E-05	Up
rno-miR-211-5p	1.772920703	0.001983935	Up
rno-miR-200b-3p	1.313750616	4.10616E-06	Up
rno-miR-203b-3p	1.231606044	0.003284706	Up
rno-miR-378b	1.22510055	4.65915E-08	Up
rno-miR-375-3p	1.181911184	1.04724E-06	Up
chr6_45562	-5.014264273	2.33365E-05	Down

chr14_17213	-4.779061254	0.002605753	Down
chr7_46545	-3.80116872	0.00189364	Down
chr1_3827	-3.792994035	0.000911788	Down
chr3_34614	-3.525879211	0.029917178	Down
rno-miR-673-5p	-2.510637842	0.014260825	Down
chr10_8577	-2.500572298	0.029461772	Down
rno-miR-92a-1-5p	-2.45602967	3.59567E-05	Down
rno-miR-1949	-2.260304341	0.004793907	Down
rno-miR-203a-5p	-2.216546922	0.030060605	Down

After comparing the model group with the control group, the P value <0.05, and according to the difference type given by log2FoldChange, when log2FoldChange>1 is marked as UP, it means up-regulated gene. Log2FoldChange<-1 is marked as Down, which means down-regulated gene.

Support Table 7.GO enrichment analysis of 429 DE mRNAs down-regulated by EA.

ID	Description	pvalue	geneID	Count	Type
GO:0098542	defense response to other organism	9.86539E-39	Parp9/Apobec3/Stat2/Slc11a1/RT1-S3/Ddx58/Stat1/Tlr2/Batf/Gbp4/Lbp/Dtx3l/Oasl/Nos2/Oasl2/Lgals9/Il10/Il17a/Ccr5/Isg15/Adar/Ifi27/Ncf1/Klrk1/Mx2/Pmaip1/Oas1e/Ifit3/Adamts4/Oas1k/Rsad2/Oas1a/Irf1/Mmp7/Zc3hav1/Tnf/Trim15/Ifit2/Oas3/Samhd1/Slfn13/Oas1b/Apobec1/Eif2ak2/Nlrp3/Gbp2/Il12rb1/Trem3/Gsdmd/Cxcl6/Irf7/Zc3h12a/Cd86/Irgm/Il1b/Oas2/Dhx58/Oas1i/Bcl3/Ifng/Irf8/Rtp4/Ifih1/Cxcl10/Lyz2/Anxa3/Nod2/Oas1h/Cxcl1/Cxcl9	70	BP
GO:0009615	response to virus	4.55674E-32	Parp9/Apobec3/Stat2/Ddx58/Stat1/Ifi27l2b/Dtx3l/Oasl/Oasl2/Lgals9/Isg15/Adar/Duox2/Ifi27/Mx2/Pmaip1/Oas1e/Ifit3/Oas1k/Rsad2/Oas1a/Irf1/Zc3hav1/Tnf/Psmb9/Trim15/Ifit2/Oas3/Samhd1/Slfn13/Oas1b/Apobec1/Eif2ak2/Nlrp3/Il12rb1/Irf7/Zc3h12a/Cd86/Irgm/Il1b/Oas2/Dhx58/Oas1i/Bcl3/Ifng/Rtp4/Ifih1/Cxcl10/Oas1h/Cxcl9	50	BP
GO:0051607	defense response to virus	1.8634E-30	Parp9/Apobec3/Stat2/Ddx58/Stat1/Dtx3l/Oasl/Oasl2/Isg15/Adar/Ifi27/Mx2/Pmaip1/Oas1e/Ifit3/Oas1k/Rsad2/Oas1a/Irf1/Zc3hav1/Trim15/Ifit2/Oas3/Samhd1/Slfn13/Oas1b/Apobec1/Eif2ak2/Nlrp3/Il12rb1/Irf7/Zc3h12a/Cd86/Il1b/Oas2/Dhx58/Oas1i/Ifng/Rtp4/Ifih1/Cxcl10/Oas1h/Cxcl9	43	BP
GO:0002237	response to molecule of bacterial origin	2.00455E-25	Scgb1a1/Slc11a1/Stat1/Tlr2/Socs1/Tap2/Lbp/Csf3/Fcgr3a/Nos2/Ptger1/Tlr1/Socs3/Lgals9/Litaf/Il10/Ccr5/Csf2rb/Il1a/Thpo/Prdm1/Ccl3/Serpina3n/Klrk1/Noct/Cd80/B2m/Cx3cl1/Tlr10/Zfp36/Ido1/Tnf/Sbno2/Acod1/Cxcl3/Cxcl16/Eif2ak2/Nlrp3/Gbp2/Il1rn/Cxcl6/Mmp9/Zc3h12a/Cd86/Irgm/Il1b/Il10ra/Ifng/Cmpk2/Irf8/Il18bp/Cxcl10/Nod2/Mefv/Cxcl1/Cxcl9	56	BP
GO:0001819	positive regulation of cytokine production	3.90174E-23	Cd74/Il17f/Slc11a1/RT1-S3/Ddx58/Stat1/Anxa1/Tlr2/Ereg/Ccl4/Lbp/C3/Tlr1/Nox1/Lgals9/Il10/Il17a/Ccr5/Il1a/Ccl3/Klrk1/Casp4/B2m/Cx3cl1/Rsad2/Irf1/Zc3h	51	BP

			av1/Ido1/Tnf/Tnfrsf8/Trim15/Mapk13/Eif2ak2/Il21/Nlrp3/Il12rb1/Il1rn/Osm/Gsdmd/Tnfsf9/Irf7/Il1b/Gbp5/Dhx58/Bcl3/Ifng/Il17c/Irf8/Sema7a/Ifih1/Nod2		
GO:0032496	response to lipopoly-saccharide	8.97682E-23	Scgb1a1/Slc11a1/Stat1/Tlr2/Socs1/Lbp/Csf3/Fcgr3a/Nos2/Ptger1/Socs3/Lgals9/Litaf/Il10/Ccr5/Csf2rb/Il1a/Thpo/Prdm1/Ccl3/Serpina3n/Klrk1/Noct/Cd80/Cx3cl1/Zfp36/Ido1/Tnf/Sbno2/Acod1/Cxcl3/Cxcl16/Eif2ak2/Nlrp3/Gbp2/Il1rn/Cxcl6/Mmp9/Zc3h12a/Cd86/Irgm/Il1b/Il10ra/Ifng/Cmpk2/Irf8/Il18bp/Cxcl10/Nod2/Mefv/Cxcl1/Cxcl9	52	BP
GO:0043900	regulation of multi-organism process	1.35788E-22	Parp9/Cd74/Tmprss2/Apobec3/Ddx58/Stat1/Tlr2/Ccl4/Lbp/Dtx3l/Oasl/Nos2/Oasl2/Lhfpl2/Lgals9/Il10/Ccr5/Ceacam1/Isg15/Prdm1/Ccl3/Adar/Ifi27/Mx2/Oas1e/Cx3cl1/Rsad2/Oas1a/Zfp36/Zc3hav1/Tnf/Trim15/Acod1/Oas3/Oas1b/Eif2ak2/Ada/Il12rb1/Cxcl6/Zc3h12a/Il1b/Dhx58/Oas1i/Ifng/Irf8/Nod2/Oas1h/Cxcl1	48	BP
GO:0002443	leukocyte mediated immunity	2.8054E-22	Cd74/Tap1/Slc11a1/RT1-S3/Ddx58/Lag3/Tlr2/Tap2/Batf/Gzmb/C3/Clec2d/Lgals9/Ceacam1/C2/Ccl3/RT1-N2/Ncf1/Klrk1/LOC102553861/B2m/Pou2f2/Rsad2/RT1-A1/RT1-CE10/Tnf/Ripk3/Gzmbl3/RT1-CE5/RT1-A2/Il21/Nlrp3/Rnf19b/Trem3/Cxcl6/Irf7/Il1b/Bcl3/Ifng/RT1-N3/RT1-T24-3/Anxa3/Nod2/RT1-CE16/Cxcl1/Jak3	46	BP
GO:0002697	regulation of immune effector process	3.61016E-22	Parp9/Cd74/Tap1/Apobec3/RT1-S3/Ddx58/Lag3/Stat1/Anxa1/Tlr2/Tap2/Lbp/Dtx3l/C3/Clec2d/Lgals9/Il10/Ceacam1/RT1-N2/Klrk1/B2m/Rsad2/RT1-DMb/RT1-A1/RT1-CE10/Zc3hav1/Tnf/Ripk3/Trim15/RT1-CE5/RT1-A2/Il21/Nlrp3/Il12rb1/Cxcl6/Zc3h12a/Il1b/Dhx58/Ifng/RT1-N3/Sema7a/Nfkbiz/RT1-T24-3/Nod2/RT1-CE16/Cxcl1/Jak3	47	BP
GO:0043903	regulation of symbi-otic process	8.20954E-22	Cd74/Tmprss2/Apobec3/Stat1/Tlr2/Ccl4/Lbp/Oasl/Oasl2/Lgals9/Il10/Ccr5/Ceacam1/Isg15/Ccl3/Adar/Ifi27/Mx2/Oas1e/Rsad2/Oas1a/Zfp36/Zc3hav1/Tnf/Trim15/Oas3/Oas1b/Eif2ak2/Cxcl6/Zc3h12a/Oas1i/Ifng/Irf8/Nod2/Oas1h/Cxcl1	36	BP
GO:0044419	interspecies interaction between organisms	1.05963E-21	Cd74/Tmprss2/Apobec3/Stat1/Tlr2/Ccl4/Gbp4/Lbp/Oasl/Nos2/Oasl2/Lgals9/Il10/Ccr5/Ceacam1/Isg15/Nectin4/Ccl3/Adar/Ifi27/Ncf1/Mx2/Oas1e/Rsad2/Oas1a/Zfp36/Zc3hav1/Tnf/Trim15/Oas3/Oas1b/Eif2ak2/Gbp2/Trem3/Gsdmd/Cxcl6/Mmp9/Zc3h12a/Irgm/Oas1i/Ifng/Irf8/Napepld/Lyz2/Nod2/Oas1h/Cxcl1	47	BP
GO:0043901	negative regulation of multi-organism pro-cess	1.23598E-21	Apo-bec3/Stat1/Tlr2/Ccl4/Lbp/Oasl/Oasl2/Il10/Ceacam1/Isg15/Prdm1/Ccl3/Adar/Mx2/Oas1e/Rsad2/Oas1a/Zfp36/Zc3hav1/Tnf/Trim15/Acod1/Oas3/Oas1b/Eif2ak2/Ada/Zc3h12a/Dhx58/Oas1i/Ifng/Irf8/Nod2/Oas1h	33	BP

GO:0031349	positive regulation of defense response	2.18629E-21	Parp9/Il17f/RT1-S3/Ddx58/Egfr/Lag3/Tlr2/Ereg/Lbp/C3/Tlr1/Lgals9/Il17a/Ccr5/Clec7a/Ccl3/Zbp1/Klrk1/Cx3cl1/Tlr10/Rsad2/Irf1/Zc3hav1/Ido1/Tnf/Trim15/Acod1/Tifa/Il21/Osm/Trem3/Irf7/Cd86/Irgm/Il1b/Gbp5/Dhx58/Il17c/Cck/Ifih1/Napepld/Nfkbiz/Nod2/Cxcl1	44	BP
GO:0044403	symbiotic process	3.84636E-21	Cd74/Tmprss2/Apobec3/Stat1/Tlr2/Ccl4/Gbp4/Lbp/Oasl/Oasl2/Lgals9/Il10/Ccr5/Ceacam1/Isg15/Nectin4/Ccl3/Adar/Ifi27/Ncf1/Mx2/Oas1e/Rsad2/Oas1a/Zfp36/Zc3hav1/Tnf/Trim15/Oas3/Oas1b/Eif2ak2/Gbp2/Trem3/Cxcl6/Mmp9/Zc3h12a/Irgm/Oas1i/Ifng/Irf8/Napepld/Nod2/Oas1h/Cxcl1	44	BP
GO:0045088	regulation of innate immune response	4.41898E-21	Parp9/Tap1/RT1-S3/Ddx58/Lag3/Tlr2/Socs1/Ereg/Tap2/Lbp/Parp14/Tlr1/Clec2d/Lgals9/Ceacam1/Clec7a/Adar/Zbp1/Klrk1/Trafd1/Tlr10/Rsad2/RT1-A1/Irf1/Zc3hav1/Tnf/Trim15/Acod1/Samhd1/Tifa/Il21/Trem3/Irf7/Cd86/Irgm/Gbp5/Dhx58/Ifih1/Nod2	39	BP
GO:0071219	cellular response to molecule of bacterial origin	7.50335E-21	Stat1/Tlr2/Lbp/Csf3/Fcgr3a/Nos2/Tlr1/Litaf/Il10/Ccr5/Thpo/Prdm1/Ccl3/Klrk1/Cd80/Cx3cl1/Tlr10/Zfp36/Tnf/Sbno2/Acod1/Cxcl3/Cxcl16/Nlrp3/Gbp2/Il1rn/Cxcl6/Mmp9/Zc3h12a/Cd86/Irgm/Il1b/Ifng/Cmpk2/Irf8/Cxcl10/Nod2/Cxcl1/Cxcl9	39	BP
GO:0071216	cellular response to biotic stimulus	2.48859E-20	Stat1/Tlr2/Lbp/Csf3/Fcgr3a/Nos2/Tlr1/Litaf/Il10/Ccr5/Thpo/Prdm1/Ccl3/Klrk1/Cd80/Cx3cl1/Tlr10/Bid/Zfp36/Tnf/Sbno2/Acod1/Cxcl3/Cxcl16/Nlrp3/Gbp2/Il1rn/Cxcl6/Mmp9/Zc3h12a/Cd86/Irgm/Il1b/Ifng/Cmpk2/Irf8/Cxcl10/Nod2/Cxcl1/Cxcl9	40	BP
GO:0019221	cytokine-mediated signaling pathway	1.11214E-19	Parp9/Cxcr3/Cd74/Stat2/Stat1/Socs1/Ereg/Ccl4/Parp14/Csf3r/Ccr5/Ceacam1/Csf2rb/Il1a/Thpo/Ccl3/Adar/Duox2/Zbp1/Ifi27/Casp4/Cx3cl1/Duox1/Irf1/Tnf/Cxcl3/Samhd1/Il12rb1/Il1rn/Osm/Cxcl6/Irf7/Irgm/Il1b/Oas2/Il10ra/Ifng/Ccrl2/Cxcl10/Cxcl1/Jak3/Cxcl9	42	BP
GO:0001906	cell killing	1.25084E-19	Tap1/RT1-S3/Lag3/Tap2/Gzmb/Nos2/Clec2d/Lgals9/Ccr5/Ceacam1/RT1-N2/Ncf1/Klrk1/LOC102553861/B2m/RT1-A1/RT1-CE10/Ripk3/Gzmbl3/RT1-CE5/RT1-A2/Il21/Rnf19b/Trem3/Cxcl6/Ifng/RT1-N3/RT1-T24-3/Lyz2/RT1-CE16/Cxcl1	31	BP
GO:0001909	leukocyte mediated cytotoxicity	2.5727E-19	Tap1/RT1-S3/Lag3/Tap2/Gzmb/Clec2d/Lgals9/Ceacam1/RT1-N2/Ncf1/Klrk1/LOC102553861/B2m/RT1-A1/RT1-CE10/Ripk3/Gzmbl3/RT1-CE5/RT1-A2/Il21/Rnf19b/Trem3/Cxcl6/RT1-N3/RT1-T24-3/RT1-CE16/Cxcl1	27	BP
GO:0002699	positive regulation of immune effector pro-	3.05977E-19	Cd74/RT1-S3/Ddx58/Lag3/Anxa1/Tlr2/Tap2/Lbp/C3/Lg	35	BP

	cess		als9/Ceacam1/RT1-N2/Klrk1/B2m/Rsad2/RT1-DMb/RT1-A1/RT1-CE10/Zc3hav1/Tnf/Trim15/RT1-CE5/RT1-A2/Il21/Nlrp3/Il1b/Dhx58/Ifng/RT1-N3/Sema7a/Nfkbiz/RT1-T24-3/Nod2/RT1-CE16/Cxcl1		
GO:0002449	lymphocyte mediated immunity	6.97428E-19	Cd74/Tap1/Slc11a1/RT1-S3/Lag3/Tap2/Batf/Gzmb/C3/Clec2d/Lgals9/Ceacam1/C2/RT1-N2/Klrk1/LOC102553861/B2m/Pou2f2/Rsad2/RT1-A1/RT1-CE10/Tnf/Ripk3/Gzmbl3/RT1-CE5/RT1-A2/Il21/Nlrp3/Rnf19b/Irf7/Il1b/Bcl3/Ifng/RT1-N3/RT1-T24-3/Nod2/RT1-CE16	37	BP
GO:0071222	cellular response to lipopolysaccharide	1.24193E-18	Stat1/Lbp/Csf3/Fcgr3a/Nos2/Litaf/Il10/Ccr5/Thpo/Prdm1/Ccl3/Klrk1/Cd80/Cx3cl1/Zfp36/Tnf/Sbno2/Acod1/Cxcl3/Cxcl16/Nlrp3/Gbp2/Il1rn/Cxcl6/Mmp9/Zc3h12a/Cd86/Irgm/Il1b/Ifng/Cmpk2/Irf8/Cxcl10/Nod2/Cxcl1/Cxcl9	36	BP
GO:0048525	negative regulation of viral process	2.44573E-18	Apo-bec3/Stat1/Ccl4/Oasl/Oasl2/Ceacam1/Isg15/Ccl3/Adar/Mx2/Oas1e/Rsad2/Oas1a/Zfp36/Zc3hav1/Tnf/Trim15/Oas3/Oas1b/Eif2ak2/Zc3h12a/Oas1i/Oas1h	23	BP
GO:0045071	negative regulation of viral genome replica-tion	8.45827E-18	Apo-bec3/Oasl/Oasl2/Ceacam1/Isg15/Adar/Mx2/Oas1e/Rsad2/Oas1a/Zc3hav1/Tnf/Oas3/Oas1b/Eif2ak2/Zc3h12a/Oas1i/Oas1h	18	BP
GO:0002460	adaptive immune response based on somatic recombination of immune receptors built from immuno-globulin superfamily domains	1.05252E-17	Cd74/Sema4a/Slc11a1/RT1-S3/Anxa1/Tap2/Batf/Gzmb/C3/Ceacam1/C2/RT1-N2/B2m/Pou2f2/Rsad2/RT1-A1/RT1-CE10/Tnf/Ripk3/RT1-CE5/RT1-A2/Nlrp3/Ada/Il12rb1/Irf7/Zc3h12a/Il1b/Bcl3/Ifng/RT1-N3/Il18bp/Nfkbiz/RT1-T24-3/Nod2/RT1-CE16/Jak3	36	BP
GO:0031341	regulation of cell killing	1.89698E-17	Tap1/RT1-S3/Lag3/Tap2/Nos2/Clec2d/Lgals9/Ccr5/Ceacam1/RT1-N2/Klrk1/B2m/RT1-A1/RT1-CE10/Ripk3/RT1-CE5/RT1-A2/Il21/Cxcl6/Ifng/RT1-N3/RT1-T24-3/RT1-CE16/Cxcl1	24	BP
GO:0034341	response to interferon-gamma	1.59094E-16	Parp9/Slc11a1/Stat1/Tlr2/Socs1/Ccl4/Gbp4/Parp14/Nos2/Lgals9/Ciita/Ccl3/Serpina3n/Cx3cl1/Irf1/Acod1/Cxcl16/Gbp2/Il12rb1/Cd86/Irgm/Gbp5/Ubd/Ifng/Irf8/Mefv	26	BP
GO:0002250	adaptive immune response	2.92642E-16	Cd74/Tap1/Sema4a/Slc11a1/RT1-S3/Anxa1/Tap2/Batf/Gzmb/C3/Ceacam1/C2/RT1-N2/Klrk1/B2m/Pou2f2/Rsad2/RT1-A1/RT1-CE10/Tnf/Ripk3/RT1-CE5/RT1-A2/Nlrp3/Ada/Il12rb1/Irf7/Zc3h12a/Il1b/Bcl3/Ifng/RT1-N3/Il18bp/Nfkbiz/RT1-T24-3/Nod2/RT1-CE16/Jak3	38	BP
GO:0002819	regulation of adaptive immune response	3.45836E-16	Slc11a1/RT1-S3/Anxa1/Tap2/C3/Ceacam1/RT1-N2/B2m/Rsad2/RT1-A1/RT1-CE10/Tnf/Ripk3/RT1-CE5/RT1-	28	BP

			A2/Nlrp3/Ada/Il12rb1/Irf7/Zc3h12a/Il1b/Ifng/RT1-N3/Nfkbiz/RT1-T24-3/Nod2/RT1-CE16/Jak3		
GO:0050727	regulation of inflammatory response	3.53751E-16	Scgb1a1/Il22/Il17f/Pla2g5/Egfr/Anxa1/Tlr2/Lbp/C3/Nos2/Socs3/Lgals9/Il10/Il17a/Ccr5/Ccl3/Duoxa2/Usp18/Cx3cl1/Zfp36/Ido1/Tnf/Sbno2/Acod1/Nlrp3/Ada/Osm/Zc3h12a/Il1b/Gbp5/Il17c/Sema7a/Napepld/Nfkbiz/Nod2/Mefv	36	BP
GO:0002703	regulation of leukocyte mediated immunity	4.62438E-16	Tap1/RT1-S3/Ddx58/Lag3/Tlr2/Tap2/C3/Clec2d/Lgals9/Ceacam1/RT1-N2/Klrk1/B2m/Rsad2/RT1-A1/RT1-CE10/Tnf/Ripk3/RT1-CE5/RT1-A2/Il21/Nlrp3/Cxcl6/Il1b/Ifng/RT1-N3/RT1-T24-3/Nod2/RT1-CE16/Cxcl1/Jak3	31	BP
GO:0002822	regulation of adaptive immune response based on somatic recombination of immune receptors built from immunoglobulin superfamily domains	6.23746E-16	Slc11a1/RT1-S3/Anxa1/Tap2/C3/Ceacam1/RT1-N2/B2m/Rsad2/RT1-A1/RT1-CE10/Tnf/Ripk3/RT1-CE5/RT1-A2/Nlrp3/Ada/Il12rb1/Zc3h12a/Il1b/Ifng/RT1-N3/Nfkbiz/RT1-T24-3/Nod2/RT1-CE16/Jak3	27	BP
GO:0001910	regulation of leukocyte mediated cytotoxicity	1.09835E-15	Tap1/RT1-S3/Lag3/Tap2/Clec2d/Lgals9/Ceacam1/RT1-N2/Klrk1/B2m/RT1-A1/RT1-CE10/Ripk3/RT1-CE5/RT1-A2/Il21/Cxcl6/RT1-N3/RT1-T24-3/RT1-CE16/Cxcl1	21	BP
GO:0002683	negative regulation of immune system process	1.53969E-15	Scgb1a1/Cd74/Tap1/RT1-S3/Lag3/Anxa1/Pdcd1/Socs1/Tap2/Parp14/Dll1/Clec2d/Lgals9/Il10/Ceacam1/Prdm1/Ccl3/Adar/Klrk1/Cd80/Trafd1/Cx3cl1/Fcrlb/Runx3/RT1-A1/Mafb/Irf1/Zfp36/Ido1/Tnf/Socs6/Acod1/Samhd1/Ada/Lilrb4/Zc3h12a/Cd86/Dhx58/Ifng/Zc3h12d/Nod2/Jak3	42	BP
GO:1903901	negative regulation of viral life cycle	1.92607E-15	Apobec3/Oasl/Oasl2/Ceacam1/Isg15/Adar/Mx2/Oas1e/Rsad2/Oas1a/Zc3hav1/Tnf/Trim15/Oas3/Oas1b/Eif2ak2/Zc3h12a/Oas1i/Oas1h	19	BP
GO:0050792	regulation of viral process	1.98347E-15	Cd74/Tmprss2/Apobec3/Stat1/Ccl4/Oasl/Oasl2/Lgals9/Ceacam1/Isg15/Ccl3/Adar/Ifi27/Mx2/Oas1e/Rsad2/Oas1a/Zfp36/Zc3hav1/Tnf/Trim15/Oas3/Oas1b/Eif2ak2/Zc3h12a/Oas1i/Oas1h	27	BP
GO:0002824	positive regulation of adaptive immune response based on somatic recombination of immune receptors built from immunoglobulin superfamily domains	2.04815E-15	Slc11a1/RT1-S3/Anxa1/Tap2/C3/RT1-N2/B2m/Rsad2/RT1-A1/RT1-CE10/Tnf/RT1-CE5/RT1-A2/Nlrp3/Ada/Il12rb1/Il1b/Ifng/RT1-N3/Nfkbiz/RT1-T24-3/Nod2/RT1-CE16	23	BP
GO:0002821	positive regulation of adaptive immune	4.19886E-15	Slc11a1/RT1-S3/Anxa1/Tap2/C3/RT1-N2/B2m/Rsad2/RT1-A1/RT1-CE10/Tnf/RT1-CE5/RT1-	23	BP

	response		A2/Nlrp3/Ada/Il12rb1/Il1b/Ifng/RT1-N3/Nfkbiz/RT1-T24-3/Nod2/RT1-CE16		
GO:0002706	regulation of lympho-cyte mediated im-munity	9.76571E-15	Tap1/RT1-S3/Lag3/Tap2/C3/Clec2d/Lgals9/Ceacam1/RT1-N2/Klrk1/B2m/Rsad2/RT1-A1/RT1-CE10/Tnf/Ripk3/RT1-CE5/RT1-A2/Il21/Nlrp3/Il1b/Ifng/RT1-N3/RT1-T24-3/Nod2/RT1-CE16	26	BP
GO:0045089	positive regulation of innate immune re-sponse	1.40833E-14	Parp9/RT1-S3/Ddx58/Lag3/Tlr2/Ereg/Lbp/Tlr1/Lgals9/Clec7a/Zbp1/Klrk1/Tlr10/Rsad2/Irf1/Zc3hav1/Tnf/Trim15/Acod1/Tifa/Il21/Trem3/Irf7/Cd86/Irgm/Gbp5/Dhx58/Ifih1/Nod2	29	BP
GO:0019882	antigen processing and presentation	2.69035E-14	Cd74/Tap1/Slc11a1/RT1-S3/Tap2/RT1-CE4/RT1-N2/B2m/RT1-DMb/RT1-A1/RT1-CE10/Psmb9/RT1-CE5/RT1-A2/Psmb8/Ifng/RT1-N3/RT1-T24-3/Nod2/RT1-CE16	20	BP
GO:1903900	regulation of viral life cycle	3.10318E-14	Cd74/Tmprss2/Apobec3/Oasl/Oasl2/Lgals9/Cea-cam1/Isg15/Adar/Ifi27/Mx2/Oas1e/Rsad2/Oas1a/Zc3hav1/Tnf/Trim15/Oas3/Oas1b/Eif2ak2/Zc3h12a/Oas1i/Oas1h	23	BP
GO:0045069	regulation of viral genome replication	3.40127E-14	Apo-bec3/Oasl/Oasl2/Ceacam1/Isg15/Adar/Ifi27/Mx2/Oas1e/Rsad2/Oas1a/Zc3hav1/Tnf/Oas3/Oas1b/Eif2ak2/Zc3h12a/Oas1i/Oas1h	19	BP
GO:0035458	cellular response to interferon-beta	9.75643E-14	MGC108823/MGC105567/Stat1/Gbp4/Trex1/Ifit3/Irf1/RGD1309362/Acod1/Igtp/Ifi47/Gbp2/Irgm	13	BP
GO:0048002	antigen processing and presentation of peptide antigen	1.53943E-13	Cd74/Tap1/Slc11a1/RT1-S3/Tap2/RT1-CE4/RT1-N2/B2m/RT1-DMb/RT1-A1/RT1-CE10/RT1-CE5/RT1-A2/RT1-N3/RT1-T24-3/RT1-CE16	16	BP
GO:0071346	cellular response to interferon-gamma	1.63848E-13	Parp9/Stat1/Tlr2/Socs1/Ccl4/Gbp4/Parp14/Nos2/Lgals9/Ciita/Ccl3/Serpina3n/Cx3cl1/Irf1/Acod1/Gbp2/Il12rb1/Irgm/Gbp5/Ifng/Irf8	21	BP
GO:0002483	antigen processing and presentation of endog-enous peptide antigen	2.85051E-13	Tap1/RT1-S3/Tap2/RT1-N2/B2m/RT1-A1/RT1-CE10/RT1-CE5/RT1-A2/RT1-N3/RT1-T24-3/RT1-CE16	12	BP
GO:0031343	positive regulation of cell killing	2.8921E-13	RT1-S3/Lag3/Tap2/Nos2/Ccr5/RT1-N2/Klrk1/B2m/RT1-A1/RT1-CE10/RT1-CE5/RT1-A2/Il21/Ifng/RT1-N3/RT1-T24-3/RT1-CE16/Cxcl1	18	BP
GO:0032103	positive regulation of response to external stimulus	3.0938E-13	Cxcr3/Cd74/Il17f/Egfr/Tlr2/Ccl4/Tiam1/Lbp/C3/Lgals9/Il17a/Ccr5/Ccl3/Cx3cl1/Zc3hav1/Ido1/Tnf/Trim15/Acod1/Osm/Edn3/Il1b/Gbp5/Dhx58/Il17c/Napepld/Nfkbiz/Cxcl10/Nod2/Cxcl1/Cxcl9	31	BP
GO:0002831	regulation of response to biotic stimulus	3.38249E-13	Parp9/Apobec3/Ddx58/Stat1/Tlr2/Dtx3l/Lgals9/Ceacam1/Prdm1/Klrk1/Cx3cl1/Zc3hav1/Trim15/Acod1/Il12rb1/Cxcl6/Zc3h12a/Il1b/Dhx58/Ifng/Nod2/Cxcl1	22	BP
GO:0002428	antigen processing and	3.94359E-13	RT1-S3/Tap2/RT1-N2/B2m/RT1-A1/RT1-	11	BP

	presentation of peptide antigen via MHC class Ib		CE10/RT1-CE5/RT1-A2/RT1-N3/RT1-T24-3/RT1-CE16		
GO:0016032	viral process	4.20011E-13	Cd74/Tmprss2/Apobec3/Stat1/Ccl4/Oasl/Oasl2/Lgals9/Ccr5/Ceacam1/Isg15/Nectin4/Ccl3/Adar/Ifi27/Mx2/Oas1e/Rsad2/Oas1a/Zfp36/Zc3hav1/Tnf/Trim15/Oas3/Oas1b/Eif2ak2/Mmp9/Zc3h12a/Oas1i/Oas1h	30	BP
GO:0002708	positive regulation of lymphocyte mediated immunity	5.91886E-13	RT1-S3/Lag3/Tap2/C3/RT1-N2/Klrk1/B2m/Rsad2/RT1-A1/RT1-CE10/Tnf/RT1-CE5/RT1-A2/Il21/Nlrp3/Il1b/Ifng/RT1-N3/RT1-T24-3/Nod2/RT1-CE16	21	BP
GO:0002705	positive regulation of leukocyte mediated immunity	7.42751E-13	RT1-S3/Ddx58/Lag3/Tap2/C3/RT1-N2/Klrk1/B2m/Rsad2/RT1-A1/RT1-CE10/Tnf/RT1-CE5/RT1-A2/Il21/Nlrp3/Il1b/Ifng/RT1-N3/RT1-T24-3/Nod2/RT1-CE16/Cxcl1	23	BP
GO:0019883	antigen processing and presentation of endog-enous antigen	7.45829E-13	Tap1/RT1-S3/Tap2/RT1-N2/B2m/RT1-A1/RT1-CE10/RT1-CE5/RT1-A2/RT1-N3/RT1-T24-3/RT1-CE16	12	BP
GO:0019079	viral genome replica-tion	1.14913E-12	Apo-bec3/Oasl/Oasl2/Ceacam1/Isg15/Adar/Ifi27/Mx2/Oas1e/Rsad2/Oas1a/Zc3hav1/Tnf/Oas3/Oas1b/Eif2ak2/Zc3h12a/Oas1i/Oas1h	19	BP
GO:0031348	negative regulation of defense response	1.19374E-12	Tap1/Il22/RT1-S3/Pla2g5/Tap2/Parp14/Clec2d/Socs3/Lgals9/Il10/Ceacam1/Adar/Trafd1/Cx3cl1/RT1-A1/Zfp36/Acod1/Samhd1/Nlrp3/Ada/Zc3h12a/Dhx58/Cck/Nod2/Mefv	25	BP
GO:0002758	innate immune re-sponse-activating signal transduction	1.25917E-12	Ddx58/Tlr2/Lbp/Tlr1/Lgals9/Clec7a/Klrk1/Tlr10/Rsad2/Irf1/Zc3hav1/Tnf/Trim15/Acod1/Tifa/Irf7/Cd86/Irgm/Dhx58/Ifih1/Nod2	21	BP
GO:0002709	regulation of T cell mediated immunity	1.29016E-12	RT1-S3/Tap2/Ceacam1/RT1-N2/B2m/Rsad2/RT1-A1/RT1-CE10/Ripk3/RT1-CE5/RT1-A2/Nlrp3/Il1b/RT1-N3/RT1-T24-3/Nod2/RT1-CE16	17	BP
GO:0042110	T cell activation	1.46729E-12	Scgb1a1/Cd74/Sema4a/Itgad/Slc11a1/RT1-S3/Lag3/Anxa1/Socs1/Batf/Lgals9/Ceacam1/Prdm1/Cd80/B2m/Rsad2/RT1-DMb/Runx3/Mafb/Irf1/Ido1/Ripk3/Socs6/Il21/Nlrp3/Ada/Il12rb1/Hsh2d/Lilrb4/Tnfsf9/Zc3h12a/Cd86/Il1b/Bcl3/Ifng/Zc3h12d/Nfkbiz/Jak3	38	BP
GO:0050663	cytokine secretion	1.53472E-12	Il17f/Ddx58/Anxa1/Tlr2/Socs1/Nos2/Tlr1/Lgals9/Il10/Il17a/Ccr5/Il1a/Ccl3/Casp4/Cx3cl1/Tnf/Nlrp3/Rnf19b/Osm/Trem3/Gsdmd/Zc3h12a/Il1b/Gbp5/Ifng/Ifih1/Nod2	27	BP
GO:0002475	antigen processing and presentation via MHC class Ib	2.12741E-12	RT1-S3/Tap2/RT1-N2/B2m/RT1-A1/RT1-CE10/RT1-CE5/RT1-A2/RT1-N3/RT1-T24-3/RT1-CE16	11	BP
GO:0032755	positive regulation of interleukin-6 produc-tion	2.99206E-12	Il17f/Ddx58/Tlr2/Ereg/Lbp/Tlr1/Lgals9/Il17a/Ccr5/Il1a/Tnf/Mapk13/Il1rn/Tnfsf9/Il1b/Ifng/Ifih1/Nod2	18	BP

GO:0022407	regulation of cell-cell adhesion	3.715E-12	Scgb1a1/Abat/Cd74/RT1-S3/Lag3/Anxa1/Socs1/Lgals9/Il10/Bmp2/Ccr5/Ceacam1/Cd80/Cx3cl1/RT1-DMb/Runx3/Irf1/Ido1/Tnf/Socs6/Il21/Nlrp3/Ada/Il12rb1/Il1rn/Lilrb4/Tnfsf9/Zc3h12a/Cd86/Il1b/Ifng/Zc3h12d/Nfkbiz/Jak3	34	BP
GO:0050900	leukocyte migration	3.98207E-12	Cxcr3/Cd74/Anxa1/Tlr2/Ccl4/Lbp/Lgals9/Csf3r/Il17a/Ccr5/Il1a/Ccl3/Klrk1/Cx3cl1/Trpm2/Tnf/Ripk3/Cxcl3/Cxcl16/Ada/Il1rn/Trem3/Cxcl6/Mmp9/Edn3/Il1b/Ifng/Cxcl10/Nod2/Cxcl1/Cxcl9	31	BP
GO:0002764	immune response-regulating signaling pathway	4.31319E-12	Ddx58/Tlr2/Lbp/Fcgr3a/Tlr1/Lgals9/Ceacam1/Clec7a/Klrk1/Btnl7/Tlr10/Rsad2/Irf1/Zc3hav1/Tnf/Trim15/Acod1/Tifa/Ada/Themis2/Lilrb4/Btnl5/Irf7/Zc3h12a/Cd86/Irgm/Dhx58/Ifng/Ifih1/Nfkbiz/Nod2	31	BP
GO:0001914	regulation of T cell mediated cytotoxicity	4.49862E-12	RT1-S3/Tap2/Ceacam1/RT1-N2/B2m/RT1-A1/RT1-CE10/Ripk3/RT1-CE5/RT1-A2/RT1-N3/RT1-T24-3/RT1-CE16	13	BP
GO:0002757	immune response-activating signal transduction	5.3194E-12	Ddx58/Tlr2/Lbp/Tlr1/Lgals9/Ceacam1/Clec7a/Klrk1/Btnl7/Tlr10/Rsad2/Irf1/Zc3hav1/Tnf/Trim15/Acod1/Tifa/Ada/Themis2/Lilrb4/Btnl5/Irf7/Zc3h12a/Cd86/Irgm/Dhx58/Ifng/Ifih1/Nfkbiz/Nod2	30	BP
GO:0035456	response to interferon-beta	6.20509E-12	MGC108823/MGC105567/Stat1/Gbp4/Trex1/Ifit3/Irf1/RGD1309362/Acod1/Igtp/Ifi47/Gbp2/Irgm	13	BP
GO:0002456	T cell mediated im-munity	8.21564E-12	Slc11a1/RT1-S3/Tap2/Gzmb/Ceacam1/RT1-N2/B2m/Rsad2/RT1-A1/RT1-CE10/Ripk3/RT1-CE5/RT1-A2/Nlrp3/Il1b/RT1-N3/RT1-T24-3/Nod2/RT1-CE16	19	BP
GO:0002221	pattern recognition receptor signaling pathway	8.33186E-12	Ddx58/Tlr2/Lbp/Tlr1/Lgals9/Clec7a/Tlr10/Rsad2/Irf1/Zc3hav1/Tnf/Trim15/Acod1/Tifa/Irf7/Cd86/Irgm/Dhx58/Ifih1/Nod2	20	BP
GO:0060700	regulation of ribonu-clease activity	8.47016E-12	Oasl/Oasl2/Oas1e/Oas1a/Oas3/Oas1b/Oas2/Oas1i/Oas1h	9	BP
GO:0002476	antigen processing and presentation of endog-enous peptide antigen via MHC class Ib	9.07346E-12	RT1-S3/Tap2/RT1-N2/RT1-A1/RT1-CE10/RT1-CE5/RT1-A2/RT1-N3/RT1-T24-3/RT1-CE16	10	BP
GO:0042742	defense response to bacterium	1.03916E-11	Slc11a1/RT1-S3/Tlr2/Gbp4/Lbp/Nos2/Lgals9/Il10/Ccr5/Isg15/Ncf1/Klrk1/Adamts4/Mmp7/Tnf/Nlrp3/Gbp2/Trem3/Gsdmd/Cxcl6/Irgm/Bcl3/Ifng/Irf8/Lyz2/Anxa3/Nod2	27	BP
GO:0050863	regulation of T cell activation	1.15368E-11	Scgb1a1/Cd74/RT1-S3/Lag3/Anxa1/Socs1/Lgals9/Ceacam1/Prdm1/Cd80/RT1-DMb/Runx3/Irf1/Ido1/Ripk3/Socs6/Il21/Nlrp3/Ada/Il12rb1/Lilrb4/Tnfsf9/Zc3h12a/Cd86/Il1b/Ifng/Zc3h12d/Nfkbiz/Jak3	29	BP
GO:1903706	regulation of hemo-poiesis	1.23949E-11	Cd74/Stat1/Anxa1/Socs1/Csf3/Dll1/Clec2d/Lgals9/Csf3r/Il17a/Ceacam1/Isg15/Thpo/Prdm1/Ccl3/B2m/Runx3/Mafb/Irf1/Zfp36/Tnf/Eif	33	BP

			2ak2/Il21/Nlrp3/Ada/Lilrb4/Tnfsf9/Zc3h12a/Il1b/Ifng/Nfkbiz/Cxcl1/Jak3		
GO:1903037	regulation of leuko-cyte cell-cell adhesion	1.51149E-11	Scgb1a1/Cd74/RT1-S3/Lag3/Anxa1/Socs1/Lgals9/Ceacam1/Cd80/RT1-DMb/Runx3/Irf1/Ido1/Tnf/Socs6/Il21/Nlrp3/Ada/Il12rb1/Lilrb4/Tnfsf9/Zc3h12a/Cd86/Il1b/Ifng/Zc3h12d/Nfkbiz/Jak3	28	BP
GO:0030593	neutrophil chemotaxis	1.58543E-11	Cd74/Ccl4/Lbp/Csf3r/Ccl3/Cx3cl1/Cxcl3/Il1rn/Trem3/Cxcl6/Edn3/Il1b/Ifng/Cxcl10/Nod2/Cxcl1/Cxcl9	17	BP
GO:0097530	granulocyte migration	1.90375E-11	Cd74/Anxa1/Ccl4/Lbp/Csf3r/Il17a/Il1a/Ccl3/Cx3cl1/Cxcl3/Il1rn/Trem3/Cxcl6/Edn3/Il1b/Ifng/Cxcl10/Nod2/Cxcl1/Cxcl9	20	BP
GO:0019058	viral life cycle	1.92183E-11	Cd74/Tmprss2/Apobec3/Oasl/Oasl2/Lgals9/Cea-cam1/Isg15/Nectin4/Adar/Ifi27/Mx2/Oas1e/Rsad2/Oas1a/Zc3hav1/Tnf/Trim15/Oas3/Oas1b/Eif2ak2/Zc3h12a/Oas1i/Oas1h	24	BP
GO:0042107	cytokine metabolic process	2.24214E-11	Il17f/Lag3/Tlr2/Ereg/Lbp/Tlr1/Il10/Il1a/Irf1/Zfp36/Tnf/Tnfrsf8/Il21/Trem3/Irf7/Il1b/Bcl3/Ifng	18	BP
GO:0002711	positive regulation of T cell mediated im-munity	2.31703E-11	RT1-S3/Tap2/RT1-N2/B2m/Rsad2/RT1-A1/RT1-CE10/RT1-CE5/RT1-A2/Nlrp3/Il1b/RT1-N3/RT1-T24-3/RT1-CE16	14	BP
GO:0032635	interleukin-6 produc-tion	2.42347E-11	Il17f/Ddx58/Tlr2/Ereg/Lbp/Nos2/Tlr1/Lgals9/Il10/Il17a/Ccr5/Il1a/Tnf/Mapk13/Il1rn/Tnfsf9/Zc3h12a/Il1b/Ifng/Ifih1/Nod2	21	BP
GO:1990266	neutrophil migration	3.05627E-11	Cd74/Ccl4/Lbp/Csf3r/Il1a/Ccl3/Cx3cl1/Cxcl3/Il1rn/Trem3/Cxcl6/Edn3/Il1b/Ifng/Cxcl10/Nod2/Cxcl1/Cxcl9	18	BP
GO:0045824	negative regulation of innate immune re-sponse	3.60477E-11	Tap1/RT1-S3/Tap2/Parp14/Clec2d/Lgals9/Ceacam1/Adar/Trafd1/RT1-A1/Acod1/Samhd1/Dhx58	13	BP
GO:0001913	T cell mediated cyto-toxicity	3.73671E-11	RT1-S3/Tap2/Gzmb/Ceacam1/RT1-N2/B2m/RT1-A1/RT1-CE10/Ripk3/RT1-CE5/RT1-A2/RT1-N3/RT1-T24-3/RT1-CE16	14	BP
GO:0001912	positive regulation of leukocyte mediated cytotoxicity	3.89127E-11	RT1-S3/Lag3/Tap2/RT1-N2/Klrk1/B2m/RT1-A1/RT1-CE10/RT1-CE5/RT1-A2/Il21/RT1-N3/RT1-T24-3/RT1-CE16/Cxcl1	15	BP
GO:0002253	activation of immune response	5.36018E-11	Ddx58/Tlr2/Lbp/C3/Tlr1/Lgals9/Ceacam1/C2/Clec7a/Klrk1/Btnl7/Tlr10/Rsad2/Irf1/Zc3hav1/Tnf/Trim15/Acod1/Tifa/Ada/Themis2/Lilrb4/Btnl5/Irf7/Zc3h12a/Cd86/Irgm/Dhx58/Ifng/Ifih1/Nfkbiz/Nod2	32	BP
GO:0002218	activation of innate immune response	5.58872E-11	Ddx58/Tlr2/Lbp/Tlr1/Lgals9/Clec7a/Klrk1/Tlr10/Rsad2/Irf1/Zc3hav1/Tnf/Trim15/Acod1/Tifa/Irf7/Cd86/Irgm/Dhx58/Ifih1/Nod2	21	BP
GO:0071621	granulocyte chemotax-is	6.44357E-11	Cd74/Anxa1/Ccl4/Lbp/Csf3r/Ccl3/Cx3cl1/Cxcl3/Il1rn/Trem3/Cxcl6/Edn3/Il1b/Ifng/Cxcl10	18	BP

			/Nod2/Cxcl1/Cxcl9		
GO:0032675	regulation of interleu-kin-6 production	7.77456E-11	Il17f/Ddx58/Tlr2/Ereg/Lbp/Tlr1/Lgals9/Il10/Il17a/Ccr5/Il1a/Tnf/Mapk13/Il1rn/Tnfsf9/Zc3h12a/Il1b/Ifng/Ifih1/Nod2	20	BP
GO:0050777	negative regulation of immune response	8.77971E-11	Tap1/RT1-S3/Anxa1/Tap2/Parp14/Clec2d/Lgals9/Il10/Ceacam1/Adar/Trafd1/Fcrlb/RT1-A1/Tnf/Acod1/Samhd1/Zc3h12a/Dhx58/Nod2/Jak3	20	BP
GO:0030595	leukocyte chemotaxis	9.03682E-11	Cxcr3/Cd74/Anxa1/Ccl4/Lbp/Lgals9/Csf3r/Ccl3/Klrk1/Cx3cl1/Trpm2/Cxcl3/Cxcl16/Il1rn/Trem3/Cxcl6/Edn3/Il1b/Ifng/Cxcl10/Nod2/Cxcl1/Cxcl9	23	BP
GO:0042089	cytokine biosynthetic process	1.13602E-10	Il17f/Lag3/Tlr2/Ereg/Lbp/Tlr1/Il10/Il1a/Irf1/Zfp36/Tnf/Tnfrsf8/Il21/Irf7/Il1b/Bcl3/Ifng	17	BP
GO:0032609	interferon-gamma production	1.71935E-10	Scgb1a1/Slc11a1/Lgals9/Il10/Isg15/Klrk1/Runx3/Tnf/Ripk3/Il21/Il12rb1/Rnf19b/Tnfsf9/Zc3h12a/Il1b/Bcl3/Irf8/Nod2	18	BP
GO:0050729	positive regulation of inflammatory response	1.71935E-10	Il17f/Egfr/Tlr2/Lbp/C3/Il17a/Ccr5/Ccl3/Cx3cl1/Ido1/Tnf/Osm/Il1b/Gbp5/Il17c/Napepld/Nfkbiz/Nod2	18	BP
GO:0050707	regulation of cytokine secretion	1.78326E-10	Il17f/Ddx58/Anxa1/Tlr2/Socs1/Tlr1/Lgals9/Il10/Il17a/Ccr5/Il1a/Ccl3/Casp4/Cx3cl1/Tnf/Nlrp3/Osm/Gsdmd/Zc3h12a/Il1b/Ifng/Ifih1/Nod2	23	BP
GO:0042035	regulation of cytokine biosynthetic process	1.92579E-10	Il17f/Lag3/Tlr2/Ereg/Lbp/Tlr1/Il10/Il1a/Irf1/Zfp36/Tnf/Tnfrsf8/Il21/Il1b/Bcl3/Ifng	16	BP
GO:0042267	natural killer cell mediated cytotoxicity	2.12692E-10	Tap1/RT1-S3/Lag3/Tap2/Gzmb/Clec2d/Lgals9/Ceacam1/Klrk1/LOC102553861/RT1-A1/Gzmbl3/Il21/Rnf19b	14	BP
GO:0007159	leukocyte cell-cell adhesion	2.37043E-10	Scgb1a1/Cd74/RT1-S3/Lag3/Anxa1/Socs1/Lgals9/Ceacam1/Cd80/RT1-DMb/Runx3/Irf1/Ido1/Tnf/Socs6/Il21/Nlrp3/Ada/Il12rb1/Lilrb4/Tnfsf9/Zc3h12a/Cd86/Il1b/Ifng/Zc3h12d/Nfkbiz/Jak3	28	BP
GO:0001916	positive regulation of T cell mediated cyto-toxicity	2.63222E-10	RT1-S3/Tap2/RT1-N2/B2m/RT1-A1/RT1-CE10/RT1-CE5/RT1-A2/RT1-N3/RT1-T24-3/RT1-CE16	11	BP
GO:0032612	interleukin-1 produc-tion	3.62102E-10	Anxa1/Tlr2/Lgals9/Ccr5/Ceacam1/Ccl3/Casp4/Cx3cl1/Nlrp3/Gsdmd/Zc3h12a/Il1b/Gbp5/Ifng/Nod2/Mefv	16	BP
GO:0002228	natural killer cell mediated immunity	3.83958E-10	Tap1/RT1-S3/Lag3/Tap2/Gzmb/Clec2d/Lgals9/Ceacam1/Klrk1/LOC102553861/RT1-A1/Gzmbl3/Il21/Rnf19b	14	BP
GO:1903708	positive regulation of hemopoiesis	5.51741E-10	Cd74/Stat1/Anxa1/Socs1/Csf3/Lgals9/Il17a/Isg15/Thpo/Prdm1/Ccl3/Runx3/Tnf/Il21/Nlrp3/Ada/Lilrb4/Tnfsf9/Il1b/Ifng/Nfkbiz/Cxcl1	22	BP
GO:0050715	positive regulation of cytokine secretion	5.6881E-10	Il17f/Ddx58/Tlr2/Tlr1/Lgals9/Il10/Il17a/Ccr5/Il1a/Ccl3/Casp4/Tnf/Nlrp3/Osm/Gsdmd/Il1b/Ifng/Ifih1/Nod2	19	BP

GO:0046631	alpha-beta T cell activation	7.98637E-10	Sema4a/RT1-S3/Anxa1/Socs1/Batf/Lgals9/Ceacam1/Prdm1/Cd80/Rsad2/Runx3/Irf1/Nlrp3/Ada/Zc3h12a/Bcl3/Ifng/Nfkbiz/Jak3	19	BP
GO:0002440	production of molecular mediator of immune response	8.75302E-10	Cd74/Slc11a1/RT1-S3/Ddx58/Tlr2/Batf/Il10/Ceacam1/B2m/Pou2f2/Rsad2/Mmp7/Tnf/Samhd1/Il21/Nlrp3/Trem3/Il1b/Ifng/Sema7a/Nod2/Jak3	22	BP
GO:0060326	cell chemotaxis	8.92285E-10	Cxcr3/Cd74/Anxa1/Ccl4/Tiam1/Lbp/Lgals9/Csf3r/Ccr5/Ccl3/Klrk1/Cx3cl1/Trpm2/Cxcl3/Cxcl16/Il1rn/Trem3/Cxcl6/Edn3/Il1b/Ifng/Ccrl2/Cxcl10/Nod2/Cxcl1/Cxcl9	26	BP
GO:0050708	regulation of protein secretion	9.51908E-10	Abat/Il17f/RT1-S3/Ddx58/Egfr/Anxa1/Tlr2/Mmp13/Socs1/Tiam1/Nos2/Tlr1/Lgals9/Il10/Il17a/Ccr5/Ceacam1/Il1a/Ccl3/Casp4/Cx3cl1/Arntl/Rsad2/Trpm2/Tnf/Nlrp3/Osm/Gsdmd/Irs1/Zc3h12a/Il1b/Ifng/Ifih1/Nod2	34	BP
GO:0046632	alpha-beta T cell differentiation	1.54661E-09	Sema4a/Anxa1/Socs1/Batf/Lgals9/Prdm1/Rsad2/Runx3/Irf1/Nlrp3/Ada/Zc3h12a/Bcl3/Ifng/Nfkbiz/Jak3	16	BP
GO:0050866	negative regulation of cell activation	1.7001E-09	Scgb1a1/Abat/Cd74/Lag3/Anxa1/Gp5/Socs1/Lgals9/Il10/Ceacam1/Prdm1/Cd80/Cx3cl1/Runx3/Irf1/Ido1/Socs6/Zc3h12a/Cd86/Zc3h12d/Jak3	21	BP
GO:1903039	positive regulation of leukocyte cell-cell adhesion	1.7001E-09	Cd74/RT1-S3/Anxa1/Socs1/Lgals9/Ceacam1/Cd80/RT1-DMb/Runx3/Tnf/Il21/Nlrp3/Ada/Il12rb1/Lilrb4/Tnfsf9/Cd86/Il1b/Ifng/Nfkbiz/Jak3	21	BP
GO:0032649	regulation of interferon-gamma production	2.02794E-09	Scgb1a1/Slc11a1/Lgals9/Il10/Isg15/Klrk1/Tnf/Ripk3/Il21/Il12rb1/Tnfsf9/Zc3h12a/Il1b/Bcl3/Irf8/Nod2	16	BP
GO:0022408	negative regulation of cell-cell adhesion	2.03791E-09	Scgb1a1/Abat/Cd74/Lag3/Anxa1/Socs1/Lgals9/Il10/Bmp2/Ceacam1/Cd80/Runx3/Irf1/Ido1/Socs6/Il1rn/Zc3h12a/Cd86/Zc3h12d/Jak3	20	BP
GO:0022409	positive regulation of cell-cell adhesion	2.07999E-09	Cd74/RT1-S3/Anxa1/Socs1/Lgals9/Ccr5/Ceacam1/Cd80/Cx3cl1/RT1-DMb/Runx3/Tnf/Il21/Nlrp3/Ada/Il12rb1/Lilrb4/Tnfsf9/Cd86/Il1b/Ifng/Nfkbiz/Jak3	23	BP
GO:0071706	tumor necrosis factor superfamily cytokine production	2.10754E-09	Il17f/RT1-S3/Ddx58/Tlr2/Ccl4/Lbp/Tlr1/Lgals9/Il10/Ccr5/Ccl3/Cx3cl1/Zfp36/Tnfrsf8/Zc3h12a/Bcl3/Ifng/Ifih1/Nod2	19	BP
GO:0051249	regulation of lymphocyte activation	2.68317E-09	Scgb1a1/Cd74/RT1-S3/Lag3/Anxa1/Socs1/Lgals9/Il10/Ceacam1/Prdm1/Cd80/RT1-DMb/Runx3/Irf1/Ido1/Ripk3/Socs6/Il21/Nlrp3/Ada/Il12rb1/Lilrb4/Tnfsf9/Zc3h12a/Cd86/Il1b/Ifng/Zc3h12d/Nfkbiz/Nod2/Jak3	31	BP
GO:0002367	cytokine production involved in immune	2.82852E-09	Cd74/Slc11a1/Ddx58/Tlr2/Il10/B2m/Rsad2/Tnf/Il21/Nlrp3/Trem3/Il1b/Sema7a/Nod2/Jak3	15	BP

	response				
GO:0050870	positive regulation of T cell activation	3.28663E-09	Cd74/RT1-S3/Anxa1/Socs1/Lgals9/Ceacam1/Cd80/RT1-DMb/Runx3/Il21/Nlrp3/Ada/Il12rb1/Lilrb4/Tnfsf9/Cd86/Il1b/Ifng/Nfkbiz/Jak3	20	BP
GO:0046634	regulation of alpha-beta T cell activation	4.32298E-09	RT1-S3/Anxa1/Socs1/Lgals9/Ceacam1/Prdm1/Cd80/Runx3/Irf1/Nlrp3/Ada/Zc3h12a/Ifng/Nfkbiz/Jak3	15	BP
GO:0050868	negative regulation of T cell activation	4.41673E-09	Scgb1a1/Cd74/Lag3/Anxa1/Socs1/Lgals9/Ceacam1/Cd80/Runx3/Irf1/Ido1/Socs6/Zc3h12a/Cd86/Zc3h12d/Jak3	16	BP
GO:0051250	negative regulation of lymphocyte activation	4.84286E-09	Scgb1a1/Cd74/Lag3/Anxa1/Socs1/Lgals9/Il10/Ceacam1/Prdm1/Cd80/Runx3/Irf1/Ido1/Socs6/Zc3h12a/Cd86/Zc3h12d/Jak3	18	BP
GO:0050670	regulation of lymphocyte proliferation	4.85786E-09	Scgb1a1/Cd74/RT1-S3/Anxa1/Lgals9/Il10/Ceacam1/Prdm1/Cd80/RT1-DMb/Irf1/Ido1/Ripk3/Il21/Ada/Il12rb1/Tnfsf9/Cd86/Il1b/Ifng/Zc3h12d/Jak3	22	BP
GO:0032652	regulation of interleukin-1 production	4.99651E-09	Anxa1/Tlr2/Lgals9/Ccr5/Ceacam1/Ccl3/Casp4/Cx3cl1/Nlrp3/Gsdmd/Zc3h12a/Ifng/Nod2/Mefv	14	BP
GO:0032944	regulation of mononuclear cell proliferation	5.70482E-09	Scgb1a1/Cd74/RT1-S3/Anxa1/Lgals9/Il10/Ceacam1/Prdm1/Cd80/RT1-DMb/Irf1/Ido1/Ripk3/Il21/Ada/Il12rb1/Tnfsf9/Cd86/Il1b/Ifng/Zc3h12d/Jak3	22	BP
GO:0001818	negative regulation of cytokine production	5.75358E-09	Scgb1a1/Slc11a1/Lag3/Anxa1/Tlr2/Lbp/Dll1/Lgals9/Il10/Ceacam1/Cx3cl1/Zfp36/Ido1/Tnf/Acod1/Nlrp3/Zc3h12a/Dhx58/Bcl3/Ifng/Nod2/Mefv/Jak3	23	BP
GO:0032069	regulation of nuclease activity	5.79787E-09	Oasl/Oasl2/Oas1e/Oas1a/Oas3/Oas1b/Oas2/Oas1i/Oas1h	9	BP
GO:0030099	myeloid cell differentiation	7.24677E-09	Cd74/Stat1/Tlr2/Batf/Csf3/Dll1/Clec2d/Csf3r/Il17a/Ceacam1/Isg15/Thpo/Ccl3/Adar/B2m/Mafb/Hoxb7/Zfp36/Tnf/Sbno2/Hhex/Lilrb4/Tnfsf9/Cd86/Ubd/Ifng/Il17c/Irf8/Jak3	29	BP
GO:0050714	positive regulation of protein secretion	7.3455E-09	Abat/Il17f/RT1-S3/Ddx58/Egfr/Tlr2/Mmp13/Tlr1/Lgals9/Il10/Il17a/Ccr5/Ceacam1/Il1a/Ccl3/Casp4/Trpm2/Tnf/Nlrp3/Osm/Gsdmd/Il1b/Ifng/Ifih1/Nod2	25	BP
GO:0097529	myeloid leukocyte migration	7.4645E-09	Cd74/Anxa1/Ccl4/Lbp/Csf3r/Il17a/Il1a/Ccl3/Cx3cl1/Cxcl3/Il1rn/Trem3/Cxcl6/Edn3/Il1b/Ifng/Cxcl10/Nod2/Cxcl1/Cxcl9	20	BP
GO:0042108	positive regulation of cytokine biosynthetic process	7.61702E-09	Tlr2/Ereg/Lbp/Tlr1/Il1a/Irf1/Tnf/Tnfrsf8/Il21/Il1b/Bcl3/Ifng	12	BP
GO:0032680	regulation of tumor necrosis factor production	8.11132E-09	RT1-S3/Ddx58/Tlr2/Ccl4/Lbp/Tlr1/Lgals9/Il10/Ccr5/Ccl3/Cx3cl1/Zfp36/Tnfrsf8/Zc3h12a/Bcl3/Ifng/Ifih1/Nod2	18	BP

GO:0030217	T cell differentiation	8.35638E-09	Cd74/Sema4a/Anxa1/Socs1/Batf/Lgals9/Prdm1/B2m/Rsad2/Runx3/Mafb/Irf1/Ripk3/Nlrp3/Ada/Lilrb4/Tnfsf9/Zc3h12a/Il1b/Bcl3/Ifng/Nfkbiz/Jak3	23	BP
GO:0043367	CD4-positive, alpha-beta T cell differentiation	8.41887E-09	Sema4a/Anxa1/Socs1/Batf/Lgals9/Rsad2/Runx3/Nlrp3/Zc3h12a/Bcl3/Ifng/Nfkbiz/Jak3	13	BP
GO:0002695	negative regulation of leukocyte activation	9.21992E-09	Scgb1a1/Cd74/Lag3/Anxa1/Socs1/Lgals9/Il10/Ceacam1/Prdm1/Cd80/Cx3cl1/Runx3/Irf1/Ido1/Socs6/Zc3h12a/Cd86/Zc3h12d/Jak3	19	BP
GO:0032640	tumor necrosis factor production	9.91283E-09	RT1-S3/Ddx58/Tlr2/Ccl4/Lbp/Tlr1/Lgals9/Il10/Ccr5/Ccl3/Cx3cl1/Zfp36/Tnfrsf8/Zc3h12a/Bcl3/Ifng/Ifih1/Nod2	18	BP
GO:1903555	regulation of tumor necrosis factor superfamily cytokine production	9.91283E-09	RT1-S3/Ddx58/Tlr2/Ccl4/Lbp/Tlr1/Lgals9/Il10/Ccr5/Ccl3/Cx3cl1/Zfp36/Tnfrsf8/Zc3h12a/Bcl3/Ifng/Ifih1/Nod2	18	BP
GO:0035710	CD4-positive, alpha-beta T cell activation	1.04597E-08	Sema4a/Anxa1/Socs1/Batf/Lgals9/Ceacam1/Rsad2/Runx3/Nlrp3/Zc3h12a/Bcl3/Ifng/Nfkbiz/Jak3	14	BP
GO:0072593	reactive oxygen species metabolic process	1.05086E-08	Il22/Egfr/Bco2/Tlr2/Nos2/Nox1/Hk2/Il10/Duox2/Duoxa2/Ncf1/Klrk1/Pmaip1/Duox1/Birc3/Tnf/Ripk3/Ncf4/Acod1/Noxo1/Zc3h12a/Il1b/Ifng/Cxcl1	24	BP
GO:0034340	response to type I interferon	1.09521E-08	Stat2/Stat1/Isg15/Adar/Zbp1/Ifi27/Mx2/Samhd1/Irf7/Oas2	10	BP
GO:0002793	positive regulation of peptide secretion	1.10145E-08	Abat/Il17f/RT1-S3/Ddx58/Egfr/Tlr2/Mmp13/Tlr1/Lgals9/Il10/Il17a/Ccr5/Ceacam1/Il1a/Ccl3/Casp4/Trpm2/Tnf/Nlrp3/Osm/Gsdmd/Irs1/Il1b/Ifng/Ifih1/Nod2	26	BP
GO:0070663	regulation of leukocyte proliferation	1.15044E-08	Scgb1a1/Cd74/RT1-S3/Anxa1/Lgals9/Il10/Ceacam1/Prdm1/Cd80/RT1-DMb/Irf1/Ido1/Ripk3/Il21/Ada/Il12rb1/Tnfsf9/Cd86/Il1b/Ifng/Zc3h12d/Jak3	22	BP
GO:0002718	regulation of cytokine production involved in immune response	1.16349E-08	Cd74/Ddx58/Tlr2/Il10/B2m/Rsad2/Tnf/Il21/Nlrp3/Il1b/Sema7a/Nod2/Jak3	13	BP
GO:0002286	T cell activation involved in immune response	1.20548E-08	Sema4a/Slc11a1/Anxa1/Batf/Lgals9/Ceacam1/RT1-DMb/Nlrp3/Zc3h12a/Cd86/Bcl3/Ifng/Nfkbiz/Jak3	14	BP
GO:0035821	modulation of process of other organism	1.23451E-08	Tlr2/Ccl4/Nos2/Ccr5/Ceacam1/Ccl3/Ifi27/Ncf1/Trem3/Gsdmd/Cxcl6/Mmp9/Zc3h12a/Ifng/Napepld/Lyz2/Cxcl1	17	BP
GO:0032655	regulation of interleukin-12 production	1.25101E-08	Tlr2/Lgals9/Il10/Irf1/Ido1/Tnfsf9/Ifng/Irf8/Nod2/Mefv/Jak3	11	BP
GO:0050867	positive regulation of cell activation	1.32784E-08	Cd74/RT1-S3/Anxa1/Socs1/Lbp/Lgals9/Il10/Plek/Ceaca	26	BP

			m1/Prdm1/Klrk1/Cd80/RT1-DMb/Runx3/Il21/Nlrp3/Ada/Il12rb1/Lilrb4/Tnfsf9/Cd86/Il1b/Ifng/Nfkbiz/Nod2/Jak3		
GO:0070661	leukocyte proliferation	1.41956E-08	Scgb1a1/Cd74/Itgad/Slc11a1/RT1-S3/Anxa1/Lgals9/Il10/Ceacam1/Prdm1/Cd80/Cx3cl1/RT1-DMb/Irf1/Ido1/Ripk3/Il21/Ada/Il12rb1/Tnfsf9/Cd86/Il1b/Ifng/Zc3h12d/Jak3	25	BP
GO:1903038	negative regulation of leukocyte cell-cell adhesion	1.63237E-08	Scgb1a1/Cd74/Lag3/Anxa1/Socs1/Lgals9/Ceacam1/Cd80/Runx3/Irf1/Ido1/Socs6/Zc3h12a/Cd86/Zc3h12d/Jak3	16	BP
GO:0030098	lymphocyte differentiation	1.83439E-08	Cd74/Sema4a/Anxa1/Socs1/Batf/Dll1/Lgals9/Prdm1/B2m/Pou2f2/Rsad2/Runx3/Mafb/Irf1/Ripk3/Hhex/Il21/Nlrp3/Ada/Lilrb4/Tnfsf9/Zc3h12a/Il1b/Bcl3/Ifng/Nfkbiz/Jak3	27	BP
GO:0032611	interleukin-1 beta production	1.85606E-08	Tlr2/Lgals9/Ccr5/Ccl3/Casp4/Nlrp3/Gsdmd/Zc3h12a/Il1b/Gbp5/Ifng/Nod2/Mefv	13	BP
GO:1990868	response to chemokine	1.85606E-08	Cxcr3/Ccl4/Ccr5/Thpo/Ccl3/Cx3cl1/Cxcl3/Cxcl6/Zc3h12a/Ccrl2/Cxcl10/Cxcl1/Cxcl9	13	BP
GO:1990869	cellular response to chemokine	1.85606E-08	Cxcr3/Ccl4/Ccr5/Thpo/Ccl3/Cx3cl1/Cxcl3/Cxcl6/Zc3h12a/Ccrl2/Cxcl10/Cxcl1/Cxcl9	13	BP
GO:0046651	lymphocyte proliferation	2.06104E-08	Scgb1a1/Cd74/Itgad/Slc11a1/RT1-S3/Anxa1/Lgals9/Il10/Ceacam1/Prdm1/Cd80/RT1-DMb/Irf1/Ido1/Ripk3/Il21/Ada/Il12rb1/Tnfsf9/Cd86/Il1b/Ifng/Zc3h12d/Jak3	24	BP
GO:0042098	T cell proliferation	2.07207E-08	Scgb1a1/Itgad/Slc11a1/RT1-S3/Anxa1/Lgals9/Ceacam1/Cd80/RT1-DMb/Irf1/Ido1/Ripk3/Il21/Il12rb1/Tnfsf9/Cd86/Il1b/Ifng/Zc3h12d/Jak3	20	BP
GO:0032615	interleukin-12 production	2.30748E-08	Tlr2/Lgals9/Il10/Irf1/Ido1/Tnfsf9/Ifng/Irf8/Nod2/Mefv/Jak3	11	BP
GO:0032943	mononuclear cell proliferation	2.34954E-08	Scgb1a1/Cd74/Itgad/Slc11a1/RT1-S3/Anxa1/Lgals9/Il10/Ceacam1/Prdm1/Cd80/RT1-DMb/Irf1/Ido1/Ripk3/Il21/Ada/Il12rb1/Tnfsf9/Cd86/Il1b/Ifng/Zc3h12d/Jak3	24	BP
GO:0002696	positive regulation of leukocyte activation	2.35608E-08	Cd74/RT1-S3/Anxa1/Socs1/Lbp/Lgals9/Il10/Ceacam1/Prdm1/Klrk1/Cd80/RT1-DMb/Runx3/Il21/Nlrp3/Ada/Il12rb1/Lilrb4/Tnfsf9/Cd86/Il1b/Ifng/Nfkbiz/Nod2/Jak3	25	BP
GO:0050688	regulation of defense response to virus	3.1337E-08	Parp9/Apobec3/Ddx58/Stat1/Dtx3l/Zc3hav1/Trim15/Il12rb1/Zc3h12a/Il1b/Dhx58/Ifng	12	BP
GO:0002474	antigen processing and presentation of peptide antigen via MHC class I	3.17735E-08	Tap1/Tap2/RT1-CE4/B2m/RT1-A1/RT1-CE10/RT1-CE5/RT1-A2	8	BP
GO:0042129	regulation of T cell proliferation	3.39309E-08	Scgb1a1/RT1-S3/Anxa1/Lgals9/Ceacam1/Cd80/RT1-DMb/Irf1/Ido1/Ripk3/Il21/Il12rb1/Tnfsf9/Cd86/Il1b/Ifng/Zc3h12d/Jak3	18	BP
GO:0045621	positive regulation of	3.52335E-08	Cd74/Anxa1/Socs1/Lgals9/Prdm1/Runx3/Il2	14	BP

	lymphocyte differentiation		1/Nlrp3/Ada/Lilrb4/Tnfsf9/Il1b/Ifng/Nfkbiz		
GO:0070098	chemokine-mediated signaling pathway	3.68889E-08	Cxcr3/Ccl4/Ccr5/Thpo/Ccl3/Cx3cl1/Cxcl3/Cxcl6/Ccrl2/Cxcl10/Cxcl1/Cxcl9	12	BP
GO:0032602	chemokine production	4.44965E-08	Cxcr3/Cd74/Tlr2/Lbp/Lgals9/Il1a/Tnf/Eif2ak2/Cxcl6/Il1b/Ifng/Nod2/Mefv	13	BP
GO:0042832	defense response to protozoan	4.50442E-08	Slc11a1/Batf/Gbp4/Il10/Gbp2/Bcl3/Ifng/Irf8	8	BP
GO:0045953	negative regulation of natural killer cell mediated cytotoxicity	4.99974E-08	Tap1/RT1-S3/Tap2/Clec2d/Lgals9/Ceacam1/RT1-A1	7	BP
GO:0032637	interleukin-8 production	5.07041E-08	Il17f/Ddx58/Anxa1/Tlr2/Lbp/Nos2/Tlr1/Lgals9/Tnf/Il1b/Bcl3/Nod2	12	BP
GO:0060760	positive regulation of response to cytokine stimulus	5.59288E-08	Parp9/Cd74/Ddx58/Tlr2/Parp14/Zbp1/Casp4/Irf7/Irgm/Ifih1	10	BP
GO:1902105	regulation of leukocyte differentiation	5.73038E-08	Cd74/Anxa1/Socs1/Clec2d/Lgals9/Il17a/Ceacam1/Prdm1/Ccl3/Runx3/Mafb/Irf1/Tnf/Il21/Nlrp3/Ada/Lilrb4/Tnfsf9/Zc3h12a/Il1b/Ifng/Nfkbiz/Jak3	23	BP
GO:0002700	regulation of production of molecular mediator of immune response	6.46403E-08	Cd74/RT1-S3/Ddx58/Tlr2/Il10/Ceacam1/B2m/Rsad2/Tnf/Il21/Nlrp3/Il1b/Ifng/Sema7a/Nod2/Jak3	16	BP
GO:0045580	regulation of T cell differentiation	7.13854E-08	Cd74/Anxa1/Socs1/Lgals9/Prdm1/Runx3/Irf1/Nlrp3/Ada/Lilrb4/Tnfsf9/Zc3h12a/Il1b/Ifng/Nfkbiz/Jak3	16	BP
GO:0002716	negative regulation of natural killer cell mediated immunity	7.77132E-08	Tap1/RT1-S3/Tap2/Clec2d/Lgals9/Ceacam1/RT1-A1	7	BP
GO:0051251	positive regulation of lymphocyte activation	7.7853E-08	Cd74/RT1-S3/Anxa1/Socs1/Lgals9/Ceacam1/Prdm1/Cd80/RT1-DMb/Runx3/Il21/Nlrp3/Ada/Il12rb1/Lilrb4/Tnfsf9/Cd86/Il1b/Ifng/Nfkbiz/Nod2/Jak3	22	BP
GO:1902107	positive regulation of leukocyte differentiation	7.90147E-08	Cd74/Anxa1/Socs1/Lgals9/Il17a/Prdm1/Ccl3/Runx3/Tnf/Il21/Nlrp3/Ada/Lilrb4/Tnfsf9/Il1b/Ifng/Nfkbiz	17	BP
GO:0098586	cellular response to virus	8.45591E-08	Ddx58/Lgals9/Adar/Zc3hav1/Psmb9/Trim15/Zc3h12a/Irgm/Dhx58/Ifih1	10	BP
GO:0070555	response to interleukin-1	8.64563E-08	Anxa1/Ccl4/Nos2/Lgals9/Il17a/Il1a/Ccl3/Serpina3n/Cx3cl1/Irf1/Psmb9/Mapk13/Acod1/Gbp2/Il1rn/Mmp9/Zc3h12a	17	BP
GO:0002720	positive regulation of cytokine production involved in immune response	1.03202E-07	Cd74/Ddx58/Tlr2/B2m/Rsad2/Il21/Nlrp3/Il1b/Sema7a/Nod2	10	BP
GO:0050691	regulation of defense response to virus by host	1.28598E-07	Parp9/Ddx58/Stat1/Dtx3l/Zc3hav1/Il12rb1/Zc3h12a/Il1b/Ifng	9	BP
GO:0060759	regulation of response	1.31385E-07	Parp9/Cd74/Ddx58/Tlr2/Socs1/Parp14/Adar/	14	BP

	to cytokine stimulus		Zbp1/Casp4/Samhd1/Il1rn/Irf7/Irgm/Ifih1		
GO:0071347	cellular response to interleukin-1	1.34904E-07	Ccl4/Nos2/Il17a/Il1a/Ccl3/Serpina3n/Cx3cl1/Irf1/Psmb9/Mapk13/Acod1/Gbp2/Il1rn/Mmp9/Zc3h12a	15	BP
GO:0046637	regulation of alpha-beta T cell differentiation	1.37475E-07	Anxa1/Socs1/Lgals9/Prdm1/Runx3/Nlrp3/Ada/Zc3h12a/Ifng/Nfkbiz/Jak3	11	BP
GO:0032760	positive regulation of tumor necrosis factor production	1.45236E-07	RT1-S3/Ddx58/Tlr2/Ccl4/Lbp/Tlr1/Lgals9/Ccr5/Ccl3/Tnfrsf8/Ifng/Ifih1/Nod2	13	BP
GO:0001562	response to protozoan	1.56589E-07	Slc11a1/Batf/Gbp4/Il10/Gbp2/Bcl3/Ifng/Irf8	8	BP
GO:0046635	positive regulation of alpha-beta T cell activation	1.61366E-07	RT1-S3/Anxa1/Socs1/Lgals9/Ceacam1/Cd80/Runx3/Nlrp3/Ada/Ifng/Nfkbiz	11	BP
GO:0002534	cytokine production involved in inflammatory response	1.61686E-07	Il17f/Nos2/Il17a/Ido1/Zc3h12a/Gbp5/Il17c/Nod2/Mefv	9	BP
GO:1903557	positive regulation of tumor necrosis factor superfamily cytokine production	1.64254E-07	RT1-S3/Ddx58/Tlr2/Ccl4/Lbp/Tlr1/Lgals9/Ccr5/Ccl3/Tnfrsf8/Ifng/Ifih1/Nod2	13	BP
GO:0060333	interferon-gamma-mediated signaling pathway	1.72835E-07	Parp9/Stat1/Socs1/Parp14/Irf1/Irgm/Ifng	7	BP
GO:0042269	regulation of natural killer cell mediated cytotoxicity	1.82569E-07	Tap1/RT1-S3/Lag3/Tap2/Clec2d/Lgals9/Ceacam1/Klrk1/RT1-A1/Il21	10	BP
GO:0051817	modulation of process of other organism involved in symbiotic interaction	1.85475E-07	Tlr2/Ccl4/Ccr5/Ceacam1/Ccl3/Ifi27/Ncf1/Trem3/Cxcl6/Mmp9/Zc3h12a/Napepld/Cxcl1	13	BP
GO:0032642	regulation of chemokine production	1.8827E-07	Cd74/Tlr2/Lbp/Lgals9/Il1a/Tnf/Eif2ak2/Cxcl6/Il1b/Ifng/Nod2/Mefv	12	BP
GO:0032677	regulation of interleukin-8 production	1.88862E-07	Il17f/Ddx58/Anxa1/Tlr2/Lbp/Tlr1/Lgals9/Tnf/Il1b/Bcl3/Nod2	11	BP
GO:0045619	regulation of lymphocyte differentiation	2.04916E-07	Cd74/Anxa1/Socs1/Lgals9/Prdm1/Runx3/Irf1/Il21/Nlrp3/Ada/Lilrb4/Tnfsf9/Zc3h12a/Il1b/Ifng/Nfkbiz/Jak3	17	BP
GO:1902106	negative regulation of leukocyte differentiation	2.09123E-07	Cd74/Anxa1/Socs1/Clec2d/Ceacam1/Ccl3/Runx3/Mafb/Irf1/Lilrb4/Zc3h12a/Ifng/Jak3	13	BP
GO:0050730	regulation of peptidyl-tyrosine phosphorylation	2.1629E-07	Parp9/Cd74/Il22/Egfr/Socs1/Ereg/Csf3/Parp14/Socs3/Ncf1/Cd80/Unc119/Irf1/Agrn/Epgn/Il21/Osm/Ifng/Cck/Areg/Nod2	21	BP
GO:0002715	regulation of natural killer cell mediated immunity	2.1894E-07	Tap1/RT1-S3/Lag3/Tap2/Clec2d/Lgals9/Ceacam1/Klrk1/RT1-A1/Il21	10	BP
GO:0050830	defense response to Gram-positive bacterium	2.35438E-07	RT1-S3/Tlr2/Gbp4/Lbp/Ncf1/Klrk1/Mmp7/Tnf/Nlrp3/Gbp2/Gsdmd/Lyz2/Nod2	13	BP
GO:0002685	regulation of leuko-	2.47405E-07	Cxcr3/Cd74/Anxa1/Tlr2/Ccl4/Lbp/Lgals9/Il1	18	BP

	cyte migration		a/Ccl3/Klrk1/Cx3cl1/Ripk3/Ada/Mmp9/Edn3/Il1b/Cxcl10/Nod2		
GO:0001911	negative regulation of leukocyte mediated cytotoxicity	2.48854E-07	Tap1/RT1-S3/Tap2/Clec2d/Lgals9/Ceacam1/RT1-A1	7	BP
GO:0002702	positive regulation of production of molecular mediator of immune response	2.64682E-07	Cd74/RT1-S3/Ddx58/Tlr2/Ceacam1/B2m/Rsad2/Il21/Nlrp3/Il1b/Ifng/Sema7a/Nod2	13	BP
GO:2000106	regulation of leukocyte apoptotic process	2.64682E-07	Cd74/Anxa1/Pdcd1/Lgals9/Il10/Ccr5/Ceacam1/Ido1/Ripk3/Ada/Hsh2d/Nod2/Jak3	13	BP
GO:1904951	positive regulation of establishment of protein localization	2.76859E-07	Abat/Il17f/RT1-S3/Ddx58/Egfr/Tlr2/Mmp13/Tlr1/Lgals9/Il10/Il17a/Ccr5/Ceacam1/Il1a/Ccl3/Casp4/Pmaip1/Trpm2/Tnf/Nlrp3/Osm/Gsdmd/Zc3h12a/Il1b/Ifng/Plk3/Ifih1/Nod2/Cdk5r1	29	BP
GO:0032651	regulation of interleukin-1 beta production	2.9787E-07	Tlr2/Lgals9/Ccr5/Ccl3/Casp4/Nlrp3/Gsdmd/Zc3h12a/Ifng/Nod2/Mefv	11	BP
GO:0002366	leukocyte activation involved in immune response	3.13294E-07	Sema4a/Slc11a1/Anxa1/Batf/Lbp/Dll1/Lgals9/Ceacam1/Ccl3/RT1-DMb/Sbno2/Nlrp3/Ada/Zc3h12a/Cd86/Bcl3/Ifng/Nfkbiz/Anxa3/Jak3	20	BP
GO:0042102	positive regulation of T cell proliferation	3.72929E-07	RT1-S3/Anxa1/Lgals9/Ceacam1/Cd80/RT1-DMb/Il21/Il12rb1/Tnfsf9/Cd86/Il1b/Ifng/Jak3	13	BP
GO:0018108	peptidyl-tyrosine phosphorylation	3.91417E-07	Parp9/Cd74/Il22/Egfr/Socs1/Ereg/Csf3/Parp14/Socs3/Ncf1/Cd80/Unc119/Irf1/Agrn/Epgn/Tesk2/Eif2ak2/Il21/Il12rb1/Osm/Ifng/Cck/Areg/Nod2/Jak3	25	BP
GO:0051222	positive regulation of protein transport	4.09338E-07	Abat/Il17f/RT1-S3/Ddx58/Egfr/Tlr2/Mmp13/Tlr1/Lgals9/Il10/Il17a/Ccr5/Ceacam1/Il1a/Ccl3/Casp4/Trpm2/Tnf/Nlrp3/Osm/Gsdmd/Zc3h12a/Il1b/Ifng/Plk3/Ifih1/Nod2/Cdk5r1	28	BP
GO:0045582	positive regulation of T cell differentiation	4.11261E-07	Cd74/Anxa1/Socs1/Lgals9/Runx3/Nlrp3/Ada/Lilrb4/Tnfsf9/Il1b/Ifng/Nfkbiz	12	BP
GO:0002274	myeloid leukocyte activation	4.16436E-07	Slc11a1/Pla2g5/Tlr2/Batf/Lbp/Fcgr3a/Tlr1/Lgals9/Il10/Ccl3/Klrk1/Cx3cl1/Sbno2/Tnfsf9/Cxcl6/Cd86/Ubd/Anxa3	18	BP
GO:0071887	leukocyte apoptotic process	4.21406E-07	Cd74/Anxa1/Pdcd1/Lgals9/Il10/Ccr5/Ceacam1/Ido1/Ripk3/Ada/Hsh2d/Ifng/Nod2/Jak3	14	BP
GO:0002263	cell activation involved in immune response	4.33534E-07	Sema4a/Slc11a1/Anxa1/Batf/Lbp/Dll1/Lgals9/Ceacam1/Ccl3/RT1-DMb/Sbno2/Nlrp3/Ada/Zc3h12a/Cd86/Bcl3/Ifng/Nfkbiz/Anxa3/Jak3	20	BP
GO:0002294	CD4-positive, alpha-beta T cell differentiation involved in immune response	4.3589E-07	Sema4a/Anxa1/Batf/Lgals9/Nlrp3/Zc3h12a/Bcl3/Ifng/Nfkbiz/Jak3	10	BP
GO:0032722	positive regulation of chemokine production	4.3589E-07	Cd74/Tlr2/Lbp/Lgals9/Il1a/Tnf/Eif2ak2/Il1b/Ifng/Nod2	10	BP

GO:0032735	positive regulation of interleukin-12 production	4.50764E-07	Tlr2/Lgals9/Irf1/Ido1/Tnfsf9/Ifng/Irf8/Nod2	8	BP
GO:0060337	type I interferon signaling pathway	4.50764E-07	Stat2/Stat1/Adar/Zbp1/Ifi27/Samhd1/Irf7/Oas2	8	BP
GO:0071357	cellular response to type I interferon	4.50764E-07	Stat2/Stat1/Adar/Zbp1/Ifi27/Samhd1/Irf7/Oas2	8	BP
GO:0018212	peptidyl-tyrosine modification	4.55378E-07	Parp9/Cd74/Il22/Egfr/Socs1/Ereg/Csf3/Parp14/Socs3/Ncf1/Cd80/Unc119/Irf1/Agrn/Epgn/Tesk2/Eif2ak2/Il21/Il12rb1/Osm/Ifng/Cck/Areg/Nod2/Jak3	25	BP
GO:0050671	positive regulation of lymphocyte proliferation	4.68751E-07	Cd74/RT1-S3/Anxa1/Lgals9/Ceacam1/Cd80/RT1-DMb/Il21/Ada/Il12rb1/Tnfsf9/Cd86/Il1b/Ifng/Jak3	15	BP
GO:1903707	negative regulation of hemopoiesis	5.12819E-07	Cd74/Anxa1/Socs1/Dll1/Clec2d/Ceacam1/Ccl3/Runx3/Mafb/Irf1/Zfp36/Lilrb4/Zc3h12a/Ifng/Jak3	15	BP
GO:0002293	alpha-beta T cell differentiation involved in immune response	5.13229E-07	Sema4a/Anxa1/Batf/Lgals9/Nlrp3/Zc3h12a/Bcl3/Ifng/Nfkbiz/Jak3	10	BP
GO:2000514	regulation of CD4-positive, alpha-beta T cell activation	5.13229E-07	Anxa1/Socs1/Lgals9/Ceacam1/Runx3/Nlrp3/Zc3h12a/Ifng/Nfkbiz/Jak3	10	BP
GO:0032946	positive regulation of mononuclear cell proliferation	5.60586E-07	Cd74/RT1-S3/Anxa1/Lgals9/Ceacam1/Cd80/RT1-DMb/Il21/Ada/Il12rb1/Tnfsf9/Cd86/Il1b/Ifng/Jak3	15	BP
GO:0042092	type 2 immune response	5.73511E-07	Cd74/Anxa1/Batf/Rsad2/Ido1/Nlrp3/Bcl3/Nod2	8	BP
GO:0002287	alpha-beta T cell activation involved in immune response	6.02316E-07	Sema4a/Anxa1/Batf/Lgals9/Nlrp3/Zc3h12a/Bcl3/Ifng/Nfkbiz/Jak3	10	BP
GO:0044117	growth of symbiont in host	6.63652E-07	Tlr2/Lbp/Il10/Tnf/Ifng/Irf8/Nod2	7	BP
GO:0002224	toll-like receptor signaling pathway	6.68433E-07	Tlr2/Lbp/Tlr1/Lgals9/Tlr10/Rsad2/Irf1/Tnf/Acod1/Irf7/Cd86/Nod2	12	BP
GO:0032731	positive regulation of interleukin-1 beta production	6.78277E-07	Tlr2/Lgals9/Ccr5/Ccl3/Casp4/Nlrp3/Gsdmd/Ifng/Nod2	9	BP
GO:0043370	regulation of CD4-positive, alpha-beta T cell differentiation	6.78277E-07	Anxa1/Socs1/Lgals9/Runx3/Nlrp3/Zc3h12a/Ifng/Nfkbiz/Jak3	9	BP
GO:0006919	activation of cysteine-type endopeptidase activity involved in apoptotic process	6.93139E-07	Stat1/Ifi27l2b/Casp12/Ifi27/Casp4/Pmaip1/Apaf1/Bid/Tnf/Nlrp3/Cck	11	BP
GO:0002285	lymphocyte activation involved in immune response	7.17068E-07	Sema4a/Slc11a1/Anxa1/Batf/Dll1/Lgals9/Ceacam1/RT1-DMb/Nlrp3/Ada/Zc3h12a/Cd86/Bcl3/Ifng/Nfkbiz/Jak3	16	BP

GO:0002698	negative regulation of immune effector process	8.28428E-07	Tap1/RT1-S3/Anxa1/Tap2/Clec2d/Lgals9/Il10/Ceacam1/RT1-A1/Tnf/Zc3h12a/Dhx58/Nod2/Jak3	14	BP
GO:0031342	negative regulation of cell killing	8.9156E-07	Tap1/RT1-S3/Tap2/Clec2d/Lgals9/Ceacam1/RT1-A1	7	BP
GO:0070665	positive regulation of leukocyte proliferation	9.41431E-07	Cd74/RT1-S3/Anxa1/Lgals9/Ceacam1/Cd80/RT1-DMb/Il21/Ada/Il12rb1/Tnfsf9/Cd86/Il1b/Ifng/Jak3	15	BP
GO:0032621	interleukin-18 production	1.00106E-06	Tlr2/Tnf/Nlrp3/Gbp5/Nod2	5	BP
GO:0002753	cytoplasmic pattern recognition receptor signaling pathway	1.12675E-06	Ddx58/Zc3hav1/Trim15/Tifa/Irgm/Dhx58/Ifih1/Nod2	8	BP
GO:1900015	regulation of cytokine production involved in inflammatory response	1.12675E-06	Il17f/Nos2/Il17a/Zc3h12a/Gbp5/Il17c/Nod2/Mefv	8	BP
GO:0043902	positive regulation of multi-organism process	1.14482E-06	Cd74/Tmprss2/Tlr2/Nos2/Lhfpl2/Lgals9/Ccr5/Ceacam1/Adar/Zc3hav1/Trim15/Acod1/Dhx58/Ifng/Nod2/Cxcl1	16	BP
GO:0002833	positive regulation of response to biotic stimulus	1.16278E-06	Tlr2/Lgals9/Klrk1/Zc3hav1/Trim15/Acod1/Dhx58/Nod2/Cxcl1	9	BP
GO:0044110	growth involved in symbiotic interaction	1.18162E-06	Tlr2/Lbp/Il10/Tnf/Ifng/Irf8/Nod2	7	BP
GO:0002292	T cell differentiation involved in immune response	1.28162E-06	Sema4a/Anxa1/Batf/Lgals9/Nlrp3/Zc3h12a/Bcl3/Ifng/Nfkbiz/Jak3	10	BP
GO:1903532	positive regulation of secretion by cell	1.31316E-06	Abat/Il17f/RT1-S3/Ddx58/Egfr/Tlr2/Mmp13/Tlr1/Lgals9/Il10/Il17a/Ccr5/Ceacam1/Il1a/Ccl3/Casp4/Trpm2/Tnf/Nlrp3/Osm/Gsdmd/Irs1/Edn3/Il1b/Ifng/Cck/Ifih1/Nod2	28	BP
GO:0007249	I-kappaB kinase/NF-kappaB signaling	1.47047E-06	Cd74/Stat1/Tlr2/Litaf/Il1a/Cx3cl1/Irf1/Zc3hav1/Tnf/Ripk3/Tifa/Il1rn/Zc3h12a/Il1b/Ubd/Bcl3/Nod2	17	BP
GO:0002532	production of molecular mediator involved in inflammatory response	1.47815E-06	Il17f/Lbp/Nos2/Il17a/Ido1/Zc3h12a/Gbp5/Il17c/Nod2/Mefv	10	BP
GO:0043331	response to dsRNA	1.63116E-06	Ddx58/Stat1/Ciita/Pmaip1/Irf1/Zc3hav1/Eif2ak2/Ifih1/Nod2	9	BP
GO:0032102	negative regulation of response to external stimulus	1.79462E-06	Abat/Sema4a/Il22/Pla2g5/Gp5/Socs3/Lgals9/Il10/Ceacam1/Prdm1/Klrk1/Cx3cl1/Zfp36/Acod1/Nlrp3/Ada/Zc3h12a/Dhx58/Cck/Sema7a/Nod2/Mefv	22	BP
GO:0070228	regulation of lymphocyte apoptotic process	1.95129E-06	Cd74/Pdcd1/Lgals9/Il10/Ceacam1/Ido1/Ripk3/Ada/Hsh2d/Jak3	10	BP
GO:0002507	tolerance induction	2.00162E-06	Pdcd1/C3/Lgals9/RT1-A1/Ido1/Acod1/Cd86	7	BP
GO:0045637	regulation of myeloid cell differentiation	2.36057E-06	Cd74/Stat1/Csf3/Dll1/Clec2d/Csf3r/Il17a/Ceacam1/Isg15/Thpo/Ccl3/B2m/Mafb/Zfp36/Tn	17	BP

			f/Lilrb4/Ifng		
GO:0032732	positive regulation of interleukin-1 produc- tion	2.63538E-06	Tlr2/Lgals9/Ccr5/Ccl3/Casp4/Nlrp3/Gsdmd/Ifng/Nod2	9	BP
GO:0007162	negative regulation of cell adhesion	2.7082E-06	Scgb1a1/Abat/Cd74/Lag3/Anxa1/Socs1/Lgals9/Il10/Bmp2/Ceacam1/Cd80/Runx3/Irf1/Ido1/Socs6/Il1rn/Zc3h12a/Cd86/Zc3h12d/Jak3	20	BP
GO:0002374	cytokine secretion involved in immune response	2.75191E-06	Tlr2/Il10/Tnf/Nlrp3/Trem3/Nod2	6	BP
GO:1901222	regulation of NIK/NF-kappaB signaling	2.75259E-06	Egfr/Tlr2/Lgals9/Litaf/Birc3/Tnf/Eif2ak2/Nlrp3/Zc3h12a/Il1b/Bcl3/Nod2	12	BP
GO:2000116	regulation of cysteine-type endopeptidase activity	3.04121E-06	Stat1/Ifi27l2b/Casp12/Lgals9/Plaur/Mical1/Ifi27/Casp4/Pmaip1/Apaf1/Bid/Birc3/Tnf/Psmb9/Nlrp3/Mmp9/Cck/Mefv	18	BP
GO:0043330	response to exogenous dsRNA	3.04348E-06	Ddx58/Stat1/Ciita/Irf1/Zc3hav1/Eif2ak2/Ifih1/Nod2	8	BP
GO:0007260	tyrosine phosphoryla- tion of STAT protein	3.30511E-06	Parp9/Il22/Socs1/Parp14/Socs3/Irf1/Il21/Osm/Ifng/Jak3	10	BP
GO:0002704	negative regulation of leukocyte mediated immunity	3.56749E-06	Tap1/RT1-S3/Tap2/Clec2d/Lgals9/Ceacam1/RT1-A1/Nod2/Jak3	9	BP
GO:0042533	tumor necrosis factor biosynthetic process	3.78353E-06	Tlr2/Lbp/Tlr1/Il10/Tnfrsf8/Bcl3	6	BP
GO:0045785	positive regulation of cell adhesion	3.78552E-06	Cd74/Mip/RT1-S3/Anxa1/Socs1/Lgals9/Ccr5/Ceacam1/Cd80/Cx3cl1/RT1-DMb/Chrd/Runx3/Tnf/Il21/Nlrp3/Ada/Il12rb1/Lilrb4/Tnfsf9/Cd86/Il1b/Ifng/Nfkbiz/Jak3	25	BP
GO:0050731	positive regulation of peptidyl-tyrosine phosphorylation	4.14075E-06	Parp9/Cd74/Ereg/Csf3/Parp14/Ncf1/Cd80/Unc119/Agrn/Epgn/Il21/Osm/Ifng/Cck/Areg/Nod2	16	BP
GO:0050920	regulation of chemo- taxis	4.76227E-06	Cxcr3/Cd74/Sema4a/Ccl4/Tiam1/Lbp/Lgals9/Ccl3/Klrk1/Cx3cl1/Agrn/Edn3/Il1b/Sema7a/Cxcl10/Nod2/Cxcl9	17	BP
GO:0051851	modulation by host of symbiont process	4.79696E-06	Ccl4/Ceacam1/Ccl3/Ifi27/Ncf1/Trem3/Cxcl6/Zc3h12a/Napepld/Cxcl1	10	BP
GO:0002573	myeloid leukocyte differentiation	5.06391E-06	Cd74/Tlr2/Batf/Clec2d/Il17a/Ceacam1/Ccl3/Mafb/Tnf/Sbno2/Hhex/Lilrb4/Tnfsf9/Cd86/Ubd/Ifng/Il17c	17	BP
GO:2000516	positive regulation of CD4-positive, alpha-beta T cell activation	5.08964E-06	Anxa1/Socs1/Lgals9/Ceacam1/Nlrp3/Ifng/Nfkbiz	7	BP
GO:0042088	T-helper 1 type im- mune response	5.17872E-06	Se-ma4a/Slc11a1/Anxa1/Il12rb1/Il1b/Bcl3/Il18bp/Jak3	8	BP
GO:0038061	NIK/NF-kappaB sig- naling	5.40549E-06	Egfr/Tlr2/Lgals9/Litaf/Birc3/Tnf/Eif2ak2/Nlrp3/Zc3h12a/Il1b/Bcl3/Nod2	12	BP
GO:0032729	positive regulation of interferon-gamma production	5.40947E-06	Slc11a1/Lgals9/Klrk1/Tnf/Il21/Il12rb1/Tnfsf9/Il1b/Bcl3/Irf8	10	BP

GO:0032481	positive regulation of type I interferon production	5.49091E-06	Ddx58/Stat1/Tlr2/Irf1/Zc3hav1/Trim15/Irf7/Dhx58/Ifih1	9	BP
GO:0002707	negative regulation of lymphocyte mediated immunity	6.12582E-06	Tap1/RT1-S3/Tap2/Clec2d/Lgals9/Ceacam1/RT1-A1/Nod2	8	BP
GO:0046638	positive regulation of alpha-beta T cell differentiation	6.12582E-06	Anxa1/Socs1/Lgals9/Runx3/Nlrp3/Ada/Ifng/Nfkbiz	8	BP
GO:0050728	negative regulation of inflammatory response	7.11948E-06	Il22/Pla2g5/Socs3/Lgals9/Il10/Cx3cl1/Zfp36/Acod1/Nlrp3/Ada/Zc3h12a/Nod2/Mefv	13	BP
GO:0050798	activated T cell proliferation	7.21533E-06	Itgad/Lgals9/Ido1/Ripk3/Il12rb1/Tnfsf9/Cd86/Jak3	8	BP
GO:0002830	positive regulation of type 2 immune response	7.40995E-06	Cd74/Rsad2/Ido1/Nlrp3/Nod2	5	BP
GO:2001056	positive regulation of cysteine-type endopeptidase activity	7.71627E-06	Stat1/Ifi27l2b/Casp12/Lgals9/Ifi27/Casp4/Pmaip1/Apaf1/Bid/Tnf/Nlrp3/Cck/Mefv	13	BP
GO:0051702	interaction with symbiont	8.58698E-06	Ccl4/Ceacam1/Ccl3/Ifi27/Ncf1/Trem3/Cxcl6/Zc3h12a/Napepld/Cxcl1	10	BP
GO:0031649	heat generation	8.8888E-06	Abat/Ccr5/Il1a/Tnf/Il1rn/Il1b	6	BP
GO:2000108	positive regulation of leukocyte apoptotic process	9.41125E-06	Anxa1/Pdcd1/Lgals9/Il10/Ceacam1/Ido1/Jak3	7	BP
GO:0051048	negative regulation of secretion	9.5281E-06	Abat/Cd74/Anxa1/Lgals9/Il10/Cry1/Ceacam1/Cx3cl1/Rsad2/Tnf/Nlrp3/Ada/Il1rn/Osm/Irs1/Edn3/Zc3h12a/Il1b	18	BP
GO:0032757	positive regulation of interleukin-8 production	9.89044E-06	Ddx58/Tlr2/Lbp/Tlr1/Lgals9/Tnf/Il1b/Nod2	8	BP
GO:0043280	positive regulation of cysteine-type endopeptidase activity involved in apoptotic process	1.00869E-05	Stat1/Ifi27l2b/Casp12/Lgals9/Ifi27/Casp4/Pmaip1/Apaf1/Bid/Tnf/Nlrp3/Cck	12	BP
GO:0001660	fever generation	1.09204E-05	Ccr5/Il1a/Tnf/Il1rn/Il1b	5	BP
GO:0001959	regulation of cytokine-mediated signaling pathway	1.14753E-05	Parp9/Cd74/Socs1/Parp14/Adar/Zbp1/Casp4/Samhd1/Il1rn/Irf7/Irgm	11	BP
GO:0032653	regulation of interleukin-10 production	1.15142E-05	Tlr2/Dll1/Lgals9/Ido1/Il21/Bcl3/Nod2/Jak3	8	BP
GO:0019730	antimicrobial humoral response	1.19252E-05	RT1-S3/Mmp7/Acod1/Cxcl3/Cxcl6/Bcl3/Cxcl10/Nod2/Cxcl1/Cxcl9	10	BP
GO:0043281	regulation of cysteine-type endopeptidase activity involved in apoptotic process	1.21296E-05	Stat1/Ifi27l2b/Casp12/Lgals9/Plaur/Mical1/Ifi27/Casp4/Pmaip1/Apaf1/Bid/Birc3/Tnf/Nlrp3/Mmp9/Cck	16	BP
GO:0006909	phagocytosis	1.22933E-05	Slc11a1/Megf10/Anxa1/Tlr2/Lbp/C3/C2/Clec7a/Ceacam4/Tnf/Ncf4/Il1b/Irf8/Anxa3/Nod	15	BP

			2		
GO:0070227	lymphocyte apoptotic process	1.47256E-05	Cd74/Pdcd1/Lgals9/Il10/Ceacam1/Ido1/Ripk3/Ada/Hsh2d/Jak3	10	BP
GO:0032623	interleukin-2 production	1.54394E-05	Il17f/Slc11a1/Lag3/Anxa1/Ceacam1/Il1a/Zfp36/Il1b/Nod2	9	BP
GO:0042509	regulation of tyrosine phosphorylation of STAT protein	1.54394E-05	Parp9/Il22/Socs1/Parp14/Socs3/Irf1/Il21/Osm/Ifng	9	BP
GO:0031640	killing of cells of other organism	1.54413E-05	Nos2/Ccr5/Ncf1/Trem3/Cxcl6/Ifng/Lyz2/Cxcl1	8	BP
GO:0044364	disruption of cells of other organism	1.54413E-05	Nos2/Ccr5/Ncf1/Trem3/Cxcl6/Ifng/Lyz2/Cxcl1	8	BP
GO:0051709	regulation of killing of cells of other organism	1.56063E-05	Nos2/Ccr5/Cxcl6/Ifng/Cxcl1	5	BP
GO:0031663	lipopolysaccharide-mediated signaling pathway	1.77927E-05	Stat1/Lbp/Prdm1/Ccl3/Cx3cl1/Tnf/Acod1/Il1b	8	BP
GO:0032613	interleukin-10 production	1.77927E-05	Tlr2/Dll1/Lgals9/Ido1/Il21/Bcl3/Nod2/Jak3	8	BP
GO:0000302	response to reactive oxygen species	1.81315E-05	Scgb1a1/Egfr/Stat1/Anxa1/Nox1/Il10/Il1a/Hk3/Stk25/Ncf1/Trpm2/Tnf/Mapk13/Ada/Mmp9/Il1b/Plk3/Il18bp/Areg	19	BP
GO:2000379	positive regulation of reactive oxygen species metabolic process	1.81468E-05	Egfr/Tlr2/Nox1/Klrk1/Tnf/Ripk3/Acod1/Zc3h12a/Il1b/Ifng/Cxcl1	11	BP
GO:0002230	positive regulation of defense response to virus by host	1.85169E-05	Parp9/Ddx58/Stat1/Dtx3l/Il12rb1/Zc3h12a	6	BP
GO:0046006	regulation of activated T cell proliferation	1.95291E-05	Lgals9/Ido1/Ripk3/Il12rb1/Tnfsf9/Cd86/Jak3	7	BP
GO:0046942	carboxylic acid transport	1.96371E-05	Abat/Slc11a1/Grm4/Mip/Pla2g5/Anxa1/Slc16a3/Nos2/Slco2a1/Ceacam1/Il1a/Tnf/Mfsd2a/Slc46a1/Il1rn/Il1b/Slc25a22/Pla2g2a/Cck/Slc7a9	20	BP
GO:0032479	regulation of type I interferon production	1.99808E-05	Ddx58/Stat1/Tlr2/Irf1/Zc3hav1/Trim15/Acod1/Irf7/Dhx58/Ifih1	10	BP
GO:0015849	organic acid transport	2.05386E-05	Abat/Slc11a1/Grm4/Mip/Pla2g5/Anxa1/Slc16a3/Nos2/Slco2a1/Ceacam1/Il1a/Tnf/Mfsd2a/Slc46a1/Il1rn/Il1b/Slc25a22/Pla2g2a/Cck/Slc7a9	20	BP
GO:0071360	cellular response to exogenous dsRNA	2.17224E-05	Ddx58/Ciita/Irf1/Zc3hav1/Ifih1	5	BP
GO:0007259	receptor signaling pathway via JAK-STAT	2.21007E-05	Parp9/Il22/Stat1/Socs1/Parp14/Socs3/Irf1/Il21/Osm/Cxcl6/Bcl3/Ifng/Jak3	13	BP
GO:0045765	regulation of angiogenesis	2.29522E-05	Cxcr3/Sema4a/Il17f/Stat1/Anxa1/Ereg/C3/Dll1/Hk2/Il10/Ceacam1/Il1a/Cx3cl1/Hhex/Mmp9/Zc3h12a/Il1b/Cxcl10/Anxa3	19	BP
GO:0043372	positive regulation of CD4-positive, alpha-beta T cell differentia-	2.31451E-05	Anxa1/Socs1/Lgals9/Nlrp3/Ifng/Nfkbiz	6	BP

	tion				
GO:0045671	negative regulation of osteoclast differentiation	2.31451E-05	Clec2d/Ceacam1/Ccl3/Mafb/Lilrb4/Ifng	6	BP
GO:0034121	regulation of toll-like receptor signaling pathway	2.34047E-05	Tlr2/Lbp/Tlr1/Rsad2/Irf1/Acod1/Irf7/Nod2	8	BP
GO:2000377	regulation of reactive oxygen species metabolic process	2.42166E-05	Egfr/Bco2/Tlr2/Nox1/Hk2/Il10/Klrk1/Birc3/Tnf/Ripk3/Acod1/Zc3h12a/Il1b/Ifng/Cxcl1	15	BP
GO:0032606	type I interferon production	2.43164E-05	Ddx58/Stat1/Tlr2/Irf1/Zc3hav1/Trim15/Acod1/Irf7/Dhx58/Ifih1	10	BP
GO:0045860	positive regulation of protein kinase activity	2.53158E-05	Cd74/Slc11a1/Grm4/Egfr/Socs1/Ereg/Tiam1/Marcks/Bmp2/Ceacam1/Stk25/Ncf1/Unc119/Agrn/Epgn/Tnf/Ripk3/Eif2ak2/Il1rn/Edn3/Irgm/Il1b/Ifng/Nod2/Cdk5r1	25	BP
GO:0034612	response to tumor necrosis factor	2.58029E-05	Stat1/Ccl4/Nos2/Ccl3/Casp4/Cx3cl1/Birc3/Irf1/Zfp36/Tnf/Acod1/Cxcl16/Gbp2/Mmp9/Zc3h12a/Ubd/Il18bp	17	BP
GO:0042093	T-helper cell differentiation	2.67244E-05	Sema4a/Anxa1/Batf/Nlrp3/Zc3h12a/Bcl3/Nfkbiz/Jak3	8	BP
GO:0001961	positive regulation of cytokine-mediated signaling pathway	2.72407E-05	Parp9/Cd74/Parp14/Zbp1/Casp4/Irf7/Irgm	7	BP
GO:0070232	regulation of T cell apoptotic process	2.72407E-05	Pdcd1/Lgals9/Ceacam1/Ido1/Ripk3/Ada/Jak3	7	BP
GO:0002828	regulation of type 2 immune response	2.86608E-05	Cd74/Anxa1/Rsad2/Ido1/Nlrp3/Nod2	6	BP
GO:0036037	CD8-positive, alpha-beta T cell activation	2.86608E-05	RT1-S3/Socs1/Ceacam1/Runx3/Irf1/Ifng	6	BP
GO:0097696	receptor signaling pathway via STAT	2.9163E-05	Parp9/Il22/Stat1/Socs1/Parp14/Socs3/Irf1/Il21/Osm/Cxcl6/Bcl3/Ifng/Jak3	13	BP
GO:1901342	regulation of vasculature development	2.91979E-05	Cxcr3/Sema4a/Il17f/Stat1/Anxa1/Ereg/C3/Dll1/Hk2/Il10/Ceacam1/Il1a/Cx3cl1/Hhex/Mmp9/Zc3h12a/Il1b/Ifng/Cxcl10/Anxa3	20	BP
GO:0032695	negative regulation of interleukin-12 production	2.95516E-05	Tlr2/Il10/Nod2/Mefv/Jak3	5	BP
GO:1900017	positive regulation of cytokine production involved in inflammatory response	2.95516E-05	Il17f/Il17a/Gbp5/Il17c/Nod2	5	BP
GO:2001185	regulation of CD8-positive, alpha-beta T cell activation	2.95516E-05	RT1-S3/Socs1/Ceacam1/Runx3/Irf1	5	BP
GO:1903531	negative regulation of secretion by cell	3.00725E-05	Abat/Anxa1/Lgals9/Il10/Cry1/Ceacam1/Cx3cl1/Rsad2/Tnf/Nlrp3/Il1rn/Osm/Irs1/Edn3/Zc3h12a/Il1b	16	BP
GO:0042116	macrophage activation	3.05724E-05	Slc11a1/Pla2g5/Tlr2/Lbp/Tlr1/Lgals9/Il10/Cx3cl1/Sbno2	9	BP

GO:0002688	regulation of leukocyte chemotaxis	3.29896E-05	Cd74/Ccl4/Lbp/Lgals9/Ccl3/Klrk1/Cx3cl1/Edn3/Il1b/Cxcl10/Nod2	11	BP
GO:0010950	positive regulation of endopeptidase activity	3.33816E-05	Stat1/Ifi27l2b/Casp12/Lgals9/Ifi27/Casp4/Pmaip1/Apaf1/Bid/Tnf/Nlrp3/Cck/Mefv	13	BP
GO:0052548	regulation of endopeptidase activity	3.35592E-05	Stat1/Ifi27l2b/C3/Casp12/Lgals9/Plaur/Mical1/Ifi27/Serpina3n/Casp4/Pmaip1/Apaf1/Bid/Birc3/Tnf/Psmb9/Nlrp3/Serpinc1/Psmb8/Mmp9/Cck/LOC299282/Mefv	23	BP
GO:1990774	tumor necrosis factor secretion	3.51852E-05	Ddx58/Tlr2/Lgals9/Cx3cl1/Zc3h12a/Ifih1	6	BP
GO:0015718	monocarboxylic acid transport	3.81235E-05	Abat/Grm4/Mip/Pla2g5/Anxa1/Slc16a3/Nos2/Slco2a1/Ceacam1/Il1a/Mfsd2a/Il1b/Pla2g2a	13	BP
GO:0002579	positive regulation of antigen processing and presentation	3.89156E-05	Cd74/Slc11a1/Tap2/Nod2	4	BP
GO:0032663	regulation of interleukin-2 production	3.91371E-05	Il17f/Lag3/Anxa1/Ceacam1/Il1a/Zfp36/Il1b/Nod2	8	BP
GO:0045581	negative regulation of T cell differentiation	4.33667E-05	Cd74/Anxa1/Socs1/Runx3/Irf1/Zc3h12a/Jak3	7	BP
GO:0046425	regulation of receptor signaling pathway via JAK-STAT	4.43449E-05	Parp9/Il22/Socs1/Parp14/Socs3/Irf1/Il21/Osm/Cxcl6/Bcl3/Ifng/Cdk5r1	12	BP
GO:0006959	humoral immune response	4.55776E-05	RT1-S3/C3/C2/Mmp7/Tnf/Acod1/Cxcl3/Cxcl6/Bcl3/Ifng/Cxcl10/Nod2/Cxcl1/Cxcl9	14	BP
GO:0051403	stress-activated MAPK cascade	4.92022E-05	Egfr/Tiam1/Nox1/Lgals9/Bmp2/Ceacam1/Il1a/Stk25/Ncf1/Zfp36/Tnf/Mapk13/Eif2ak2/Il1rn/Zc3h12a/Il1b/Nod2	17	BP
GO:0002687	positive regulation of leukocyte migration	5.10288E-05	Cd74/Tlr2/Ccl4/Lbp/Lgals9/Il1a/Ccl3/Cx3cl1/Mmp9/Edn3/Il1b/Cxcl10	12	BP
GO:0051818	disruption of cells of other organism involved in symbiotic interaction	5.16229E-05	Ccr5/Ncf1/Trem3/Cxcl6/Cxcl1	5	BP
GO:0051883	killing of cells in other organism involved in symbiotic interaction	5.16229E-05	Ccr5/Ncf1/Trem3/Cxcl6/Cxcl1	5	BP
GO:0032728	positive regulation of interferon-beta production	5.17954E-05	Ddx58/Tlr2/Irf1/Zc3hav1/Irf7/Ifih1	6	BP
GO:1903409	reactive oxygen species biosynthetic process	5.30965E-05	Tlr2/Nos2/Il10/Duox2/Ncf1/Klrk1/Duox1/Tnf/Zc3h12a/Il1b/Ifng	11	BP
GO:0050921	positive regulation of chemotaxis	5.46857E-05	Cxcr3/Cd74/Ccl4/Tiam1/Lbp/Lgals9/Ccl3/Cx3cl1/Edn3/Il1b/Cxcl10/Cxcl9	12	BP
GO:0006801	superoxide metabolic process	5.60309E-05	Egfr/Nos2/Nox1/Ncf1/Tnf/Ncf4/Noxo1/Cxcl1	8	BP
GO:0032490	detection of molecule of bacterial origin	6.01227E-05	Tlr2/Lbp/Tlr1/Nod2	4	BP
GO:0070943	neutrophil-mediated killing of symbiont	6.01227E-05	Ncf1/Trem3/Cxcl6/Cxcl1	4	BP

	cell				
GO:1904892	regulation of receptor signaling pathway via STAT	6.26841E-05	Parp9/Il22/Socs1/Parp14/Socs3/Irf1/Il21/Osm/Cxcl6/Bcl3/Ifng/Cdk5r1	12	BP
GO:0050829	defense response to Gram-negative bacterium	7.57028E-05	Slc11a1/Lbp/Nos2/Mmp7/Trem3/Gsdmd/Cxcl6/Irgm/Lyz2	9	BP
GO:0032874	positive regulation of stress-activated MAPK cascade	7.61885E-05	Tiam1/Nox1/Bmp2/Ceacam1/Il1a/Stk25/Ncf1/Tnf/Eif2ak2/Il1rn/Zc3h12a/Il1b/Nod2	13	BP
GO:0048247	lymphocyte chemotaxis	7.62865E-05	Cxcr3/Ccl4/Ccl3/Klrk1/Cx3cl1/Cxcl16/Cxcl10	7	BP
GO:0051384	response to glucocorticoid	7.90077E-05	Scgb1a1/Il22/Egfr/Anxa1/C3/Nos2/Socs3/Il10/Il17a/Serpina3n/Zfp36/Tnf/Il1rn/Il1b/Acsbg1/Areg/Anxa3/Cxcl1	18	BP
GO:1904018	positive regulation of vasculature development	8.07036E-05	Cxcr3/Anxa1/Ereg/C3/Dll1/Hk2/Il10/Ceacam1/Il1a/Cx3cl1/Mmp9/Zc3h12a/Il1b/Anxa3	14	BP
GO:0070304	positive regulation of stress-activated protein kinase signaling cascade	8.08938E-05	Tiam1/Nox1/Bmp2/Ceacam1/Il1a/Stk25/Ncf1/Tnf/Eif2ak2/Il1rn/Zc3h12a/Il1b/Nod2	13	BP
GO:0039528	cytoplasmic pattern recognition receptor signaling pathway in response to virus	8.46502E-05	Ddx58/Zc3hav1/Trim15/Dhx58/Ifih1	5	BP
GO:0015711	organic anion transport	8.75425E-05	Abat/Slc11a1/Grm4/Mip/Pla2g5/Slc4a5/Anxa1/Slc16a3/Nos2/Slco2a1/Slc4a7/Ceacam1/Il1a/Tnf/Mfsd2a/Slco4a1/Slc46a1/Il1rn/Il1b/Slc25a22/Pla2g2a/Cck/Slc7a9	23	BP
GO:0042532	negative regulation of tyrosine phosphorylation of STAT protein	8.86656E-05	Socs1/Parp14/Socs3/Irf1	4	BP
GO:0071639	positive regulation of monocyte chemotactic protein-1 production	8.86656E-05	Lgals9/Il1a/Il1b/Nod2	4	BP
GO:0046717	acid secretion	8.90943E-05	Abat/Grm4/Mip/Pla2g5/Anxa1/Nos2/Il1a/Il1rn/Il1b/Pla2g2a/Cck	11	BP
GO:0042136	neurotransmitter biosynthetic process	9.0795E-05	Abat/Tlr2/Nos2/Il10/Klrk1/Tnf/Moxd2/Zc3h12a/Il1b/Ifng	10	BP
GO:0010952	positive regulation of peptidase activity	9.10642E-05	Stat1/Ifi27l2b/Casp12/Lgals9/Ifi27/Casp4/Pmaip1/Apaf1/Bid/Tnf/Nlrp3/Cck/Mefv	13	BP
GO:0032945	negative regulation of mononuclear cell proliferation	9.10886E-05	Scgb1a1/Lgals9/Il10/Ceacam1/Prdm1/Cd80/Ido1/Cd86/Zc3h12d	9	BP
GO:0050672	negative regulation of lymphocyte proliferation	9.10886E-05	Scgb1a1/Lgals9/Il10/Ceacam1/Prdm1/Cd80/Ido1/Cd86/Zc3h12d	9	BP
GO:0071356	cellular response to tumor necrosis factor	9.47431E-05	Stat1/Ccl4/Nos2/Ccl3/Casp4/Cx3cl1/Birc3/Irf1/Zfp36/Tnf/Acod1/Gbp2/Mmp9/Zc3h12a/Il18bp	15	BP

GO:0043123	positive regulation of I-kappaB kinase/NF-kappaB signaling	9.56391E-05	Cd74/Litaf/Il1a/Cx3cl1/Zc3hav1/Tnf/Tifa/Il1rn/Il1b/Ubd/Nod2	11	BP
GO:0002437	inflammatory response to antigenic stimulus	9.7471E-05	C3/Il10/Ccr5/Tnf/Il1rn/Serpinc1/Il1b/Nod2	8	BP
GO:0071715	icosanoid transport	9.91194E-05	Pla2g5/Anxa1/Nos2/Slco2a1/Il1a/Il1b/Pla2g2a	7	BP
GO:1901571	fatty acid derivative transport	9.91194E-05	Pla2g5/Anxa1/Nos2/Slco2a1/Il1a/Il1b/Pla2g2a	7	BP
GO:0001773	myeloid dendritic cell activation	0.000103589	Batf/Il10/Klrk1/Tnfsf9/Cd86/Ubd	6	BP
GO:0046426	negative regulation of receptor signaling pathway via JAK-STAT	0.000106279	Socs1/Parp14/Socs3/Irf1/Bcl3	5	BP
GO:0070230	positive regulation of lymphocyte apoptotic process	0.000106279	Pdcd1/Lgals9/Il10/Ceacam1/Ido1	5	BP
GO:0001776	leukocyte homeostasis	0.000106291	Cd74/Anxa1/Lgals9/Ceacam1/Pmaip1/Ripk3/Ada/Cxcl6/Ifng/Jak3	10	BP
GO:0043122	regulation of I-kappaB kinase/NF-kappaB signaling	0.00010839	Cd74/Stat1/Litaf/Il1a/Cx3cl1/Zc3hav1/Tnf/Tifa/Il1rn/Zc3h12a/Il1b/Ubd/Nod2	13	BP
GO:0043488	regulation of mRNA stability	0.000109016	Scgb1a1/Slc11a1/Plekhn1/Igf2bp1/Noct/Zfp36/Apobec1/Zc3h12a/Zc3h12d	9	BP
GO:0052547	regulation of peptidase activity	0.000110564	Stat1/Ifi27l2b/C3/Casp12/Lgals9/Plaur/Mical1/Ifi27/Serpina3n/Casp4/Pmaip1/Apaf1/Bid/Birc3/Tnf/Psmb9/Nlrp3/Serpinc1/Psmb8/Mmp9/Cck/LOC299282/Mefv	23	BP
GO:0046700	heterocycle catabolic process	0.000114259	Scgb1a1/Apobec3/Slc11a1/Plekhn1/Igf2bp1/Hk2/Pde7a/Hk3/Eno3/Noct/Apaf1/Zfp36/Zc3hav1/Ido1/Samhd1/Slfn13/Apobec1/Ada/Zc3h12a/Oas2/Pde9a/Zc3h12d/Dao	23	BP
GO:0045766	positive regulation of angiogenesis	0.000114765	Cxcr3/Anxa1/Ereg/C3/Dll1/Hk2/Il10/Il1a/Cx3cl1/Mmp9/Zc3h12a/Il1b/Anxa3	13	BP
GO:0045670	regulation of osteo-clast differentiation	0.000119951	Clec2d/Il17a/Ceacam1/Ccl3/Mafb/Tnf/Lilrb4/Ifng	8	BP
GO:0002730	regulation of dendritic cell cytokine produc-tion	0.000125918	Ddx58/Tlr2/Nod2/Jak3	4	BP
GO:0044546	NLRP3 inflammasome complex assembly	0.000125918	Eif2ak2/Nlrp3/Gbp5/Mefv	4	BP
GO:0045342	MHC class II biosyn-thetic process	0.000125918	Slc11a1/Il10/Ciita/Ifng	4	BP
GO:0070942	neutrophil mediated cytotoxicity	0.000125918	Ncf1/Trem3/Cxcl6/Cxcl1	4	BP
GO:0051092	positive regulation of NF-kappaB transcrip-tion factor activity	0.000126089	Tlr2/Lgals9/Cx3cl1/Tnf/Ripk3/Tnfrsf8/Trim15/Eif2ak2/Nlrp3/Il1b/Nod2	11	BP
GO:0002690	positive regulation of	0.000129807	Cd74/Ccl4/Lbp/Lgals9/Ccl3/Cx3cl1/Edn3/Il1	9	BP

	leukocyte chemotaxis		b/Cxcl10		
GO:0070664	negative regulation of leukocyte proliferation	0.000129807	Scgb1a1/Lgals9/Il10/Ceacam1/Prdm1/Cd80/Ido1/Cd86/Zc3h12d	9	BP
GO:1904893	negative regulation of receptor signaling pathway via STAT	0.000131907	Socs1/Parp14/Socs3/Irf1/Bcl3	5	BP
GO:0061013	regulation of mRNA catabolic process	0.000133698	Scgb1a1/Slc11a1/Plekhn1/Igf2bp1/Noct/Zfp36/Zc3hav1/Apobec1/Zc3h12a/Zc3h12d	10	BP
GO:0034655	nucleobase-containing compound catabolic process	0.000156888	Scgb1a1/Apobec3/Slc11a1/Plekhn1/Igf2bp1/Hk2/Pde7a/Hk3/Eno3/Noct/Apaf1/Zfp36/Zc3hav1/Samhd1/Slfn13/Apobec1/Ada/Zc3h12a/Oas2/Pde9a/Zc3h12d	21	BP
GO:0045862	positive regulation of proteolysis	0.000157131	Stat1/Ifi27l2b/C3/Casp12/Lgals9/Ifi27/Casp4/Pmaip1/Apaf1/Bid/Tnf/Nlrp3/Rnf19b/Zc3h12a/Il1b/Ifng/Cck/Plk3/Mefv	19	BP
GO:0045620	negative regulation of lymphocyte differentiation	0.000161472	Cd74/Anxa1/Socs1/Runx3/Irf1/Zc3h12a/Jak3	7	BP
GO:0072676	lymphocyte migration	0.00016714	Cxcr3/Ccl4/Lgals9/Ccl3/Klrk1/Cx3cl1/Ripk3/Cxcl16/Cxcl10	9	BP
GO:0019439	aromatic compound catabolic process	0.000167981	Scgb1a1/Apobec3/Slc11a1/Plekhn1/Igf2bp1/Hk2/Pde7a/Hk3/Eno3/Noct/Apaf1/Zfp36/Zc3hav1/Ido1/Samhd1/Slfn13/Apobec1/Ada/Moxd2/Zc3h12a/Oas2/Pde9a/Zc3h12d	23	BP
GO:0002371	dendritic cell cytokine production	0.000173321	Ddx58/Tlr2/Nod2/Jak3	4	BP
GO:0031652	positive regulation of heat generation	0.000173321	Abat/Ccr5/Tnf/Il1b	4	BP
GO:0002526	acute inflammatory response	0.000175432	Lbp/C3/Ccr5/Il1a/Tnf/Nlrp3/Il1rn/Serpinc1/Trem3/Il1b/Cxcl1	11	BP
GO:1901361	organic cyclic compound catabolic process	0.00018609	Scgb1a1/Apobec3/Slc11a1/Plekhn1/Igf2bp1/Hk2/Pde7a/Hk3/Eno3/Noct/Apaf1/Zfp36/Zc3hav1/Ido1/Samhd1/Slfn13/Apobec1/Ada/Moxd2/Zc3h12a/Oas2/Pde9a/Zc3h12d/Dao	24	BP
GO:0002478	antigen processing and presentation of exogenous peptide antigen	0.000197096	Cd74/Tap1/Tap2/B2m/RT1-DMb	5	BP
GO:0032692	negative regulation of interleukin-1 production	0.000197096	Ceacam1/Cx3cl1/Nlrp3/Zc3h12a/Mefv	5	BP
GO:0071359	cellular response to dsRNA	0.000197096	Ddx58/Ciita/Irf1/Zc3hav1/Ifih1	5	BP
GO:0031098	stress-activated protein kinase signaling cascade	0.000200854	Egfr/Tiam1/Nox1/Lgals9/Bmp2/Ceacam1/Il1a/Stk25/Ncf1/Zfp36/Tnf/Mapk13/Eif2ak2/Il1rn/Zc3h12a/Il1b/Nod2	17	BP
GO:0070231	T cell apoptotic process	0.000202822	Pdcd1/Lgals9/Ceacam1/Ido1/Ripk3/Ada/Jak3	7	BP
GO:0070374	positive regulation of ERK1 and ERK2 cascade	0.000204854	Cd74/Pla2g5/Egfr/Tlr2/Ccl4/C3/Lgals9/Bmp2/Il1a/Thpo/Ccl3/Cx3cl1/Il1b/Pla2g2a/Sema7a/Nod2	16	BP
GO:0031960	response to cortico-	0.000208275	Scgb1a1/Il22/Egfr/Anxa1/C3/Nos2/Socs3/Il1	18	BP

	steroid		0/Il17a/Serpina3n/Zfp36/Tnf/Il1rn/Il1b/Acsbg1/Areg/Anxa3/Cxcl1		
GO:0071902	positive regulation of protein serine/threonine kinase activity	0.000226488	Cd74/Grm4/Egfr/Tiam1/Marcks/Bmp2/Ceacam1/Epgn/Tnf/Eif2ak2/Il1rn/Edn3/Irgm/Il1b/Ifng/Nod2/Cdk5r1	17	BP
GO:0050755	chemokine metabolic process	0.000232375	Tnf/Trem3/Il1b/Ifng	4	BP
GO:0051712	positive regulation of killing of cells of other organism	0.000232375	Nos2/Ccr5/Ifng/Cxcl1	4	BP
GO:0060330	regulation of response to interferon-gamma	0.000232375	Parp9/Socs1/Parp14/Irgm	4	BP
GO:0060334	regulation of interferon-gamma-mediated signaling pathway	0.000232375	Parp9/Socs1/Parp14/Irgm	4	BP
GO:0035455	response to interferon-alpha	0.000237682	Adar/Ifit3/Oas1a/Ifit2/Eif2ak2	5	BP
GO:0045742	positive regulation of epidermal growth factor receptor signaling pathway	0.000237682	Ereg/Plaur/Ncf1/Epgn/Mmp9	5	BP
GO:0046639	negative regulation of alpha-beta T cell differentiation	0.000237682	Anxa1/Socs1/Runx3/Zc3h12a/Jak3	5	BP
GO:0043487	regulation of RNA stability	0.000249072	Scgb1a1/Slc11a1/Plekhn1/Igf2bp1/Noct/Zfp36/Apobec1/Zc3h12a/Zc3h12d	9	BP
GO:0046636	negative regulation of alpha-beta T cell activation	0.000250451	Anxa1/Socs1/Lgals9/Runx3/Zc3h12a/Jak3	6	BP
GO:0097028	dendritic cell differentiation	0.000250451	Batf/Lgals9/Trpm2/Tnfsf9/Cd86/Ubd	6	BP
GO:0032720	negative regulation of tumor necrosis factor production	0.00025232	Lbp/Lgals9/Il10/Cx3cl1/Zc3h12a/Bcl3/Nod2	7	BP
GO:0044270	cellular nitrogen compound catabolic process	0.000255885	Scgb1a1/Apobec3/Slc11a1/Plekhn1/Igf2bp1/Hk2/Pde7a/Hk3/Eno3/Noct/Apaf1/Zfp36/Zc3hav1/Ido1/Samhd1/Slfn13/Apobec1/Ada/Zc3h12a/Oas2/Pde9a/Zc3h12d	22	BP
GO:0051091	positive regulation of DNA-binding transcription factor activity	0.000277842	Ddx58/Tlr2/Csf3/Lgals9/Il10/Cx3cl1/Tnf/Ripk3/Tnfrsf8/Trim15/Eif2ak2/Nlrp3/Il1b/Anxa3/Nod2	15	BP
GO:1903556	negative regulation of tumor necrosis factor superfamily cytokine production	0.000280477	Lbp/Lgals9/Il10/Cx3cl1/Zc3h12a/Bcl3/Nod2	7	BP
GO:2000107	negative regulation of leukocyte apoptotic process	0.000280477	Cd74/Ccr5/Ido1/Ada/Hsh2d/Nod2/Jak3	7	BP
GO:0006809	nitric oxide biosynthetic process	0.000280624	Tlr2/Nos2/Il10/Klrk1/Tnf/Zc3h12a/Il1b/Ifng	8	BP

GO:0002825	regulation of T-helper 1 type immune response	0.00028432	Slc11a1/Anxa1/Il12rb1/Il1b/Jak3	5	BP
GO:0045589	regulation of regulatory T cell differentiation	0.00028432	Socs1/Lgals9/Irf1/Lilrb4/Ifng	5	BP
GO:0002444	myeloid leukocyte mediated immunity	0.000290042	Ddx58/C3/Lgals9/Ccl3/Ncf1/Trem3/Cxcl6/Anxa3/Cxcl1	9	BP
GO:0031650	regulation of heat generation	0.000304632	Abat/Ccr5/Tnf/Il1b	4	BP
GO:0034134	toll-like receptor 2 signaling pathway	0.000304632	Tlr2/Tlr1/Acod1/Nod2	4	BP
GO:0043374	CD8-positive, alpha-beta T cell differentiation	0.000304632	Socs1/Runx3/Irf1/Ifng	4	BP
GO:0045064	T-helper 2 cell differentiation	0.000304632	Anxa1/Batf/Nlrp3/Bcl3	4	BP
GO:0045591	positive regulation of regulatory T cell differentiation	0.000304632	Socs1/Lgals9/Lilrb4/Ifng	4	BP
GO:0051873	killing by host of symbiont cells	0.000304632	Ncf1/Trem3/Cxcl6/Cxcl1	4	BP
GO:0070234	positive regulation of T cell apoptotic process	0.000304632	Pdcd1/Lgals9/Ceacam1/Ido1	4	BP
GO:0045639	positive regulation of myeloid cell differentiation	0.000336438	Cd74/Stat1/Csf3/Il17a/Isg15/Thpo/Ccl3/Tnf/Ifng	9	BP
GO:0002544	chronic inflammatory response	0.000337582	Il10/Ido1/Tnf/Il1rn/Il1b	5	BP
GO:1901186	positive regulation of ERBB signaling pathway	0.000337582	Ereg/Plaur/Ncf1/Epgn/Mmp9	5	BP
GO:0050766	positive regulation of phagocytosis	0.000380396	Slc11a1/Lbp/C3/C2/Tnf/Il1b/Nod2	7	BP
GO:0002577	regulation of antigen processing and presentation	0.000391681	Cd74/Slc11a1/Tap2/Nod2	4	BP
GO:0061081	positive regulation of myeloid leukocyte cytokine production involved in immune response	0.000391681	Cd74/Ddx58/Tlr2/Sema7a	4	BP
GO:0046209	nitric oxide metabolic process	0.000394043	Tlr2/Nos2/Il10/Klrk1/Tnf/Zc3h12a/Il1b/Ifng	8	BP
GO:0010876	lipid localization	0.000394636	Apol3/Mip/Pla2g5/Anxa1/Lbp/Apol9a/C3/Nos2/Slco2a1/Cry1/Ceacam1/Il1a/Abca13/RGD1309808/Tnf/Mfsd2a/Zc3h12a/Il1b/Pla2g2a	19	BP
GO:0032660	regulation of interleukin-17 production	0.000398062	Tlr2/Il21/Osm/Ifng/Nod2	5	BP

GO:0016064	immunoglobulin mediated immune response	0.000401451	Cd74/Batf/C3/C2/Pou2f2/Tnf/Irf7/Bcl3/Ifng/Nod2	10	BP
GO:0048872	homeostasis of number of cells	0.000402014	Cd74/Stat1/Anxa1/Lgals9/Ceacam1/Isg15/Adar/Pmaip1/B2m/Mafb/Zfp36/Ripk3/Ada/Cxcl6/Ifng/Napepld/Jak3	17	BP
GO:0030316	osteoclast differentiation	0.00041739	Clec2d/Il17a/Ceacam1/Ccl3/Mafb/Tnf/Sbno2/Lilrb4/Ifng	9	BP
GO:0050710	negative regulation of cytokine secretion	0.000419392	Anxa1/Lgals9/Il10/Cx3cl1/Tnf/Nlrp3/Zc3h12a	7	BP
GO:0002792	negative regulation of peptide secretion	0.000431799	Cd74/Anxa1/Lgals9/Il10/Cx3cl1/Rsad2/Tnf/Nlrp3/Irs1/Zc3h12a/Il1b	11	BP
GO:0019724	B cell mediated immunity	0.00045466	Cd74/Batf/C3/C2/Pou2f2/Tnf/Irf7/Bcl3/Ifng/Nod2	10	BP
GO:0061844	antimicrobial humoral immune response mediated by antimicrobial peptide	0.000461516	Mmp7/Cxcl3/Cxcl6/Cxcl10/Nod2/Cxcl1/Cxcl9	7	BP
GO:0019884	antigen processing and presentation of exogenous antigen	0.000466372	Cd74/Tap1/Tap2/B2m/RT1-DMb	5	BP
GO:0044003	modulation by symbiont of host process	0.000466372	Tlr2/Ccr5/Ceacam1/Mmp9/Zc3h12a	5	BP
GO:0045066	regulatory T cell differentiation	0.000466372	Socs1/Lgals9/Irf1/Lilrb4/Ifng	5	BP
GO:2000515	negative regulation of CD4-positive, alpha-beta T cell activation	0.000466372	Anxa1/Lgals9/Runx3/Zc3h12a/Jak3	5	BP
GO:0032309	icosanoid secretion	0.000468655	Pla2g5/Anxa1/Nos2/Il1a/Il1b/Pla2g2a	6	BP
GO:0010623	programmed cell death involved in cell development	0.000495146	Slc4a7/Il1a/Casp4/Il1b	4	BP
GO:0039529	RIG-I signaling pathway	0.000495146	Ddx58/Zc3hav1/Trim15/Dhx58	4	BP
GO:0061158	3'-UTR-mediated mRNA destabilization	0.000495146	Plekhn1/Zfp36/Zc3h12a/Zc3h12d	4	BP
GO:0070372	regulation of ERK1 and ERK2 cascade	0.000511226	Cd74/Pla2g5/Egfr/Tlr2/Ccl4/Tiam1/C3/Lgals9/Bmp2/Ceacam1/Il1a/Thpo/Ccl3/Cx3cl1/Il1b/Pla2g2a/Sema7a/Nod2	18	BP
GO:0002762	negative regulation of myeloid leukocyte differentiation	0.000526092	Clec2d/Ceacam1/Ccl3/Mafb/Lilrb4/Ifng	6	BP
GO:0038066	p38MAPK cascade	0.000526092	Lgals9/Bmp2/Ncf1/Zfp36/Zc3h12a/Il1b	6	BP
GO:0032872	regulation of stress-activated MAPK cascade	0.000532106	Egfr/Tiam1/Nox1/Bmp2/Ceacam1/Il1a/Stk25/Ncf1/Tnf/Eif2ak2/Il1rn/Zc3h12a/Il1b/Nod2	14	BP
GO:0045638	negative regulation of myeloid cell differentiation	0.000542209	Dll1/Clec2d/Ceacam1/Ccl3/Mafb/Zfp36/Lilrb4/Ifng	8	BP
GO:2001057	reactive nitrogen spe-	0.000542209	Tlr2/Nos2/Il10/Klrk1/Tnf/Zc3h12a/Il1b/Ifng	8	BP

	cies metabolic process				
GO:0051701	interaction with host	0.000578734	Cd74/Tmprss2/Tlr2/Lgals9/Ccr5/Ceacam1/Nectin4/Trim15/Mmp9/Zc3h12a	10	BP
GO:0070302	regulation of stress-activated protein kinase signaling cascade	0.000578914	Egfr/Tiam1/Nox1/Bmp2/Ceacam1/Il1a/Stk25/Ncf1/Tnf/Eif2ak2/Il1rn/Zc3h12a/Il1b/Nod2	14	BP
GO:0018958	phenol-containing compound metabolic process	0.000587567	Abat/Ly6e/Duox2/Duoxa2/Duox1/Slco4a1/Moxd2/Nr4a2/Dao	9	BP
GO:0031331	positive regulation of cellular catabolic process	0.000604379	Tlr2/Dtx3l/Plekhn1/Hk2/Bid/Zfp36/Zc3hav1/Tnf/Rnf19b/Irs1/Zc3h12a/Irgm/Il1b/Ifng/Zc3h12d/Plk3/Nod2/Mefv	18	BP
GO:0002643	regulation of tolerance induction	0.000616677	Pdcd1/RT1-A1/Ido1/Cd86	4	BP
GO:0015732	prostaglandin transport	0.000616677	Nos2/Slco2a1/Il1a/Il1b	4	BP
GO:0051956	negative regulation of amino acid transport	0.000616677	Abat/Tnf/Il1rn/Il1b	4	BP
GO:0071605	monocyte chemotactic protein-1 production	0.000616677	Lgals9/Il1a/Il1b/Nod2	4	BP
GO:0071637	regulation of monocyte chemotactic protein-1 production	0.000616677	Lgals9/Il1a/Il1b/Nod2	4	BP
GO:0050852	T cell receptor signaling pathway	0.000627614	Ceacam1/Btnl7/Ada/Themis2/Lilrb4/Btnl5/Zc3h12a/Ifng/Nfkbiz	9	BP
GO:0032727	positive regulation of interferon-alpha production	0.000629033	Ddx58/Stat1/Zc3hav1/Irf7/Ifih1	5	BP
GO:0032733	positive regulation of interleukin-10 production	0.000629033	Tlr2/Lgals9/Il21/Bcl3/Nod2	5	BP
GO:0050764	regulation of phagocytosis	0.000631602	Slc11a1/Tlr2/Lbp/C3/C2/Tnf/Il1b/Nod2	8	BP
GO:0006953	acute-phase response	0.000657277	Lbp/Ccr5/Il1a/Tnf/Il1rn/Il1b	6	BP
GO:0051353	positive regulation of oxidoreductase activity	0.000657277	Lgals9/Tnf/Ripk3/Il1b/Ifng/Nod2	6	BP
GO:0045428	regulation of nitric oxide biosynthetic process	0.000664952	Tlr2/Il10/Klrk1/Tnf/Zc3h12a/Il1b/Ifng	7	BP
GO:0015908	fatty acid transport	0.000680557	Pla2g5/Anxa1/Nos2/Slco2a1/Il1a/Mfsd2a/Il1b/Pla2g2a	8	BP
GO:0045622	regulation of T-helper cell differentiation	0.000724703	Anxa1/Nlrp3/Zc3h12a/Nfkbiz/Jak3	5	BP
GO:0032648	regulation of interferon-beta production	0.000731698	Ddx58/Tlr2/Irf1/Zc3hav1/Irf7/Ifih1	6	BP
GO:0002726	positive regulation of T cell cytokine production	0.000757941	B2m/Rsad2/Nlrp3/Il1b	4	BP

GO:0002922	positive regulation of humoral immune response	0.000757941	C3/Tnf/Acod1/Nod2	4	BP
GO:0032656	regulation of interleukin-13 production	0.000757941	Scgb1a1/RT1-S3/Lgals9/Nlrp3	4	BP
GO:0051770	positive regulation of nitric-oxide synthase biosynthetic process	0.000757941	Stat1/Tlr2/Nod2/Jak3	4	BP
GO:0060338	regulation of type I interferon-mediated signaling pathway	0.000757941	Adar/Zbp1/Samhd1/Irf7	4	BP
GO:0042130	negative regulation of T cell proliferation	0.000790346	Scgb1a1/Lgals9/Ceacam1/Cd80/Ido1/Cd86/Zc3h12d	7	BP
GO:0032608	interferon-beta production	0.000812468	Ddx58/Tlr2/Irf1/Zc3hav1/Irf7/Ifih1	6	BP
GO:0042060	wound healing	0.000893921	Abat/Slc11a1/Egfr/Anxa1/Gp5/C3/Fcgr3a/Nos2/F10/Plek/Plaur/Ceacam1/Il1a/Eno3/LOC683313/Cx3cl1/Trim72/Serpinc1/Il1b/Grhl3	20	BP
GO:0070371	ERK1 and ERK2 cascade	0.000917787	Cd74/Pla2g5/Egfr/Tlr2/Ccl4/Tiam1/C3/Lgals9/Bmp2/Ceacam1/Il1a/Thpo/Ccl3/Cx3cl1/Il1b/Pla2g2a/Sema7a/Nod2	18	BP
GO:0002827	positive regulation of T-helper 1 type immune response	0.000920616	Slc11a1/Anxa1/Il12rb1/Il1b	4	BP
GO:0010818	T cell chemotaxis	0.000920616	Cxcr3/Ccl3/Cxcl16/Cxcl10	4	BP
GO:0032616	interleukin-13 production	0.000920616	Scgb1a1/RT1-S3/Lgals9/Nlrp3	4	BP
GO:0002446	neutrophil mediated immunity	0.000948136	Ncf1/Trem3/Cxcl6/Anxa3/Cxcl1	5	BP
GO:0032620	interleukin-17 production	0.000948136	Tlr2/Il21/Osm/Ifng/Nod2	5	BP
GO:0032689	negative regulation of interferon-gamma production	0.000948136	Scgb1a1/Lgals9/Il10/Zc3h12a/Nod2	5	BP
GO:0050709	negative regulation of protein secretion	0.000962309	Anxa1/Lgals9/Il10/Cx3cl1/Rsad2/Tnf/Nlrp3/Irs1/Zc3h12a/Il1b	10	BP
GO:0007173	epidermal growth factor receptor signaling pathway	0.000973263	Egfr/Ereg/Plaur/Ceacam1/Ncf1/Epgn/Mmp9/Areg	8	BP
GO:0010332	response to gamma radiation	0.001012695	Socs3/Il1a/Apobec1/Cxcl6/Il1b/Cxcl10/Cxcl1	7	BP
GO:0002468	dendritic cell antigen processing and presentation	0.001038761	Cd74/Slc11a1/Nod2	3	BP
GO:0010041	response to iron(III) ion	0.001038761	Casp12/B2m/Mmp9	3	BP
GO:0031622	positive regulation of fever generation	0.001038761	Ccr5/Tnf/Il1b	3	BP
GO:0045741	positive regulation of epidermal growth	0.001038761	Ereg/Ncf1/Epgn	3	BP

	factor-activated receptor activity				
GO:0046598	positive regulation of viral entry into host cell	0.001038761	Cd74/Tmprss2/Lgals9	3	BP
GO:2001187	positive regulation of CD8-positive, alpha-beta T cell activation	0.001038761	RT1-S3/Ceacam1/Runx3	3	BP
GO:0042743	hydrogen peroxide metabolic process	0.001096502	Egfr/Nox1/Duox2/Duoxa2/Ncf1/Duox1	6	BP
GO:0051043	regulation of membrane protein ectodomain proteolysis	0.001106386	Il10/Tnf/Il1b/Ifng	4	BP
GO:0009408	response to heat	0.00112898	Tfec/Socs3/Il1a/Trpm2/Irf1/Osm/Mmp9/Il1b/Cxcl10/Cxcl1	10	BP
GO:0010508	positive regulation of autophagy	0.001164991	Tlr2/Hk2/Bid/Zc3h12a/Irgm/Ifng/Plk3/Nod2/Mefv	9	BP
GO:0042113	B cell activation	0.00118148	Cd74/Batf/Dll1/Il10/Prdm1/Pou2f2/Hhex/Il21/Ada/Cd86/Bcl3/Ifng/Nod2/Jak3	14	BP
GO:0032647	regulation of interferon-alpha production	0.001219077	Ddx58/Stat1/Zc3hav1/Irf7/Ifih1	5	BP
GO:0046640	regulation of alpha-beta T cell proliferation	0.001219077	RT1-S3/Lgals9/Ceacam1/Cd80/Irf1	5	BP
GO:0048255	mRNA stabilization	0.001219077	Slc11a1/Igf2bp1/Noct/Zfp36/Apobec1	5	BP
GO:0002260	lymphocyte homeostasis	0.001281497	Cd74/Lgals9/Ceacam1/Pmaip1/Ripk3/Ada/Jak3	7	BP
GO:1901224	positive regulation of NIK/NF-kappaB signaling	0.001281497	Egfr/Tlr2/Lgals9/Tnf/Eif2ak2/Il1b/Nod2	7	BP
GO:0002902	regulation of B cell apoptotic process	0.001316935	Cd74/Il10/Ada/Hsh2d	4	BP
GO:0034123	positive regulation of toll-like receptor signaling pathway	0.001316935	Tlr2/Lbp/Tlr1/Rsad2	4	BP
GO:0043011	myeloid dendritic cell differentiation	0.001316935	Batf/Tnfsf9/Cd86/Ubd	4	BP
GO:0043371	negative regulation of CD4-positive, alpha-beta T cell differentiation	0.001316935	Anxa1/Runx3/Zc3h12a/Jak3	4	BP
GO:0098581	detection of external biotic stimulus	0.001316935	Tlr2/Lbp/Tlr1/Nod2	4	BP
GO:1901623	regulation of lymphocyte chemotaxis	0.001316935	Ccl4/Ccl3/Klrk1/Cxcl10	4	BP
GO:0043406	positive regulation of MAP kinase activity	0.001344617	Cd74/Grm4/Egfr/Tiam1/Bmp2/Ceacam1/Epgn/Tnf/Eif2ak2/Il1rn/Edn3/Il1b/Nod2	13	BP
GO:0032607	interferon-alpha production	0.001374177	Ddx58/Stat1/Zc3hav1/Irf7/Ifih1	5	BP

GO:0002676	regulation of chronic inflammatory response	0.00140575	Il10/Ido1/Tnf	3	BP
GO:0010193	response to ozone	0.00140575	Scgb1a1/Il1a/Il1b	3	BP
GO:0031620	regulation of fever generation	0.00140575	Ccr5/Tnf/Il1b	3	BP
GO:0034135	regulation of toll-like receptor 2 signaling pathway	0.00140575	Tlr1/Acod1/Nod2	3	BP
GO:0042368	vitamin D biosynthetic process	0.00140575	Tnf/Il1b/Ifng	3	BP
GO:0002761	regulation of myeloid leukocyte differentia-tion	0.001463933	Cd74/Clec2d/Il17a/Ceacam1/Ccl3/Mafb/Tnf/Lilrb4/Ifng	9	BP
GO:0002369	T cell cytokine pro-duction	0.001543351	Slc11a1/B2m/Rsad2/Nlrp3/Il1b	5	BP
GO:0044764	multi-organism cellu-lar process	0.001543351	Ddx58/Zc3hav1/Trim15/Dhx58/Ifih1	5	BP
GO:0046627	negative regulation of insulin receptor signal-ing pathway	0.001543351	Socs1/Socs3/Trim72/Irs1/Il1b	5	BP
GO:0070229	negative regulation of lymphocyte apoptotic process	0.001543351	Cd74/Ido1/Ada/Hsh2d/Jak3	5	BP
GO:0002335	mature B cell differen-tiation	0.00155394	Dll1/Pou2f2/Ada/Bcl3	4	BP
GO:0034114	regulation of hetero-typic cell-cell adhe-sion	0.00155394	Il10/Tnf/Il1rn/Il1b	4	BP
GO:0046641	positive regulation of alpha-beta T cell pro-liferation	0.00155394	RT1-S3/Lgals9/Ceacam1/Cd80	4	BP
GO:0002768	immune response-regulating cell surface receptor signaling pathway	0.00157228	Tlr2/Fcgr3a/Ceacam1/Klrk1/Btnl7/Ada/Themis2/Lilrb4/Btnl5/Zc3h12a/Ifng/Nfkbiz	12	BP
GO:0045840	positive regulation of mitotic nuclear divi-sion	0.001586792	Ereg/Il1a/Epgn/Tnf/Edn3/Il1b	6	BP
GO:0072678	T cell migration	0.001586792	Cxcr3/Lgals9/Ccl3/Ripk3/Cxcl16/Cxcl10	6	BP
GO:0042133	neurotransmitter met-abolic process	0.001691505	Abat/Tlr2/Nos2/Il10/Klrk1/Tnf/Moxd2/Zc3h12a/Il1b/Ifng	10	BP
GO:0046633	alpha-beta T cell pro-liferation	0.001727349	RT1-S3/Lgals9/Ceacam1/Cd80/Irf1	5	BP
GO:1902622	regulation of neutro-phil migration	0.001727349	Cd74/Lbp/Il1a/Il1b/Nod2	5	BP
GO:0009595	detection of biotic stimulus	0.001819069	Tlr2/Lbp/Tlr1/Nod2	4	BP
GO:0010575	positive regulation of vascular endothelial growth factor produc-	0.001819069	C3/Nox1/Il1a/Il1b	4	BP

	tion				
GO:0042403	thyroid hormone metabolic process	0.001819069	Duox2/Duoxa2/Duox1/Slco4a1	4	BP
GO:0048305	immunoglobulin secretion	0.001819069	RT1-S3/Ceacam1/Pou2f2/Tnf	4	BP
GO:0051767	nitric-oxide synthase biosynthetic process	0.001819069	Stat1/Tlr2/Nod2/Jak3	4	BP
GO:0051769	regulation of nitric-oxide synthase biosynthetic process	0.001819069	Stat1/Tlr2/Nod2/Jak3	4	BP
GO:0072539	T-helper 17 cell differentiation	0.001819069	Batf/Nlrp3/Zc3h12a/Nfkbiz	4	BP
GO:0016338	calcium-independent cell-cell adhesion via plasma membrane cell-adhesion molecules	0.001844778	Bmp2/Ceacam1/Cx3cl1	3	BP
GO:0045346	regulation of MHC class II biosynthetic process	0.001844778	Il10/Ciita/Ifng	3	BP
GO:1900225	regulation of NLRP3 inflammasome complex assembly	0.001844778	Eif2ak2/Gbp5/Mefv	3	BP
GO:0051099	positive regulation of binding	0.001849937	Parp9/Tiam1/Stpg1/Csf3/Dtx3l/Lgals9/Bmp2/Plaur/B2m/Agrn/Mmp9/Ifng	12	BP
GO:0001525	angiogenesis	0.001878255	Cxcr3/Sema4a/Il17f/Stat1/Anxa1/Ereg/C3/Dll1/Nox1/Hk2/Il10/Ceacam1/Il1a/Cx3cl1/Epgn/Hhex/Mmp9/Zc3h12a/Il1b/Cxcl10/Anxa3	21	BP
GO:2000401	regulation of lymphocyte migration	0.001886938	Ccl4/Lgals9/Ccl3/Klrk1/Ripk3/Cxcl10	6	BP
GO:2001235	positive regulation of apoptotic signaling pathway	0.001893094	Nox1/Lgals9/Plaur/Pmaip1/Apaf1/Bid/Runx3/Ripk3/Osm/Mmp9/Jak3	11	BP
GO:1900077	negative regulation of cellular response to insulin stimulus	0.00192693	Socs1/Socs3/Trim72/Irs1/Il1b	5	BP
GO:0038127	ERBB signaling pathway	0.00197268	Egfr/Ereg/Plaur/Ceacam1/Ncf1/Epgn/Mmp9/Areg	8	BP
GO:0019932	second-messenger-mediated signaling	0.002011031	Cxcr3/Gnal/Egfr/Ccl4/Nos2/Ptger1/Ccr5/Ceacam1/Pde7a/Ccl3/Trpm2/Tnni3/Tnf/Ada/Edn3/Irgm/Pde9a/Ccrl2/Cxcl10/Cxcl9	20	BP
GO:0051897	positive regulation of protein kinase B signaling	0.002093318	Egfr/Csf3/F10/Thpo/Ccl3/Cx3cl1/Tnf/Osm	8	BP
GO:0006925	inflammatory cell apoptotic process	0.002113974	Anxa1/Ccr5/Ifng/Nod2	4	BP
GO:0019048	modulation by virus of host process	0.002113974	Ccr5/Ceacam1/Mmp9/Zc3h12a	4	BP
GO:0032703	negative regulation of interleukin-2 production	0.002113974	Lag3/Ceacam1/Zfp36/Nod2	4	BP

GO:0032891	negative regulation of organic acid transport	0.002113974	Abat/Tnf/Il1rn/Il1b	4	BP
GO:0045730	respiratory burst	0.002113974	Slc11a1/Lbp/Ncf1/Ncf4	4	BP
GO:0051341	regulation of oxidore-ductase activity	0.002125871	Egfr/Lgals9/Tnf/Ripk3/Il1b/Ifng/Nod2	7	BP
GO:0043489	RNA stabilization	0.002142857	Slc11a1/Igf2bp1/Noct/Zfp36/Apobec1	5	BP
GO:0060147	regulation of posttran-scriptional gene si-lencing	0.002142857	Egfr/Adar/Zfp36/Zc3h12a/Mael	5	BP
GO:0060966	regulation of gene silencing by RNA	0.002142857	Egfr/Adar/Zfp36/Zc3h12a/Mael	5	BP
GO:0042058	regulation of epider-mal growth factor receptor signaling pathway	0.002228196	Ereg/Plaur/Ceacam1/Ncf1/Epgn/Mmp9	6	BP
GO:0006869	lipid transport	0.002258606	Apol3/Mip/Pla2g5/Anxa1/Lbp/Apol9a/Nos2/Slco2a1/Cry1/Ceacam1/Il1a/Abca13/RGD1309808/Mfsd2a/Il1b/Pla2g2a	16	BP
GO:0043491	protein kinase B sig-naling	0.002341362	Egfr/Csf3/F10/Thpo/Ccl3/Cx3cl1/Tnf/Osm/Irs1/Il1b/Plk3	11	BP
GO:0032092	positive regulation of protein binding	0.002351966	Tiam1/Stpg1/Csf3/Dtx3l/Bmp2/B2m/Agrn/Mmp9	8	BP
GO:0010935	regulation of macro-phage cytokine pro-duction	0.002360436	Cd74/Tlr2/Sema7a	3	BP
GO:0016553	base conversion or substitution editing	0.002360436	Apobec3/Adar/Apobec1	3	BP
GO:0042033	chemokine biosynthet-ic process	0.002360436	Tnf/Il1b/Ifng	3	BP
GO:0042362	fat-soluble vitamin biosynthetic process	0.002360436	Tnf/Il1b/Ifng	3	BP
GO:0010893	positive regulation of steroid biosynthetic process	0.002440285	Il1a/Tnf/Il1b/Ifng	4	BP
GO:0042104	positive regulation of activated T cell prolif-eration	0.002440285	Il12rb1/Tnfsf9/Cd86/Jak3	4	BP
GO:0061157	mRNA destabilization	0.002440285	Plekhn1/Zfp36/Zc3h12a/Zc3h12d	4	BP
GO:1900745	positive regulation of p38MAPK cascade	0.002440285	Bmp2/Ncf1/Zc3h12a/Il1b	4	BP
GO:0002429	immune response-activating cell surface receptor signaling pathway	0.002440578	Tlr2/Ceacam1/Klrk1/Btnl7/Ada/Themis2/Lilrb4/Btnl5/Zc3h12a/Ifng/Nfkbiz	11	BP
GO:0051453	regulation of intracel-lular pH	0.002614004	Slc9a5/Slc11a1/Slc4a5/Nox1/Slc4a7/Tcirg1	6	BP
GO:0061014	positive regulation of mRNA catabolic pro-cess	0.002626825	Plekhn1/Zfp36/Zc3hav1/Zc3h12a/Zc3h12d	5	BP

GO:0061082	myeloid leukocyte cytokine production	0.002799613	Cd74/Ddx58/Tlr2/Sema7a	4	BP
GO:0072538	T-helper 17 type immune response	0.002799613	Batf/Nlrp3/Zc3h12a/Nfkbiz	4	BP
GO:0043029	T cell homeostasis	0.002896411	Lgals9/Ceacam1/Pmaip1/Ripk3/Jak3	5	BP
GO:0043030	regulation of macrophage activation	0.002896411	Pla2g5/Lbp/Lgals9/Il10/Cx3cl1	5	BP
GO:0045429	positive regulation of nitric oxide biosynthetic process	0.002896411	Tlr2/Klrk1/Tnf/Il1b/Ifng	5	BP
GO:1902373	negative regulation of mRNA catabolic process	0.002896411	Slc11a1/Igf2bp1/Noct/Zfp36/Apobec1	5	BP
GO:0002517	T cell tolerance induction	0.002956922	RT1-A1/Ido1/Cd86	3	BP
GO:0019054	modulation by virus of host cellular process	0.002956922	Ccr5/Ceacam1/Zc3h12a	3	BP
GO:0032494	response to peptidoglycan	0.002956922	Tlr2/Nlrp3/Nod2	3	BP
GO:0032604	granulocyte macrophage colony-stimulating factor production	0.002956922	Il17f/Ddx58/Il1b	3	BP
GO:0032645	regulation of granulocyte macrophage colony-stimulating factor production	0.002956922	Il17f/Ddx58/Il1b	3	BP
GO:0009914	hormone transport	0.002968007	Abat/Egfr/Anxa1/Ly6e/Tiam1/Nos2/Cry1/Arntl/Trpm2/Tnf/Slco4a1/Il1rn/Osm/Irs1/Edn3/Il1b/Ifng	17	BP
GO:0032355	response to estradiol	0.003009562	Egfr/Anxa1/Socs1/C3/Nos2/Socs3/Il10/Ifi27/Bid/Mmp9/Il1b/Areg/Cxcl1	13	BP
GO:0015800	acidic amino acid transport	0.003047875	Abat/Tnf/Il1rn/Il1b/Slc25a22/Cck	6	BP
GO:0036293	response to decreased oxygen levels	0.003138189	Abat/Tlr2/Mmp13/Casp12/Nos2/Plekhn1/Socs3/Hk2/Bmp2/Il1a/Pmaip1/Cx3cl1/Apaf1/Tnf/Ada/Mmp9/Il1b/Plk3/Nr4a2	19	BP
GO:1904407	positive regulation of nitric oxide metabolic process	0.003185429	Tlr2/Klrk1/Tnf/Il1b/Ifng	5	BP
GO:0002724	regulation of T cell cytokine production	0.003193538	B2m/Rsad2/Nlrp3/Il1b	4	BP
GO:0042036	negative regulation of cytokine biosynthetic process	0.003193538	Lag3/Il10/Zfp36/Bcl3	4	BP
GO:0045672	positive regulation of osteoclast differentiation	0.003193538	Il17a/Ccl3/Tnf/Ifng	4	BP
GO:0050779	RNA destabilization	0.003193538	Plekhn1/Zfp36/Zc3h12a/Zc3h12d	4	BP
GO:0090200	positive regulation of	0.003193538	Plaur/Pmaip1/Bid/Mmp9	4	BP

	release of cytochrome c from mitochondria				
GO:1901184	regulation of ERBB signaling pathway	0.003283946	Ereg/Plaur/Ceacam1/Ncf1/Epgn/Mmp9	6	BP
GO:0030004	cellular monovalent inorganic cation homeostasis	0.003347018	Slc9a5/Slc11a1/Slc4a5/Nox1/Slc4a7/Atp1a3/Tcirg1	7	BP
GO:0042698	ovulation cycle	0.003459689	Egfr/Anxa1/Mmp13/Ereg/Casp12/Bmp2/Trpm2/Mmp7	8	BP
GO:0034614	cellular response to reactive oxygen species	0.003495678	Egfr/Anxa1/Il10/Hk3/Stk25/Ncf1/Trpm2/Tnf/Mapk13/Mmp9/Il18bp	11	BP
GO:0006401	RNA catabolic process	0.003515305	Scgb1a1/Slc11a1/Plekhn1/Igf2bp1/Noct/Zfp36/Zc3hav1/Slfn13/Apobec1/Zc3h12a/Oas2/Zc3h12d	12	BP
GO:0001783	B cell apoptotic process	0.003623611	Cd74/Il10/Ada/Hsh2d	4	BP
GO:0006590	thyroid hormone generation	0.003638059	Duox2/Duoxa2/Duox1	3	BP
GO:0010934	macrophage cytokine production	0.003638059	Cd74/Tlr2/Sema7a	3	BP
GO:0032736	positive regulation of interleukin-13 production	0.003638059	RT1-S3/Lgals9/Nlrp3	3	BP
GO:0032740	positive regulation of interleukin-17 production	0.003638059	Il21/Osm/Nod2	3	BP
GO:0039535	regulation of RIG-I signaling pathway	0.003638059	Zc3hav1/Trim15/Dhx58	3	BP
GO:0051044	positive regulation of membrane protein ectodomain proteolysis	0.003638059	Tnf/Il1b/Ifng	3	BP
GO:2001267	regulation of cysteine-type endopeptidase activity involved in apoptotic signaling pathway	0.003638059	Lgals9/Plaur/Mmp9	3	BP
GO:0046330	positive regulation of JNK cascade	0.003656088	Tiam1/Nox1/Ceacam1/Il1a/Ncf1/Tnf/Il1rn/Il1b/Nod2	9	BP
GO:0006835	dicarboxylic acid transport	0.003778514	Abat/Tnf/Slc46a1/Il1rn/Il1b/Slc25a22/Cck	7	BP
GO:0071900	regulation of protein serine/threonine kinase activity	0.003811929	Cd74/Grm4/Egfr/Tiam1/Marcks/Bmp2/Ceacam1/Epgn/Dusp2/Tnf/Eif2ak2/Hhex/Il1rn/Edn3/Irgm/Il1b/Ifng/Nod2/Cdk5r1	19	BP
GO:0009620	response to fungus	0.003824859	Tlr2/Il17a/Ncf1/Cd86/Cxcl1	5	BP
GO:0009896	positive regulation of catabolic process	0.003910279	Tlr2/Dtx3l/Plekhn1/Hk2/Bid/Zfp36/Zc3hav1/Tnf/Rnf19b/Irs1/Zc3h12a/Irgm/Il1b/Ifng/Zc3h12d/Plk3/Nod2/Mefv	18	BP
GO:0002377	immunoglobulin production	0.004042745	RT1-S3/Batf/Ceacam1/Pou2f2/Tnf/Samhd1/Ifng/N	8	BP

			od2		
GO:0030641	regulation of cellular pH	0.004074112	Slc9a5/Slc11a1/Slc4a5/Nox1/Slc4a7/Tcirg1	6	BP
GO:0046626	regulation of insulin receptor signaling pathway	0.004074112	Socs1/Marcks/Socs3/Trim72/Irs1/Il1b	6	BP
GO:0006195	purine nucleotide catabolic process	0.00409135	Pde7a/Samhd1/Ada/Pde9a	4	BP
GO:0010939	regulation of necrotic cell death	0.00409135	Gzmb/Il17a/Birc3/Ripk3	4	BP
GO:0032673	regulation of interleu-kin-4 production	0.00409135	Scgb1a1/RT1-S3/Lgals9/Nlrp3	4	BP
GO:0090022	regulation of neutro-phil chemotaxis	0.00409135	Cd74/Lbp/Il1b/Nod2	4	BP
GO:0001666	response to hypoxia	0.004108588	Abat/Tlr2/Mmp13/Nos2/Plekhn1/Socs3/Hk2/Bmp2/Il1a/Pmaip1/Cx3cl1/Apaf1/Tnf/Ada/Mmp9/Il1b/Plk3/Nr4a2	18	BP
GO:0071622	regulation of granulo-cyte chemotaxis	0.004176813	Cd74/Lbp/Cx3cl1/Il1b/Nod2	5	BP
GO:0034599	cellular response to oxidative stress	0.004199415	Egfr/Anxa1/Nox1/Il10/Hk3/Stk25/Ncf1/Arntl/Trpm2/Tnf/Mapk13/Mmp9/Zc3h12a/Il18bp/Nr4a2	15	BP
GO:0097193	intrinsic apoptotic signaling pathway	0.004361538	Cd74/Casp12/Nox1/Plaur/Stk25/Casp4/Pmaip1/Apaf1/Bid/Tnf/Ripk3/Mmp9/Bcl3/Mael	14	BP
GO:0002903	negative regulation of B cell apoptotic pro-cess	0.004407311	Cd74/Ada/Hsh2d	3	BP
GO:0030656	regulation of vitamin metabolic process	0.004407311	Tnf/Il1b/Ifng	3	BP
GO:0032310	prostaglandin secre-tion	0.004407311	Nos2/Il1a/Il1b	3	BP
GO:0035457	cellular response to interferon-alpha	0.004407311	Ifit3/Oas1a/Ifit2	3	BP
GO:0042454	ribonucleoside cata-bolic process	0.004407311	Apobec3/Apobec1/Ada	3	BP
GO:0044827	modulation by host of viral genome replica-tion	0.004407311	Ceacam1/Ifi27/Zc3h12a	3	BP
GO:2000319	regulation of T-helper 17 cell differentiation	0.004407311	Nlrp3/Zc3h12a/Nfkbiz	3	BP
GO:0046427	positive regulation of receptor signaling pathway via JAK-STAT	0.004503367	Parp9/Socs1/Parp14/Il21/Osm/Cxcl6/Ifng	7	BP
GO:0045646	regulation of erythro-cyte differentiation	0.004551282	Stat1/Isg15/B2m/Mafb/Zfp36	5	BP
GO:0051955	regulation of amino acid transport	0.004551282	Abat/Tnf/Il1rn/Il1b/Cck	5	BP
GO:0061098	positive regulation of protein tyrosine kinase	0.004551282	Ereg/Ncf1/Unc119/Agrn/Epgn	5	BP

	activity				
GO:0002861	regulation of inflammatory response to antigenic stimulus	0.004598237	C3/Il10/Tnf/Nod2	4	BP
GO:0019835	cytolysis	0.004598237	Lbp/Gzmb/Gsdmd/Lyz2	4	BP
GO:0042554	superoxide anion generation	0.004598237	Egfr/Nox1/Ncf1/Cxcl1	4	BP
GO:0044849	estrous cycle	0.004598237	Anxa1/Mmp13/Trpm2/Mmp7	4	BP
GO:0046879	hormone secretion	0.004821217	Abat/Egfr/Anxa1/Ly6e/Tiam1/Nos2/Cry1/Arntl/Trpm2/Tnf/Il1rn/Osm/Irs1/Edn3/Il1b/Ifng	16	BP
GO:0002548	monocyte chemotaxis	0.004949028	Anxa1/Ccl4/Ccl3/Cx3cl1/Cxcl10	5	BP
GO:1902930	regulation of alcohol biosynthetic process	0.004949028	Bmp2/Plek/Tnf/Il1b/Ifng	5	BP
GO:1903311	regulation of mRNA metabolic process	0.004958173	Scgb1a1/Slc11a1/Plekhn1/Igf2bp1/Noct/Srsf12/Zfp36/Zc3hav1/Apobec1/Srrm4/Zc3h12a/Zc3h12d	12	BP
GO:0051785	positive regulation of nuclear division	0.004996764	Ereg/Il1a/Epgn/Tnf/Edn3/Il1b	6	BP
GO:0070482	response to oxygen levels	0.00505587	Abat/Tlr2/Mmp13/Casp12/Nos2/Plekhn1/Socs3/Nox1/Hk2/Bmp2/Il1a/Pmaip1/Cx3cl1/Apaf1/Tnf/Ada/Mmp9/Il1b/Plk3/Nr4a2	20	BP
GO:0048511	rhythmic process	0.005080081	Egfr/Anxa1/Mmp13/Ereg/Casp12/Nos2/Bmp2/Cry1/Noct/Arntl/Trpm2/Mmp7/Tnf/Ada/Egr2/Cdk5r1	16	BP
GO:0010543	regulation of platelet activation	0.005145713	Abat/Gp5/Plek/Ceacam1	4	BP
GO:0010574	regulation of vascular endothelial growth factor production	0.005145713	C3/Nox1/Il1a/Il1b	4	BP
GO:1902624	positive regulation of neutrophil migration	0.005145713	Cd74/Lbp/Il1a/Il1b	4	BP
GO:0006865	amino acid transport	0.00521998	Abat/Slc11a1/Grm4/Tnf/Il1rn/Il1b/Slc25a22/Cck/Slc7a9	9	BP
GO:0051090	regulation of DNA-binding transcription factor activity	0.005250487	Ddx58/Tlr2/Csf3/Lgals9/Il10/Cx3cl1/Tnf/Ripk3/Tnfrsf8/Trim15/Acod1/Eif2ak2/Nlrp3/Zc3h12a/Il1b/Anxa3/Nod2	17	BP
GO:0016045	detection of bacterium	0.005267803	Tlr2/Tlr1/Nod2	3	BP
GO:0042359	vitamin D metabolic process	0.005267803	Tnf/Il1b/Ifng	3	BP
GO:0044068	modulation by symbiont of host cellular process	0.005267803	Ccr5/Ceacam1/Zc3h12a	3	BP
GO:0050665	hydrogen peroxide biosynthetic process	0.005267803	Duox2/Ncf1/Duox1	3	BP
GO:0098543	detection of other organism	0.005267803	Tlr2/Tlr1/Nod2	3	BP
GO:0060968	regulation of gene silencing	0.005335889	Egfr/Adar/Zfp36/Apobec1/Zc3h12a/Mael	6	BP

GO:1902369	negative regulation of RNA catabolic process	0.005370807	Slc11a1/Igf2bp1/Noct/Zfp36/Apobec1	5	BP
GO:0030522	intracellular receptor signaling pathway	0.005472699	Ddx58/Nos2/Cry1/Arntl/Zc3hav1/Trim15/Tifa/Irgm/Dhx58/Ifih1/Nod2/Nr4a2	12	BP
GO:1903426	regulation of reactive oxygen species biosynthetic process	0.00562734	Tlr2/Il10/Klrk1/Tnf/Zc3h12a/Il1b/Ifng	7	BP
GO:1904894	positive regulation of receptor signaling pathway via STAT	0.00562734	Parp9/Socs1/Parp14/Il21/Osm/Cxcl6/Ifng	7	BP
GO:0002675	positive regulation of acute inflammatory response	0.005735181	C3/Ccr5/Tnf/Il1b	4	BP
GO:0010922	positive regulation of phosphatase activity	0.005735181	Bmp2/Plek/Ripk3/Ifng	4	BP
GO:0045940	positive regulation of steroid metabolic process	0.005735181	Il1a/Tnf/Il1b/Ifng	4	BP
GO:0070266	necroptotic process	0.005735181	Gzmb/Birc3/Tnf/Ripk3	4	BP
GO:0002455	humoral immune response mediated by circulating immunoglobulin	0.005817369	C3/C2/Tnf/Bcl3/Nod2	5	BP
GO:0032350	regulation of hormone metabolic process	0.005817369	Bmp2/Duoxa2/Tnf/Il1b/Ifng	5	BP
GO:0050850	positive regulation of calcium-mediated signaling	0.005817369	Ccl4/Ceacam1/Ccl3/Tnf/Ada	5	BP
GO:0046883	regulation of hormone secretion	0.005831675	Abat/Egfr/Anxa1/Tiam1/Nos2/Cry1/Arntl/Trpm2/Tnf/Osm/Irs1/Edn3/Il1b/Ifng	14	BP
GO:0045444	fat cell differentiation	0.006028831	Cebpd/Socs1/Bmp2/Ccr5/Noct/Arntl/Zfp36/Psmb8/Zc3h12a/Egr2/Napepld/Nr4a2	12	BP
GO:0032890	regulation of organic acid transport	0.006064246	Abat/Il1a/Tnf/Il1rn/Il1b/Cck	6	BP
GO:0030072	peptide hormone secretion	0.006070485	Abat/Egfr/Anxa1/Tiam1/Nos2/Arntl/Trpm2/Tnf/Il1rn/Irs1/Edn3/Il1b/Ifng	13	BP
GO:0022612	gland morphogenesis	0.006174694	Elf3/Egfr/Ceacam1/Hoxd13/Duox2/Tnf/Lama1/Ifng/Areg	9	BP
GO:0002755	MyD88-dependent toll-like receptor signaling pathway	0.006222334	Tlr2/Irf1/Irf7	3	BP
GO:0009164	nucleoside catabolic process	0.006222334	Apobec3/Apobec1/Ada	3	BP
GO:0010544	negative regulation of platelet activation	0.006222334	Abat/Gp5/Ceacam1	3	BP
GO:0032691	negative regulation of interleukin-1 beta production	0.006222334	Nlrp3/Zc3h12a/Mefv	3	BP
GO:0032780	negative regulation of	0.006222334	Tnnt2/Agrn/Tnni3	3	BP

	ATPase activity				
GO:0039531	regulation of viral-induced cytoplasmic pattern recognition receptor signaling pathway	0.006222334	Zc3hav1/Trim15/Dhx58	3	BP
GO:0045063	T-helper 1 cell differ-entiation	0.006222334	Sema4a/Anxa1/Jak3	3	BP
GO:0045624	positive regulation of T-helper cell differen-tiation	0.006222334	Anxa1/Nlrp3/Nfkbiz	3	BP
GO:0046886	positive regulation of hormone biosynthetic process	0.006222334	Tnf/Il1b/Ifng	3	BP
GO:0010573	vascular endothelial growth factor produc-tion	0.006367999	C3/Nox1/Il1a/Il1b	4	BP
GO:0050851	antigen receptor-mediated signaling pathway	0.006433134	Cea-cam1/Btnl7/Ada/Themis2/Lilrb4/Btnl5/Zc3h12a/Ifng/Nfkbiz	9	BP
GO:0044106	cellular amine meta-bolic process	0.006454391	Abat/Smox/Ido1/Moxd2/Napepld/Nr4a2	6	BP
GO:0034113	heterotypic cell-cell adhesion	0.0067878	Itgad/Il10/Tnf/Il1rn/Il1b	5	BP
GO:0042531	positive regulation of tyrosine phosphoryla-tion of STAT protein	0.0067878	Parp9/Parp14/Il21/Osm/Ifng	5	BP
GO:0051930	regulation of sensory perception of pain	0.0067878	Fcgr3a/Il10/Il1a/Ccl3/Cck	5	BP
GO:0070265	necrotic cell death	0.0067878	Gzmb/Il17a/Birc3/Tnf/Ripk3	5	BP
GO:1900076	regulation of cellular response to insulin stimulus	0.006862452	Socs1/Marcks/Socs3/Trim72/Irs1/Il1b	6	BP
GO:0043405	regulation of MAP kinase activity	0.006889465	Cd74/Grm4/Egfr/Tiam1/Bmp2/Ceacam1/Epgn/Dusp2/Tnf/Eif2ak2/Il1rn/Edn3/Il1b/Nod2	14	BP
GO:0002313	mature B cell differen-tiation involved in immune response	0.007273395	Dll1/Ada/Bcl3	3	BP
GO:0006691	leukotriene metabolic process	0.007273395	Pla2g5/Tlr2/Ncf1	3	BP
GO:0032693	negative regulation of interleukin-10 produc-tion	0.007273395	Dll1/Ido1/Jak3	3	BP
GO:0032823	regulation of natural killer cell differentia-tion	0.007273395	Lgals9/Prdm1/Il21	3	BP
GO:0034138	toll-like receptor 3 signaling pathway	0.007273395	Lgals9/Tnf/Cd86	3	BP
GO:0045623	negative regulation of T-helper cell differen-tiation	0.007273395	Anxa1/Zc3h12a/Jak3	3	BP

GO:0060544	regulation of necroptotic process	0.007273395	Gzmb/Birc3/Ripk3	3	BP
GO:1900424	regulation of defense response to bacterium	0.007273395	Lgals9/Cxcl6/Nod2	3	BP
GO:0042446	hormone biosynthetic process	0.007288884	Bmp2/Duox2/Duox1/Tnf/Il1b/Ifng	6	BP
GO:1903313	positive regulation of mRNA metabolic process	0.007288884	Plekhn1/Zfp36/Zc3hav1/Apobec1/Zc3h12a/Zc3h12d	6	BP
GO:0051931	regulation of sensory perception	0.007313127	Fcgr3a/Il10/Il1a/Ccl3/Cck	5	BP
GO:0046328	regulation of JNK cascade	0.007319851	Egfr/Tiam1/Nox1/Ceacam1/Il1a/Ncf1/Tnf/Il1rn/Il1b/Nod2	10	BP
GO:0008015	blood circulation	0.007493928	Abat/Slc4a5/Egfr/Stat1/Ccl4/Smtnl1/Dll1/Nos2/Nox1/Ceacam1/Tnnt2/Cx3cl1/Agrn/Tnni3/Ada/Atp1a3/Edn3/Zc3h12a/Ifng	19	BP
GO:0010212	response to ionizing radiation	0.007586704	Anxa1/Socs3/Ccr5/Il1a/Pmaip1/Apobec1/Cxcl6/Il1b/Cxcl10/Cxcl1	10	BP
GO:0002717	positive regulation of natural killer cell mediated immunity	0.007768897	RT1-S3/Lag3/Klrk1/Il21	4	BP
GO:0036230	granulocyte activation	0.007768897	Fcgr3a/Ccl3/Cxcl6/Anxa3	4	BP
GO:0046885	regulation of hormone biosynthetic process	0.007768897	Bmp2/Tnf/Il1b/Ifng	4	BP
GO:0051953	negative regulation of amine transport	0.007768897	Abat/Tnf/Il1rn/Il1b	4	BP
GO:1903792	negative regulation of anion transport	0.007768897	Abat/Tnf/Il1rn/Il1b	4	BP
GO:0006584	catecholamine metabolic process	0.00786615	Abat/Ly6e/Moxd2/Nr4a2/Dao	5	BP
GO:0009712	catechol-containing compound metabolic process	0.00786615	Abat/Ly6e/Moxd2/Nr4a2/Dao	5	BP
GO:0014009	glial cell proliferation	0.00786615	Cx3cl1/Tnf/Il1b/Ifng/Areg	5	BP
GO:0030073	insulin secretion	0.007892509	Abat/Anxa1/Tiam1/Nos2/Arntl/Trpm2/Tnf/Il1rn/Irs1/Il1b/Ifng	11	BP
GO:0043525	positive regulation of neuron apoptotic process	0.008198655	Ccl3/Pmaip1/Agrn/Tnf/Il1b/Cdk5r1	6	BP
GO:0009200	deoxyribonucleoside triphosphate metabolic process	0.008423181	Samhd1/Ada/Cmpk2	3	BP
GO:0034656	nucleobase-containing small molecule catabolic process	0.008423181	Apobec3/Apobec1/Ada	3	BP
GO:0048245	eosinophil chemotaxis	0.008423181	Ccl4/Ccl3/Cx3cl1	3	BP
GO:0051023	regulation of immunoglobulin secretion	0.008423181	RT1-S3/Ceacam1/Tnf	3	BP
GO:2000316	regulation of T-helper	0.008423181	Nlrp3/Zc3h12a/Nfkbiz	3	BP

	17 type immune response				
GO:1903428	positive regulation of reactive oxygen species biosynthetic process	0.008447572	Tlr2/Klrk1/Tnf/Il1b/Ifng	5	BP
GO:0032633	interleukin-4 production	0.008539465	Scgb1a1/RT1-S3/Lgals9/Nlrp3	4	BP
GO:0061900	glial cell activation	0.008539465	Egfr/Tlr2/Tlr1/Cx3cl1	4	BP
GO:2000403	positive regulation of lymphocyte migration	0.008539465	Ccl4/Lgals9/Ccl3/Cxcl10	4	BP
GO:0006885	regulation of pH	0.008682887	Slc9a5/Slc11a1/Slc4a5/Nox1/Slc4a7/Tcirg1	6	BP
GO:0042542	response to hydrogen peroxide	0.008729965	Stat1/Anxa1/Il10/Hk3/Stk25/Trpm2/Mapk13/Ada/Il18bp/Areg	10	BP
GO:0045807	positive regulation of endocytosis	0.008811324	Slc11a1/Lbp/C3/Dll1/C2/B2m/Tnf/Il1b/Nod2	9	BP
GO:0045926	negative regulation of growth	0.008812201	Sema4a/Tlr2/Lbp/Il10/Ccr5/Tbx5/Tnf/Ifng/Zc3h12d/Irf8/Sema7a/Nod2/Cdk5r1	13	BP
GO:0003013	circulatory system process	0.009039226	Abat/Slc4a5/Egfr/Stat1/Ccl4/Smtnl1/Dll1/Nos2/Nox1/Ceacam1/Tnnt2/Cx3cl1/Agrn/Tnni3/Ada/Atp1a3/Edn3/Zc3h12a/Ifng	19	BP
GO:0001836	release of cytochrome c from mitochondria	0.009058085	Plaur/Pmaip1/Bid/Mmp9/Cck	5	BP
GO:0003229	ventricular cardiac muscle tissue development	0.009058085	Ly6e/Marcks/Tbx5/Tnnt2/Tnni3	5	BP
GO:0051148	negative regulation of muscle cell differentiation	0.009058085	Ereg/Dll1/Bmp2/Trim72/Cxcl10	5	BP
GO:0002920	regulation of humoral immune response	0.009358355	C3/Tnf/Acod1/Nod2	4	BP
GO:0033028	myeloid cell apoptotic process	0.009358355	Anxa1/Ccr5/Ifng/Nod2	4	BP
GO:0006402	mRNA catabolic process	0.00967081	Scgb1a1/Slc11a1/Plekhn1/Igf2bp1/Noct/Zfp36/Zc3hav1/Apobec1/Zc3h12a/Zc3h12d	10	BP
GO:0035634	response to stilbenoid	0.009673609	Usp18/Ifit3/Il1b	3	BP
GO:1900409	positive regulation of cellular response to oxidative stress	0.009673609	Nox1/Trpm2/Tnf	3	BP
GO:0002886	regulation of myeloid leukocyte mediated immunity	0.009698366	Ddx58/C3/Lgals9/Cxcl6/Cxcl1	5	BP
GO:0045123	cellular extravasation	0.009698366	Il1a/Cx3cl1/Tnf/Ripk3/Trem3	5	BP
GO:0008637	apoptotic mitochondrial changes	0.009797706	Stpg1/Hk2/Plaur/Pmaip1/Bid/Mmp9/Cck	7	BP
GO:0048661	positive regulation of smooth muscle cell proliferation	0.009797706	Egfr/Stat1/Ereg/Nox1/Cx3cl1/Tnf/Mmp9	7	BP

GO:0019722	calcium-mediated signaling	0.009853173	Cxcr3/Ccl4/Ccr5/Ceacam1/Ccl3/Trpm2/Tnni3/Tnf/Ada/Irgm/Ccrl2	11	BP
GO:0060964	regulation of gene silencing by miRNA	0.010226688	Egfr/Zfp36/Zc3h12a/Mael	4	BP
GO:0150076	neuroinflammatory response	0.010226688	Egfr/Tlr2/Tlr1/Cx3cl1	4	BP
GO:1900744	regulation of p38MAPK cascade	0.010226688	Bmp2/Ncf1/Zc3h12a/Il1b	4	BP
GO:0009308	amine metabolic process	0.010258252	Abat/Smox/Ido1/Moxd2/Napepld/Nr4a2	6	BP
GO:0033077	T cell differentiation in thymus	0.010258252	Cd74/B2m/Mafb/Ripk3/Ada/Il1b	6	BP
GO:0001503	ossification	0.010279075	Col11a2/Cebpd/Mmp13/Bmp2/Isg15/Ccl3/Adar/Duox2/Noct/Rsad2/Chrd/Tnf/Sbno2/Mmp9/Egr2/Areg	16	BP
GO:0001649	osteoblast differentiation	0.010339781	Col11a2/Cebpd/Mmp13/Bmp2/Ccl3/Adar/Noct/Chrd/Tnf/Areg	10	BP
GO:0045600	positive regulation of fat cell differentiation	0.010369081	Bmp2/Noct/Zfp36/Zc3h12a/Napepld	5	BP
GO:0051224	negative regulation of protein transport	0.010687196	Anxa1/Lgals9/Il10/Cx3cl1/Rsad2/Tnf/Nlrp3/Irs1/Zc3h12a/Il1b	10	BP
GO:0009110	vitamin biosynthetic process	0.011026328	Tnf/Il1b/Ifng	3	BP
GO:0017000	antibiotic biosynthetic process	0.011026328	Duox2/Ncf1/Duox1	3	BP
GO:0031664	regulation of lipopolysaccharide-mediated signaling pathway	0.011026328	Prdm1/Cx3cl1/Acod1	3	BP
GO:1901223	negative regulation of NIK/NF-kappaB signaling	0.011026328	Litaf/Nlrp3/Zc3h12a	3	BP
GO:0009266	response to temperature stimulus	0.011130706	Tfec/Casp12/Socs3/Il1a/Trpm2/Irf1/Osm/Mmp9/Il1b/Cxcl10/Cxcl1	11	BP
GO:0002832	negative regulation of response to biotic stimulus	0.011145536	Ceacam1/Prdm1/Acod1/Dhx58	4	BP
GO:0051154	negative regulation of striated muscle cell differentiation	0.011145536	Dll1/Bmp2/Trim72/Cxcl10	4	BP
GO:0048660	regulation of smooth muscle cell proliferation	0.011803422	Egfr/Stat1/Ereg/Nox1/Il10/Cx3cl1/Tnf/Mmp9/Ifng	9	BP
GO:0002763	positive regulation of myeloid leukocyte differentiation	0.011804404	Cd74/Il17a/Ccl3/Tnf/Ifng	5	BP
GO:0051235	maintenance of location	0.011847527	Cxcr3/C3/Hk2/Lgals9/Cry1/Ccr5/Ccl3/Cx3cl1/Trpm2/Tnf/Zc3h12a/Il1b/Cxcl10/Cxcl9	14	BP
GO:0097300	programmed necrotic cell death	0.012115915	Gzmb/Birc3/Tnf/Ripk3	4	BP

GO:2001024	negative regulation of response to drug	0.012115915	Abat/Il10/Hk3/Tnf	4	BP
GO:0007254	JNK cascade	0.012166224	Egfr/Tiam1/Nox1/Ceacam1/Il1a/Ncf1/Tnf/Il1rn/Il1b/Nod2	10	BP
GO:1904950	negative regulation of establishment of protein localization	0.012166224	Anxa1/Lgals9/Il10/Cx3cl1/Rsad2/Tnf/Nlrp3/Irs1/Zc3h12a/Il1b	10	BP
GO:0035743	CD4-positive, alpha-beta T cell cytokine production	0.012482737	Rsad2/Nlrp3/Il1b	3	BP
GO:0095500	acetylcholine receptor signaling pathway	0.012482737	Ly6e/Agrn/Cdk5r1	3	BP
GO:1903831	signal transduction involved in cellular response to ammonium ion	0.012482737	Ly6e/Agrn/Cdk5r1	3	BP
GO:1905144	response to acetylcholine	0.012482737	Ly6e/Agrn/Cdk5r1	3	BP
GO:1905145	cellular response to acetylcholine	0.012482737	Ly6e/Agrn/Cdk5r1	3	BP
GO:0002673	regulation of acute inflammatory response	0.01257027	C3/Ccr5/Tnf/Nlrp3/Il1b	5	BP
GO:0046718	viral entry into host cell	0.01257027	Cd74/Tmprss2/Lgals9/Ceacam1/Nectin4	5	BP
GO:0002823	negative regulation of adaptive immune response based on somatic recombination of immune receptors built from immunoglobulin superfamily domains	0.013138793	Ceacam1/Zc3h12a/Nod2/Jak3	4	BP
GO:0006509	membrane protein ectodomain proteolysis	0.013138793	Il10/Tnf/Il1b/Ifng	4	BP
GO:0006576	cellular biogenic amine metabolic process	0.013138793	Smox/Ido1/Moxd2/Napepld	4	BP
GO:0042417	dopamine metabolic process	0.013138793	Abat/Moxd2/Nr4a2/Dao	4	BP
GO:0046456	icosanoid biosynthetic process	0.013138793	Cd74/Pla2g5/Anxa1/Cyp4a3	4	BP
GO:0072523	purine-containing compound catabolic process	0.013138793	Pde7a/Samhd1/Ada/Pde9a	4	BP
GO:0090559	regulation of membrane permeability	0.013369087	Lbp/Stpg1/Hk2/Pmaip1/Bid	5	BP
GO:0002262	myeloid cell homeostasis	0.013556989	Stat1/Anxa1/Isg15/Adar/B2m/Mafb/Zfp36/Ifng/Jak3	9	BP
GO:0033273	response to vitamin	0.013556989	Egfr/Nos2/Il1a/Serpina3n/Eif2ak2/Ada/Mmp9/Il1b/Cxcl10	9	BP

GO:0051098	regulation of binding	0.013681471	Parp9/Tiam1/Stpg1/Csf3/Dtx3l/Lgals9/Il10/Bmp2/Plaur/B2m/Agrn/Ifit2/Mitd1/Mmp9/Bcl3/Ifng	16	BP
GO:0042063	gliogenesis	0.014007356	Egfr/Anxa1/Tlr2/Tiam1/Dll1/Bmp2/Ccl3/Cx3cl1/Tnf/Il1b/Ifng/Egr2/Areg/Cdk5r1	14	BP
GO:0002719	negative regulation of cytokine production involved in immune response	0.014043994	Il10/Tnf/Jak3	3	BP
GO:0070233	negative regulation of T cell apoptotic process	0.014043994	Ido1/Ada/Jak3	3	BP
GO:0072677	eosinophil migration	0.014043994	Ccl4/Ccl3/Cx3cl1	3	BP
GO:1900120	regulation of receptor binding	0.014043994	Il10/B2m/Mmp9	3	BP
GO:1902884	positive regulation of response to oxidative stress	0.014043994	Nox1/Trpm2/Tnf	3	BP
GO:1905208	negative regulation of cardiocyte differentiation	0.014043994	Egfr/Dll1/Bmp2	3	BP
GO:0001763	morphogenesis of a branching structure	0.014062333	Socs3/Il10/Bmp2/Hoxd13/Prdm1/Hoxb7/Tnf/Hhex/Lama1/Il1b/Areg	11	BP
GO:0019915	lipid storage	0.014201443	C3/Cry1/Tnf/Zc3h12a/Il1b	5	BP
GO:0030856	regulation of epithelial cell differentiation	0.014320682	Dll1/Ceacam1/Il1a/Arntl/Zfp36/Tnf/Il1b/Ifng	8	BP
GO:0048659	smooth muscle cell proliferation	0.014502904	Egfr/Stat1/Ereg/Nox1/Il10/Cx3cl1/Tnf/Mmp9/Ifng	9	BP
GO:0050871	positive regulation of B cell activation	0.01470434	Cd74/Prdm1/Il21/Ada/Ifng/Nod2	6	BP
GO:0048871	multicellular organismal homeostasis	0.014869277	Col11a2/Abat/Slc11a1/Egfr/Fcgr3a/Dll1/Ccr5/Ceacam1/Il1a/Cdhr1/Arntl/Trpm2/Tnf/Il1rn/Il1b/Grhl3/Napepld/Nod2	18	BP
GO:0043393	regulation of protein binding	0.014878881	Tiam1/Stpg1/Csf3/Dtx3l/Il10/Bmp2/B2m/Agrn/Ifit2/Mitd1/Mmp9	11	BP
GO:0030888	regulation of B cell proliferation	0.015067911	Cd74/Il10/Prdm1/Il21/Ada	5	BP
GO:0044409	entry into host	0.015067911	Cd74/Tmprss2/Lgals9/Ceacam1/Nectin4	5	BP
GO:0032147	activation of protein kinase activity	0.015172264	Cd74/Slc11a1/Grm4/Egfr/Socs1/Bmp2/Stk25/Epgn/Tnf/Ripk3/Eif2ak2/Il1b	12	BP
GO:1901216	positive regulation of neuron death	0.015229039	Ccl3/Pmaip1/Trpm2/Agrn/Tnf/Il1b/Cdk5r1	7	BP
GO:0033002	muscle cell proliferation	0.015300312	Megf10/Egfr/Stat1/Ereg/Nox1/Il10/Tbx5/Cx3cl1/Tnf/Mmp9/Ifng	11	BP
GO:0014066	regulation of phosphatidylinositol 3-kinase signaling	0.015433535	Egfr/Csf3/Ceacam1/Ncf1/Tnf/Osm	6	BP
GO:0007567	parturition	0.015711029	Mmp13/Arntl/Mmp9	3	BP
GO:0030574	collagen catabolic	0.015711029	Mmp13/Mmp7/Mmp9	3	BP

	process				
GO:0032352	positive regulation of hormone metabolic process	0.015711029	Tnf/Il1b/Ifng	3	BP
GO:0032753	positive regulation of interleukin-4 production	0.015711029	RT1-S3/Lgals9/Nlrp3	3	BP
GO:0032897	negative regulation of viral transcription	0.015711029	Ccl4/Ccl3/Zfp36	3	BP
GO:0048521	negative regulation of behavior	0.015711029	Ada/Cck/Napepld	3	BP
GO:0071425	hematopoietic stem cell proliferation	0.015711029	Thpo/Eif2ak2/Cxcl1	3	BP
GO:0090023	positive regulation of neutrophil chemotaxis	0.015711029	Cd74/Lbp/Il1b	3	BP
GO:0007204	positive regulation of cytosolic calcium ion concentration	0.015727695	Cxcr3/Ptger1/Ccr5/Ccl3/Gnat2/Cx3cl1/Trpm2/Cxcl3/Il1b/Ccrl2/Cxcl10/Cxcl1/Jak3/Cxcl9	14	BP
GO:0030183	B cell differentiation	0.015870008	Dll1/Prdm1/Pou2f2/Hhex/Ada/Bcl3/Jak3	7	BP
GO:2001233	regulation of apoptotic signaling pathway	0.016176669	Cd74/Nox1/Lgals9/Plaur/Pmaip1/Cx3cl1/Apaf1/Bid/Runx3/Tnf/Ripk3/Osm/Mmp9/Il1b/Nr4a2/Jak3	16	BP
GO:0002714	positive regulation of B cell mediated immunity	0.016531286	C3/Tnf/Ifng/Nod2	4	BP
GO:0002820	negative regulation of adaptive immune response	0.016531286	Ceacam1/Zc3h12a/Nod2/Jak3	4	BP
GO:0002891	positive regulation of immunoglobulin mediated immune response	0.016531286	C3/Tnf/Ifng/Nod2	4	BP
GO:0032768	regulation of monooxygenase activity	0.016531286	Egfr/Tnf/Il1b/Ifng	4	BP
GO:0007215	glutamate receptor signaling pathway	0.016966349	Grm4/Tiam1/Cx3cl1/Atp1a3/Anxa3/Cdk5r1	6	BP
GO:1903522	regulation of blood circulation	0.017076324	Egfr/Smtnl1/Nos2/Tnnt2/Cx3cl1/Agrn/Tnni3/Ada/Edn3/Zc3h12a/Ifng	11	BP
GO:0050796	regulation of insulin secretion	0.017080056	Abat/Tiam1/Nos2/Arntl/Trpm2/Tnf/Irs1/Il1b/Ifng	9	BP
GO:0016999	antibiotic metabolic process	0.017174076	Egfr/Nox1/Duox2/Duoxa2/Ncf1/Duox1/Cyp4a3/Acod1	8	BP
GO:0070301	cellular response to hydrogen peroxide	0.017208971	Anxa1/Il10/Hk3/Stk25/Trpm2/Mapk13/Il18bp	7	BP
GO:1901343	negative regulation of vasculature development	0.017208971	Cxcr3/Sema4a/Il17f/Stat1/Hhex/Ifng/Cxcl10	7	BP
GO:0007176	regulation of epidermal growth factor-activated receptor	0.017484558	Ereg/Ncf1/Epgn	3	BP

	activity				
GO:0009065	glutamine family amino acid catabolic process	0.017484558	Padi3/Nos2/Dao	3	BP
GO:0009394	2'-deoxyribonucleotide metabolic process	0.017484558	Samhd1/Ada/Cmpk2	3	BP
GO:0019692	deoxyribose phosphate metabolic process	0.017484558	Samhd1/Ada/Cmpk2	3	BP
GO:0032461	positive regulation of protein oligomeriza-tion	0.017484558	Pmaip1/Bid/Cck	3	BP
GO:0043032	positive regulation of macrophage activation	0.017484558	Lbp/Lgals9/Il10	3	BP
GO:0044788	modulation by host of viral process	0.017484558	Ceacam1/Ifi27/Zc3h12a	3	BP
GO:0048857	neural nucleus devel-opment	0.017484558	Slc4a7/Kirrel3/Cdk5r1	3	BP
GO:0009612	response to mechani-cal stimulus	0.01754853	Egfr/Stat1/Mmp13/Nos2/Bmp2/Irf1/Mmp7/Tnf/Tnfrsf8/Eif2ak2/Mmp9/Il1b/Cxcl10	13	BP
GO:0032892	positive regulation of organic acid transport	0.017772761	Abat/Il1a/Il1b/Cck	4	BP
GO:0051281	positive regulation of release of sequestered calcium ion into cyto-sol	0.017772761	Cxcr3/Cx3cl1/Cxcl10/Cxcl9	4	BP
GO:0014065	phosphatidylinositol 3-kinase signaling	0.017907505	Egfr/Csf3/Ceacam1/Ncf1/Tnf/Osm/Irs1	7	BP
GO:0051209	release of sequestered calcium ion into cyto-sol	0.017907505	Cxcr3/Ccr5/Ccl3/Cx3cl1/Trpm2/Cxcl10/Cxcl9	7	BP
GO:0098742	cell-cell adhesion via plasma-membrane adhesion molecules	0.018050401	Il10/Bmp2/Ceacam1/Nectin4/Cdhr1/Cx3cl1/Il1rn/Kirrel3/Anxa3/Dsg3	10	BP
GO:0007191	adenylate cyclase-activating dopamine receptor signaling pathway	0.018295062	Gnal/Ptger1	2	BP
GO:0018298	protein-chromophore linkage	0.018295062	Cry1/Opn3	2	BP
GO:0034115	negative regulation of heterotypic cell-cell adhesion	0.018295062	Il10/Il1rn	2	BP
GO:0044793	negative regulation by host of viral process	0.018295062	Ceacam1/Zc3h12a	2	BP
GO:0046386	deoxyribose phosphate catabolic process	0.018295062	Samhd1/Ada	2	BP
GO:0070244	negative regulation of thymocyte apoptotic process	0.018295062	Ada/Jak3	2	BP
GO:0070587	regulation of cell-cell	0.018295062	Il10/Il1rn	2	BP

	adhesion involved in gastrulation				
GO:0071888	macrophage apoptotic process	0.018295062	Ccr5/Nod2	2	BP
GO:2000109	regulation of macro-phage apoptotic pro-cess	0.018295062	Ccr5/Nod2	2	BP
GO:0032868	response to insulin	0.018390865	Stat1/Tlr2/Socs1/Marcks/Socs3/Il10/Cry1/Ceacam1/Trim72/Apobec1/Irs1/Il1b/Egr2	13	BP
GO:0001659	temperature homeosta-sis	0.018778538	Abat/Ccr5/Il1a/Arntl/Trpm2/Tnf/Il1rn/Il1b/Napepld	9	BP
GO:0010822	positive regulation of mitochondrion organi-zation	0.018885747	Hk2/Plaur/Pmaip1/Bid/Mmp9	5	BP
GO:0032570	response to progester-one	0.018885747	Tlr2/Socs1/C3/Socs3/Acod1	5	BP
GO:0035690	cellular response to drug	0.018964568	Egfr/Anxa1/Ly6e/Ccl4/Nos2/Il10/Hk3/Stk25/Trpm2/Agrn/Tnf/Mapk13/Acod1/Zc3h12a/Il1b/Il18bp/Nod2/Cdk5r1	18	BP
GO:0034504	protein localization to nucleus	0.01904911	Parp9/Dtx3l/Adar/Arntl/Tnf/Hhex/Gbp2/Lilrb4/Irs1/Zc3h12a/Bcl3/Ifng	12	BP
GO:0001960	negative regulation of cytokine-mediated signaling pathway	0.01907077	Parp14/Adar/Samhd1/Il1rn	4	BP
GO:0030517	negative regulation of axon extension	0.01907077	Sema4a/Ccr5/Sema7a/Cdk5r1	4	BP
GO:0090199	regulation of release of cytochrome c from mitochondria	0.01907077	Plaur/Pmaip1/Bid/Mmp9	4	BP
GO:0009166	nucleotide catabolic process	0.019364	Hk2/Pde7a/Hk3/Eno3/Samhd1/Ada/Pde9a	7	BP
GO:0051283	negative regulation of sequestering of calci-um ion	0.019364	Cxcr3/Ccr5/Ccl3/Cx3cl1/Trpm2/Cxcl10/Cxcl9	7	BP
GO:0009713	catechol-containing compound biosynthet-ic process	0.019365092	Moxd2/Nr4a2/Dao	3	BP
GO:0014048	regulation of gluta-mate secretion	0.019365092	Il1rn/Il1b/Cck	3	BP
GO:0032770	positive regulation of monooxygenase ac-tivity	0.019365092	Tnf/Il1b/Ifng	3	BP
GO:0042423	catecholamine biosyn-thetic process	0.019365092	Moxd2/Nr4a2/Dao	3	BP
GO:0071624	positive regulation of granulocyte chemotax-is	0.019365092	Cd74/Lbp/Il1b	3	BP
GO:1900117	regulation of execu-tion phase of apoptosis	0.019365092	Cxcr3/Apaf1/Zc3h12a	3	BP
GO:0043270	positive regulation of	0.019711177	Cxcr3/Abat/Ccl4/Il1a/Ccl3/Cx3cl1/Edn3/Il1b	13	BP

	ion transport		/Cck/Cxcl10/Cxcl1/Jak3/Cxcl9		
GO:0051896	regulation of protein kinase B signaling	0.019734946	Egfr/Csf3/F10/Thpo/Ccl3/Cx3cl1/Tnf/Osm	8	BP
GO:0030278	regulation of ossifica-tion	0.020224223	Ce-bpd/Bmp2/Isg15/Ccl3/Noct/Rsad2/Chrd/Tnf/Egr2/Areg	10	BP
GO:0043551	regulation of phospha-tidylinositol 3-kinase activity	0.020425962	Socs1/Socs6/Irs1/Nod2	4	BP
GO:0051282	regulation of seques-tering of calcium ion	0.020901447	Cxcr3/Ccr5/Ccl3/Cx3cl1/Trpm2/Cxcl10/Cxcl9	7	BP
GO:0035235	ionotropic glutamate receptor signaling pathway	0.021352949	Tiam1/Atp1a3/Cdk5r1	3	BP
GO:0045662	negative regulation of myoblast differentia-tion	0.021352949	Dll1/Tnf/Cxcl10	3	BP
GO:1901658	glycosyl compound catabolic process	0.021352949	Apobec3/Apobec1/Ada	3	BP
GO:0030218	erythrocyte differen-tiation	0.021701155	Stat1/Isg15/Adar/B2m/Mafb/Zfp36/Jak3	7	BP
GO:0051208	sequestering of calci-um ion	0.021701155	Cxcr3/Ccr5/Ccl3/Cx3cl1/Trpm2/Cxcl10/Cxcl9	7	BP
GO:0007596	blood coagulation	0.021826307	Abat/Gp5/C3/F10/Plek/Ceacam1/Cx3cl1/Serpinc1	8	BP
GO:0002420	natural killer cell mediated cytotoxicity directed against tumor cell target	0.022048243	Ceacam1/Klrk1	2	BP
GO:0002423	natural killer cell mediated immune response to tumor cell	0.022048243	Ceacam1/Klrk1	2	BP
GO:0002645	positive regulation of tolerance induction	0.022048243	RT1-A1/Ido1	2	BP
GO:0002664	regulation of T cell tolerance induction	0.022048243	Ido1/Cd86	2	BP
GO:0002855	regulation of natural killer cell mediated immune response to tumor cell	0.022048243	Ceacam1/Klrk1	2	BP
GO:0002858	regulation of natural killer cell mediated cytotoxicity directed against tumor cell target	0.022048243	Ceacam1/Klrk1	2	BP
GO:0009214	cyclic nucleotide catabolic process	0.022048243	Pde7a/Pde9a	2	BP
GO:0009264	deoxyribonucleotide catabolic process	0.022048243	Samhd1/Ada	2	BP
GO:0035739	CD4-positive, alpha-beta T cell prolifera-	0.022048243	Lgals9/Ceacam1	2	BP

	tion				
GO:0043301	negative regulation of leukocyte degranulation	0.022048243	Lgals9/Ceacam1	2	BP
GO:0060340	positive regulation of type I interferon-mediated signaling pathway	0.022048243	Zbp1/Irf7	2	BP
GO:0070391	response to lipoteichoic acid	0.022048243	Tlr2/Lbp	2	BP
GO:0070586	cell-cell adhesion involved in gastrulation	0.022048243	Il10/Il1rn	2	BP
GO:0071223	cellular response to lipoteichoic acid	0.022048243	Tlr2/Lbp	2	BP
GO:1900426	positive regulation of defense response to bacterium	0.022048243	Lgals9/Nod2	2	BP
GO:1902075	cellular response to salt	0.022048243	Mapk13/Zc3h12a	2	BP
GO:2000341	regulation of chemokine (C-X-C motif) ligand 2 production	0.022048243	Cd74/Tnf	2	BP
GO:2000561	regulation of CD4-positive, alpha-beta T cell proliferation	0.022048243	Lgals9/Ceacam1	2	BP
GO:2001034	positive regulation of double-strand break repair via nonhomologous end joining	0.022048243	Parp9/Dtx3l	2	BP
GO:0002312	B cell activation involved in immune response	0.022132708	Batf/Dll1/Ada/Bcl3/Ifng	5	BP
GO:0030193	regulation of blood coagulation	0.022132708	Abat/Gp5/Plek/Ceacam1/Serpinc1	5	BP
GO:0050810	regulation of steroid biosynthetic process	0.022132708	Bmp2/Il1a/Tnf/Il1b/Ifng	5	BP
GO:0014823	response to activity	0.022521846	Smtnl1/Nos2/Il10/Tnf/Il1rn/Perm1/Irs1	7	BP
GO:0014074	response to purine-containing compound	0.02320337	Pla2g5/Gnal/Stat1/Duox2/Serpina3n/Duox1/Trpm2/Irs1/Il1b/Areg	10	BP
GO:0071674	mononuclear cell migration	0.023290535	Anxa1/Ccl4/Ccl3/Cx3cl1/Cxcl10	5	BP
GO:1900046	regulation of hemostasis	0.023290535	Abat/Gp5/Plek/Ceacam1/Serpinc1	5	BP
GO:0007599	hemostasis	0.023304735	Abat/Gp5/C3/F10/Plek/Ceacam1/Cx3cl1/Serpinc1	8	BP
GO:2001239	regulation of extrinsic apoptotic signaling pathway in absence of ligand	0.023310221	Cx3cl1/Tnf/Il1b/Jak3	4	BP

GO:0001774	microglial cell activation	0.023448266	Tlr2/Tlr1/Cx3cl1	3	BP
GO:0002269	leukocyte activation involved in inflammatory response	0.023448266	Tlr2/Tlr1/Cx3cl1	3	BP
GO:0031645	negative regulation of nervous system process	0.023448266	Il10/Tnf/Ifng	3	BP
GO:0060571	morphogenesis of an epithelial fold	0.023448266	Egfr/Hoxd13/Hhex	3	BP
GO:1902932	positive regulation of alcohol biosynthetic process	0.023448266	Tnf/Il1b/Ifng	3	BP
GO:0090276	regulation of peptide hormone secretion	0.02447971	Abat/Egfr/Tiam1/Nos2/Arntl/Trpm2/Tnf/Irs1/Il1b/Ifng	10	BP
GO:0060761	negative regulation of response to cytokine stimulus	0.024840321	Parp14/Adar/Samhd1/Il1rn	4	BP
GO:0097191	extrinsic apoptotic signaling pathway	0.025136513	Il1a/Ifi27/Pmaip1/Cx3cl1/Bid/Runx3/Tnf/Il1b/Ifng/Jak3	10	BP
GO:0033138	positive regulation of peptidyl-serine phosphorylation	0.025162149	Egfr/Csf3/Tnf/Osm/Irgm/Ifng	6	BP
GO:0007584	response to nutrient	0.025285932	Egfr/Stat1/Nos2/C2/Il1a/Serpina3n/Apaf1/Eif2ak2/Ada/Serpinc1/Mmp9/Il1b/Cxcl10/Nod2	14	BP
GO:0006471	protein ADP-ribosylation	0.025651006	Parp9/Parp14/Ifng	3	BP
GO:0042119	neutrophil activation	0.025651006	Fcgr3a/Cxcl6/Anxa3	3	BP
GO:0050832	defense response to fungus	0.025651006	Il17a/Ncf1/Cxcl1	3	BP
GO:0051085	chaperone cofactor-dependent protein refolding	0.025651006	Dnajb1/Cd74/RT1-DMb	3	BP
GO:0038034	signal transduction in absence of ligand	0.025721902	Il1a/Cx3cl1/Tnf/Il1b/Jak3	5	BP
GO:0097192	extrinsic apoptotic signaling pathway in absence of ligand	0.025721902	Il1a/Cx3cl1/Tnf/Il1b/Jak3	5	BP
GO:1903312	negative regulation of mRNA metabolic process	0.025721902	Slc11a1/Igf2bp1/Noct/Zfp36/Apobec1	5	BP
GO:0001554	luteolysis	0.026088936	Mmp13/Casp12	2	BP
GO:0006527	arginine catabolic process	0.026088936	Padi3/Nos2	2	BP
GO:0009143	nucleoside triphosphate catabolic process	0.026088936	Samhd1/Ada	2	BP
GO:0009215	purine deoxyribonucleoside triphosphate metabolic process	0.026088936	Samhd1/Ada	2	BP

GO:0032308	positive regulation of prostaglandin secretion	0.026088936	Il1a/Il1b	2	BP
GO:0032725	positive regulation of granulocyte macrophage colony-stimulating factor production	0.026088936	Ddx58/Il1b	2	BP
GO:0033089	positive regulation of T cell differentiation in thymus	0.026088936	Ada/Il1b	2	BP
GO:0043922	negative regulation by host of viral transcription	0.026088936	Ccl4/Ccl3	2	BP
GO:0045628	regulation of T-helper 2 cell differentiation	0.026088936	Anxa1/Nlrp3	2	BP
GO:0046007	negative regulation of activated T cell proliferation	0.026088936	Lgals9/Ido1	2	BP
GO:0051024	positive regulation of immunoglobulin secretion	0.026088936	RT1-S3/Ceacam1	2	BP
GO:0051775	response to redox state	0.026088936	Nos2/Arntl	2	BP
GO:0061299	retina vasculature morphogenesis in camera-type eye	0.026088936	Slc4a7/Lama1	2	BP
GO:0098792	xenophagy	0.026088936	Tlr2/Nod2	2	BP
GO:1901550	regulation of endothelial cell development	0.026088936	Tnf/Il1b	2	BP
GO:1902916	positive regulation of protein polyubiquitination	0.026088936	Birc3/Nod2	2	BP
GO:1903140	regulation of establishment of endothelial barrier	0.026088936	Tnf/Il1b	2	BP
GO:1903998	regulation of eating behavior	0.026088936	Cck/Napepld	2	BP
GO:2000322	regulation of glucocorticoid receptor signaling pathway	0.026088936	Cry1/Arntl	2	BP
GO:0010039	response to iron ion	0.026429669	Abat/Casp12/B2m/Mmp9	4	BP
GO:0033619	membrane protein proteolysis	0.026429669	Il10/Tnf/Il1b/Ifng	4	BP
GO:0045668	negative regulation of osteoblast differentiation	0.026429669	Noct/Chrd/Tnf/Areg	4	BP
GO:0031644	regulation of nervous system process	0.026470289	Abat/Fcgr3a/Il10/Il1a/Ccl3/Tnf/Ifng/Cck	8	BP
GO:0050817	coagulation	0.026470289	Abat/Gp5/C3/F10/Plek/Ceacam1/Cx3cl1/Serpinc1	8	BP

GO:1901292	nucleoside phosphate catabolic process	0.026948205	Hk2/Pde7a/Hk3/Eno3/Samhd1/Ada/Pde9a	7	BP
GO:0050818	regulation of coagulation	0.026996189	Abat/Gp5/Plek/Ceacam1/Serpinc1	5	BP
GO:0050864	regulation of B cell activation	0.027899624	Cd74/Il10/Prdm1/Il21/Ada/Ifng/Nod2	7	BP
GO:0051881	regulation of mitochondrial membrane potential	0.02831001	Bco2/Pmaip1/Bid/Hsh2d/Cck	5	BP
GO:0006690	icosanoid metabolic process	0.028393181	Cd74/Pla2g5/Anxa1/Tlr2/Ncf1/Cyp4a3	6	BP
GO:0008630	intrinsic apoptotic signaling pathway in response to DNA damage	0.028393181	Cd74/Pmaip1/Bid/Tnf/Bcl3/Mael	6	BP
GO:0050878	regulation of body fluid levels	0.029084255	Abat/Slc4a5/Mmp13/Gp5/C3/Hk2/F10/Plek/Ceacam1/Cx3cl1/Ada/Serpinc1/Grhl3/Oas2	14	BP
GO:0016525	negative regulation of angiogenesis	0.029528138	Cxcr3/Sema4a/Il17f/Stat1/Hhex/Cxcl10	6	BP
GO:0046902	regulation of mitochondrial membrane permeability	0.029787609	Stpg1/Hk2/Pmaip1/Bid	4	BP
GO:0050732	negative regulation of peptidyl-tyrosine phosphorylation	0.029787609	Socs1/Parp14/Socs3/Irf1	4	BP
GO:0055067	monovalent inorganic cation homeostasis	0.029870302	Slc9a5/Slc11a1/Slc4a5/Nox1/Slc4a7/Atp1a3/Tcirg1	7	BP
GO:0001779	natural killer cell differentiation	0.030377803	Lgals9/Prdm1/Il21	3	BP
GO:0009262	deoxyribonucleotide metabolic process	0.030377803	Samhd1/Ada/Cmpk2	3	BP
GO:0050482	arachidonic acid secretion	0.030377803	Pla2g5/Anxa1/Pla2g2a	3	BP
GO:0060142	regulation of syncytium formation by plasma membrane fusion	0.030377803	Stat1/Cxcl10/Cxcl9	3	BP
GO:0071276	cellular response to cadmium ion	0.030377803	Egfr/Ncf1/Mmp9	3	BP
GO:1903963	arachidonate transport	0.030377803	Pla2g5/Anxa1/Pla2g2a	3	BP
GO:0001781	neutrophil apoptotic process	0.030403208	Anxa1/Ifng	2	BP
GO:0002281	macrophage activation involved in immune response	0.030403208	Lbp/Sbno2	2	BP
GO:0002566	somatic diversification of immune receptors via somatic mutation	0.030403208	Adar/Samhd1	2	BP
GO:0006924	activation-induced cell death of T cells	0.030403208	Ceacam1/Ripk3	2	BP

GO:0007213	G protein-coupled acetylcholine receptor signaling pathway	0.030403208	Agrn/Cdk5r1	2	BP
GO:0009151	purine deoxyribonu-cleotide metabolic process	0.030403208	Samhd1/Ada	2	BP
GO:0021548	pons development	0.030403208	Slc4a7/Cdk5r1	2	BP
GO:0032306	regulation of prosta-glandin secretion	0.030403208	Il1a/Il1b	2	BP
GO:0035745	T-helper 2 cell cyto-kine production	0.030403208	Rsad2/Nlrp3	2	BP
GO:0060100	positive regulation of phagocytosis, engulf-ment	0.030403208	Lbp/C3	2	BP
GO:0060670	branching involved in labyrinthine layer morphogenesis	0.030403208	Socs3/Il10	2	BP
GO:0070269	pyroptosis	0.030403208	Casp4/Gsdmd	2	BP
GO:0070986	left/right axis specifi-cation	0.030403208	Mns1/Dll1	2	BP
GO:0072567	chemokine (C-X-C motif) ligand 2 pro-duction	0.030403208	Cd74/Tnf	2	BP
GO:0090331	negative regulation of platelet aggregation	0.030403208	Abat/Ceacam1	2	BP
GO:1900121	negative regulation of receptor binding	0.030403208	Il10/B2m	2	BP
GO:1905155	positive regulation of membrane invagina-tion	0.030403208	Lbp/C3	2	BP
GO:0046890	regulation of lipid biosynthetic process	0.030832927	Anxa1/C3/Bmp2/Ceacam1/Il1a/Tnf/Il1b/Ifng	8	BP
GO:0002244	hematopoietic pro-genitor cell differen-tiation	0.030889961	Apo-bec3/Cebpd/Batf/Plek/Adar/Krt75/Eif2ak2	7	BP
GO:0051928	positive regulation of calcium ion transport	0.030889961	Cxcr3/Ccl4/Ccl3/Cx3cl1/Cxcl10/Jak3/Cxcl9	7	BP
GO:0007187	G protein-coupled receptor signaling pathway, coupled to cyclic nucleotide second messenger	0.031627753	Cxcr3/Grm4/Gnal/Anxa1/Nos2/Ptger1/Htr1f/Gnat2/Cxcl10/Cxcl9	10	BP
GO:0048145	regulation of fibroblast proliferation	0.03188648	Cd74/Egfr/Ereg/Pmaip1/Mmp9/Ifng	6	BP
GO:0034101	erythrocyte homeosta-sis	0.031932755	Stat1/Isg15/Adar/B2m/Mafb/Zfp36/Jak3	7	BP
GO:0030168	platelet activation	0.03249179	Abat/Gp5/Plek/Ceacam1/Cx3cl1	5	BP
GO:0006775	fat-soluble vitamin metabolic process	0.032901012	Tnf/Il1b/Ifng	3	BP

GO:0045920	negative regulation of exocytosis	0.032901012	Anxa1/Lgals9/Ceacam1	3	BP
GO:0060969	negative regulation of gene silencing	0.032901012	Adar/Apobec1/Zc3h12a	3	BP
GO:0090322	regulation of superoxide metabolic process	0.032901012	Egfr/Tnf/Cxcl1	3	BP
GO:1902110	positive regulation of mitochondrial membrane permeability involved in apoptotic process	0.032901012	Stpg1/Pmaip1/Bid	3	BP
GO:0097553	calcium ion transmembrane import into cytosol	0.032998871	Cxcr3/Ccr5/Ccl3/Cx3cl1/Trpm2/Cxcl10/Cxcl9	7	BP
GO:2000181	negative regulation of blood vessel morphogenesis	0.033110329	Cxcr3/Sema4a/Il17f/Stat1/Hhex/Cxcl10	6	BP
GO:0006814	sodium ion transport	0.033350739	Slc9a5/Slc4a5/Slc4a7/Fxyd4/Trpm2/Agrn/Atp1a3/Atp1b4/Cxcl1	9	BP
GO:0035306	positive regulation of dephosphorylation	0.033386539	Bmp2/Plek/Ripk3/Ifng	4	BP
GO:0019216	regulation of lipid metabolic process	0.034306672	Anxa1/Socs1/C3/Bmp2/Ceacam1/Il1a/Tnf/Socs6/Apobec1/Irs1/Il1b/Ifng/Nod2	13	BP
GO:0002923	regulation of humoral immune response mediated by circulating immunoglobulin	0.03497759	Tnf/Nod2	2	BP
GO:0006828	manganese ion transport	0.03497759	Slc11a1/Trpm2	2	BP
GO:0007379	segment specification	0.03497759	Dll1/Mafb	2	BP
GO:0021984	adenohypophysis development	0.03497759	Bmp2/Duox2	2	BP
GO:0034116	positive regulation of heterotypic cell-cell adhesion	0.03497759	Tnf/Il1b	2	BP
GO:0036376	sodium ion export across plasma membrane	0.03497759	Agrn/Atp1a3	2	BP
GO:0042402	cellular biogenic amine catabolic process	0.03497759	Smox/Ido1	2	BP
GO:0045647	negative regulation of erythrocyte differentiation	0.03497759	Mafb/Zfp36	2	BP
GO:0045837	negative regulation of membrane potential	0.03497759	Pmaip1/Il1rn	2	BP
GO:0048308	organelle inheritance	0.03497759	Stk25/Plk3	2	BP
GO:0048313	Golgi inheritance	0.03497759	Stk25/Plk3	2	BP
GO:0048537	mucosa-associated lymphoid tissue de-	0.03497759	Ceacam1/Ada	2	BP

	velopment				
GO:0048541	Peyer's patch development	0.03497759	Ceacam1/Ada	2	BP
GO:0051451	myoblast migration	0.03497759	Megf10/Anxa1	2	BP
GO:0070423	nucleotide-binding oligomerization domain containing signaling pathway	0.03497759	Irgm/Nod2	2	BP
GO:0070431	nucleotide-binding oligomerization domain containing 2 signaling pathway	0.03497759	Irgm/Nod2	2	BP
GO:0097011	cellular response to granulocyte macrophage colony-stimulating factor stimulus	0.03497759	Etv3/Zfp36	2	BP
GO:0097012	response to granulocyte macrophage colony-stimulating factor	0.03497759	Etv3/Zfp36	2	BP
GO:0099159	regulation of modification of postsynaptic structure	0.03497759	Tiam1/Marcks	2	BP
GO:1900119	positive regulation of execution phase of apoptosis	0.03497759	Cxcr3/Zc3h12a	2	BP
GO:1902931	negative regulation of alcohol biosynthetic process	0.03497759	Bmp2/Plek	2	BP
GO:2000252	negative regulation of feeding behavior	0.03497759	Cck/Napepld	2	BP
GO:0043550	regulation of lipid kinase activity	0.035276938	Socs1/Socs6/Irs1/Nod2	4	BP
GO:1903902	positive regulation of viral life cycle	0.035276938	Cd74/Tmprss2/Lgals9/Adar	4	BP
GO:0010608	posttranscriptional regulation of gene expression	0.035458581	Parp9/Scgb1a1/Slc11a1/Egfr/Plekhn1/Igf2bp1/Adar/Noct/Zfp36/Tnf/Apobec1/Eif2ak2/Zc3h12a/Bcl3/Zc3h12d/Mael	16	BP
GO:0014015	positive regulation of gliogenesis	0.035482667	Tlr2/Tiam1/Bmp2/Tnf/Il1b	5	BP
GO:0048524	positive regulation of viral process	0.035482667	Cd74/Tmprss2/Lgals9/Ceacam1/Adar	5	BP
GO:0017085	response to insecticide	0.035529963	Mapk13/Zc3h12a/Nr4a2	3	BP
GO:0032743	positive regulation of interleukin-2 production	0.035529963	Anxa1/Il1a/Il1b	3	BP
GO:0043243	positive regulation of protein-containing complex disassembly	0.035529963	Plek/Tnf/Irgm	3	BP
GO:0030282	bone mineralization	0.035648471	Mmp13/Bmp2/Isg15/Ccl3/Duox2/Sbno2	6	BP

GO:0048144	fibroblast proliferation	0.035648471	Cd74/Egfr/Ereg/Pmaip1/Mmp9/Ifng	6	BP
GO:0030900	forebrain development	0.03611906	Dnajb1/Ptchd1/Egfr/Igf2bp1/Bmp2/Ccr5/Duox2/Chrd/Apaf1/Mfsd2a/Hhex/Atp1a3/Kirrel3/Sema7a/Anxa3/Cdk5r1/Nr4a2	17	BP
GO:0002275	myeloid cell activation involved in immune response	0.037039729	Lbp/Lgals9/Ccl3/Sbno2/Anxa3	5	BP
GO:0010827	regulation of glucose transmembrane transport	0.037039729	C3/Hk2/Tnf/Irs1/Il1b	5	BP
GO:0061097	regulation of protein tyrosine kinase activity	0.037039729	Ereg/Ncf1/Unc119/Agrn/Epgn	5	BP
GO:0030968	endoplasmic reticulum unfolded protein response	0.037228164	Casp12/Creb3l3/Eif2ak2/Ifng	4	BP
GO:0045661	regulation of myoblast differentiation	0.037228164	Dll1/Tnf/Cxcl10/Cxcl9	4	BP
GO:0002701	negative regulation of production of molecular mediator of immune response	0.038263898	Il10/Tnf/Jak3	3	BP
GO:0046596	regulation of viral entry into host cell	0.038263898	Cd74/Tmprss2/Lgals9	3	BP
GO:1902686	mitochondrial outer membrane permeabilization involved in programmed cell death	0.038263898	Stpg1/Pmaip1/Bid	3	BP
GO:0030279	negative regulation of ossification	0.038638145	Ccl3/Noct/Chrd/Tnf/Areg	5	BP
GO:0002381	immunoglobulin production involved in immunoglobulin mediated immune response	0.039240313	Batf/Pou2f2/Ifng/Nod2	4	BP
GO:0031047	gene silencing by RNA	0.039684731	Egfr/Znfx1/Adar/Zfp36/Zc3h12a/Mael	6	BP
GO:0002863	positive regulation of inflammatory response to antigenic stimulus	0.039799056	C3/Tnf	2	BP
GO:0009310	amine catabolic process	0.039799056	Smox/Ido1	2	BP
GO:0010421	hydrogen peroxide-mediated programmed cell death	0.039799056	Hk3/Stk25	2	BP
GO:0035563	positive regulation of chromatin binding	0.039799056	Parp9/Dtx3l	2	BP
GO:0035872	nucleotide-binding domain, leucine rich repeat containing receptor signaling	0.039799056	Irgm/Nod2	2	BP

	pathway				
GO:0042953	lipoprotein transport	0.039799056	Unc119/Apobec1	2	BP
GO:0044872	lipoprotein localization	0.039799056	Unc119/Apobec1	2	BP
GO:0061029	eyelid development in camera-type eye	0.039799056	Egfr/Grhl3	2	BP
GO:0072672	neutrophil extravasation	0.039799056	Il1a/Trem3	2	BP
GO:0097468	programmed cell death in response to reactive oxygen species	0.039799056	Hk3/Stk25	2	BP
GO:1900016	negative regulation of cytokine production involved in inflammatory response	0.039799056	Zc3h12a/Mefv	2	BP
GO:0060047	heart contraction	0.039917694	Nos2/Tnnt2/Agrn/Tnni3/Ada/Atp1a3/Edn3/Zc3h12a/Ifng	9	BP
GO:0045833	negative regulation of lipid metabolic process	0.040278091	Bmp2/Ceacam1/Tnf/Apobec1/Il1b	5	BP
GO:1900407	regulation of cellular response to oxidative stress	0.040278091	Nox1/Il10/Hk3/Trpm2/Tnf	5	BP
GO:0000187	activation of MAPK activity	0.041091938	Cd74/Grm4/Bmp2/Epgn/Tnf/Il1b	6	BP
GO:0014047	glutamate secretion	0.041101935	Il1rn/Il1b/Cck	3	BP
GO:0033032	regulation of myeloid cell apoptotic process	0.041101935	Anxa1/Ccr5/Nod2	3	BP
GO:0045601	regulation of endothelial cell differentiation	0.041101935	Ceacam1/Tnf/Il1b	3	BP
GO:0045954	positive regulation of natural killer cell mediated cytotoxicity	0.041101935	Lag3/Klrk1/Il21	3	BP
GO:0048147	negative regulation of fibroblast proliferation	0.041101935	Pmaip1/Mmp9/Ifng	3	BP
GO:0060441	epithelial tube branching involved in lung morphogenesis	0.041101935	Tnf/Hhex/Lama1	3	BP
GO:1901099	negative regulation of signal transduction in absence of ligand	0.041101935	Cx3cl1/Tnf/Il1b	3	BP
GO:2001240	negative regulation of extrinsic apoptotic signaling pathway in absence of ligand	0.041101935	Cx3cl1/Tnf/Il1b	3	BP
GO:1901615	organic hydroxy compound metabolic process	0.041171903	Abat/Ly6e/Bmp2/Plek/Duox2/Duoxa2/Duox1/Tnf/Slco4a1/Moxd2/Slc5a3/Il1b/Ifng/Napepld/Nr4a2/Dao	16	BP
GO:0002712	regulation of B cell mediated immunity	0.041313432	C3/Tnf/Ifng/Nod2	4	BP

GO:0002889	regulation of immuno-globulin mediated immune response	0.041313432	C3/Tnf/Ifng/Nod2	4	BP
GO:2001238	positive regulation of extrinsic apoptotic signaling pathway	0.041313432	Pmaip1/Bid/Runx3/Jak3	4	BP
GO:0009895	negative regulation of catabolic process	0.042349394	Slc11a1/Egfr/Psmf1/Nos2/Igf2bp1/Il10/Noct/Zfp36/Tnf/Apobec1/Il1b	11	BP
GO:0030100	regulation of endocy-tosis	0.042349394	Slc11a1/Tlr2/Lbp/C3/Dll1/C2/B2m/Unc119/Tnf/Il1b/Nod2	11	BP
GO:0044070	regulation of anion transport	0.042530302	Abat/Il1a/Tnf/Il1rn/Il1b/Cck	6	BP
GO:0071260	cellular response to mechanical stimulus	0.042530302	Egfr/Bmp2/Irf1/Mmp7/Tnfrsf8/Il1b	6	BP
GO:0050673	epithelial cell prolifer-ation	0.043237796	Cxcr3/Egfr/Stat1/Ereg/Nos2/Il10/Bmp2/Ceacam1/Runx3/Zfp36/Epgn/Tnf/Ifng/Areg/Nod2	15	BP
GO:0008016	regulation of heart contraction	0.043253187	Nos2/Tnnt2/Agrn/Tnni3/Ada/Edn3/Zc3h12a/Ifng	8	BP
GO:0000768	syncytium formation by plasma membrane fusion	0.043447525	Stat1/Sbno2/Cxcl10/Cxcl9	4	BP
GO:0070059	intrinsic apoptotic signaling pathway in response to endoplas-mic reticulum stress	0.043447525	Casp12/Casp4/Pmaip1/Apaf1	4	BP
GO:0140253	cell-cell fusion	0.043447525	Stat1/Sbno2/Cxcl10/Cxcl9	4	BP
GO:1905207	regulation of cardio-cyte differentiation	0.043447525	Egfr/Dll1/Bmp2/Tbx5	4	BP
GO:0048015	phosphatidylinositol-mediated signaling	0.04367091	Egfr/Csf3/Ceacam1/Ncf1/Tnf/Osm/Irs1	7	BP
GO:0051084	'de novo' posttransla-tional protein folding	0.044043086	Dnajb1/Cd74/RT1-DMb	3	BP
GO:0009642	response to light inten-sity	0.044855019	Gnat2/Mmp9	2	BP
GO:0010960	magnesium ion home-ostasis	0.044855019	Egfr/Edn3	2	BP
GO:0016540	protein autoprocessing	0.044855019	Tmprss2/Casp12	2	BP
GO:0019886	antigen processing and presentation of exoge-nous peptide antigen via MHC class II	0.044855019	Cd74/RT1-DMb	2	BP
GO:0030730	sequestering of tri-glyceride	0.044855019	Tnf/Il1b	2	BP
GO:0032305	positive regulation of icosanoid secretion	0.044855019	Il1a/Il1b	2	BP
GO:0032717	negative regulation of interleukin-8 produc-tion	0.044855019	Anxa1/Bcl3	2	BP
GO:0033591	response to L-ascorbic	0.044855019	Il1a/Il1b	2	BP

	acid				
GO:0035461	vitamin transmembrane transport	0.044855019	Slc19a3/Slc46a1	2	BP
GO:0042416	dopamine biosynthetic process	0.044855019	Nr4a2/Dao	2	BP
GO:0042921	glucocorticoid receptor signaling pathway	0.044855019	Cry1/Arntl	2	BP
GO:0044406	adhesion of symbiont to host	0.044855019	Gbp4/Gbp2	2	BP
GO:0048711	positive regulation of astrocyte differentiation	0.044855019	Bmp2/Il1b	2	BP
GO:0060099	regulation of phagocytosis, engulfment	0.044855019	Lbp/C3	2	BP
GO:0019233	sensory perception of pain	0.044979076	Fcgr3a/Il10/Il1a/Ccl3/Tnf/Il1rn/Cck	7	BP
GO:0062013	positive regulation of small molecule metabolic process	0.044979076	Anxa1/Nos2/Pmaip1/Tnf/Irs1/Il1b/Ifng	7	BP
GO:0035195	gene silencing by miRNA	0.045448567	Egfr/Adar/Zfp36/Zc3h12a/Mael	5	BP
GO:1901570	fatty acid derivative biosynthetic process	0.045642552	Cd74/Pla2g5/Anxa1/Cyp4a3	4	BP
GO:1903793	positive regulation of anion transport	0.045642552	Abat/Il1a/Il1b/Cck	4	BP
GO:0048017	inositol lipid-mediated signaling	0.046312192	Egfr/Csf3/Ceacam1/Ncf1/Tnf/Osm/Irs1	7	BP
GO:0051480	regulation of cytosolic calcium ion concentration	0.046885604	Cxcr3/Ptger1/Ccr5/Ccl3/Gnat2/Cx3cl1/Trpm2/Cxcl3/Il1b/Ccrl2/Cxcl10/Cxcl1/Jak3/Cxcl9	14	BP
GO:0008286	insulin receptor signaling pathway	0.047033697	Socs1/Marcks/Socs3/Trim72/Irs1/Il1b	6	BP
GO:0006458	'de novo' protein folding	0.047086253	Dnajb1/Cd74/RT1-DMb	3	BP
GO:0035794	positive regulation of mitochondrial membrane permeability	0.047086253	Stpg1/Pmaip1/Bid	3	BP
GO:0048009	insulin-like growth factor receptor signaling pathway	0.047086253	Bmp2/Trim72/Irs1	3	BP
GO:0050856	regulation of T cell receptor signaling pathway	0.047086253	Ceacam1/Ada/Lilrb4	3	BP
GO:0070670	response to interleukin-4	0.047086253	Parp14/Il1rn/Jak3	3	BP
GO:0048644	muscle organ morphogenesis	0.047255988	Ly6e/Smtnl1/Bmp2/Tnnt2/Tnni3	5	BP
GO:0061138	morphogenesis of a branching epithelium	0.047311262	Socs3/Il10/Bmp2/Hoxd13/Hoxb7/Tnf/Hhex/Lama1/Areg	9	BP

GO:0006949	syncytium formation	0.047898428	Stat1/Sbno2/Cxcl10/Cxcl9	4	BP
GO:0003015	heart process	0.048436895	Nos2/Tnnt2/Agrn/Tnni3/Ada/Atp1a3/Edn3/Zc3h12a/Ifng	9	BP
GO:0051952	regulation of amine transport	0.048597995	Abat/Syt8/Tnf/Il1rn/Il1b/Cck	6	BP
GO:1901136	carbohydrate derivative catabolic process	0.048597995	Apobec3/Pde7a/Samhd1/Apobec1/Ada/Pde9a	6	BP
GO:0018105	peptidyl-serine phosphorylation	0.048904998	Egfr/Csf3/Smtnl1/Tnf/Mapk13/Sgk2/Osm/Irgm/Ifng/Cdk5r1/MAST1	11	BP
GO:0006937	regulation of muscle contraction	0.049053706	Abat/Tnnt2/Ncf1/Agrn/Tnni3/Ada/Zc3h12a	7	BP
GO:0045834	positive regulation of lipid metabolic process	0.049053706	Anxa1/Il1a/Tnf/Irs1/Il1b/Ifng/Nod2	7	BP
GO:0042100	B cell proliferation	0.049105513	Cd74/Il10/Prdm1/Il21/Ada	5	BP
GO:0046889	positive regulation of lipid biosynthetic process	0.049105513	Anxa1/Il1a/Tnf/Il1b/Ifng	5	BP
GO:0097237	cellular response to toxic substance	0.049894665	Anxa1/Il10/Hk3/Duox2/Stk25/Duox1/Trpm2/Tnf/Mapk13/Zc3h12a/Il18bp	11	BP
GO:0042612	MHC class I protein complex	4.80049E-10	RT1-S3/RT1-CE4/B2m/RT1-A1/RT1-CE10/RT1-CE5/RT1-A2	7	CC
GO:0042611	MHC protein complex	1.47843E-09	Cd74/RT1-S3/RT1-CE4/B2m/RT1-DMb/RT1-A1/RT1-CE10/RT1-CE5/RT1-A2	9	CC
GO:0009897	external side of plasma membrane	1.7784E-08	Cxcr3/Cd74/Lag3/Anxa1/Tlr2/Pdcd1/Fcgr3a/Clec2d/Csf3r/Il17a/Ccr5/Ceacam1/Klrk1/Btnl7/Cd80/B2m/Tnf/RT1-A2/Ada/Il12rb1/Btnl5/Cd86/Ifng/Ccrl2/Sema7a/Cxcl10/Cxcl9	27	CC
GO:0043020	NADPH oxidase complex	3.464E-05	Nox1/Ncf1/Ncf4/Noxo1	4	CC
GO:0036464	cytoplasmic ribonucleoprotein granule	0.000288115	Apobec3/Socs1/Marcks/Igf2bp1/Dync1i1/Noct/Arntl/Zfp36/Zc3h12a/Hoxd10/Zc3h12d/Nfkbiz/Mael	13	CC
GO:0035770	ribonucleoprotein granule	0.000550185	Apobec3/Socs1/Marcks/Igf2bp1/Dync1i1/Noct/Arntl/Zfp36/Zc3h12a/Hoxd10/Zc3h12d/Nfkbiz/Mael	13	CC
GO:0001891	phagocytic cup	0.001392849	Megf10/Anxa1/Tnf/Irgm	4	CC
GO:0005890	sodium:potassium-exchanging ATPase complex	0.001693829	Fxyd4/Atp1a3/Atp1b4	3	CC
GO:0090533	cation-transporting ATPase complex	0.003345032	Fxyd4/Atp1a3/Atp1b4	3	CC
GO:0033391	chromatoid body	0.004054198	Marcks/Arntl/Mael	3	CC
GO:0043230	extracellular organelle	0.00479947	Anxa1/Gbp4/Ppfia3/Gbp2/Cd86/Il1b/Xpnpep2	7	CC
GO:0005764	lysosome	0.005397038	Cd74/Prss16/Slc11a1/Anxa1/Mmp13/Marcks	17	CC

			/Gzmb/Dtx3l/Litaf/Slc15a3/RT1-DMb/Trpm2/Zc3hav1/Ada/Rnf19b/Irgm/Il1b		
GO:0000323	lytic vacuole	0.00552675	Cd74/Prss16/Slc11a1/Anxa1/Mmp13/Marcks/Gzmb/Dtx3l/Litaf/Slc15a3/RT1-DMb/Trpm2/Zc3hav1/Ada/Rnf19b/Irgm/Il1b	17	CC
GO:0098533	ATPase dependent transmembrane transport complex	0.00572908	Fxyd4/Atp1a3/Atp1b4	3	CC
GO:0098802	plasma membrane signaling receptor complex	0.013890266	Itgad/Tlr2/Tlr1/Bmp2/Ceacam1/B2m/Tlr10/Il12rb1/Irs1	9	CC
GO:0030670	phagocytic vesicle membrane	0.014511834	Slc11a1/Irgm/Anxa3	3	CC
GO:0019774	proteasome core complex, beta-subunit complex	0.017291292	Psmb9/Psmb8	2	CC
GO:0030430	host cell cytoplasm	0.017291292	Gbp4/Gbp2	2	CC
GO:0033655	host cell cytoplasm part	0.017291292	Gbp4/Gbp2	2	CC
GO:0000932	P-body	0.018743314	Apobec3/Noct/Zfp36/Zc3h12a/Zc3h12d	5	CC
GO:0016324	apical plasma membrane	0.024194363	Mip/Cfap126/Egfr/Anxa1/Dll1/S100g/Slc4a7/Ceacam1/Duox2/Duox1/Slc46a1/Tcirg1/Slc7a9	13	CC
GO:0061702	inflammasome complex	0.024677764	Nlrp3/Gsdmd	2	CC
GO:0033646	host intracellular part	0.028770393	Gbp4/Gbp2	2	CC
GO:0043656	host intracellular region	0.028770393	Gbp4/Gbp2	2	CC
GO:0042613	MHC class II protein complex	0.033112542	Cd74/RT1-DMb	2	CC
GO:0033643	host cell part	0.037692159	Gbp4/Gbp2	2	CC
GO:0043235	receptor complex	0.039564754	Cd74/Itgad/Egfr/Tlr2/Tlr1/Bmp2/Csf3r/Ceacam1/B2m/Tlr10/Gabrq/Gpr84/Il12rb1/Irs1	14	CC
GO:0031983	vesicle lumen	0.042497582	Egfr/Ada	2	CC
GO:1903561	extracellular vesicle	0.04251548	Anxa1/Ppfia3/Cd86/Il1b/Xpnpep2	5	CC
GO:0005125	cytokine activity	1.41849E-15	Il17f/Ccl4/Csf3/Sectm1b/Il10/Sectm1a/Bmp2/Il17a/Il1a/Thpo/Ccl3/Cx3cl1/Tnf/Cxcl3/Cxcl16/Il21/Il1rn/Osm/Tnfsf9/Cxcl6/Il1b/Ifng/Il17c/Cxcl10/Areg/Cxcl1/Cxcl9	27	MF
GO:0042605	peptide antigen binding	5.28788E-12	Tap1/RT1-S3/Tap2/RT1-N2/RT1-A1/RT1-CE10/RT1-CE5/RT1-A2/RT1-N3/Slc7a9/RT1-T24-3/RT1-CE16	12	MF
GO:0030545	receptor regulator activity	2.59468E-10	Sema4a/Il17f/Ereg/Ly6e/Ccl4/Csf3/Sectm1b/Il10/Sectm1a/Bmp2/Il17a/Il1a/Thpo/Ccl3/Cx3cl1/Agrn/Epgn/Tnf/Cxcl3/Cxcl16/Il21/Il1rn/Osm/Tnfsf9/Cxcl6/Edn3/Il1b/Ifng/Il17c/Cck/Sema7a/Cxcl10/Areg/Cxcl1/Cxcl9	35	MF
GO:0003725	double-stranded RNA	2.95572E-10	Ddx58/Oasl/Oasl2/Adar/Oas1e/Oas1k/Oas1a/Oas3/Oas1b/Eif2ak2/Oas2/Dhx58/Oas1i/Ifih	15	MF

	binding		1/Oas1h		
GO:0048018	receptor ligand activity	5.72278E-10	Sema4a/Il17f/Ereg/Ccl4/Csf3/Sectm1b/Il10/Sectm1a/Bmp2/Il17a/Il1a/Thpo/Ccl3/Cx3cl1/Epgn/Tnf/Cxcl3/Cxcl16/Il21/Il1rn/Osm/Tnfsf9/Cxcl6/Edn3/Il1b/Ifng/Il17c/Cck/Sema7a/Cxcl10/Areg/Cxcl1/Cxcl9	33	MF
GO:0005126	cytokine receptor binding	6.553E-09	Il17f/Stat1/Ccl4/Csf3/Il10/Bmp2/Ceacam1/Il1a/Ccl3/Cx3cl1/Tnf/Cxcl3/Cxcl16/Il21/Il12rb1/Il1rn/Osm/Tnfsf9/Cxcl6/Il1b/Ifng/Ccrl2/Cxcl10/Cxcl1/Cxcl9	25	MF
GO:0042379	chemokine receptor binding	3.51924E-08	Stat1/Ccl4/Ccl3/Cx3cl1/Cxcl3/Cxcl16/Cxcl6/Ccrl2/Cxcl10/Cxcl1/Cxcl9	11	MF
GO:0003823	antigen binding	4.70223E-08	Tap1/RT1-S3/Tap2/RT1-N2/RT1-A1/RT1-CE10/RT1-CE5/RT1-A2/RT1-N3/Slc7a9/RT1-T24-3/RT1-CE16	12	MF
GO:0008009	chemokine activity	7.49092E-08	Ccl4/Ccl3/Cx3cl1/Cxcl3/Cxcl16/Cxcl6/Cxcl10/Cxcl1/Cxcl9	9	MF
GO:0001664	G protein-coupled receptor binding	1.753E-05	Gnal/Stat1/Ccl4/C3/Ptger1/Ccl3/Gnat2/Cx3cl1/Cxcl3/Cxcl16/Atp1a3/Clic6/Cxcl6/Edn3/Ccrl2/Rtp4/Cxcl10/Cxcl1/Cxcl9	19	MF
GO:0070566	adenylyltransferase activity	3.25156E-05	Oasl2/Oas1a/Oas3/Oas1b/Oas2/Oas1i	6	MF
GO:0019955	cytokine binding	3.55535E-05	Cxcr3/Cd74/Il17f/Csf3r/Ccr5/Zfp36/Agrn/Il12rb1/Il1rn/Il10ra/Ccrl2/Il18bp	12	MF
GO:0042277	peptide binding	4.6566E-05	Cd74/Tap1/RT1-S3/Tlr2/Tap2/Lbp/Tlr1/RT1-N2/Tlr10/RT1-A1/RT1-CE10/RT1-CE5/RT1-A2/Atp1a3/RT1-N3/Slc7a9/RT1-T24-3/Nod2/RT1-CE16	19	MF
GO:0042287	MHC protein binding	4.8573E-05	Cd74/Tap1/RT1-S3/Lag3/Tap2/Klrk1	6	MF
GO:0019239	deaminase activity	0.000118163	Apobec3/Adar/Zbp1/Apobec1/Ada	5	MF
GO:0035325	Toll-like receptor binding	0.000137397	Tlr2/Tlr1/Ceacam1/Tlr10	4	MF
GO:0050664	oxidoreductase activity, acting on NAD(P)H, oxygen as acceptor	0.000189048	Nox1/Duox2/Ncf1/Duox1	4	MF
GO:0016814	hydrolase activity, acting on carbon-nitrogen (but not peptide) bonds, in cyclic amidines	0.000218874	Apobec3/Adar/Zbp1/Apobec1/Ada	5	MF
GO:0033218	amide binding	0.000227325	Cd74/Tap1/RT1-S3/Tlr2/Tap2/Lbp/Tlr1/RT1-N2/Tlr10/RT1-A1/RT1-CE10/RT1-CE5/Slc46a1/RT1-A2/Atp1a3/RT1-N3/Slc7a9/RT1-T24-3/Nod2/RT1-CE16	20	MF
GO:0070851	growth factor receptor binding	0.000273484	Ereg/Csf3/Il10/Ceacam1/Il1a/Epgn/Il21/Il12rb1/Il1rn/Il1b/Areg	11	MF
GO:0004896	cytokine receptor activity	0.000537246	Cxcr3/Cd74/Csf3r/Ccr5/Csf2rb/Il12rb1/Il10ra/Ccrl2	8	MF

GO:0042288	MHC class I protein binding	0.000824812	Tap1/RT1-S3/Tap2/Klrk1	4	MF
GO:0003950	NAD+ ADP-ribosyltransferase activity	0.001203078	Parp9/Parp14/Zc3hav1/Parp12	4	MF
GO:0048020	CCR chemokine receptor binding	0.0015182	Stat1/Ccl4/Ccl3/Cx3cl1/Ccrl2	5	MF
GO:0050700	CARD domain binding	0.001968447	Casp4/Irgm/Nod2	3	MF
GO:0055102	lipase inhibitor activity	0.001968447	Scgb1a1/Anxa1/Anxa3	3	MF
GO:0019957	C-C chemokine binding	0.001975785	Cxcr3/Ccr5/Zfp36/Ccrl2	4	MF
GO:0004175	endopeptidase activity	0.002729456	Tmprss2/Adamts15/Mmp13/Gzmb/Casp12/F10/C2/Htra4/LOC102553861/Casp4/Usp18/Adamts4/Mmp7/Psmb9/Gzmbl3/Adam32/Psmb8/Gzmk/Mmp9/Adam18	20	MF
GO:0048306	calcium-dependent protein binding	0.004236845	Grm4/Syt8/Anxa1/Mmp13/Tnni3/Anxa3	6	MF
GO:0003924	GTPase activity	0.004558223	MGC108823/MGC105567/Gnal/Gbp4/Gnat2/Mx2/RGD1309362/Igtp/Ifi47/Gbp2/Rnd3/Irgm/Gbp5/Rem2	14	MF
GO:0015291	secondary active transmembrane transporter activity	0.005558769	Slc9a5/Slc11a1/Slc4a5/Slc16a3/Slco2a1/Slc4a7/Slc15a3/Slc6a14/Mfsd2a/Slco4a1/Atp1a3	11	MF
GO:0019956	chemokine binding	0.005572153	Cxcr3/Ccr5/Zfp36/Ccrl2	4	MF
GO:0005149	interleukin-1 receptor binding	0.005610825	Il1a/Il1rn/Il1b	3	MF
GO:0022804	active transmembrane transporter activity	0.006067783	Tap1/Slc9a5/Slc11a1/Slc4a5/Tap2/Slc16a3/Slco2a1/Slc4a7/Abca13/Slc15a3/Slc6a14/Mfsd2a/Slco4a1/Atp1a3/Tcirg1	15	MF
GO:0070628	proteasome binding	0.006625141	Psmf1/Psmb9/Ubd	3	MF
GO:0001786	phosphatidylserine binding	0.006905581	Syt8/Marcks/Plekhn1/Trim72/Gsdmd	5	MF
GO:0001883	purine nucleoside binding	0.00803242	MGC108823/MGC105567/Gnal/Gbp4/Gnat2/Mx2/RGD1309362/Igtp/Ifi47/Gbp2/Ada/Arl5b/Rnd3/Irgm/Gbp5/Rem2	16	MF
GO:0008236	serine-type peptidase activity	0.008669272	Tmprss2/Prss16/Mmp13/Gzmb/F10/C2/Htra4/LOC102553861/Gzmbl3/Gzmk/Mmp9	11	MF
GO:0001882	nucleoside binding	0.009957356	MGC108823/MGC105567/Gnal/Gbp4/Gnat2/Mx2/RGD1309362/Igtp/Ifi47/Gbp2/Ada/Arl5b/Rnd3/Irgm/Gbp5/Rem2	16	MF
GO:0017171	serine hydrolase activity	0.010172425	Tmprss2/Prss16/Mmp13/Gzmb/F10/C2/Htra4/LOC102553861/Gzmbl3/Gzmk/Mmp9	11	MF
GO:0019001	guanyl nucleotide binding	0.01092265	MGC108823/MGC105567/Gnal/Gbp4/Gnat2/Mx2/RGD1309362/Igtp/Samhd1/Ifi47/Gbp2/Arl5b/Rnd3/Irgm/Gbp5/Rem2	16	MF
GO:0004222	metalloendopeptidase activity	0.010997147	Adamts15/Mmp13/Adamts4/Mmp7/Adam32/Mmp9/Adam18	7	MF

GO:0004252	serine-type endopeptidase activity	0.011963061	Tmprss2/Mmp13/Gzmb/F10/C2/Htra4/LOC102553861/Gzmbl3/Gzmk/Mmp9	10	MF
GO:0001228	DNA-binding transcription activator activity, RNA polymerase II-specific	0.012558434	Elf3/Cebpd/Tfec/Batf/Tbx5/Hoxd13/Arntl/Pou2f2/Mafb/Irf1/Nobox/Hoxb7/Creb3l3/Hoxb5/Hoxd10/Grhl3/Egr2/Nr4a2	18	MF
GO:0008514	organic anion transmembrane transporter activity	0.012766897	Slc4a5/Slc16a3/Slco2a1/Slc4a7/Ceacam1/Mfsd2a/Slco4a1/Slc46a1/Slc25a22/Slc7a9	10	MF
GO:0005539	glycosaminoglycan binding	0.014048031	Adamts15/Pla2g5/Tlr2/Ccl3/Chrd/Mmp7/Agrn/Serpinc1/Cxcl10/Nod2	10	MF
GO:0005525	GTP binding	0.014128731	MGC108823/MGC105567/Gnal/Gbp4/Gnat2/Mx2/RGD1309362/Igtp/Ifi47/Gbp2/Arl5b/Rnd3/Irgm/Gbp5/Rem2	15	MF
GO:0042887	amide transmembrane transporter activity	0.014174769	Tap1/Tap2/Slc15a3/Slc46a1	4	MF
GO:0016493	C-C chemokine receptor activity	0.014921328	Cxcr3/Ccr5/Ccrl2	3	MF
GO:0032550	purine ribonucleoside binding	0.016162051	MGC108823/MGC105567/Gnal/Gbp4/Gnat2/Mx2/RGD1309362/Igtp/Ifi47/Gbp2/Arl5b/Rnd3/Irgm/Gbp5/Rem2	15	MF
GO:0004950	chemokine receptor activity	0.01668663	Cxcr3/Ccr5/Ccrl2	3	MF
GO:0016779	nucleotidyltransferase activity	0.017026618	Oasl2/Oas1k/Oas1a/Oas3/Oas1b/Oas2/Oas1i	7	MF
GO:0032549	ribonucleoside binding	0.017638066	MGC108823/MGC105567/Gnal/Gbp4/Gnat2/Mx2/RGD1309362/Igtp/Ifi47/Gbp2/Arl5b/Rnd3/Irgm/Gbp5/Rem2	15	MF
GO:0016810	hydrolase activity, acting on carbon-nitrogen (but not peptide) bonds	0.017736339	Apobec3/Padi3/Padi1/Adar/Zbp1/Apobec1/Ada	7	MF
GO:0001637	G protein-coupled chemoattractant receptor activity	0.018563769	Cxcr3/Ccr5/Ccrl2	3	MF
GO:0043539	protein serine/threonine kinase activator activity	0.018563769	Lgals9/Irgm/Cdk5r1	3	MF
GO:0050780	dopamine receptor binding	0.018563769	Ptger1/Atp1a3/Clic6	3	MF
GO:0072341	modified amino acid binding	0.018743063	Syt8/Marcks/Plekhn1/Trim72/Slc46a1/Gsdmd	6	MF
GO:0008028	monocarboxylic acid transmembrane transporter activity	0.01914593	Slc16a3/Slco2a1/Ceacam1/Mfsd2a	4	MF
GO:0016763	transferase activity, transferring pentosyl groups	0.020536707	Parp9/Parp14/Zc3hav1/Parp12	4	MF
GO:0005543	phospholipid binding	0.021558356	Pla2g5/Syt8/Anxa1/Tiam1/Marcks/Plekhn1/F10/Plek/Ncf1/Ncf4/Trim72/Noxo1/Gsdmd/Mitd1/Pla2g2a/Anxa3	16	MF

GO:0032561	guanyl ribonucleotide binding	0.022231245	MGC108823/MGC105567/Gnal/Gbp4/Gnat2/Mx2/RGD1309362/Igtp/Ifi47/Gbp2/Arl5b/Rnd3/Irgm/Gbp5/Rem2	15	MF
GO:0009881	photoreceptor activity	0.023007962	Cry1/Opn3	2	MF
GO:0005044	scavenger receptor activity	0.023500485	Tmprss2/Megf10/Lgals3bp/Cxcl16	4	MF
GO:0017091	AU-rich element binding	0.0248695	Zfp36/Apobec1/Zc3h12a	3	MF
GO:1904680	peptide transmembrane transporter activity	0.027196295	Tap1/Tap2/Slc15a3	3	MF
GO:0005452	inorganic anion exchanger activity	0.027215865	Slc4a5/Slc4a7	2	MF
GO:0005536	glucose binding	0.027215865	Hk2/Hk3	2	MF
GO:0016813	hydrolase activity, acting on carbon-nitrogen (but not peptide) bonds, in linear amidines	0.027215865	Padi3/Padi1	2	MF
GO:0046703	natural killer cell lectin-like receptor binding	0.027215865	RT1-S3/Clec2d	2	MF
GO:0046935	1-phosphatidylinositol-3-kinase regulator activity	0.027215865	Socs1/Socs6	2	MF
GO:0072349	modified amino acid transmembrane transporter activity	0.027215865	Slc46a1/Slc7a9	2	MF
GO:1901612	cardiolipin binding	0.027215865	Plekhn1/Gsdmd	2	MF
GO:0047498	calcium-dependent phospholipase A2 activity	0.031706429	Pla2g5/Pla2g2a	2	MF
GO:0097153	cysteine-type endopeptidase activity involved in apoptotic process	0.031706429	Casp12/Casp4	2	MF
GO:0043394	proteoglycan binding	0.034846889	Chrd/Agrn/Atp1a3	3	MF
GO:0016651	oxidoreductase activity, acting on NAD(P)H	0.035299882	Nos2/Nox1/Duox2/Ncf1/Duox1	5	MF
GO:0005159	insulin-like growth factor receptor binding	0.036465356	Socs1/Irs1	2	MF
GO:0005523	tropomyosin binding	0.036465356	Smtnl1/Tnnt2	2	MF
GO:0031748	D1 dopamine receptor binding	0.036465356	Ptger1/Atp1a3	2	MF
GO:0035925	mRNA 3'-UTR AU-rich region binding	0.036465356	Zfp36/Zc3h12a	2	MF
GO:0042834	peptidoglycan binding	0.036465356	Tlr2/Nod2	2	MF

GO:0005164	tumor necrosis factor receptor binding	0.037618381	Stat1/Tnf/Tnfsf9	3	MF
GO:0008509	anion transmembrane transporter activity	0.038119013	Slc4a5/Clca4/Slc16a3/Slco2a1/Slc4a7/Ceacam1/Mfsd2a/Slco4a1/Slc46a1/Clic6/Slc25a22/Slc7a9	12	MF
GO:0008083	growth factor activity	0.039010067	Ereg/Csf3/Bmp2/Epgn/Osm/Areg/Cxcl1	7	MF
GO:0005342	organic acid trans-membrane transporter activity	0.040254928	Slc16a3/Slco2a1/Ceacam1/Mfsd2a/Slc46a1/Slc25a22/Slc7a9	7	MF
GO:0046943	carboxylic acid trans-membrane transporter activity	0.040254928	Slc16a3/Slco2a1/Ceacam1/Mfsd2a/Slc46a1/Slc25a22/Slc7a9	7	MF
GO:0005154	epidermal growth factor receptor binding	0.040499113	Ereg/Epgn/Areg	3	MF
GO:0030548	acetylcholine receptor regulator activity	0.041478835	Ly6e/Agrn	2	MF
GO:0099602	neurotransmitter re-ceptor regulator activi-ty	0.041478835	Ly6e/Agrn	2	MF
GO:1901611	phosphatidylglycerol binding	0.041478835	Plekhn1/Gsdmd	2	MF
GO:0038024	cargo receptor activity	0.041915634	Tmprss2/Megf10/Lgals3bp/Timd2/Cxcl16	5	MF
GO:0035014	phosphatidylinositol 3-kinase regulator activi-ty	0.046733528	Socs1/Socs6	2	MF
GO:0102567	phospholipase A2 activity (consuming 1,2-dipalmitoylphosphati-dylcholine)	0.046733528	Pla2g5/Pla2g2a	2	MF
GO:0102568	phospholipase A2 activity consuming 1,2-dioleoylphosphatidyl-ethanolamine)	0.046733528	Pla2g5/Pla2g2a	2	MF
GO:0008237	metallopeptidase activity	0.046977677	Adamts15/Mmp13/Adamts4/Mmp7/Adam32/Mmp9/Adam18/Xpnpep2	8	MF
GO:0008201	heparin binding	0.049694554	Adamts15/Pla2g5/Ccl3/Chrd/Mmp7/Serpinc1/Cxcl10	7	MF

ID represents the serial number of GO, Description represents the description information corresponding to GO, GeneID indicates the names of all the enriched genes in this GO term, which are separated by "/",Count indicates the number of all enriched genes on this GO term.

Support Table 8.GO enrichment analysis of 426 DE mRNAs up-regulated by EA.

ID	Description	pvalue	geneID	Count	Type
GO:0009914	hormone transport	4.06598E-11	Nr0b2/Chga/Sstr5/Fzd4/Gcg/Neurod1/Pim3/Ffar2/Aqp1/Nmu/Sfrp1/Gpr119/Pparg/Avpr1a/Lepr/Spink3/Abcb1a/Slc30a8/Ptprn2/Sytl4/Bmp8a/Bmp6/Cckar/Igfbp3/Pex5l/Isl1/Syt7/Adipoq/Selenom/Cftr/Edn2/Agt	32	BP
GO:0046879	hormone secretion	7.0689E-11	Nr0b2/Chga/Sstr5/Fzd4/Gcg/Neurod1/Pim3/Ffar2/Aqp1/Nmu/Sfrp1/Gpr119/Pparg/Avpr1a/Lepr/Spink3/Slc30a8/Ptprn2/Sytl4/Bmp8a/Bmp6/Cckar/Igfbp3/Pex5l/Isl1/Syt7/Adipoq/Selenom/Cftr/Edn2/Agt	31	BP

GO:1903532	positive regulation of secretion by cell	4.10022E-09	Nr0b2/Rab27a/Rab27b/Gcg/Fcna/Ffar2/Tgfb3/Cd34/Nmu/Snca/Avpr1a/Lepr/Spink3/Xbp1/Slc30a8/Chrna4/Ang/Sytl4/Cpb2/Bmp6/Oxtr/Lpl/Cckar/Pex5l/Isl1/Syt7/Slc18a1/Rgcc/Fcer1a/Cftr/Ms4a2/Edn2/Agt	33	BP
GO:0030072	peptide hormone secretion	7.05153E-09	Nr0b2/Chga/Sstr5/Gcg/Neurod1/Pim3/Ffar2/Aqp1/Nmu/Sfrp1/Gpr119/Pparg/Avpr1a/Lepr/Spink3/Slc30a8/Ptprn2/Sytl4/Bmp8a/Cckar/Pex5l/Isl1/Syt7/Cftr	24	BP
GO:0051384	response to glucocorticoid	8.79992E-09	Srd5a1/Sstr5/Ephx1/Sfrp4/Aqp1/Avpr1a/Lepr/Serpinf1/Pck1/Abcb1a/Ugt1a1/Bmp6/Cckar/Isl1/Cps1/Fabp4/Adipoq/Aldob/Cdo1/Cpn1/Agtr1b/Ptgds/Sftpa1/Hmgcs2/Sstr2	25	BP
GO:0031960	response to corticosteroid	9.76554E-09	Srd5a1/Sstr5/Ephx1/Sfrp4/Aqp1/Avpr1a/Lepr/Serpinf1/Pck1/Abcb1a/Ugt1a1/Bmp6/Cckar/Isl1/Cps1/Fabp4/Adipoq/Aldob/Cdo1/Cpn1/Htr1b/Agtr1b/Ptgds/Sftpa1/Hmgcs2/Sstr2	26	BP
GO:0046883	regulation of hormone secretion	1.22945E-08	Nr0b2/Chga/Sstr5/Gcg/Pim3/Ffar2/Nmu/Sfrp1/Pparg/Avpr1a/Lepr/Spink3/Slc30a8/Sytl4/Bmp8a/Bmp6/Cckar/Igfbp3/Pex5l/Isl1/Syt7/Adipoq/Cftr/Edn2/Agt	25	BP
GO:0008015	blood circulation	1.90412E-08	Chga/Adh5/Kcnh2/Ramp2/Pcsk5/Ace2/Kcnj5/Klk1b3/Klk1c9/Tac3/Cd34/Tac4/Nmu/Tacr3/Trdn/Pparg/Avpr1a/Sgcg/Gata4/Oxtr/Fxyd1/Cps1/Kcne3/Cbs/Pde2a/Adipoq/Klk10/Htr1b/Agtr1b/Cftr/Edn2/Agt	32	BP
GO:0015850	organic hydroxy compound transport	1.99535E-08	Chga/Slc16a7/Shh/Egf/Aqp1/Snca/Apob/Xbp1/Chrna4/Bmp6/Oxtr/Npc1l1/Igfbp3/Syt7/Slc18a1/Adipoq/Aqp8/Selenom/Htr1b/Nat8l/Fcer1a/Cftr/Agt	23	BP
GO:0007586	digestion	2.27528E-08	Tff3/Pgc/Capn8/Neurod1/Slc26a7/Tac4/Aqp1/Nmu/Capn9/Chrm1/Spink3/Fabp2/Abcb1a/Oxtr/Cckar/Npc1l1	16	BP
GO:0003013	circulatory system process	2.94925E-08	Chga/Adh5/Kcnh2/Ramp2/Pcsk5/Ace2/Kcnj5/Klk1b3/Klk1c9/Tac3/Cd34/Tac4/Nmu/Tacr3/Trdn/Pparg/Avpr1a/Sgcg/Gata4/Oxtr/Fxyd1/Cps1/Kcne3/Cbs/Pde2a/Adipoq/Klk10/Htr1b/Agtr1b/Cftr/Edn2/Agt	32	BP
GO:0008217	regulation of blood pressure	6.19247E-08	Adh5/Ramp2/Pcsk5/Ace2/Klk1b3/Klk1c9/Tac3/Cd34/Tac4/Nmu/Tacr3/Pparg/Avpr1a/Oxtr/Adipoq/Klk10/Agtr1b/Edn2/Agt	19	BP
GO:0016053	organic acid biosynthetic process	7.48911E-08	Pycr1/Cth/Dhtkd1/Rgn/Ptgis/Crabp2/Fbp1/Thnsl2/Acsm5/Egf/Esrrb/Oat/Avpr1a/Cyp4a1/Lpl/Cbs/Bcat2/Pklr/Aldob/Cdo1/Ltc4s/Gatm/Fcer1a/Ptgds/Edn2/Agt	26	BP
GO:0090276	regulation of peptide hormone secretion	1.24886E-07	Nr0b2/Chga/Sstr5/Gcg/Pim3/Ffar2/Nmu/Sfrp1/Pparg/Avpr1a/Lepr/Spink3/Slc30a8/Sytl4/Bmp8a/Cckar/Pex5l/Isl1/Syt7/Cftr	20	BP
GO:0006631	fatty acid metabolic process	1.78205E-07	Rgn/Ptgis/Cyp2d5/Cyp2d2/Cryl1/Thnsl2/Acsm5/Snca/Pnpla3/Cyp2c24/Pparg/Avpr1a/Ces1d/Pck1/Fabp2/Cyp4a1/Cyp4f1/Lpl/Phyh/Fabp4/Adipoq/Ptgds/Alox12e/Edn2/Gsta1/Agt	26	BP
GO:0046394	carboxylic acid biosynthetic process	2.53904E-07	Pycr1/Cth/Dhtkd1/Rgn/Ptgis/Crabp2/Fbp1/Thnsl2/Acsm5/Egf/Esrrb/Oat/Avpr1a/Cyp4a1/Lpl/Cbs/Bcat2/Pklr/Aldob/Ltc4s/Gatm/Fcer1a/Ptgds/Edn2/Agt	25	BP
GO:0072009	nephron epithelium development	3.7579E-07	Calb1/Shh/Six4/Tcf21/Cd34/Aqp1/Prom1/Wnt2b/Klf15/Adipoq/Agtr1b/Maged1/Agt/Gpc3	14	BP
GO:0001655	urogenital system development	6.23719E-07	Srd5a1/Calb1/Pcsk5/Rgn/Dcn/Shh/Egf/Six4/Tcf21/Tp63/Cd34/Aqp1/Prom1/Sfrp1/Serpinf1/Cyp4a1/Wnt2b/Bmp6/Prlr/Klf15/Adipoq/Agtr1b/Maged1/Hmgcs2/Agt/Gpc3	26	BP
GO:0072001	renal system development	7.15123E-07	Calb1/Pcsk5/Rgn/Dcn/Shh/Egf/Six4/Tcf21/Tp63/Cd34/Aqp1/Prom1/Sfrp1/Serpinf1/Cyp4a1/Wnt2b/Bmp6/Klf15/Adipoq/Agtr1b/Maged1/Hmgcs2/Agt/Gpc3	24	BP

GO:0022600	digestive system process	7.679E-07	Tff3/Neurod1/Slc26a7/Tac4/Aqp1/Nmu/Chrm1/Spink3/Fabp2/Abcb1a/Oxtr/Cckar/Npc1l1	13	BP
GO:0001822	kidney development	8.73384E-07	Calb1/Pcsk5/Rgn/Dcn/Shh/Egf/Six4/Tcf21/Cd34/Aqp1/Prom1/Sfrp1/Serpinf1/Cyp4a1/Wnt2b/Bmp6/Klf15/Adipoq/Agtr1b/Maged1/Hmgcs2/Agt/Gpc3	23	BP
GO:0042737	drug catabolic process	9.67592E-07	Adh5/Aadat/Cyp4b1/Hbb/Cyp2d5/Hba-a1/Aldh1b1/Cyp2d2/Snca/Cyp2c24/Pck1/Fah/Prdx6/Hba-a2/Cpn1	15	BP
GO:0036293	response to decreased oxygen levels	1.39522E-06	Ramp2/Mmp10/Ptgis/Serpina1/Shh/Tgfb3/Cd34/Aqp1/Gpr182/Sfrp1/Clca1/Lepr/Pck1/Tf/Abcb1a/Chrna4/Ang/Oxtr/Igfbp3/Cbs/Pklr/Adipoq/Rgcc/Agtr1b/Cftr/Sftpa1/Arnt2	27	BP
GO:0072073	kidney epithelium development	1.72795E-06	Calb1/Shh/Six4/Tcf21/Cd34/Aqp1/Prom1/Sfrp1/Wnt2b/Klf15/Adipoq/Agtr1b/Maged1/Agt/Gpc3	15	BP
GO:0050878	regulation of body fluid levels	2.10549E-06	Rab27a/Agr2/Shh/Serpina10/Fbln1/Tp63/Pf4/Cd34/Tac4/Aqp1/Thbd/F13a1/Avpr1a/Chrm1/Spink3/Xbp1/Abcb1a/Cpb2/Prlr/Cckar/F2/Bcat2/Cdo1/Cftr	24	BP
GO:0050886	endocrine process	2.14787E-06	Pcsk5/Ace2/Fzd4/Aqp1/Avpr1a/Bmp6/Oxtr/Pex5l/Selenom/Agtr1b/Edn2/Agt	12	BP
GO:0017001	antibiotic catabolic process	2.18614E-06	Adh5/Hbb/Hba-a1/Aldh1b1/Snca/Pck1/Ugt1a1/Prdx6/Hba-a2	9	BP
GO:0050673	epithelial cell proliferation	2.64413E-06	Rgn/Ccl24/Shh/Six4/Fmc1/Tp63/Tgfb3/Cd34/Sfrp1/Pparg/Serpinf1/Spink3/Xbp1/Ccr3/Ang/Cpb2/Bmp6/Igfbp3/Isl1/Tnmd/Klk8/Ccl11/Rgcc/Maged1/Adk/Gpc3	26	BP
GO:0006690	icosanoid metabolic process	3.94477E-06	Ptgis/Cyp2d5/Cyp2d2/Cyp2c24/Avpr1a/Cyp4a1/Cyp4f1/Ltc4s/Fcer1a/Ptgds/Alox12e/Edn2	12	BP
GO:0070482	response to oxygen levels	4.10974E-06	Ramp2/Mmp10/Ptgis/Serpina1/Shh/Tgfb3/Cd34/Aqp1/Gpr182/Sfrp1/Clca1/Pparg/Lepr/Pck1/Tf/Abcb1a/Chrna4/Ang/Oxtr/Igfbp3/Cbs/Pklr/Adipoq/Rgcc/Agtr1b/Cftr/Sftpa1/Arnt2	28	BP
GO:0015669	gas transport	4.14273E-06	Hbb/Hba-a1/Aqp1/Hbb-b1/LOC100134871/Hba-a2	6	BP
GO:0015893	drug transport	4.53052E-06	Hbb/Amn/Slc1a7/Hba-a1/Fzd4/Slc16a12/Aqp1/Hbb-b1/Snca/Abcb1a/Chrna4/Syt7/Slc18a1/LOC100134871/Hba-a2/Htr1b/Nat8l/Agt	18	BP
GO:0090184	positive regulation of kidney development	4.89404E-06	Shh/Six4/Prom1/Wnt2b/Adipoq/Agtr1b/Maged1/Agt	8	BP
GO:0051591	response to cAMP	5.03515E-06	Srd5a1/Aqp1/Thbd/Pck1/Tf/Cps1/Pde2a/Pklr/Adipoq/Aldob/Cdo1/Aqp8/Cftr/Hmgcs2	14	BP
GO:0001666	response to hypoxia	5.4278E-06	Ramp2/Mmp10/Ptgis/Serpina1/Shh/Tgfb3/Cd34/Aqp1/Gpr182/Sfrp1/Clca1/Lepr/Pck1/Tf/Abcb1a/Chrna4/Ang/Igfbp3/Cbs/Pklr/Adipoq/Rgcc/Agtr1b/Sftpa1/Arnt2	25	BP
GO:0030073	insulin secretion	6.62588E-06	Nr0b2/Chga/Sstr5/Gcg/Neurod1/Pim3/Sfrp1/Gpr119/Lepr/Slc30a8/Ptprn2/Sytl4/Bmp8a/Cckar/Isl1/Syt7/Cftr	17	BP
GO:0072006	nephron development	6.84352E-06	Calb1/Shh/Six4/Tcf21/Cd34/Aqp1/Prom1/Wnt2b/Klf15/Adipoq/Agtr1b/Maged1/Agt/Gpc3	14	BP
GO:0045765	regulation of angiogenesis	8.31094E-06	Ramp2/Dcn/Ptgis/Ccl24/Shh/Pf4/Cd34/Aqp1/Pparg/Serpinf1/Xbp1/Mmrn2/Ccr3/Gata4/Isl1/Tnmd/Ccl11/Meox2/Rgcc/Agt	20	BP
GO:0001525	angiogenesis	8.50155E-06	Ramp2/Dcn/Ptgis/Ccl24/Shh/Egf/Tcf21/Pf4/Cd34/Aqp1/Pparg/Lepr/Serpinf1/Xbp1/Mmrn2/Ccr3/Ang/Gata4/Ramp1/Isl1/Tnmd/Ccl11/Meox2/Rgcc/Col23a1/Esm1/Agt	27	BP
GO:0006790	sulfur compound	9.2234E-06	Cth/Aadat/Dcn/Hbb/Chst13/Tst/Sult1c2a/Acsm5/Snca/Gstm5/Ch	19	BP

	metabolic process		st9/Gstm1/Cps1/Cbs/Mgst1/Chst4/Cdo1/Gsta1/Hmgcs2		
GO:0071385	cellular response to glucocorticoid stimulus	9.93565E-06	Srd5a1/Sstr5/Ephx1/Aqp1/Serpinf1/Pck1/Abcb1a/Ugt1a1/Isl1/Agtr1b/Hmgcs2/Sstr2	12	BP
GO:1901568	fatty acid derivative metabolic process	1.06981E-05	Ptgis/Cyp2d5/Cyp2d2/Cyp2c24/Avpr1a/Cyp4a1/Cyp4f1/Ltc4s/Fcer1a/Ptgds/Alox12e/Edn2/Hmgcs2	13	BP
GO:1901342	regulation of vasculature development	1.12441E-05	Cth/Ramp2/Dcn/Ptgis/Ccl24/Shh/Pf4/Cd34/Aqp1/Pparg/Serpinf1/Xbp1/Mmrn2/Ccr3/Gata4/Isl1/Tnmd/Ccl11/Meox2/Rgcc/Agt	21	BP
GO:0006638	neutral lipid metabolic process	1.18095E-05	Rgn/Snca/Pnpla3/Apob/Ces1d/Pck1/Ang/G6pc/Lpl/Cps1/Thrsp/Aadac	12	BP
GO:0045777	positive regulation of blood pressure	1.29055E-05	Adh5/Tac3/Nmu/Tacr3/Avpr1a/Oxtr/Agtr1b/Agt	8	BP
GO:0007188	adenylate cyclase-modulating G protein-coupled receptor signaling pathway	1.30746E-05	Gpr37/Chga/Sstr5/Ramp2/Hrh4/Adgrg2/Mc2r/Gcg/Pf4/Chrm1/Ramp1/Mrap2/Pde2a/Htr1b/Sstr2/Htr5b	16	BP
GO:0072080	nephron tubule development	1.4597E-05	Calb1/Shh/Six4/Tcf21/Aqp1/Prom1/Wnt2b/Agtr1b/Maged1/Agt/Gpc3	11	BP
GO:1990748	cellular detoxification	1.4597E-05	Adh5/Rgn/Hbb/Hba-a1/Gstm5/Gstm1/Prdx6/Mgst1/LOC100134871/Hba-a2/Ltc4s	11	BP
GO:0050678	regulation of epithelial cell proliferation	1.56675E-05	Rgn/Ccl24/Shh/Six4/Fmc1/Tp63/Tgfb3/Sfrp1/Pparg/Serpinf1/Spink3/Xbp1/Ccr3/Ang/Cpb2/Bmp6/Isl1/Tnmd/Ccl11/Rgcc/Maged1/Gpc3	22	BP
GO:0015671	oxygen transport	1.68388E-05	Hbb/Hba-a1/Hbb-b1/LOC100134871/Hba-a2	5	BP
GO:0071384	cellular response to corticosteroid stimulus	1.79054E-05	Srd5a1/Sstr5/Ephx1/Aqp1/Serpinf1/Pck1/Abcb1a/Ugt1a1/Isl1/Agtr1b/Hmgcs2/Sstr2	12	BP
GO:0009410	response to xenobiotic stimulus	1.98502E-05	Srd5a1/Cyp2d5/Cyp2d2/Tac3/Aqp1/Snca/Gstm5/Tacr3/Cyp2c24/Pparg/Lepr/Serpinf1/Pck1/Abcb1a/Ugt1a1/Oxtr/Gstm1/Cps1/Pde2a/Cdo1/Htr1b/Agtr1b/Gsta1	23	BP
GO:0061326	renal tubule development	2.29214E-05	Calb1/Shh/Six4/Tcf21/Aqp1/Prom1/Wnt2b/Agtr1b/Maged1/Agt/Gpc3	11	BP
GO:0071383	cellular response to steroid hormone stimulus	2.3633E-05	Srd5a1/Nr0b2/Sstr5/Ephx1/Tcf21/Tp63/Esrrb/Aqp1/Sfrp1/Pparg/Serpinf1/Pck1/Abcb1a/Ugt1a1/Isl1/Agtr1b/Padi2/Hmgcs2/Sstr2	19	BP
GO:0016051	carbohydrate biosynthetic process	2.42961E-05	Rgn/Chst13/Gcg/Sord/Fbp1/Egf/Esrrb/Snca/B3gnt6/Lepr/Pck1/Chst9/G6pc/Adipoq/B3galt1	15	BP
GO:0015844	monoamine transport	2.7253E-05	Chga/Snca/Xbp1/Chrna4/Oxtr/Syt7/Slc18a1/Htr1b/Nat8l/Fcer1a/Agt	11	BP
GO:0033559	unsaturated fatty acid metabolic process	2.96719E-05	Ptgis/Cyp2d5/Cyp2d2/Cyp2c24/Avpr1a/Cyp4a1/Cyp4f1/Ptgds/Alox12e/Edn2/Gsta1	11	BP
GO:0001990	regulation of systemic arterial blood pressure by hormone	3.01248E-05	Pcsk5/Ace2/Avpr1a/Oxtr/Agtr1b/Edn2/Agt	7	BP
GO:0003073	regulation of systemic arterial	3.2274E-05	Pcsk5/Ace2/Klk1b3/Klk1c9/Tac4/Avpr1a/Oxtr/Klk10/Agtr1b/Ed	11	BP

	blood pressure		n2/Agt		
GO:0046456	icosanoid biosynthetic process	3.53175E-05	Ptgis/Avpr1a/Cyp4a1/Ltc4s/Fcer1a/Ptgds/Edn2	7	BP
GO:0002793	positive regulation of peptide secretion	3.64665E-05	Nr0b2/Gcg/Fcna/Ffar2/Tgfb3/Cd34/Nmu/Lepr/Spink3/Xbp1/Slc30a8/Ang/Sytl4/Lpl/Cckar/Pex5l/Isl1/Rgcc/Cftr/Agt	20	BP
GO:0032355	response to estradiol	3.66576E-05	Srd5a1/Ramp2/Sfrp4/Serpina1/Shh/Sfrp1/Tacr3/Apob/Abcb1a/Ugt1a1/Sstr1/Oxtr/Igfbp3/Hba-a2/Sstr2/Arnt2/Agt	17	BP
GO:0098754	detoxification	3.80735E-05	Adh5/Rgn/Hbb/Hba-a1/Gstm5/Gstm1/Prdx6/Mgst1/LOC100134871/Hba-a2/Ltc4s	11	BP
GO:0071320	cellular response to cAMP	3.84762E-05	Srd5a1/Aqp1/Pck1/Tf/Cps1/Pde2a/Adipoq/Aqp8/Cftr	9	BP
GO:0090183	regulation of kidney development	3.86372E-05	Shh/Six4/Prom1/Wnt2b/Adipoq/Agtr1b/Maged1/Agt	8	BP
GO:0071466	cellular response to xenobiotic stimulus	4.10869E-05	Srd5a1/Cyp2d5/Cyp2d2/Aqp1/Gstm5/Cyp2c24/Serpinf1/Pck1/Abcb1a/Ugt1a1/Gstm1/Pde2a/Cdo1/Agtr1b/Gsta1	15	BP
GO:0010743	regulation of macrophage derived foam cell differentiation	4.24808E-05	Pf4/Apob/Pparg/Lpl/Adipoq	5	BP
GO:0051048	negative regulation of secretion	4.25749E-05	Chga/Egf/Pim3/Fbln1/Cd34/Nmu/Snca/Sfrp1/Pparg/Sytl4/Bmp8a/Oxtr/Igfbp3/Adipoq/Rgcc/Htr1b/Edn2	17	BP
GO:0034764	positive regulation of transmembrane transport	4.73286E-05	Kcnh2/Rgn/Slc9a3r2/Snca/Trdn/Abcb1a/Fxyd1/F2/Kcne3/Klf15/Adipoq/Cftr/Itln1/Lrrc26/Agt/Gpc3	16	BP
GO:0050708	regulation of protein secretion	5.14431E-05	Nr0b2/Chga/Sstr5/Gcg/Fcna/Egf/Pim3/Fbln1/Ffar2/Tgfb3/Cd34/Nmu/Sfrp1/Lepr/Xbp1/Slc30a8/Ang/Sytl4/Bmp8a/Lpl/Isl1/Syt7/Rgcc/Cftr/Agt	25	BP
GO:0006639	acylglycerol metabolic process	5.24077E-05	Rgn/Pnpla3/Apob/Ces1d/Pck1/Ang/G6pc/Lpl/Cps1/Thrsp/Aadac	11	BP
GO:0010876	lipid localization	6.09515E-05	Fzd4/Crabp2/Shh/Egf/Ffar2/Apob/Pparg/Fabp2/Abcb1a/Bmp6/Lpl/Npc1l1/Igfbp3/Syt7/Fabp4/Adipoq/Aqp8/Selenom/Apod/Cftr/Agt	21	BP
GO:0042060	wound healing	6.15736E-05	Tff3/Rab27a/Dcn/Shh/Serpina10/Fbln1/Pf4/Tgfb3/Cd34/Aqp1/Thbd/F13a1/Arhgef19/Lepr/Xbp1/Gata4/Cpb2/F2/Syt7/Cpq/Gatm/Apod/Igfbp1	23	BP
GO:1901570	fatty acid derivative biosynthetic process	6.25756E-05	Ptgis/Avpr1a/Cyp4a1/Ltc4s/Fcer1a/Ptgds/Edn2/Hmgcs2	8	BP
GO:0007187	G protein-coupled receptor signaling pathway, coupled to cyclic nucleotide second messenger	6.42875E-05	Gpr37/Chga/Sstr5/Ramp2/Hrh4/Adgrg2/Mc2r/Gcg/Pf4/Chrm1/Ramp1/Mrap2/Pde2a/Htr1b/Sstr2/Htr5b	16	BP
GO:0042594	response to starvation	7.10144E-05	Srd5a1/Foxa3/Sfrp1/Pparg/Pck1/Xbp1/Wnt2b/Ugt1a1/Rnase4/Sstr1/Cckar/Cps1/Aldob/Hmgcs2/Sstr2	15	BP
GO:0043270	positive regulation of ion transport	7.17209E-05	Kcnh2/Rgn/Ace2/Slc9a3r2/Gcg/Snca/Trdn/Avpr1a/Chrm1/Abcb1a/Chrna4/Fxyd1/F2/Kcne3/Slc18a1/Nat8l/Cftr/Lrrc26/Agt	19	BP

GO:0006837	serotonin transport	7.17281E-05	Snca/Xbp1/Slc18a1/Htr1b/Fcer1a	5	BP
GO:0097237	cellular response to toxic substance	7.44407E-05	Gpr37/Pycr1/Adh5/Rgn/Hbb/Hba-a1/Aqp1/Gstm5/Abcb1a/Ugt1a1/Gstm1/Cps1/Prdx6/Cfh/Mgst1/LOC100134871/Hba-a2/Ltc4s	18	BP
GO:0051952	regulation of amine transport	7.66465E-05	Chga/Ace2/Snca/Avpr1a/Chrna4/Oxtr/Syt7/Slc18a1/Htr1b/Nat8l/Agt	11	BP
GO:0046461	neutral lipid catabolic process	8.08818E-05	Pnpla3/Apob/Ces1d/Lpl/Cps1/Aadac	6	BP
GO:0046464	acylglycerol catabolic process	8.08818E-05	Pnpla3/Apob/Ces1d/Lpl/Cps1/Aadac	6	BP
GO:0046683	response to organophosphorus	8.53851E-05	Srd5a1/Aqp1/Thbd/Pck1/Tf/Cps1/Pde2a/Pklr/Adipoq/Aldob/Cdo1/Aqp8/Cftr/Hmgcs2	14	BP
GO:0001889	liver development	8.63756E-05	Srd5a1/Rgn/Ephx1/Shh/Serpina10/Dbp/Foxa3/Pck1/Xbp1/Ang/Ugt1a1/Cpb2/Cps1/Aldob/Cftr/Hmgcs2	16	BP
GO:0001658	branching involved in ureteric bud morphogenesis	8.76979E-05	Shh/Six4/Tcf21/Wnt2b/Agtr1b/Maged1/Agt/Gpc3	8	BP
GO:1903961	positive regulation of anion transmembrane transport	9.42468E-05	Abcb1a/Fxyd1/Cftr/Agt	4	BP
GO:0055123	digestive system development	9.47437E-05	Pcsk5/Agr2/Shh/Tcf21/Tp63/Tgfb3/Sfrp1/Xbp1/Gata4/Oxtr/Cps1/Hmgcs2/Spdef	13	BP
GO:0060688	regulation of morphogenesis of a branching structure	9.77301E-05	Shh/Six4/Sfrp1/Wnt2b/Agtr1b/Maged1/Etv5/Agt	8	BP
GO:0061008	hepaticobiliary system development	9.97294E-05	Srd5a1/Rgn/Ephx1/Shh/Serpina10/Dbp/Foxa3/Pck1/Xbp1/Ang/Ugt1a1/Cpb2/Cps1/Aldob/Cftr/Hmgcs2	16	BP
GO:1903522	regulation of blood circulation	9.97294E-05	Chga/Kcnh2/Ace2/Kcnj5/Klk1c9/Nmu/Tacr3/Trdn/Avpr1a/Gata4/Oxtr/Fxyd1/Kcne3/Agtr1b/Edn2/Agt	16	BP
GO:0015837	amine transport	0.000102415	Chga/Ace2/Snca/Avpr1a/Chrna4/Oxtr/Syt7/Slc18a1/Htr1b/Nat8l/Agt	11	BP
GO:0003044	regulation of systemic arterial blood pressure mediated by a chemical signal	0.000109323	Pcsk5/Ace2/Avpr1a/Oxtr/Agtr1b/Edn2/Agt	7	BP
GO:0007631	feeding behavior	0.000109905	Ace2/Nmu/Tacr3/Lepr/Mrap2/Oxtr/Cckar/Pyy/Htr1b/Agtr1b/Agt	11	BP
GO:0010742	macrophage derived foam cell differentiation	0.000114444	Pf4/Apob/Pparg/Lpl/Adipoq	5	BP
GO:0090077	foam cell differentiation	0.000114444	Pf4/Apob/Pparg/Lpl/Adipoq	5	BP
GO:0090190	positive regulation of branching involved in ureteric bud morphogenesis	0.000114444	Six4/Wnt2b/Agtr1b/Maged1/Agt	5	BP
GO:0044058	regulation of digestive system	0.000124	Neurod1/Tac4/Aqp1/Nmu/Spink3/Abcb1a/Oxtr	7	BP

	process				
GO:0051954	positive regulation of amine transport	0.000124	Ace2/Avpr1a/Chrna4/Oxtr/Slc18a1/Nat8l/Agt	7	BP
GO:0014074	response to purine-containing compound	0.000124531	Srd5a1/Aqp1/Thbd/Pparg/Pck1/Tf/Cps1/Pde2a/Pklr/Adipoq/Aldob/Cdo1/Aqp8/Cftr/Hmgcs2	15	BP
GO:0016999	antibiotic metabolic process	0.000130311	Adh5/Dhtkd1/Cyp4b1/Hbb/Hba-a1/Aldh1b1/Snca/Pck1/Cyp4a1/Ugt1a1/Prdx6/Hba-a2	12	BP
GO:0001657	ureteric bud development	0.000130621	Calb1/Shh/Six4/Tcf21/Sfrp1/Wnt2b/Agtr1b/Maged1/Agt/Gpc3	10	BP
GO:0006869	lipid transport	0.000131355	Fzd4/Crabp2/Shh/Egf/Apob/Pparg/Fabp2/Abcb1a/Bmp6/Npc1l1/Igfbp3/Syt7/Fabp4/Adipoq/Aqp8/Selenom/Apod/Cftr/Agt	19	BP
GO:0048871	multicellular organismal homeostasis	0.000131423	Tff3/Neurod1/Col14a1/Tp63/Cd34/Esrrb/Aqp1/Nmu/Prom1/Lepr/Tf/Mrap2/Bmp8a/Bmp6/Prlr/Oxtr/Cckar/Cytl1/Fabp4/Adipoq/Gatm/Cftr/Sftpa1/Edn2	24	BP
GO:0046460	neutral lipid biosynthetic process	0.000132344	Rgn/Pnpla3/Pck1/Ang/Lpl/Thrsp	6	BP
GO:0046463	acylglycerol biosynthetic process	0.000132344	Rgn/Pnpla3/Pck1/Ang/Lpl/Thrsp	6	BP
GO:0042593	glucose homeostasis	0.000134593	Sstr5/Gcg/Neurod1/Pim3/Ffar2/Foxa3/Pparg/Lepr/Serpinf1/Pck1/Xbp1/Ptprn2/Gata4/Cpb2/G6pc/Klf15/Adipoq/Cftr	18	BP
GO:0033500	carbohydrate homeostasis	0.00014019	Sstr5/Gcg/Neurod1/Pim3/Ffar2/Foxa3/Pparg/Lepr/Serpinf1/Pck1/Xbp1/Ptprn2/Gata4/Cpb2/G6pc/Klf15/Adipoq/Cftr	18	BP
GO:0046503	glycerolipid catabolic process	0.000140238	Pnpla3/Apob/Ces1d/Lpl/Cps1/Prdx6/Aadac	7	BP
GO:0072163	mesonephric epithelium development	0.000140957	Calb1/Shh/Six4/Tcf21/Sfrp1/Wnt2b/Agtr1b/Maged1/Agt/Gpc3	10	BP
GO:0072164	mesonephric tubule development	0.000140957	Calb1/Shh/Six4/Tcf21/Sfrp1/Wnt2b/Agtr1b/Maged1/Agt/Gpc3	10	BP
GO:0071392	cellular response to estradiol stimulus	0.000158157	Srd5a1/Sfrp1/Abcb1a/Ugt1a1/Sstr1/Igfbp3/Sstr2	7	BP
GO:0048565	digestive tract development	0.000166294	Pcsk5/Agr2/Shh/Tcf21/Tp63/Tgfb3/Sfrp1/Gata4/Oxtr/Cps1/Hmgcs2/Spdef	12	BP
GO:0019318	hexose metabolic process	0.000166439	Tff3/Rgn/Gcg/Sord/Fbp1/Esrrb/Avpr1a/Lepr/Pck1/G6pc/Igfbp3/Adipoq/Aldob/Apod/Igfbp1	15	BP
GO:0019932	second-messenger-mediated signaling	0.000169356	Chga/Ramp2/Rgn/Adgrg2/Mc2r/Gcg/Neurod1/Pf4/Rasd1/Aqp1/Trdn/Avpr1a/Spink3/Ackr2/Ccr3/Ramp1/Mrap2/Pex5l/Cbs/Pde2a/Fcer1a/Edn2/Trem2	23	BP
GO:0061213	positive regulation of mesonephros development	0.000174362	Six4/Wnt2b/Agtr1b/Maged1/Agt	5	BP
GO:0060675	ureteric bud morphogenesis	0.000179901	Shh/Six4/Tcf21/Wnt2b/Agtr1b/Maged1/Agt/Gpc3	8	BP
GO:0008016	regulation of heart contraction	0.000186979	Chga/Kcnh2/Ace2/Kcnj5/Nmu/Tacr3/Trdn/Avpr1a/Gata4/Fxyd1/Kcne3/Edn2/Agt	13	BP
GO:0050796	regulation of insulin secretion	0.000186979	Nr0b2/Chga/Sstr5/Gcg/Pim3/Sfrp1/Lepr/Slc30a8/Sytl4/Bmp8a/Isl1/Syt7/Cftr	13	BP
GO:0001823	mesonephros de-	0.000189482	Calb1/Shh/Six4/Tcf21/Sfrp1/Wnt2b/Agtr1b/Maged1/Agt/Gpc3	10	BP

	velopment				
GO:0050433	regulation of catecholamine secretion	0.000197948	Chga/Snca/Chrna4/Oxtr/Syt7/Slc18a1/Htr1b/Agt	8	BP
GO:0072171	mesonephric tubule morphogenesis	0.000197948	Shh/Six4/Tcf21/Wnt2b/Agtr1b/Maged1/Agt/Gpc3	8	BP
GO:0072376	protein activation cascade	0.000197948	Masp1/Fcna/Fbln1/F13a1/F2/Cfh/Rgcc/Cfd	8	BP
GO:1990874	vascular associated smooth muscle cell proliferation	0.000199555	Pcsk5/Tgfb3/Pparg/Xbp1/Adipoq/Htr1b/Agt	7	BP
GO:0002526	acute inflammatory response	0.000201046	Masp1/Serpina1/Ffar2/Nupr1/Pparg/Tf/Ugt1a1/Klk1/F2/Cfh/Fcer1a	11	BP
GO:0046888	negative regulation of hormone secretion	0.000203979	Chga/Pim3/Sfrp1/Pparg/Sytl4/Bmp8a/Igfbp3/Adipoq/Edn2	9	BP
GO:0051937	catecholamine transport	0.000203979	Chga/Snca/Chrna4/Oxtr/Syt7/Slc18a1/Htr1b/Nat8l/Agt	9	BP
GO:0019433	triglyceride catabolic process	0.000212059	Pnpla3/Apob/Lpl/Cps1/Aadac	5	BP
GO:0090189	regulation of branching involved in ureteric bud morphogenesis	0.000212059	Six4/Wnt2b/Agtr1b/Maged1/Agt	5	BP
GO:0005996	monosaccharide metabolic process	0.000215122	Tff3/Rgn/Gcg/Sord/Fbp1/Esrrb/Avpr1a/Lepr/Pck1/Ugt1a1/G6pc/Igfbp3/Adipoq/Aldob/Apod/Igfbp1	16	BP
GO:0007193	adenylate cyclase-inhibiting G protein-coupled receptor signaling pathway	0.000217456	Gpr37/Sstr5/Hrh4/Chrm1/Pde2a/Htr1b/Sstr2/Htr5b	8	BP
GO:0060047	heart contraction	0.000240783	Chga/Kcnh2/Ace2/Kcnj5/Nmu/Tacr3/Trdn/Avpr1a/Sgcg/Gata4/Fxyd1/Kcne3/Edn2/Agt	14	BP
GO:0048662	negative regulation of smooth muscle cell proliferation	0.000249286	Ace2/Tgfb3/Pparg/Ang/Igfbp3/Adipoq/Apod	7	BP
GO:0032496	response to lipopolysaccharide	0.000253965	Adh5/Dcn/Serpina1/Cebpe/Pf4/Thbd/Snca/Apob/Pck1/Xbp1/Abcb1a/Ugt1a1/Bmp6/Cckar/Igfbp3/Cps1/Cfh/Mgst1/Pde2a/Ltc4s/Sftpa1/Hmgcs2/Trem2	23	BP
GO:0007263	nitric oxide mediated signal transduction	0.000255655	Neurod1/Rasd1/Spink3/Cbs/Pde2a	5	BP
GO:0033762	response to glucagon	0.000255655	Pck1/Abcb1a/Cps1/Cdo1/Hmgcs2	5	BP
GO:0006641	triglyceride metabolic process	0.000258893	Rgn/Pnpla3/Apob/Pck1/G6pc/Lpl/Cps1/Thrsp/Aadac	9	BP
GO:0046887	positive regulation of hormone secretion	0.000279368	Nr0b2/Gcg/Nmu/Lepr/Spink3/Slc30a8/Bmp6/Cckar/Pex5l/Isl1/Cftr/Edn2	12	BP

GO:0007218	neuropeptide signaling pathway	0.000279716	Sstr5/Mc2r/Tac3/Nmu/Ppy/Prokr2/Sstr1/Pyy/Sstr2	9	BP
GO:0050432	catecholamine secretion	0.000285644	Chga/Snca/Chrna4/Oxtr/Syt7/Slc18a1/Htr1b/Agt	8	BP
GO:0046661	male sex differentiation	0.000305383	Srd5a1/Zfpm2/Shh/Six4/Tcf21/Sfrp1/Nupr1/Lepr/Abcb1a/Gata4/Wnt2b/Bmp6/Mgst1/Cftr	14	BP
GO:0061217	regulation of mesonephros development	0.000305734	Six4/Wnt2b/Agtr1b/Maged1/Agt	5	BP
GO:0090277	positive regulation of peptide hormone secretion	0.000308124	Nr0b2/Gcg/Nmu/Lepr/Spink3/Slc30a8/Cckar/Pex5l/Isl1/Cftr	10	BP
GO:0007589	body fluid secretion	0.000310309	Agr2/Tac4/Aqp1/Chrm1/Spink3/Xbp1/Abcb1a/Prlr/Cckar/Bcat2/Cdo1	11	BP
GO:0050714	positive regulation of protein secretion	0.000318146	Nr0b2/Gcg/Fcna/Ffar2/Tgfb3/Cd34/Nmu/Lepr/Xbp1/Slc30a8/Ang/Sytl4/Lpl/Isl1/Rgcc/Cftr/Agt	17	BP
GO:0042756	drinking behavior	0.000323455	Ace2/Htr1b/Agtr1b/Agt	4	BP
GO:2001023	regulation of response to drug	0.000329283	Gpr37/Pycr1/Snca/Abcb1a/Chrna4/Syt7/Slc18a1/Htr1b/Nat8l/Agt	10	BP
GO:0034284	response to monosaccharide	0.000330638	Nr0b2/Gcg/Neurod1/Pim3/Lepr/Serpinf1/Pck1/Xbp1/Slc30a8/Ptprn2/Gata4/Cpb2/Pklr/Adipoq/Aldob/Cftr/Hmgcs2	17	BP
GO:0003015	heart process	0.000350868	Chga/Kcnh2/Ace2/Kcnj5/Nmu/Tacr3/Trdn/Avpr1a/Sgcg/Gata4/Fxyd1/Kcne3/Edn2/Agt	14	BP
GO:0042744	hydrogen peroxide catabolic process	0.000362906	Hbb/Hba-a1/Snca/Prdx6/Hba-a2	5	BP
GO:0007548	sex differentiation	0.000378347	Srd5a1/Zfpm2/Fzd4/Dmrta1/Shh/Six4/Tcf21/Tp63/Sfrp1/Nupr1/Lepr/Abcb1a/Gata4/Wnt2b/Bmp6/Mgst1/Cftr/Edn2/Agt	19	BP
GO:0048511	rhythmic process	0.000378347	Srd5a1/Chga/Fzd4/Dbp/Nmu/Pparg/Lepr/Serpinf1/Abcb1a/Prokr2/Oxtr/Adipoq/Hlf/Ptgds/Maged1/Sftpa1/Edn2/Adk/Agt	19	BP
GO:0030856	regulation of epithelial cell differentiation	0.000392648	Sfrp4/Shh/Tp63/Tgfb3/Foxa3/Prom1/Abcb1a/Bmp6/Prlr/Adipoq/Cftr	11	BP
GO:0002237	response to molecule of bacterial origin	0.000396174	Adh5/Dcn/Serpina1/Cebpe/Pf4/Thbd/Snca/Apob/Pck1/Xbp1/Abcb1a/Ugt1a1/Bmp6/Cckar/Igfbp3/Cps1/Cfh/Mgst1/Pde2a/Ltc4s/Sftpa1/Hmgcs2/Trem2	23	BP
GO:0010226	response to lithium ion	0.000400668	Shh/Pparg/Gstm1/Slc18a1/Fabp4/Pklr	6	BP
GO:0045823	positive regulation of heart contraction	0.000400668	Chga/Ace2/Nmu/Tacr3/Avpr1a/Edn2	6	BP
GO:0007584	response to nutrient	0.00040497	Gcg/Sfrp1/Pparg/Lepr/Xbp1/Abcb1a/Gata4/Ugt1a1/Klk1/Cckar/Cbs/Slc6a19/Pklr/Adipoq/Ltc4s/Gatm/Agtr1b/Sftpa1/Hmgcs2	19	BP
GO:0060993	kidney morphogenesis	0.000405747	Calb1/Shh/Six4/Tcf21/Wnt2b/Agtr1b/Maged1/Agt/Gpc3	9	BP
GO:0009743	response to carbohydrate	0.000406121	Nr0b2/Gcg/Neurod1/Pim3/Apob/Lepr/Serpinf1/Pck1/Xbp1/Slc30a8/Ptprn2/Gata4/Cpb2/Pklr/Adipoq/Aldob/Cftr/Hmgcs2	18	BP
GO:0072330	monocarboxylic acid biosynthetic process	0.000406321	Dhtkd1/Rgn/Ptgis/Crabp2/Fbp1/Thnsl2/Acsm5/Esrrb/Avpr1a/Lpl/Pklr/Aldob/Ptgds/Edn2/Agt	15	BP

GO:0072224	metanephric glomerulus development	0.00041577	Tcf21/Cd34/Aqp1/Adipoq	4	BP
GO:0030858	positive regulation of epithelial cell differentiation	0.000418082	Sfrp4/Foxa3/Prom1/Abcb1a/Bmp6/Adipoq/Cftr	7	BP
GO:0060986	endocrine hormone secretion	0.000418082	Fzd4/Aqp1/Avpr1a/Bmp6/Pex5l/Selenom/Agt	7	BP
GO:0003018	vascular process in circulatory system	0.000422391	Chga/Ramp2/Klk1c9/Avpr1a/Oxtr/Cps1/Cbs/Pde2a/Htr1b/Agtr1b/Cftr/Edn2/Agt	13	BP
GO:0046364	monosaccharide biosynthetic process	0.000437462	Rgn/Gcg/Sord/Fbp1/Lepr/Pck1/G6pc/Adipoq	8	BP
GO:0072078	nephron tubule morphogenesis	0.000437462	Shh/Six4/Tcf21/Wnt2b/Agtr1b/Maged1/Agt/Gpc3	8	BP
GO:1903531	negative regulation of secretion by cell	0.00043957	Chga/Pim3/Fbln1/Cd34/Snca/Sfrp1/Pparg/Sytl4/Bmp8a/Igfbp3/Adipoq/Rgcc/Htr1b/Edn2	14	BP
GO:0034767	positive regulation of ion transmembrane transport	0.000451989	Kcnh2/Rgn/Slc9a3r2/Snca/Trdn/Abcb1a/Fxyd1/F2/Kcne3/Cftr/Lrrc26/Agt	12	BP
GO:0051480	regulation of cytosolic calcium ion concentration	0.000452399	Jph1/Calb1/Chga/Hrh4/Tac4/Nmu/Snca/Trdn/Avpr1a/Spink3/Ackr2/Ccr3/Oxtr/Fxyd1/Cckar/F2/Htr1b/Ms4a2/Edn2/Agt	20	BP
GO:0072088	nephron epithelium morphogenesis	0.000474524	Shh/Six4/Tcf21/Wnt2b/Agtr1b/Maged1/Agt/Gpc3	8	BP
GO:0006006	glucose metabolic process	0.000487046	Tff3/Rgn/Gcg/Fbp1/Esrrb/Avpr1a/Lepr/Pck1/G6pc/Igfbp3/Adipoq/Apod/Igfbp1	13	BP
GO:0062013	positive regulation of small molecule metabolic process	0.000492594	Rgn/Guca2a/Gcg/Egf/Esrrb/Snca/Pparg/Avpr1a/Bmp6/Adipoq/Agt	11	BP
GO:0001678	cellular glucose homeostasis	0.00051038	Gcg/Neurod1/Pim3/Foxa3/Lepr/Serpinf1/Pck1/Xbp1/Ptprn2/Gata4/Cpb2/Klf15/Cftr	13	BP
GO:1901606	alpha-amino acid catabolic process	0.000514091	Aadat/Thnsl2/Adhfe1/Oat/Fah/Cbs/Bcat2/Cdo1	8	BP
GO:0010675	regulation of cellular carbohydrate metabolic process	0.000520658	Tff3/Rgn/Gcg/Fbp1/Esrrb/Snca/Avpr1a/Lepr/Ugt1a1/Igfbp3/Adipoq	11	BP
GO:0007565	female pregnancy	0.000524514	Ramp2/Pcsk5/Sfrp4/Ptgis/Ace2/Cyp2d2/Fbln1/Tgfb3/Thbd/Lepr/Oxtr/Igfbp3/Cbs/Pzp/Maged2/Agt	16	BP
GO:0009746	response to hexose	0.000544835	Nr0b2/Gcg/Neurod1/Pim3/Lepr/Serpinf1/Pck1/Xbp1/Slc30a8/Ptprn2/Gata4/Cpb2/Pklr/Adipoq/Aldob/Cftr	16	BP
GO:0071496	cellular response to external stimulus	0.000546592	Srd5a1/Aqp1/Foxa3/Sfrp1/Pparg/Avpr1a/Pck1/Xbp1/Abcb1a/Asgr1/Wnt2b/Rnase4/Bmp6/F2/Cbs/Pde2a/Aldob/Ltc4s/Sftpa1/Agt	20	BP
GO:0006109	regulation of carbohydrate metabolic process	0.000553611	Tff3/Rgn/Gcg/Fbp1/Egf/Esrrb/Snca/Avpr1a/Lepr/Ugt1a1/Igfbp3/Adipoq	12	BP
GO:0072028	nephron morphogenesis	0.000556289	Shh/Six4/Tcf21/Wnt2b/Agtr1b/Maged1/Agt/Gpc3	8	BP
GO:0015696	ammonium transport	0.000580298	Aqp1/Snca/Xbp1/Abcb1a/Chrna4/Syt7/Slc18a1/Htr1b/Nat8l/Fcer1a	10	BP

GO:0043627	response to estrogen	0.000580298	Srd5a1/Tgfb3/Aqp1/Sfrp1/Gstm5/Pparg/Lepr/Gata4/Agtr1b/Cftr	10	BP
GO:0062012	regulation of small molecule metabolic process	0.000594158	Tff3/Rgn/Guca2a/Gcg/Fbp1/Egf/Esrrb/Snca/Apob/Pparg/Avpr1a/Lepr/Ugt1a1/Bmp6/Igfbp3/Pde2a/Adipoq/Agt	18	BP
GO:2000027	regulation of animal organ morphogenesis	0.000613421	Shh/Six4/Cd34/Sfrp1/Xbp1/Wnt2b/Cpb2/Isl1/Agtr1b/Maged1/Etv5/Agt/Gpc3	13	BP
GO:0016525	negative regulation of angiogenesis	0.000615235	Dcn/Pf4/Pparg/Serpinf1/Mmrn2/Tnmd/Meox2/Rgcc/Agt	9	BP
GO:0044706	multi-multicellular organism process	0.000640258	Ramp2/Pcsk5/Sfrp4/Ptgis/Ace2/Cyp2d2/Fbln1/Tgfb3/Thbd/Avpr1a/Lepr/Oxtr/Igfbp3/Cbs/Pzp/Maged2/Agt	17	BP
GO:0061333	renal tubule morphogenesis	0.000649089	Shh/Six4/Tcf21/Wnt2b/Agtr1b/Maged1/Agt/Gpc3	8	BP
GO:0042178	xenobiotic catabolic process	0.00065425	Gstm5/Ugt1a1/Gstm1/Gsta1	4	BP
GO:0015849	organic acid transport	0.000686552	Slc35a1/Slc38a11/Slc16a7/Ace2/Slc1a7/Slc16a12/Slc26a7/Slc16a5/Snca/Pparg/Avpr1a/Fabp2/Fxyd1/Slc6a19/Fabp4/Aqp8/Agt	17	BP
GO:0050727	regulation of inflammatory response	0.000686552	Ptgis/Ccl24/Masp1/Ffar2/Snca/Pparg/Serpinf1/Klk1/F2/Isl1/Cfh/Fabp4/Pde2a/Adipoq/Apod/Fcer1a/Agt	17	BP
GO:1905330	regulation of morphogenesis of an epithelium	0.000702103	Shh/Six4/Sfrp1/Wnt2b/Agtr1b/Maged1/Etv5/Agt/Gpc3	9	BP
GO:2000181	negative regulation of blood vessel morphogenesis	0.000749147	Dcn/Pf4/Pparg/Serpinf1/Mmrn2/Tnmd/Meox2/Rgcc/Agt	9	BP
GO:0032941	secretion by tissue	0.000794243	Agr2/Tac4/Aqp1/Chrm1/Spink3/Cckar	6	BP
GO:0015698	inorganic anion transport	0.00079705	Sfrp4/Slc26a7/Slc4a10/Clca1/Ano4/Abcb1a/Fxyd1/Ankh/Slc12a8/Cftr/Ros1	11	BP
GO:0003014	renal system process	0.000798729	Pcsk5/Hbb/Cd34/Aqp1/Avpr1a/Adipoq/Agtr1b/Maged2/Agt	9	BP
GO:0070293	renal absorption	0.000803903	Hbb/Aqp1/Adipoq/Maged2	4	BP
GO:1903524	positive regulation of blood circulation	0.000811328	Chga/Ace2/Klk1c9/Nmu/Tacr3/Avpr1a/Oxtr/Edn2	8	BP
GO:0051271	negative regulation of cellular component movement	0.000815022	Rgn/Dcn/Slit1/Shh/Fbln1/Sfrp1/Pparg/Serpinf1/Mmrn2/Nexmif/Igfbp3/Kcne3/Adipoq/Meox2/Apod/Rgcc/Padi2	17	BP
GO:0033002	muscle cell proliferation	0.000862682	Pcsk5/Zfpm2/Ace2/Shh/Tgfb3/Pparg/Xbp1/Ang/Gata4/Igfbp3/Adipoq/Apod/Htr1b/Agt	14	BP
GO:0031099	regeneration	0.000876984	Nr0b2/Rgn/Shh/Serpina10/Pparg/Gata4/Ugt1a1/Cpb2/Cckar/Isl1/Klk8/Cpq/Gatm/Apod/Cftr/Igfbp1	16	BP
GO:0010596	negative regulation of endothelial cell migration	0.000881666	Dcn/Pparg/Serpinf1/Mmrn2/Meox2/Rgcc	6	BP
GO:0019432	triglyceride biosynthetic process	0.000891665	Rgn/Pnpla3/Pck1/Lpl/Thrsp	5	BP
GO:0008584	male gonad devel-	0.000894562	Srd5a1/Zfpm2/Six4/Tcf21/Sfrp1/Nupr1/Lepr/Abcb1a/Gata4/Wnt	12	BP

	opment		2b/Mgst1/Cftr		
GO:0046546	development of primary male sexual characteristics	0.000936647	Srd5a1/Zfpm2/Six4/Tcf21/Sfrp1/Nupr1/Lepr/Abcb1a/Gata4/Wnt2b/Mgst1/Cftr	12	BP
GO:0070098	chemokine-mediated signaling pathway	0.00094238	Ccl24/Pf4/Ackr2/Ccr3/Ccl11/Padi2/Trem2	7	BP
GO:0006749	glutathione metabolic process	0.000976316	Cth/Hbb/Gstm5/Gstm1/Mgst1/Gsta1	6	BP
GO:0090257	regulation of muscle system process	0.001010522	Chga/Ace2/Pi16/Col14a1/Nmu/Tacr3/Gata4/Oxtr/Fxyd1/Kcne3/Edn2/Adk/Sstr2/Agt	14	BP
GO:0042537	benzene-containing compound metabolic process	0.001017267	Aadat/Cyp4b1/Gstm5/Ugt1a1/Gstm1	5	BP
GO:0097009	energy homeostasis	0.001017267	Nmu/Lepr/Mrap2/Bmp8a/Edn2	5	BP
GO:0001656	metanephros development	0.0010767	Calb1/Shh/Six4/Tcf21/Cd34/Aqp1/Adipoq/Gpc3	8	BP
GO:0001676	long-chain fatty acid metabolic process	0.0010767	Cyp2d5/Cyp2d2/Pnpla3/Cyp2c24/Cyp4a1/Cyp4f1/Alox12e/Gsta1	8	BP
GO:0061138	morphogenesis of a branching epithelium	0.001077491	Shh/Egf/Six4/Tcf21/Tp63/Sfrp1/Wnt2b/Ccl11/Agtr1b/Maged1/Etv5/Agt/Gpc3	13	BP
GO:0002067	glandular epithelial cell differentiation	0.001078586	Neurod1/Agr2/Tp63/Bmp6/Cftr/Spdef	6	BP
GO:0019369	arachidonic acid metabolic process	0.001078586	Cyp2d5/Cyp2d2/Cyp2c24/Cyp4a1/Cyp4f1/Alox12e	6	BP
GO:0045933	positive regulation of muscle contraction	0.001078586	Chga/Ace2/Nmu/Tacr3/Oxtr/Edn2	6	BP
GO:0001999	renal response to blood flow involved in circulatory renin-angiotensin regulation of systemic arterial blood pressure	0.001087194	Pcsk5/Agtr1b/Agt	3	BP
GO:0070278	extracellular matrix constituent secretion	0.001087194	Cpb2/Rgcc/Agt	3	BP
GO:2000020	positive regulation of male gonad development	0.001087194	Zfpm2/Abcb1a/Cftr	3	BP
GO:0009749	response to glucose	0.001118163	Nr0b2/Gcg/Neurod1/Pim3/Lepr/Serpinf1/Pck1/Xbp1/Slc30a8/Ptprn2/Gata4/Cpb2/Pklr/Adipoq/Cftr	15	BP
GO:0035690	cellular response to drug	0.001138662	Srd5a1/Gpr37/Pycr1/Kcnh2/Hrh4/Fbp1/Aqp1/Sfrp1/Chrm1/Serpinf1/Pck1/Abcb1a/Chrna4/Ugt1a1/Gstm1/Cfh/Pde2a/Adipoq/Htr1b/Agtr1b/Cftr/Sftpa1	22	BP

GO:0044070	regulation of anion transport	0.001155356	Sfrp4/Ace2/Snca/Avpr1a/Abcb1a/Fxyd1/Cftr/Ros1/Agt	9	BP
GO:0008406	gonad development	0.001156938	Srd5a1/Zfpm2/Fzd4/Dmrta1/Six4/Tcf21/Sfrp1/Nupr1/Lepr/Abcb1a/Gata4/Wnt2b/Mgst1/Cftr/Edn2/Agt	16	BP
GO:1904018	positive regulation of vasculature development	0.00117262	Cth/Ramp2/Ptgis/Ccl24/Shh/Cd34/Aqp1/Xbp1/Ccr3/Gata4/Isl1/Ccl11	12	BP
GO:0044342	type B pancreatic cell proliferation	0.001172847	Fmc1/Sfrp1/Igfbp3/Adk	4	BP
GO:0061005	cell differentiation involved in kidney development	0.001188876	Shh/Tcf21/Cd34/Prom1/Klf15/Adipoq	6	BP
GO:1904705	regulation of vascular associated smooth muscle cell proliferation	0.001188876	Tgfb3/Pparg/Xbp1/Adipoq/Htr1b/Agt	6	BP
GO:0010906	regulation of glucose metabolic process	0.001299405	Tff3/Rgn/Gcg/Fbp1/Esrrb/Avpr1a/Lepr/Igfbp3/Adipoq	9	BP
GO:1905332	positive regulation of morphogenesis of an epithelium	0.001307237	Six4/Wnt2b/Agtr1b/Maged1/Agt	5	BP
GO:0048546	digestive tract morphogenesis	0.001307594	Agr2/Shh/Tcf21/Tp63/Sfrp1/Gata4	6	BP
GO:0009063	cellular amino acid catabolic process	0.001317925	Aadat/Thnsl2/Adhfe1/Oat/Fah/Cbs/Bcat2/Cdo1	8	BP
GO:0098869	cellular oxidant detoxification	0.001317925	Rgn/Hbb/Hba-a1/Prdx6/Mgst1/LOC100134871/Hba-a2/Ltc4s	8	BP
GO:0051224	negative regulation of protein transport	0.001395146	Chga/Pim3/Fbln1/Cd34/Sfrp1/Sytl4/Anxa13/Bmp8a/Pde2a/Adipoq/Apod/Rgcc	12	BP
GO:0001696	gastric acid secretion	0.001395668	Slc26a7/Nmu/Oxtr/Cckar	4	BP
GO:0071285	cellular response to lithium ion	0.001395668	Shh/Pparg/Slc18a1/Fabp4	4	BP
GO:0019319	hexose biosynthetic process	0.00140275	Gcg/Sord/Fbp1/Lepr/Pck1/G6pc/Adipoq	7	BP
GO:0002027	regulation of heart rate	0.001407245	Kcnh2/Kcnj5/Nmu/Tacr3/Avpr1a/Kcne3/Edn2/Agt	8	BP
GO:0050680	negative regulation of epithelial cell proliferation	0.001408595	Rgn/Sfrp1/Pparg/Serpinf1/Cpb2/Isl1/Tnmd/Rgcc/Maged1/Gpc3	10	BP
GO:0045137	development of primary sexual characteristics	0.001413541	Srd5a1/Zfpm2/Fzd4/Dmrta1/Six4/Tcf21/Sfrp1/Nupr1/Lepr/Abcb1a/Gata4/Wnt2b/Mgst1/Cftr/Edn2/Agt	16	BP
GO:0003012	muscle system process	0.001443444	Chga/Kcnh2/Ace2/Pi16/Kcnj5/Col14a1/Nmu/Tacr3/Gata4/Oxtr/Fxyd1/Kcne3/Klf15/Myom2/Gatm/Edn2/Adk/Sstr2/Agt	19	BP
GO:0006000	fructose metabolic process	0.00147092	Sord/Fbp1/Aldob	3	BP
GO:0010744	positive regulation of macrophage	0.00147092	Pf4/Apob/Lpl	3	BP

	derived foam cell differentiation				
GO:0051458	corticotropin secretion	0.00147092	Aqp1/Avpr1a/Pex5l	3	BP
GO:0001935	endothelial cell proliferation	0.001482214	Ccl24/Cd34/Pparg/Xbp1/Ccr3/Ang/Bmp6/Tnmd/Ccl11/Rgcc	10	BP
GO:0035150	regulation of tube size	0.001499229	Chga/Klk1c9/Avpr1a/Oxtr/Cps1/Cbs/Htr1b/Agtr1b/Cftr/Edn2/Agt	11	BP
GO:0044262	cellular carbohydrate metabolic process	0.001534915	Tff3/Rgn/Gcg/Sord/Fbp1/Esrrb/Snca/B3gnt6/Avpr1a/Lepr/Pck1/Ugt1a1/G6pc/Igfbp3/Adipoq	15	BP
GO:1901343	negative regulation of vasculature development	0.0015423	Dcn/Pf4/Pparg/Serpinf1/Mmrn2/Tnmd/Meox2/Rgcc/Agt	9	BP
GO:0071548	response to dexamethasone	0.001600287	Srd5a1/Aqp1/Lepr/Serpinf1/Pck1/Abcb1a/Cps1/Agtr1b	8	BP
GO:0072010	glomerular epithelium development	0.001646401	Cd34/Prom1/Klf15/Adipoq	4	BP
GO:0098810	neurotransmitter reuptake	0.001646401	Slc1a7/Snca/Slc18a1/Nat8l	4	BP
GO:1904950	negative regulation of establishment of protein localization	0.001651476	Chga/Pim3/Fbln1/Cd34/Sfrp1/Sytl4/Anxa13/Bmp8a/Pde2a/Adipoq/Apod/Rgcc	12	BP
GO:0045923	positive regulation of fatty acid metabolic process	0.001654044	Rgn/Pparg/Avpr1a/Adipoq/Agt	5	BP
GO:0009266	response to temperature stimulus	0.001671684	Cd34/Tac4/Pparg/Cpb2/Lpl/Scara5/Cckar/Hspb7/Pklr/Htr1b/Cftr/Hmgcs2/Agt	13	BP
GO:0045766	positive regulation of angiogenesis	0.001718041	Ramp2/Ptgis/Ccl24/Shh/Cd34/Aqp1/Xbp1/Ccr3/Gata4/Isl1/Ccl11	11	BP
GO:0015711	organic anion transport	0.001725017	Slc35a1/Slc38a11/Slc16a7/Ace2/Slc1a7/Slc16a12/Slc26a7/Slc16a5/Slc4a10/Snca/Pparg/Avpr1a/Fabp2/Abcb1a/G6pc/Slc6a19/Fabp4/Aqp8/Cftr/Agt	20	BP
GO:0046942	carboxylic acid transport	0.001771978	Slc35a1/Slc38a11/Slc16a7/Ace2/Slc1a7/Slc16a12/Slc26a7/Slc16a5/Snca/Pparg/Avpr1a/Fabp2/Slc6a19/Fabp4/Aqp8/Agt	16	BP
GO:0048659	smooth muscle cell proliferation	0.001796522	Pcsk5/Ace2/Tgfb3/Pparg/Xbp1/Ang/Igfbp3/Adipoq/Apod/Htr1b/Agt	11	BP
GO:2001025	positive regulation of response to drug	0.001850727	Abcb1a/Chrna4/Slc18a1/Nat8l/Agt	5	BP
GO:0061337	cardiac conduction	0.001875184	Kcnh2/Ace2/Kcnj5/Trdn/Kcne3/Agt	6	BP
GO:2000146	negative regulation of cell motility	0.001879513	Rgn/Dcn/Shh/Fbln1/Sfrp1/Pparg/Serpinf1/Mmrn2/Nexmif/Igfbp3/Adipoq/Meox2/Apod/Rgcc/Padi2	15	BP
GO:0051222	positive regulation of protein transport	0.001916177	Nr0b2/Gcg/Fcna/Shh/Ffar2/Tgfb3/Cd34/Nmu/Chrm1/Lepr/Xbp1/Slc30a8/Ang/Sytl4/Anxa13/Lpl/Isl1/Rgcc/Cftr/Agt	20	BP
GO:0001516	prostaglandin biosynthetic process	0.00192679	Ptgis/Avpr1a/Ptgds/Edn2	4	BP
GO:0046457	prostanoid biosynthetic process	0.00192679	Ptgis/Avpr1a/Ptgds/Edn2	4	BP

GO:0002016	regulation of blood volume by renin-angiotensin	0.00192981	Ace2/Agtr1b/Agt	3	BP
GO:0010749	regulation of nitric oxide mediated signal transduction	0.00192981	Spink3/Cbs/Pde2a	3	BP
GO:0046459	short-chain fatty acid metabolic process	0.00192981	Thnsl2/Ces1d/Pck1	3	BP
GO:0070471	uterine smooth muscle contraction	0.00192981	Tacr3/Oxtr/Agt	3	BP
GO:0072075	metanephric mesenchyme development	0.00192981	Shh/Six4/Tcf21	3	BP
GO:0072378	blood coagulation, fibrin clot formation	0.00192981	Fbln1/F13a1/F2	3	BP
GO:1901605	alpha-amino acid metabolic process	0.001945384	Pycr1/Cth/Aadat/Thnsl2/Adhfe1/Oat/Fah/Cps1/Cbs/Bcat2/Cdo1/Gatm	12	BP
GO:1990868	response to chemokine	0.002022377	Ccl24/Pf4/Ackr2/Ccr3/Ccl11/Padi2/Trem2	7	BP
GO:1990869	cellular response to chemokine	0.002022377	Ccl24/Pf4/Ackr2/Ccr3/Ccl11/Padi2/Trem2	7	BP
GO:0015872	dopamine transport	0.002042427	Snca/Chrna4/Syt7/Slc18a1/Htr1b/Nat8l	6	BP
GO:0006937	regulation of muscle contraction	0.002089676	Chga/Ace2/Nmu/Tacr3/Gata4/Oxtr/Fxyd1/Kcne3/Edn2/Sstr2	10	BP
GO:0022612	gland morphogenesis	0.002089676	Edar/Sfrp4/Shh/Tp63/Tgfb3/Sfrp1/Xbp1/Cpb2/Ccl11/Etv5	10	BP
GO:0009755	hormone-mediated signaling pathway	0.002192881	Nr0b2/Sstr5/Tcf21/Tp63/Esrrb/Sfrp1/Pparg/Prlr/Sstr1/Isl1/Padi2/Sstr2	12	BP
GO:0048754	branching morphogenesis of an epithelial tube	0.002234029	Shh/Egf/Six4/Tcf21/Wnt2b/Ccl11/Agtr1b/Maged1/Etv5/Agt/Gpc3	11	BP
GO:0006633	fatty acid biosynthetic process	0.002252066	Rgn/Ptgis/Thnsl2/Acsm5/Avpr1a/Lpl/Ptgds/Edn2/Agt	9	BP
GO:0001763	morphogenesis of a branching structure	0.00225628	Shh/Egf/Six4/Tcf21/Tp63/Sfrp1/Wnt2b/Ccl11/Agtr1b/Maged1/Etv5/Agt/Gpc3	13	BP
GO:0030301	cholesterol transport	0.002322341	Shh/Egf/Apob/Npc1l1/Syt7/Adipoq/Cftr	7	BP
GO:0046165	alcohol biosynthetic process	0.002371778	Ephx1/Snca/Apob/Ces1d/Pck1/Bmp6/Npc1l1/Cftr/Hmgcs2	9	BP
GO:0090278	negative regulation of peptide hormone secretion	0.002410445	Chga/Pim3/Sfrp1/Pparg/Sytl4/Bmp8a	6	BP
GO:1901607	alpha-amino acid biosynthetic process	0.002410445	Pycr1/Cth/Oat/Cbs/Bcat2/Gatm	6	BP
GO:1903793	positive regulation of anion transport	0.002410445	Ace2/Avpr1a/Abcb1a/Fxyd1/Cftr/Agt	6	BP

GO:0006805	xenobiotic metabolic process	0.002446305	Cyp2d5/Cyp2d2/Gstm5/Cyp2c24/Ugt1a1/Gstm1/Cdo1/Gsta1	8	BP
GO:0002551	mast cell chemotaxis	0.002468611	Chga/Ccr3/Ccl11	3	BP
GO:0009071	serine family amino acid catabolic process	0.002468611	Thnsl2/Cbs/Cdo1	3	BP
GO:0010885	regulation of cholesterol storage	0.002468611	Apob/Pparg/Lpl	3	BP
GO:0014820	tonic smooth muscle contraction	0.002468611	Nmu/Edn2/Agt	3	BP
GO:0050667	homocysteine metabolic process	0.002468611	Cth/Cps1/Cbs	3	BP
GO:0071872	cellular response to epinephrine stimulus	0.002468611	Srd5a1/Pklr/Adipoq	3	BP
GO:0033189	response to vitamin A	0.002543493	Pparg/Abcb1a/Gata4/Ltc4s/Sftpa1	5	BP
GO:0006874	cellular calcium ion homeostasis	0.002560737	Jph1/Calb1/Chga/Rgn/Hrh4/Tac4/Nmu/Snca/Trdn/Avpr1a/Spink3/Ackr2/Ccr3/Oxtr/Fxyd1/Cckar/F2/Htr1b/Ms4a2/Edn2/Agt	21	BP
GO:0035929	steroid hormone secretion	0.002583412	Fzd4/Bmp6/Selenom/Agt	4	BP
GO:1904738	vascular associated smooth muscle cell migration	0.002583412	Pcsk5/Xbp1/Adipoq/Agt	4	BP
GO:1904752	regulation of vascular associated smooth muscle cell migration	0.002583412	Pcsk5/Xbp1/Adipoq/Agt	4	BP
GO:1905939	regulation of gonad development	0.002583412	Zfpm2/Nupr1/Abcb1a/Cftr	4	BP
GO:0097756	negative regulation of blood vessel diameter	0.00259134	Chga/Klk1c9/Avpr1a/Oxtr/Htr1b/Agtr1b/Edn2/Agt	8	BP
GO:0007596	blood coagulation	0.002635507	Rab27a/Shh/Serpina10/Fbln1/Pf4/Cd34/Thbd/F13a1/Cpb2/F2	10	BP
GO:0070371	ERK1 and ERK2 cascade	0.002717725	Rps6ka6/Alkal2/Ace2/Ccl24/Gcg/Egf/Fbln1/Tf/Xbp1/Ccr3/Gata4/Oxtr/Adipoq/Ccl11/Ros1/Agt/Trem2	17	BP
GO:0071333	cellular response to glucose stimulus	0.002754281	Gcg/Neurod1/Pim3/Lepr/Serpinf1/Pck1/Xbp1/Ptprn2/Gata4/Cpb2/Cftr	11	BP
GO:0006695	cholesterol biosynthetic process	0.002811346	Apob/Ces1d/Npc1l1/Cftr/Hmgcs2	5	BP
GO:0002673	regulation of acute inflammatory response	0.002826218	Masp1/Ffar2/Pparg/Klk1/Cfh/Fcer1a	6	BP
GO:1901615	organic hydroxy compound metabolic process	0.00287046	Srd5a1/Gpr37/Calb1/Adh5/Ephx1/Aldh1b1/Sord/Snca/Tacr3/Apob/Ces1d/Lepr/Pck1/Bmp6/Npc1l1/Fah/Cpq/Cftr/Rbp2/Hmgcs2	20	BP
GO:0007599	hemostasis	0.002883469	Rab27a/Shh/Serpina10/Fbln1/Pf4/Cd34/Thbd/F13a1/Cpb2/F2	10	BP
GO:0035296	regulation of tube	0.002883469	Chga/Klk1c9/Avpr1a/Oxtr/Cps1/Htr1b/Agtr1b/Cftr/Edn2/Agt	10	BP

	diameter				
GO:0097746	regulation of blood vessel diameter	0.002883469	Chga/Klk1c9/Avpr1a/Oxtr/Cps1/Htr1b/Agtr1b/Cftr/Edn2/Agt	10	BP
GO:0030323	respiratory tube development	0.002896876	Pcsk5/Zfpm2/Agr2/Shh/Tcf21/Tgfb3/Gata4/Wnt2b/Cftr/Edn2/Hmgcs2/Spdef/Gpc3	13	BP
GO:0003081	regulation of systemic arterial blood pressure by renin-angiotensin	0.002963025	Pcsk5/Ace2/Agtr1b/Agt	4	BP
GO:0010460	positive regulation of heart rate	0.002963025	Nmu/Tacr3/Avpr1a/Edn2	4	BP
GO:0071331	cellular response to hexose stimulus	0.002987968	Gcg/Neurod1/Pim3/Lepr/Serpinf1/Pck1/Xbp1/Ptprn2/Gata4/Cpb2/Cftr	11	BP
GO:1904951	positive regulation of establishment of protein localization	0.003013866	Nr0b2/Gcg/Fcna/Shh/Ffar2/Tgfb3/Cd34/Nmu/Chrm1/Lepr/Xbp1/Slc30a8/Ang/Sytl4/Anxa13/Lpl/Isl1/Rgcc/Cftr/Agt	20	BP
GO:0060541	respiratory system development	0.003046818	Zfpm2/Agr2/Shh/Six4/Tcf21/Tgfb3/Fgfrl1/Gata4/Wnt2b/Cftr/Edn2/Hmgcs2/Spdef/Gpc3	14	BP
GO:0010642	negative regulation of platelet-derived growth factor receptor signaling pathway	0.003091652	Snca/Adipoq/Apod	3	BP
GO:0097531	mast cell migration	0.003091652	Chga/Ccr3/Ccl11	3	BP
GO:2000018	regulation of male gonad development	0.003091652	Zfpm2/Abcb1a/Cftr	3	BP
GO:0014046	dopamine secretion	0.003099016	Snca/Chrna4/Syt7/Slc18a1/Htr1b	5	BP
GO:0014059	regulation of dopamine secretion	0.003099016	Snca/Chrna4/Syt7/Slc18a1/Htr1b	5	BP
GO:1902653	secondary alcohol biosynthetic process	0.003099016	Apob/Ces1d/Npc1l1/Cftr/Hmgcs2	5	BP
GO:0042180	cellular ketone metabolic process	0.003237359	Srd5a1/Aadat/Rgn/Snca/Pparg/Avpr1a/Ugt1a1/Bmp6/Coq8a/Adipoq/Agt	11	BP
GO:0008652	cellular amino acid biosynthetic process	0.003293461	Pycr1/Cth/Oat/Cbs/Bcat2/Gatm	6	BP
GO:0016054	organic acid catabolic process	0.003330086	Aadat/Thnsl2/Adhfe1/Oat/Ces1d/Pck1/Phyh/Fah/Cbs/Bcat2/Adipoq/Cdo1	12	BP
GO:0046395	carboxylic acid catabolic process	0.003330086	Aadat/Thnsl2/Adhfe1/Oat/Ces1d/Pck1/Phyh/Fah/Cbs/Bcat2/Adipoq/Cdo1	12	BP
GO:0071326	cellular response to monosaccharide stimulus	0.003368177	Gcg/Neurod1/Pim3/Lepr/Serpinf1/Pck1/Xbp1/Ptprn2/Gata4/Cpb2/Cftr	11	BP
GO:0001558	regulation of cell growth	0.003373823	Cth/Crabp2/Pi16/Slit1/Fbp1/Igfbp6/Col14a1/Sfrp1/Cgref1/Pparg/Avpr1a/Xbp1/Igfbp3/F2/Ros1/Igfbp1/Dact3/Esm1/Agt	19	BP
GO:0007204	positive regulation of cytosolic calcium ion concentra-	0.003428917	Jph1/Hrh4/Tac4/Nmu/Snca/Trdn/Avpr1a/Spink3/Ackr2/Ccr3/Oxtr/Cckar/F2/Ms4a2/Edn2/Agt	16	BP

	tion				
GO:0050817	coagulation	0.003435207	Rab27a/Shh/Serpina10/Fbln1/Pf4/Cd34/Thbd/F13a1/Cpb2/F2	10	BP
GO:0032835	glomerulus development	0.003547559	Tcf21/Cd34/Aqp1/Prom1/Klf15/Adipoq	6	BP
GO:0040013	negative regulation of locomotion	0.003628161	Rgn/Dcn/Slit1/Shh/Fbln1/Sfrp1/Pparg/Serpinf1/Mmrn2/Nexmif/Igfbp3/Adipoq/Meox2/Apod/Rgcc/Padi2	16	BP
GO:0001542	ovulation from ovarian follicle	0.003802864	Lepr/Edn2/Agt	3	BP
GO:0009750	response to fructose	0.003802864	Pck1/Xbp1/Aldob	3	BP
GO:0010878	cholesterol storage	0.003802864	Apob/Pparg/Lpl	3	BP
GO:0046541	saliva secretion	0.003802864	Tac4/Aqp1/Chrm1	3	BP
GO:0002021	response to dietary excess	0.003833082	Pnpla3/Pparg/Bmp8a/Cckar	4	BP
GO:0006720	isoprenoid metabolic process	0.00387773	Srd5a1/Adh5/Crabp2/Ces2a/Phyh/Rbp2/Hmgcs2	7	BP
GO:2000241	regulation of reproductive process	0.004067727	Zfpm2/Shh/Sfrp1/Nupr1/Serpinf1/Spink3/Abcb1a/Oxtr/Ptgds/Cftr	10	BP
GO:0009651	response to salt stress	0.004089045	Aqp1/Tacr3/Abcb1a/Agtr1b/Agt	5	BP
GO:0006721	terpenoid metabolic process	0.004099082	Srd5a1/Adh5/Crabp2/Ces2a/Rbp2/Hmgcs2	6	BP
GO:0071398	cellular response to fatty acid	0.004099082	Ffar2/Sfrp1/Apob/Pparg/Cps1/Hmgcs2	6	BP
GO:0031016	pancreas development	0.004367562	Neurod1/Shh/Xbp1/Bmp6/Cckar/Isl1/Cftr	7	BP
GO:0048660	regulation of smooth muscle cell proliferation	0.004416742	Ace2/Tgfb3/Pparg/Xbp1/Ang/Igfbp3/Adipoq/Apod/Htr1b/Agt	10	BP
GO:0008643	carbohydrate transport	0.004438091	RGD1304770/Slc2a10/Trarg1/Aqp1/Abcb1a/Klf15/Adipoq/Itln1/Gpc3	9	BP
GO:0042755	eating behavior	0.004464088	Nmu/Lepr/Oxtr/Cckar/Pyy	5	BP
GO:0001820	serotonin secretion	0.004605805	Xbp1/Htr1b/Fcer1a	3	BP
GO:0001991	regulation of systemic arterial blood pressure by circulatory renin-angiotensin	0.004605805	Pcsk5/Agtr1b/Agt	3	BP
GO:0006534	cysteine metabolic process	0.004605805	Cth/Cbs/Cdo1	3	BP
GO:0043651	linoleic acid metabolic process	0.004605805	Cyp2c24/Cyp4a1/Gsta1	3	BP
GO:0072074	kidney mesenchyme development	0.004605805	Shh/Six4/Tcf21	3	BP
GO:0086013	membrane repolarization during cardiac muscle cell	0.004605805	Kcnh2/Kcnj5/Kcne3	3	BP

	action potential				
GO:1905941	positive regulation of gonad development	0.004605805	Zfpm2/Abcb1a/Cftr	3	BP
GO:0015918	sterol transport	0.004629335	Shh/Egf/Apob/Npc1l1/Syt7/Adipoq/Cftr	7	BP
GO:1905952	regulation of lipid localization	0.004641748	Shh/Egf/Apob/Pparg/Bmp6/Lpl/Igfbp3/Adipoq/Agt	9	BP
GO:0019221	cytokine-mediated signaling pathway	0.004646385	Grem2/Ccl24/Pf4/Pparg/Lepr/Ackr2/Ccr3/Il5ra/Prlr/Tnfrsf18/Adipoq/Ccl11/Krt18/Padi2/Edn2/Trem2	16	BP
GO:0010633	negative regulation of epithelial cell migration	0.00471156	Dcn/Pparg/Serpinf1/Mmrn2/Meox2/Rgcc	6	BP
GO:0071549	cellular response to dexamethasone stimulus	0.00471156	Srd5a1/Aqp1/Serpinf1/Pck1/Abcb1a/Agtr1b	6	BP
GO:0032102	negative regulation of response to external stimulus	0.004712547	Ptgis/Gcg/Slit1/Cd34/Thbd/Pparg/Serpinf1/Cpb2/F2/Isl1/Pyy/Klk8/Adipoq/Apod/Padi2	15	BP
GO:0071248	cellular response to metal ion	0.004902914	Fbp1/Shh/Aqp1/Snca/Hsd17b2/Pparg/Serpinf1/Tf/Bmp6/Syt7/Slc18a1/Fabp4	12	BP
GO:0016042	lipid catabolic process	0.005000694	Srd5a1/Ces1e/Fmc1/Plin1/Pnpla3/Apob/Ces1d/Pck1/Lpl/Phyh/Cps1/Prdx6/Adipoq/Aadac	14	BP
GO:0009064	glutamine family amino acid metabolic process	0.005041857	Pycr1/Aadat/Adhfe1/Oat/Fah/Cps1	6	BP
GO:0046660	female sex differentiation	0.00518537	Srd5a1/Zfpm2/Fzd4/Dmrta1/Tp63/Sfrp1/Nupr1/Lepr/Edn2/Agt	10	BP
GO:0010959	regulation of metal ion transport	0.005192721	Kcnh2/Rgn/Gcg/Egf/Kcns1/Fxyd3/Snca/Trdn/Spink3/Tf/Fxyd1/Cckar/F2/Tspan13/Kcne3/Lrrc26/Ms4a2/Agt	18	BP
GO:0032869	cellular response to insulin stimulus	0.005243966	Srd5a1/Fbp1/Trarg1/Pparg/Pck1/Xbp1/Pklr/Adipoq/Aldob/Igfbp1/Hmgcs2/Agt	12	BP
GO:0044282	small molecule catabolic process	0.005270081	Adh5/Aadat/Aldh1b1/Sord/Thnsl2/Adhfe1/Oat/Ces1d/Pck1/Phyh/Fah/Cbs/Bcat2/Adipoq/Cdo1	15	BP
GO:0046676	negative regulation of insulin secretion	0.005286528	Chga/Pim3/Sfrp1/Sytl4/Bmp8a	5	BP
GO:0032526	response to retinoic acid	0.005296598	Shh/Aqp1/Hsd17b2/Pparg/Serpinf1/Pck1/Gata4/Bmp6/Sftpa1	9	BP
GO:0010565	regulation of cellular ketone metabolic process	0.005415093	Rgn/Snca/Pparg/Avpr1a/Ugt1a1/Bmp6/Adipoq/Agt	8	BP
GO:0043537	negative regulation of blood vessel endothelial cell migration	0.005438852	Pparg/Mmrn2/Meox2/Rgcc	4	BP
GO:0031668	cellular response to extracellular stimulus	0.005460466	Srd5a1/Foxa3/Sfrp1/Pparg/Avpr1a/Pck1/Xbp1/Abcb1a/Asgr1/Wnt2b/Rnase4/Cbs/Aldob/Ltc4s	14	BP
GO:0071322	cellular response to carbohydrate stimulus	0.005487555	Gcg/Neurod1/Pim3/Lepr/Serpinf1/Pck1/Xbp1/Ptprn2/Gata4/Cpb2/Cftr	11	BP

GO:0006677	glycosylceramide metabolic process	0.00550367	Ugt8/Gal3st1/B3galt1	3	BP
GO:0007202	activation of phospholipase C activity	0.00550367	Ang/Ms4a2/Agt	3	BP
GO:0043084	penile erection	0.00550367	Shh/Avpr1a/Oxtr	3	BP
GO:0045019	negative regulation of nitric oxide biosynthetic process	0.00550367	Rgn/Ptgis/Cd34	3	BP
GO:1903779	regulation of cardiac conduction	0.00550367	Ace2/Trdn/Agt	3	BP
GO:1904406	negative regulation of nitric oxide metabolic process	0.00550367	Rgn/Ptgis/Cd34	3	BP
GO:0006936	muscle contraction	0.005621073	Chga/Kcnh2/Ace2/Kcnj5/Nmu/Tacr3/Gata4/Oxtr/Fxyd1/Kcne3/Myom2/Edn2/Sstr2/Agt	14	BP
GO:0032094	response to food	0.005735509	Gcg/Pparg/G6pc/Cps1/Pyy	5	BP
GO:0006094	gluconeogenesis	0.005752965	Gcg/Fbp1/Lepr/Pck1/G6pc/Adipoq	6	BP
GO:0034765	regulation of ion transmembrane transport	0.005947614	Kcnh2/Rgn/Slc9a3r2/Kcnj5/Kcns1/Fxyd3/Snca/Trdn/Kcnv2/Abcb1a/Chrna4/Fxyd1/F2/Tspan13/Kcne3/Cftr/Lrrc26/Ms4a2/Agt	19	BP
GO:0006066	alcohol metabolic process	0.005953687	Adh5/Ephx1/Aldh1b1/Sord/Snca/Apob/Ces1d/Lepr/Pck1/Bmp6/Npc1l1/Cftr/Rbp2/Hmgcs2	14	BP
GO:0002792	negative regulation of peptide secretion	0.006020835	Chga/Pim3/Fbln1/Cd34/Sfrp1/Pparg/Sytl4/Bmp8a/Rgcc	9	BP
GO:0000096	sulfur amino acid metabolic process	0.006060302	Cth/Cps1/Cbs/Cdo1	4	BP
GO:0060008	Sertoli cell differentiation	0.006060302	Tcf21/Abcb1a/Gata4/Cftr	4	BP
GO:0110110	positive regulation of animal organ morphogenesis	0.006119499	Six4/Cd34/Xbp1/Wnt2b/Agtr1b/Maged1/Agt	7	BP
GO:0007623	circadian rhythm	0.006179684	Srd5a1/Dbp/Nmu/Pparg/Lepr/Abcb1a/Prokr2/Adipoq/Ptgds/Maged1/Sftpa1/Adk	12	BP
GO:0016126	sterol biosynthetic process	0.006210705	Apob/Ces1d/Npc1l1/Cftr/Hmgcs2	5	BP
GO:0035567	non-canonical Wnt signaling pathway	0.006210705	Sfrp4/Fzd4/Sfrp1/Fzd10/Gpc3	5	BP
GO:0043255	regulation of carbohydrate biosynthetic process	0.006456036	Gcg/Fbp1/Egf/Esrrb/Snca/Lepr/Adipoq	7	BP
GO:0045921	positive regulation of exocytosis	0.006456036	Rab27a/Rab27b/Sytl4/Syt7/Fcer1a/Cftr/Ms4a2	7	BP
GO:0006688	glycosphingolipid biosynthetic process	0.006499312	Ugt8/Gal3st1/B3galt1	3	BP
GO:0006833	water transport	0.006499312	Aqp1/Aqp8/Cftr	3	BP

GO:0060572	morphogenesis of an epithelial bud	0.006499312	Shh/Tp63/Wnt2b	3	BP
GO:1901317	regulation of flag-ellated sperm motility	0.006499312	Rgn/Tac4/Tacr3	3	BP
GO:0031100	animal organ re-generation	0.006544507	Nr0b2/Rgn/Shh/Serpina10/Pparg/Ugt1a1/Cpb2/Cckar/Cftr	9	BP
GO:0042743	hydrogen peroxide metabolic process	0.006712888	Hbb/Hba-a1/Snca/Prdx6/Hba-a2	5	BP
GO:0042310	vasoconstriction	0.006806159	Klk1c9/Avpr1a/Oxtr/Htr1b/Agtr1b/Edn2/Agt	7	BP
GO:0031589	cell-substrate ad-hesion	0.00690024	Pcsk5/Fat2/Fzd4/Agr2/Fbln1/Cd34/Spock1/Bcam/Mmrn2/Nexmif/Itgbl1/Ccdc80/Emid1/Apod/Agt	15	BP
GO:0030324	lung development	0.007019574	Zfpm2/Agr2/Shh/Tcf21/Tgfb3/Gata4/Wnt2b/Cftr/Edn2/Hmgcs2/Spdef/Gpc3	12	BP
GO:0042445	hormone metabolic process	0.007241788	Srd5a1/Pcsk5/Crabp2/Shh/Hsd17b2/Bmp6/Selenom/Cpq/Rbp2/Pcsk6/Agt	11	BP
GO:1901654	response to ketone	0.007383669	Srd5a1/Ramp2/Tgfb3/Aqp1/Sfrp1/Pparg/Avpr1a/Lepr/Serpinf1/Pck1/Abcb1a/Oxtr/Cps1/Agtr1b/Cftr/Hmgcs2	16	BP
GO:0001702	gastrulation with mouth forming second	0.007440967	Nat8f3/Gata4/Nat8f5/Nat8f1	4	BP
GO:0045987	positive regulation of smooth muscle contraction	0.007440967	Nmu/Tacr3/Oxtr/Edn2	4	BP
GO:0048566	embryonic diges-tive tract develop-ment	0.007440967	Pcsk5/Shh/Tcf21/Gata4	4	BP
GO:1903959	regulation of anion transmembrane transport	0.007440967	Abcb1a/Fxyd1/Cftr/Agt	4	BP
GO:1904037	positive regulation of epithelial cell apoptotic process	0.007440967	Sfrp4/Cd248/Xbp1/Rgcc	4	BP
GO:0030336	negative regulation of cell migration	0.007524722	Dcn/Shh/Sfrp1/Pparg/Serpinf1/Mmrn2/Nexmif/Igfbp3/Adipoq/Meox2/Apod/Rgcc/Padi2	13	BP
GO:0003215	cardiac right ven-tricle morphogene-sis	0.007595261	Zfpm2/Gata4/Isl1	3	BP
GO:0006103	2-oxoglutarate metabolic process	0.007595261	Aadat/Adhfe1/Phyh	3	BP
GO:0021794	thalamus develop-ment	0.007595261	Srd5a1/Shh/Tal2	3	BP
GO:0030157	pancreatic juice secretion	0.007595261	Aqp1/Spink3/Cckar	3	BP
GO:0061318	renal filtration cell differentiation	0.007595261	Prom1/Klf15/Adipoq	3	BP
GO:0070365	hepatocyte differ-entiation	0.007595261	Foxa3/Pck1/Cps1	3	BP
GO:0071871	response to epi-	0.007595261	Srd5a1/Pklr/Adipoq	3	BP

	nephrine				
GO:0072112	glomerular visceral epithelial cell differentiation	0.007595261	Prom1/Klf15/Adipoq	3	BP
GO:0086011	membrane repolarization during action potential	0.007595261	Kcnh2/Kcnj5/Kcne3	3	BP
GO:1904754	positive regulation of vascular associated smooth muscle cell migration	0.007595261	Pcsk5/Xbp1/Agt	3	BP
GO:0006520	cellular amino acid metabolic process	0.007745225	Pycr1/Cth/Aadat/Acy1/Thnsl2/Adhfe1/Oat/Fah/Cps1/Cbs/Bcat2/Cdo1/Gatm	13	BP
GO:0001936	regulation of endothelial cell proliferation	0.007842555	Ccl24/Pparg/Ccr3/Ang/Bmp6/Tnmd/Ccl11/Rgcc	8	BP
GO:0030317	flagellated sperm motility	0.007941271	Slc9c1/Rgn/Sord/Tac4/Tacr3/Apob/Tekt1	7	BP
GO:1904064	positive regulation of cation transmembrane transport	0.008006865	Kcnh2/Rgn/Snca/Trdn/Fxyd1/F2/Kcne3/Lrrc26/Agt	9	BP
GO:0070838	divalent metal ion transport	0.008249736	Jph1/Ramp2/Rgn/Slc39a8/Gcg/Slc30a2/Egf/Snca/Trdn/Spink3/Slc30a8/Chrna4/Ramp1/Cckar/F2/Tspan13/Kcne3/Ms4a2/Agt	19	BP
GO:0097722	sperm motility	0.008348962	Slc9c1/Rgn/Sord/Tac4/Tacr3/Apob/Tekt1	7	BP
GO:0006956	complement activation	0.008388913	Masp1/Fcna/Cfh/Rgcc/Cfd	5	BP
GO:0044242	cellular lipid catabolic process	0.008426506	Pnpla3/Apob/Ces1d/Pck1/Lpl/Phyh/Cps1/Prdx6/Adipoq/Aadac	10	BP
GO:0007189	adenylate cyclase-activating G protein-coupled receptor signaling pathway	0.008560245	Chga/Ramp2/Adgrg2/Mc2r/Gcg/Pf4/Ramp1/Mrap2	8	BP
GO:0019933	cAMP-mediated signaling	0.008728647	Chga/Ramp2/Adgrg2/Mc2r/Gcg/Pf4/Ramp1/Mrap2/Pex5l/Pde2a	10	BP
GO:0072511	divalent inorganic cation transport	0.008786966	Jph1/Ramp2/Rgn/Slc39a8/Gcg/Slc30a2/Egf/Snca/Trdn/Spink3/Slc30a8/Chrna4/Ramp1/Cckar/F2/Tspan13/Kcne3/Ms4a2/Agt	19	BP
GO:0033605	positive regulation of catecholamine secretion	0.008793736	Chrna4/Oxtr/Slc18a1	3	BP
GO:0035930	corticosteroid hormone secretion	0.008793736	Bmp6/Selenom/Agt	3	BP
GO:0048245	eosinophil chemotaxis	0.008793736	Ccl24/Ccr3/Ccl11	3	BP
GO:0048557	embryonic digestive tract morphogenesis	0.008793736	Shh/Tcf21/Gata4	3	BP
GO:0060456	positive regulation of digestive system	0.008793736	Tac4/Aqp1/Spink3	3	BP

	process				
GO:0072311	glomerular epithelial cell differentiation	0.008793736	Prom1/Klf15/Adipoq	3	BP
GO:2000353	positive regulation of endothelial cell apoptotic process	0.008793736	Cd248/Xbp1/Rgcc	3	BP
GO:0042632	cholesterol homeostasis	0.008822374	Apob/Xbp1/G6pc/Lpl/Npc1l1/Fabp4	6	BP
GO:0045913	positive regulation of carbohydrate metabolic process	0.008822374	Rgn/Gcg/Egf/Esrrb/Snca/Avpr1a	6	BP
GO:0016101	diterpenoid metabolic process	0.009006542	Srd5a1/Adh5/Crabp2/Ces2a/Rbp2	5	BP
GO:0072593	reactive oxygen species metabolic process	0.009177576	Rab27a/Rgn/Hbb/Ptgis/Hba-a1/Cd34/Snca/F2/Cps1/Prdx6/Cbs/Hba-a2/Agt	13	BP
GO:0006090	pyruvate metabolic process	0.009210255	Dhtkd1/Fbp1/Esrrb/Pck1/Pklr/Aldob/Me3	7	BP
GO:0032868	response to insulin	0.009220707	Srd5a1/Fbp1/Trarg1/Pparg/Pck1/Xbp1/Igfbp3/Klf15/Pklr/Adipoq/Aldob/Igfbp1/Hmgcs2/Agt	14	BP
GO:0002064	epithelial cell development	0.009238217	Tp63/Xbp1/Abcb1a/Bmp6/Tnmd/Pde2a/Adipoq/Cftr/Ros1/Col23a1/Agt/Spdef	12	BP
GO:0055088	lipid homeostasis	0.009326008	Pnpla3/Apob/Xbp1/G6pc/Lpl/Asgr2/Npc1l1/Fabp4	8	BP
GO:0055092	sterol homeostasis	0.009340869	Apob/Xbp1/G6pc/Lpl/Npc1l1/Fabp4	6	BP
GO:0019935	cyclic-nucleotide-mediated signaling	0.00940614	Chga/Ramp2/Adgrg2/Mc2r/Gcg/Pf4/Aqp1/Ramp1/Mrap2/Pex5l/Pde2a	11	BP
GO:0071375	cellular response to peptide hormone stimulus	0.009599105	Srd5a1/Fbp1/Trarg1/Pparg/Pck1/Spink3/Xbp1/Cps1/Pklr/Adipoq/Aldob/Agtr1b/Igfbp1/Hmgcs2/Agt	15	BP
GO:0010812	negative regulation of cell-substrate adhesion	0.009654848	Fzd4/Fbln1/Spock1/Nexmif/Apod	5	BP
GO:0033144	negative regulation of intracellular steroid hormone receptor signaling pathway	0.009875906	Tcf21/Tp63/Sfrp1/Isl1	4	BP
GO:0008202	steroid metabolic process	0.009967635	Srd5a1/Calb1/Shh/Hsd17b2/Apob/Ces1d/Lepr/Bmp6/G6pc/Npc1l1/Cftr/Hmgcs2/Agt	13	BP
GO:0032400	melanosome localization	0.010096663	Rab27a/Rab27b/Mlph	3	BP
GO:0042053	regulation of dopamine metabolic process	0.010096663	Gpr37/Snca/Tacr3	3	BP
GO:0045723	positive regulation of fatty acid biosynthetic process	0.010096663	Rgn/Avpr1a/Agt	3	BP
GO:1903055	positive regulation of extracellular	0.010096663	Cpb2/Rgcc/Agt	3	BP

	matrix organization				
GO:1904706	negative regulation of vascular associated smooth muscle cell proliferation	0.010096663	Tgfb3/Pparg/Adipoq	3	BP
GO:0071241	cellular response to inorganic substance	0.010242473	Fbp1/Shh/Aqp1/Snca/Hsd17b2/Pparg/Serpinf1/Tf/Bmp6/Syt7/Slc18a1/Fabp4/Sftpa1	13	BP
GO:0048145	regulation of fibroblast proliferation	0.010621746	Egf/Fbln1/Aqp1/Sfrp1/Nupr1/Pparg/Agt	7	BP
GO:0009100	glycoprotein metabolic process	0.010758997	Fut9/Dcn/Chst13/Dpm3/B3gnt6/Galnt5/Ramp1/St3gal3/Asgr2/Cytl1/Bace2/St3gal1/B3galt1/Pcsk6	14	BP
GO:0034694	response to prostaglandin	0.010789446	Sfrp1/Apob/Pparg/Hmgcs2	4	BP
GO:0046326	positive regulation of glucose import	0.010789446	Klf15/Adipoq/Itln1/Gpc3	4	BP
GO:0098664	G protein-coupled serotonin receptor signaling pathway	0.010789446	Hrh4/Chrm1/Htr1b/Htr5b	4	BP
GO:0098656	anion transmembrane transport	0.010997149	Slc38a11/Slc16a7/Slc1a7/Slc16a12/Slc26a7/Slc4a10/Abcb1a/Fxyd1/Ankh/Slc12a8/Cftr/Agt	12	BP
GO:0016052	carbohydrate catabolic process	0.011009234	Dhtkd1/Sord/Fbp1/Esrrb/Avpr1a/G6pc/Pklr/Aldob	8	BP
GO:0019217	regulation of fatty acid metabolic process	0.011026513	Rgn/Snca/Pparg/Avpr1a/Adipoq/Agt	6	BP
GO:0034103	regulation of tissue remodeling	0.011026513	Sfrp1/Lepr/Tf/Gata4/Syt7/Agt	6	BP
GO:1904035	regulation of epithelial cell apoptotic process	0.011026513	Ramp2/Sfrp4/Neurod1/Cd248/Xbp1/Rgcc	6	BP
GO:0030850	prostate gland development	0.011046268	Shh/Tp63/Sfrp1/Serpinf1/Prlr	5	BP
GO:0086003	cardiac muscle cell contraction	0.011046268	Kcnh2/Kcnj5/Gata4/Fxyd1/Kcne3	5	BP
GO:0043062	extracellular structure organization	0.011102583	Ramp2/Col14a1/Fbln1/Cd34/Apob/Ces1d/Cpb2/Lpl/Lum/Ccdc80/Rgcc/Col23a1/Agt	13	BP
GO:0048678	response to axon injury	0.011125389	Shh/Gstm1/Isl1/Flrt3/Klk8/Ltc4s/Apod	7	BP
GO:1904019	epithelial cell apoptotic process	0.011125389	Ramp2/Sfrp4/Neurod1/Cd248/Xbp1/Rgcc/Krt18	7	BP
GO:0030879	mammary gland development	0.011454535	Sfrp4/Egf/Tgfb3/Xbp1/Abcb1a/Prlr/Bcat2/Ccl11/Cdo1/Etv5	10	BP
GO:0002065	columnar/cuboidal epithelial cell differentiation	0.011463005	Neurod1/Agr2/Tp63/Gata4/Bmp6/Cftr/Ros1/Spdef	8	BP
GO:0031290	retinal ganglion	0.011505686	Slit1/Isl1/Isl2	3	BP

	cell axon guidance				
GO:0032800	receptor biosynthetic process	0.011505686	Ace2/Pparg/Adipoq	3	BP
GO:0042069	regulation of catecholamine metabolic process	0.011505686	Gpr37/Snca/Tacr3	3	BP
GO:0051580	regulation of neurotransmitter uptake	0.011505686	Snca/Slc18a1/Nat8l	3	BP
GO:0051875	pigment granule localization	0.011505686	Rab27a/Rab27b/Mlph	3	BP
GO:0071577	zinc ion transmembrane transport	0.011505686	Slc39a8/Slc30a2/Slc30a8	3	BP
GO:0006692	prostanoid metabolic process	0.011755794	Ptgis/Avpr1a/Ptgds/Edn2	4	BP
GO:0006693	prostaglandin metabolic process	0.011755794	Ptgis/Avpr1a/Ptgds/Edn2	4	BP
GO:0009069	serine family amino acid metabolic process	0.011755794	Cth/Thnsl2/Cbs/Cdo1	4	BP
GO:0043112	receptor metabolic process	0.011836001	Ramp2/Sfrp4/Ace2/Egf/Snca/Pparg/Ramp1/Adipoq/Htr1b/Rep15	10	BP
GO:0048144	fibroblast proliferation	0.01218427	Egf/Fbln1/Aqp1/Sfrp1/Nupr1/Pparg/Agt	7	BP
GO:0006855	drug transmembrane transport	0.01226321	Slc1a7/Slc16a12/Aqp1/Abcb1a/Slc18a1/Agt	6	BP
GO:0016485	protein processing	0.012403893	Pcsk5/Masp1/Klk1b3/Shh/Klk1c9/Cpxm2/Cpb2/Cfh/Cpn1/Klk10/Pcsk6	11	BP
GO:0050709	negative regulation of protein secretion	0.012411724	Chga/Pim3/Fbln1/Cd34/Sfrp1/Sytl4/Bmp8a/Rgcc	8	BP
GO:0007210	serotonin receptor signaling pathway	0.012775984	Hrh4/Chrm1/Htr1b/Htr5b	4	BP
GO:0072210	metanephric nephron development	0.012775984	Tcf21/Cd34/Aqp1/Adipoq	4	BP
GO:0032024	positive regulation of insulin secretion	0.012916751	Nr0b2/Gcg/Lepr/Slc30a8/Isl1/Cftr	6	BP
GO:0010874	regulation of cholesterol efflux	0.013022189	Shh/Egf/Adipoq	3	BP
GO:0015701	bicarbonate transport	0.013022189	Slc26a7/Slc4a10/Cftr	3	BP
GO:0095500	acetylcholine receptor signaling pathway	0.013022189	Hrh4/Chrm1/Chrna4	3	BP
GO:1903831	signal transduction involved in cellular response to ammonium ion	0.013022189	Hrh4/Chrm1/Chrna4	3	BP
GO:1905144	response to acetyl-	0.013022189	Hrh4/Chrm1/Chrna4	3	BP

	choline				
GO:1905145	cellular response to acetylcholine	0.013022189	Hrh4/Chrm1/Chrna4	3	BP
GO:0042698	ovulation cycle	0.013313999	Chga/Fzd4/Lepr/Serpinf1/Oxtr/Edn2/Agt	7	BP
GO:0006575	cellular modified amino acid metabolic process	0.013431442	Cth/Hbb/Chdh/Gstm5/Gstm1/Mgst1/Cpq/Gatm/Gsta1	9	BP
GO:0051235	maintenance of location	0.013435134	Scin/Jph1/Slc30a2/Ffar2/Snca/Apob/Trdn/Pparg/Slc30a8/Lpl/F2/Pex5l/Pfn4/Ms4a2	14	BP
GO:1905953	negative regulation of lipid localization	0.013850993	Shh/Egf/Pparg/Igfbp3	4	BP
GO:0048771	tissue remodeling	0.013889074	Sfrp4/Tgfb3/Sfrp1/Lepr/Tf/Gata4/Syt7/Cbs/Htr1b/Agt	10	BP
GO:0090287	regulation of cellular response to growth factor stimulus	0.014107339	Dcn/Sfrp4/Grem2/Fzd4/Tgfb3/Sfrp1/Xbp1/Mmrn2/Gata4/Dok5/Agt/Gpc3	12	BP
GO:0050679	positive regulation of epithelial cell proliferation	0.014329832	Ccl24/Shh/Tp63/Sfrp1/Spink3/Xbp1/Ccr3/Ang/Bmp6/Ccl11	10	BP
GO:1904062	regulation of cation transmembrane transport	0.014433194	Kcnh2/Rgn/Kcns1/Fxyd3/Snca/Trdn/Chrna4/Fxyd1/F2/Tspan13/Kcne3/Lrrc26/Ms4a2/Agt	14	BP
GO:0090090	negative regulation of canonical Wnt signaling pathway	0.014517004	Sfrp4/Shh/Sfrp1/Dkk2/Isl1/Dact3/Gpc3	7	BP
GO:0010640	regulation of platelet-derived growth factor receptor signaling pathway	0.014647301	Snca/Adipoq/Apod	3	BP
GO:0072202	cell differentiation involved in metanephros development	0.014647301	Tcf21/Cd34/Adipoq	3	BP
GO:0072243	metanephric nephron epithelium development	0.014647301	Calb1/Aqp1/Adipoq	3	BP
GO:0072677	eosinophil migration	0.014647301	Ccl24/Ccr3/Ccl11	3	BP
GO:0071453	cellular response to oxygen levels	0.014780969	Ptgis/Cd34/Aqp1/Sfrp1/Clca1/Pparg/Pck1/Cbs/Rgcc/Cftr	10	BP
GO:0042738	exogenous drug catabolic process	0.014981743	Cyp4b1/Cyp2d5/Cyp2d2/Cyp2c24	4	BP
GO:0060416	response to growth hormone	0.014981743	Srd5a1/Igfbp3/Cps1/Hmgcs2	4	BP
GO:0070207	protein homotrimerization	0.014981743	Scara5/Mgst1/Itln1/Col23a1	4	BP
GO:0001505	regulation of neurotransmitter levels	0.015008635	Srd5a1/Rgn/Chdh/Ptgis/Slc1a7/Cd34/Snca/Xbp1/Chrna4/Ptprn2/Cps1/Syt7/Slc18a1/Htr1b/Nat8l/Agt	16	BP
GO:0097421	liver regeneration	0.015108677	Rgn/Shh/Serpina10/Cpb2/Cftr	5	BP

GO:1901655	cellular response to ketone	0.015390618	Srd5a1/Aqp1/Sfrp1/Pparg/Serpinf1/Pck1/Abcb1a/Agtr1b/Cftr	9	BP
GO:0043500	muscle adaptation	0.015795666	Pi16/Col14a1/Gata4/Klf15/Gatm/Adk/Agt	7	BP
GO:1904659	glucose transmembrane transport	0.015795666	RGD1304770/Slc2a10/Trarg1/Klf15/Adipoq/Itln1/Gpc3	7	BP
GO:0010676	positive regulation of cellular carbohydrate metabolic process	0.016026226	Rgn/Gcg/Esrrb/Snca/Avpr1a	5	BP
GO:0003298	physiological muscle hypertrophy	0.016169099	Pi16/Col14a1/Gata4/Agt	4	BP
GO:0003301	physiological cardiac muscle hypertrophy	0.016169099	Pi16/Col14a1/Gata4/Agt	4	BP
GO:0010677	negative regulation of cellular carbohydrate metabolic process	0.016169099	Fbp1/Lepr/Ugt1a1/Adipoq	4	BP
GO:0035850	epithelial cell differentiation involved in kidney development	0.016169099	Cd34/Prom1/Klf15/Adipoq	4	BP
GO:0061049	cell growth involved in cardiac muscle cell development	0.016169099	Pi16/Col14a1/Gata4/Agt	4	BP
GO:0008209	androgen metabolic process	0.016381916	Srd5a1/Shh/Hsd17b2	3	BP
GO:0014821	phasic smooth muscle contraction	0.016381916	Edn2/Sstr2/Agt	3	BP
GO:0034104	negative regulation of tissue remodeling	0.016381916	Sfrp1/Gata4/Agt	3	BP
GO:0042538	hyperosmotic salinity response	0.016381916	Aqp1/Tacr3/Abcb1a	3	BP
GO:0043171	peptide catabolic process	0.016381916	Trhde/Cpn1/Cpq	3	BP
GO:0071379	cellular response to prostaglandin stimulus	0.016381916	Sfrp1/Apob/Pparg	3	BP
GO:2000050	regulation of non-canonical Wnt signaling pathway	0.016381916	Sfrp4/Sfrp1/Gpc3	3	BP
GO:0060070	canonical Wnt signaling pathway	0.016526043	Sfrp4/Fzd4/Shh/Egf/Sfrp1/Fzd10/Dkk2/Gata4/Wnt2b/Isl1/Dact3/Gpc3	12	BP
GO:0006813	potassium ion transport	0.016533057	Kcnh2/Kcnj5/Aqp1/Kcns1/Fxyd3/Kcnv2/Fxyd1/Cckar/Kcne3/Slc12a8/Lrrc26	11	BP
GO:0043506	regulation of JUN kinase activity	0.01655317	Fzd4/Sfrp1/Tf/Fzd10/Cbs/Fcer1a	6	BP

GO:0008585	female gonad development	0.016789021	Zfpm2/Fzd4/Dmrta1/Sfrp1/Nupr1/Lepr/Edn2/Agt	8	BP
GO:0070206	protein trimerization	0.016980155	Scara5/Mgst1/Adipoq/Itln1/Col23a1	5	BP
GO:1901653	cellular response to peptide	0.017017774	Srd5a1/Fbp1/Trarg1/Pparg/Pck1/Spink3/Xbp1/Cps1/Klf15/Pklr/Adipoq/Aldob/Agtr1b/Igfbp1/Hmgcs2/Agt	16	BP
GO:0043200	response to amino acid	0.017197241	Gcg/Pck1/Xbp1/Abcb1a/F2/Gstm1/Cps1/Aldob/Cdo1/Hmgcs2	10	BP
GO:0000041	transition metal ion transport	0.017357024	Slc39a8/Slc30a2/Tf/Slc30a8/Scara5/Rep15	6	BP
GO:0046916	cellular transition metal ion homeostasis	0.017357024	Slc39a8/Slc30a2/Tf/Slc30a8/Bmp6/Scara5	6	BP
GO:0007517	muscle organ development	0.017371896	Jph1/Dcn/Zfpm2/Pi16/Shh/Six4/Col14a1/Tcf21/Fgfrl1/Nupr1/Xbp1/Gata4/Isl1/Myom2/Meox2/Hlf	16	BP
GO:0051924	regulation of calcium ion transport	0.017398074	Rgn/Gcg/Egf/Snca/Trdn/Spink3/Cckar/F2/Tspan13/Kcne3/Ms4a2/Agt	12	BP
GO:0006694	steroid biosynthetic process	0.01740539	Srd5a1/Hsd17b2/Apob/Ces1d/Bmp6/Npc1l1/Cftr/Hmgcs2	8	BP
GO:0006865	amino acid transport	0.01740539	Slc38a11/Ace2/Slc1a7/Slc16a12/Snca/Avpr1a/Slc6a19/Agt	8	BP
GO:0010907	positive regulation of glucose metabolic process	0.017413869	Rgn/Gcg/Esrrb/Avpr1a	4	BP
GO:0030431	sleep	0.017413869	Srd5a1/Nmu/Oxtr/Ptgds	4	BP
GO:0032965	regulation of collagen biosynthetic process	0.017413869	Tgfb3/Pparg/F2/Rgcc	4	BP
GO:0060359	response to ammonium ion	0.017552392	Hrh4/Tac3/Snca/Tacr3/Chrm1/Abcb1a/Chrna4/Oxtr/Htr1b	9	BP
GO:0008645	hexose transmembrane transport	0.017860596	RGD1304770/Slc2a10/Trarg1/Klf15/Adipoq/Itln1/Gpc3	7	BP
GO:0043949	regulation of cAMP-mediated signaling	0.017971007	Chga/Pf4/Mrap2/Pex5l/Pde2a	5	BP
GO:0045576	mast cell activation	0.017971007	Chga/Tac4/Fcer1a/Cftr/Ms4a2	5	BP
GO:0003071	renal system process involved in regulation of systemic arterial blood pressure	0.018226702	Pcsk5/Agtr1b/Agt	3	BP
GO:0006817	phosphate ion transport	0.018226702	Sfrp4/Ankh/Ros1	3	BP
GO:0006882	cellular zinc ion homeostasis	0.018226702	Slc39a8/Slc30a2/Slc30a8	3	BP
GO:0009065	glutamine family amino acid catabolic process	0.018226702	Adhfe1/Oat/Fah	3	BP
GO:0010863	positive regulation	0.018226702	Ang/Ms4a2/Agt	3	BP

	of phospholipase C activity				
GO:0015874	norepinephrine transport	0.018226702	Snca/Oxtr/Agt	3	BP
GO:0030539	male genitalia development	0.018226702	Srd5a1/Shh/Bmp6	3	BP
GO:0042044	fluid transport	0.018226702	Aqp1/Aqp8/Cftr	3	BP
GO:0019216	regulation of lipid metabolic process	0.018588703	Rgn/Gcg/Fmc1/Snca/Apob/Pparg/Avpr1a/Bmp6/F2/Thrsp/Adipoq/Apod/Aadac/Agt	14	BP
GO:0003158	endothelium development	0.018589219	Tgfb3/Cd34/Bmp6/Tnmd/Pde2a/Col23a1/Agt	7	BP
GO:0060048	cardiac muscle contraction	0.018589219	Chga/Kcnh2/Ace2/Kcnj5/Gata4/Fxyd1/Kcne3	7	BP
GO:0001504	neurotransmitter uptake	0.018716804	Slc1a7/Snca/Slc18a1/Nat8l	4	BP
GO:0006636	unsaturated fatty acid biosynthetic process	0.018716804	Ptgis/Avpr1a/Ptgds/Edn2	4	BP
GO:0007431	salivary gland development	0.018716804	Edar/Shh/Tgfb3/Xbp1	4	BP
GO:0030195	negative regulation of blood coagulation	0.018716804	Cd34/Thbd/Cpb2/F2	4	BP
GO:0032892	positive regulation of organic acid transport	0.018716804	Ace2/Avpr1a/Fxyd1/Agt	4	BP
GO:0033344	cholesterol efflux	0.018716804	Shh/Egf/Apob/Adipoq	4	BP
GO:0018879	biphenyl metabolic process	0.018847072	Cyp4b1/Ugt1a1	2	BP
GO:0021940	positive regulation of cerebellar granule cell precursor proliferation	0.018847072	Shh/Egf	2	BP
GO:0030388	fructose 1,6-bisphosphate metabolic process	0.018847072	Fbp1/Aldob	2	BP
GO:0060406	positive regulation of penile erection	0.018847072	Shh/Oxtr	2	BP
GO:0060462	lung lobe development	0.018847072	Shh/Gata4	2	BP
GO:0060513	prostatic bud formation	0.018847072	Shh/Tp63	2	BP
GO:0072203	cell proliferation involved in metanephros development	0.018847072	Shh/Gpc3	2	BP
GO:1900103	positive regulation of endoplasmic reticulum unfolded protein response	0.018847072	Agr2/Xbp1	2	BP

GO:2001225	regulation of chloride transport	0.018847072	Abcb1a/Cftr	2	BP
GO:0055117	regulation of cardiac muscle contraction	0.018999306	Chga/Ace2/Gata4/Fxyd1/Kcne3	5	BP
GO:0006165	nucleoside diphosphate phosphorylation	0.019044393	Dhtkd1/Fbp1/Esrrb/Pck1/Pklr/Aldob	6	BP
GO:0015749	monosaccharide transmembrane transport	0.019338458	RGD1304770/Slc2a10/Trarg1/Klf15/Adipoq/Itln1/Gpc3	7	BP
GO:0050729	positive regulation of inflammatory response	0.019338458	Ccl24/Ffar2/Snca/Klk1/Fabp4/Pde2a/Fcer1a	7	BP
GO:0010810	regulation of cell-substrate adhesion	0.019893877	Pcsk5/Fzd4/Agr2/Fbln1/Spock1/Mmrn2/Nexmif/Ccdc80/Emid1/Apod	10	BP
GO:0006959	humoral immune response	0.019927716	Pgc/Masp1/Fcna/Rpl39/Pf4/F2/Cfh/Rgcc/Cfd	9	BP
GO:0010594	regulation of endothelial cell migration	0.020034043	Dcn/Shh/Pparg/Serpinf1/Mmrn2/Meox2/Rgcc/Agt	8	BP
GO:0045834	positive regulation of lipid metabolic process	0.020034043	Rgn/Pparg/Avpr1a/Bmp6/F2/Adipoq/Aadac/Agt	8	BP
GO:0043299	leukocyte degranulation	0.020065555	Chga/Rab27a/Tac4/Fcer1a/Ms4a2	5	BP
GO:1900047	negative regulation of hemostasis	0.020078599	Cd34/Thbd/Cpb2/F2	4	BP
GO:0070542	response to fatty acid	0.020108582	Ffar2/Sfrp1/Apob/Pparg/Cps1/Adipoq/Hmgcs2	7	BP
GO:0007620	copulation	0.020182113	Shh/Avpr1a/Oxtr	3	BP
GO:0045742	positive regulation of epidermal growth factor receptor signaling pathway	0.020182113	Agr2/Egf/Agt	3	BP
GO:1900117	regulation of execution phase of apoptosis	0.020182113	Ptgis/Gcg/Igfbp3	3	BP
GO:0045861	negative regulation of proteolysis	0.02024154	Masp1/Serpina1/Shh/Serpina10/Aqp1/Snca/Spock1/Serpinf1/Spink3/Cpb2/F2/Pzp/Cst6/Agt/Gpc3	15	BP
GO:0001659	temperature homeostasis	0.020556169	Nmu/Lepr/Prlr/Oxtr/Cckar/Fabp4/Adipoq/Gatm/Edn2	9	BP
GO:0036294	cellular response to decreased oxygen levels	0.020556169	Ptgis/Cd34/Aqp1/Sfrp1/Clca1/Pck1/Cbs/Rgcc/Cftr	9	BP
GO:0006887	exocytosis	0.020665544	Chga/Rab27a/Rab27b/Trarg1/Sytl1/Tac4/Snca/Chrna4/Sytl4/Pex5l/Syt7/Htr1b/Fcer1a/Cftr/Ms4a2	15	BP
GO:0046545	development of primary female	0.020733046	Zfpm2/Fzd4/Dmrta1/Sfrp1/Nupr1/Lepr/Edn2/Agt	8	BP

	sexual characteristics				
GO:0046939	nucleotide phosphorylation	0.020840412	Dhtkd1/Fbp1/Esrrb/Pck1/Pklr/Aldob	6	BP
GO:0034219	carbohydrate transmembrane transport	0.020899855	RGD1304770/Slc2a10/Trarg1/Klf15/Adipoq/Itln1/Gpc3	7	BP
GO:0046717	acid secretion	0.020899855	Slc26a7/Nmu/Snca/Avpr1a/Oxtr/Cckar/Aqp8	7	BP
GO:0006111	regulation of gluconeogenesis	0.021499893	Gcg/Fbp1/Lepr/Adipoq	4	BP
GO:0046688	response to copper ion	0.021499893	Sord/Aqp1/Snca/Aldob	4	BP
GO:0050819	negative regulation of coagulation	0.021499893	Cd34/Thbd/Cpb2/F2	4	BP
GO:0008277	regulation of G protein-coupled receptor signaling pathway	0.021712538	Chga/Ramp2/Snca/Ramp1/Mrap2/F2/Htr1b	7	BP
GO:0001890	placenta development	0.021855764	Dcn/Sfrp4/Ptgis/Mc2r/Esrrb/Hsd17b2/Pparg/Abcb1a/Cyp4a1	9	BP
GO:0006829	zinc ion transport	0.022248404	Slc39a8/Slc30a2/Slc30a8	3	BP
GO:0007205	protein kinase C-activating G protein-coupled receptor signaling pathway	0.022248404	Htr1b/Ms4a2/Edn2	3	BP
GO:0032026	response to magnesium ion	0.022248404	Fbp1/Snca/Bmp6	3	BP
GO:0051589	negative regulation of neurotransmitter transport	0.022248404	Snca/Slc18a1/Htr1b	3	BP
GO:0055069	zinc ion homeostasis	0.022248404	Slc39a8/Slc30a2/Slc30a8	3	BP
GO:0072012	glomerulus vasculature development	0.022248404	Tcf21/Cd34/Aqp1	3	BP
GO:0072207	metanephric epithelium development	0.022248404	Calb1/Aqp1/Adipoq	3	BP
GO:0006560	proline metabolic process	0.022708439	Pycr1/Oat	2	BP
GO:0014824	artery smooth muscle contraction	0.022708439	Edn2/Agt	2	BP
GO:0033603	positive regulation of dopamine secretion	0.022708439	Chrna4/Slc18a1	2	BP
GO:0043587	tongue morphogenesis	0.022708439	Shh/Six4	2	BP
GO:0048617	embryonic foregut morphogenesis	0.022708439	Shh/Gata4	2	BP

GO:0060539	diaphragm development	0.022708439	Tcf21/Fgfrl1	2	BP
GO:0060768	regulation of epithelial cell proliferation involved in prostate gland development	0.022708439	Shh/Serpinf1	2	BP
GO:0061042	vascular wound healing	0.022708439	Cd34/Xbp1	2	BP
GO:0070472	regulation of uterine smooth muscle contraction	0.022708439	Tacr3/Oxtr	2	BP
GO:0071377	cellular response to glucagon stimulus	0.022708439	Pck1/Cps1	2	BP
GO:0097068	response to thyroxine	0.022708439	Abcb1a/F2	2	BP
GO:0098915	membrane repolarization during ventricular cardiac muscle cell action potential	0.022708439	Kcnh2/Kcne3	2	BP
GO:1902093	positive regulation of flagellated sperm motility	0.022708439	Tac4/Tacr3	2	BP
GO:1902459	positive regulation of stem cell population maintenance	0.022708439	Tp63/Esrrb	2	BP
GO:1904016	response to Thyroglobulin triiodothyronine	0.022708439	Igfbp3/F2	2	BP
GO:1905288	vascular associated smooth muscle cell apoptotic process	0.022708439	Pparg/Cftr	2	BP
GO:1905459	regulation of vascular associated smooth muscle cell apoptotic process	0.022708439	Pparg/Cftr	2	BP
GO:0120162	positive regulation of cold-induced thermogenesis	0.022747877	Lepr/Prlr/Oxtr/Fabp4/Adipoq/Gatm	6	BP
GO:0022898	regulation of transmembrane transporter activity	0.022755067	Rgn/Kcns1/Fxyd3/Snca/Trdn/Abcb1a/Chrna4/Fxyd1/Kcne3/Cftr/Lrrc26	11	BP
GO:1901617	organic hydroxy compound biosynthetic process	0.022887851	Gpr37/Ephx1/Snca/Apob/Ces1d/Pck1/Bmp6/Npc1l1/Cftr/Hmgcs2	10	BP
GO:0001523	retinoid metabolic process	0.022981267	Adh5/Crabp2/Ces2a/Rbp2	4	BP
GO:0006953	acute-phase response	0.022981267	Serpina1/Tf/Ugt1a1/F2	4	BP
GO:0007618	mating	0.022981267	Dmrta1/Shh/Avpr1a/Oxtr	4	BP

GO:0043303	mast cell degranulation	0.022981267	Chga/Tac4/Fcer1a/Ms4a2	4	BP
GO:0045912	negative regulation of carbohydrate metabolic process	0.022981267	Fbp1/Lepr/Ugt1a1/Adipoq	4	BP
GO:0046323	glucose import	0.023496737	Trarg1/Klf15/Adipoq/Itln1/Gpc3	5	BP
GO:0070373	negative regulation of ERK1 and ERK2 cascade	0.023496737	Rps6ka6/Ace2/Fbln1/Xbp1/Adipoq	5	BP
GO:1990845	adaptive thermogenesis	0.0237025	Pparg/Lepr/Bmp8a/Prlr/Oxtr/Fabp4/Adipoq/Gatm	8	BP
GO:0061448	connective tissue development	0.02393377	Col14a1/Cd34/Xbp1/Efemp1/Wnt2b/Bmp6/Lum/Tnmd/Cytl1/Cbs/Selenom/Hmgcs2	12	BP
GO:0031645	negative regulation of nervous system process	0.024425638	Tac4/Avpr1a/Klk8	3	BP
GO:0032369	negative regulation of lipid transport	0.024425638	Shh/Egf/Igfbp3	3	BP
GO:0033005	positive regulation of mast cell activation	0.024425638	Fcer1a/Cftr/Ms4a2	3	BP
GO:0050654	chondroitin sulfate proteoglycan metabolic process	0.024425638	Dcn/Chst13/Cytl1	3	BP
GO:0051957	positive regulation of amino acid transport	0.024425638	Ace2/Avpr1a/Agt	3	BP
GO:0060571	morphogenesis of an epithelial fold	0.024425638	Shh/Tp63/Wnt2b	3	BP
GO:1901186	positive regulation of ERBB signaling pathway	0.024425638	Agr2/Egf/Agt	3	BP
GO:0002279	mast cell activation involved in immune response	0.024523249	Chga/Tac4/Fcer1a/Ms4a2	4	BP
GO:0010828	positive regulation of glucose transmembrane transport	0.024523249	Klf15/Adipoq/Itln1/Gpc3	4	BP
GO:0051955	regulation of amino acid transport	0.024523249	Ace2/Snca/Avpr1a/Agt	4	BP
GO:0070741	response to interleukin-6	0.024523249	Ptgis/Pck1/Aldob/Sftpa1	4	BP
GO:0006821	chloride transport	0.024769433	Slc26a7/Clca1/Ano4/Abcb1a/Slc12a8/Cftr	6	BP
GO:0071229	cellular response to acid chemical	0.02508072	Ffar2/Aqp1/Sfrp1/Apob/Pparg/Avpr1a/Serpinf1/Pck1/Xbp1/Abcb1a/Cps1/Hmgcs2	12	BP
GO:0006816	calcium ion transport	0.025715569	Jph1/Ramp2/Rgn/Gcg/Egf/Snca/Trdn/Spink3/Chrna4/Ramp1/Cckar/F2/Tspan13/Kcne3/Ms4a2/Agt	16	BP
GO:0046849	bone remodeling	0.025823778	Tgfb3/Sfrp1/Lepr/Tf/Syt7/Htr1b	6	BP

GO:0032890	regulation of organic acid transport	0.025982246	Ace2/Snca/Avpr1a/Fxyd1/Agt	5	BP
GO:0002448	mast cell mediated immunity	0.026126309	Chga/Tac4/Fcer1a/Ms4a2	4	BP
GO:2000351	regulation of endothelial cell apoptotic process	0.026126309	Ramp2/Cd248/Xbp1/Rgcc	4	BP
GO:0048011	neurotrophin TRK receptor signaling pathway	0.0267137	Mageh1/Dok5/Agt	3	BP
GO:0061437	renal system vasculature development	0.0267137	Tcf21/Cd34/Aqp1	3	BP
GO:0061440	kidney vasculature development	0.0267137	Tcf21/Cd34/Aqp1	3	BP
GO:0071985	multivesicular body sorting pathway	0.0267137	Rab27a/Rab27b/Sytl4	3	BP
GO:1900274	regulation of phospholipase C activity	0.0267137	Ang/Ms4a2/Agt	3	BP
GO:0006068	ethanol catabolic process	0.026864164	Adh5/Aldh1b1	2	BP
GO:0006527	arginine catabolic process	0.026864164	Oat/Fah	2	BP
GO:0007440	foregut morphogenesis	0.026864164	Shh/Gata4	2	BP
GO:0016264	gap junction assembly	0.026864164	Ace2/Agt	2	BP
GO:0030432	peristalsis	0.026864164	Sstr2/Agt	2	BP
GO:0032372	negative regulation of sterol transport	0.026864164	Shh/Egf	2	BP
GO:0032375	negative regulation of cholesterol transport	0.026864164	Shh/Egf	2	BP
GO:0032725	positive regulation of granulocyte macrophage colony-stimulating factor production	0.026864164	Isl1/Fcer1a	2	BP
GO:0032799	low-density lipoprotein receptor particle metabolic process	0.026864164	Pparg/Adipoq	2	BP
GO:0045346	regulation of MHC class II biosynthetic process	0.026864164	Pf4/Xbp1	2	BP
GO:0060290	transdifferentiation	0.026864164	Cd34/Gata4	2	BP
GO:0060405	regulation of pe-	0.026864164	Shh/Oxtr	2	BP

	nile erection				
GO:0060433	bronchus development	0.026864164	Agr2/Spdef	2	BP
GO:0060453	regulation of gastric acid secretion	0.026864164	Nmu/Oxtr	2	BP
GO:0060525	prostate glandular acinus development	0.026864164	Tp63/Sfrp1	2	BP
GO:0060767	epithelial cell proliferation involved in prostate gland development	0.026864164	Shh/Serpinf1	2	BP
GO:0061469	regulation of type B pancreatic cell proliferation	0.026864164	Fmc1/Sfrp1	2	BP
GO:0070071	proton-transporting two-sector ATPase complex assembly	0.026864164	Fmc1/Aldob	2	BP
GO:0070444	oligodendrocyte progenitor proliferation	0.026864164	Shh/Tf	2	BP
GO:0070445	regulation of oligodendrocyte progenitor proliferation	0.026864164	Shh/Tf	2	BP
GO:1903998	regulation of eating behavior	0.026864164	Nmu/Lepr	2	BP
GO:2000047	regulation of cell-cell adhesion mediated by cadherin	0.026864164	Nexmif/Rgcc	2	BP
GO:0034754	cellular hormone metabolic process	0.026907567	Srd5a1/Crabp2/Shh/Hsd17b2/Bmp6/Rbp2	6	BP
GO:0050900	leukocyte migration	0.026937502	Chga/Ccl24/Pf4/Ffar2/Cd34/Ccr3/Tnfrsf18/Chst4/Ccl11/Apod/Cd99l2/Padi2/Edn2	13	BP
GO:0045807	positive regulation of endocytosis	0.026958755	Rab27a/Sfrp4/Egf/Snca/Pparg/Tf/Sftpa1/Gpc3	8	BP
GO:0009101	glycoprotein biosynthetic process	0.027094938	Fut9/Chst13/Dpm3/B3gnt6/Galnt5/Ramp1/St3gal3/Cytl1/Bace2/St3gal1/B3galt1	11	BP
GO:0019229	regulation of vasoconstriction	0.027285613	Klk1c9/Avpr1a/Oxtr/Agtr1b/Edn2	5	BP
GO:0048806	genitalia development	0.027790864	Srd5a1/Shh/Tp63/Bmp6	4	BP
GO:0003300	cardiac muscle hypertrophy	0.028021083	Pi16/Col14a1/Gata4/Klf15/Adk/Agt	6	BP
GO:0046486	glycerolipid metabolic process	0.028113619	Rgn/Dpm3/Pnpla3/Apob/Ces1d/Pck1/Ang/G6pc/Lpl/Cps1/Prdx6/Thrsp/Aadac	13	BP
GO:0006096	glycolytic process	0.028629867	Dhtkd1/Fbp1/Esrrb/Pklr/Aldob	5	BP
GO:0071456	cellular response to hypoxia	0.028698135	Ptgis/Cd34/Aqp1/Sfrp1/Clca1/Pck1/Cbs/Rgcc	8	BP

GO:0050728	negative regulation of inflammatory response	0.029014251	Ptgis/Pparg/Serpinf1/F2/Isl1/Adipoq/Apod	7	BP
GO:0009952	anterior/posterior pattern specifica-tion	0.029078149	Pcsk5/Pcdh8/Neurod1/Shh/Sfrp1/Gata4/Wnt2b/Meox2/Pcsk6/Gp c3	10	BP
GO:0035640	exploration behav-ior	0.029112305	Slc4a10/Chrna4/Lsamp	3	BP
GO:0035883	enteroendocrine cell differentiation	0.029112305	Neurod1/Bmp6/Cftr	3	BP
GO:0099622	cardiac muscle cell membrane repolar-ization	0.029112305	Kcnh2/Kcnj5/Kcne3	3	BP
GO:0032409	regulation of transporter activity	0.029126864	Rgn/Kcns1/Fxyd3/Snca/Trdn/Abcb1a/Chrna4/Fxyd1/Kcne3/Cftr/ Lrrc26	11	BP
GO:0045446	endothelial cell differentiation	0.029164602	Tgfb3/Bmp6/Tnmd/Pde2a/Col23a1/Agt	6	BP
GO:0030308	negative regulation of cell growth	0.02924814	Cth/Pi16/Slit1/Fbp1/Sfrp1/Cgref1/Pparg/Dact3/Agt	9	BP
GO:0032103	positive regulation of response to external stimulus	0.029413147	Pgc/Ccl24/Shh/Ffar2/Snca/Cpb2/Bmp6/Klk1/Fabp4/Pde2a/Fcer1 a/Edn2	12	BP
GO:0010517	regulation of phos-pholipase activity	0.029517277	Snca/Ang/Ms4a2/Agt	4	BP
GO:0010712	regulation of col-lagen metabolic process	0.029517277	Tgfb3/Pparg/F2/Rgcc	4	BP
GO:0006757	ATP generation from ADP	0.030015343	Dhtkd1/Fbp1/Esrrb/Pklr/Aldob	5	BP
GO:0043401	steroid hormone mediated signaling pathway	0.030513552	Nr0b2/Tcf21/Tp63/Esrrb/Sfrp1/Pparg/Isl1/Padi2	8	BP
GO:0006898	receptor-mediated endocytosis	0.031265925	Ramp2/Sfrp4/Amn/Egf/Snca/Ackr2/Tf/Asgr1/Ramp1/Scara5/Htr 1b	11	BP
GO:0002024	diet induced ther-mogenesis	0.031299719	Pparg/Bmp8a	2	BP
GO:0007213	G protein-coupled acetylcholine re-ceptor signaling pathway	0.031299719	Hrh4/Chrm1	2	BP
GO:0007320	insemination	0.031299719	Avpr1a/Oxtr	2	BP
GO:0007614	short-term memory	0.031299719	Calb1/Serpinf1	2	BP
GO:0010889	regulation of se-questering of tri-glyceride	0.031299719	Pparg/Lpl	2	BP
GO:0019370	leukotriene biosyn-thetic process	0.031299719	Ltc4s/Fcer1a	2	BP
GO:0021520	spinal cord motor neuron cell fate specification	0.031299719	Isl1/Isl2	2	BP

GO:0021924	cell proliferation in external granule layer	0.031299719	Shh/Egf	2	BP
GO:0021930	cerebellar granule cell precursor proliferation	0.031299719	Shh/Egf	2	BP
GO:0021936	regulation of cerebellar granule cell precursor proliferation	0.031299719	Shh/Egf	2	BP
GO:0042268	regulation of cytolysis	0.031299719	Pf4/Cfh	2	BP
GO:0045342	MHC class II biosynthetic process	0.031299719	Pf4/Xbp1	2	BP
GO:0051152	positive regulation of smooth muscle cell differentiation	0.031299719	Cth/Shh	2	BP
GO:0060391	positive regulation of SMAD protein signal transduction	0.031299719	Tgfb3/Bmp6	2	BP
GO:0060452	positive regulation of cardiac muscle contraction	0.031299719	Chga/Ace2	2	BP
GO:0060601	lateral sprouting from an epithelium	0.031299719	Shh/Tp63	2	BP
GO:0061140	lung secretory cell differentiation	0.031299719	Agr2/Spdef	2	BP
GO:1903624	regulation of DNA catabolic process	0.031299719	Rgn/Igfbp3	2	BP
GO:1904386	response to L-phenylalanine derivative	0.031299719	Abcb1a/F2	2	BP
GO:2000052	positive regulation of non-canonical Wnt signaling pathway	0.031299719	Sfrp1/Gpc3	2	BP
GO:2000402	negative regulation of lymphocyte migration	0.031299719	Apod/Padi2	2	BP
GO:2001212	regulation of vasculogenesis	0.031299719	Ramp2/Cd34	2	BP
GO:0032964	collagen biosynthetic process	0.031305856	Tgfb3/Pparg/F2/Rgcc	4	BP
GO:0060395	SMAD protein signal transduction	0.031442352	Tgfb3/Tf/Gata4/Bmp8a/Bmp6	5	BP
GO:2000243	positive regulation of reproductive process	0.031442352	Zfpm2/Shh/Abcb1a/Oxtr/Cftr	5	BP
GO:0014897	striated muscle hypertrophy	0.031542691	Pi16/Col14a1/Gata4/Klf15/Adk/Agt	6	BP

GO:0032414	positive regulation of ion transmembrane transporter activity	0.031542691	Rgn/Trdn/Abcb1a/Kcne3/Cftr/Lrrc26	6	BP
GO:0043648	dicarboxylic acid metabolic process	0.031542691	Aadat/Adhfe1/Oat/Pck1/Phyh/Me3	6	BP
GO:0007274	neuromuscular synaptic transmission	0.031621012	Chrm1/Chrna4/Etv5	3	BP
GO:0019835	cytolysis	0.031621012	Pf4/F2/Cfh	3	BP
GO:0032967	positive regulation of collagen biosynthetic process	0.031621012	Tgfb3/F2/Rgcc	3	BP
GO:0071772	response to BMP	0.032406335	Sfrp4/Grem2/Sfrp1/Gata4/Bmp8a/Bmp6/Tnmd/Gpc3	8	BP
GO:0071773	cellular response to BMP stimulus	0.032406335	Sfrp4/Grem2/Sfrp1/Gata4/Bmp8a/Bmp6/Tnmd/Gpc3	8	BP
GO:0014812	muscle cell migration	0.032777757	Pcsk5/Six4/Xbp1/Igfbp3/Adipoq/Agt	6	BP
GO:0018958	phenol-containing compound metabolic process	0.032777757	Srd5a1/Gpr37/Snca/Tacr3/Fah/Cpq	6	BP
GO:0043266	regulation of potassium ion transport	0.032777757	Kcnh2/Kcns1/Fxyd1/Cckar/Kcne3/Lrrc26	6	BP
GO:0001570	vasculogenesis	0.032911182	Ramp2/Zfpm2/Fzd4/Shh/Cd34	5	BP
GO:0042246	tissue regeneration	0.032911182	Gata4/Cpq/Gatm/Apod/Igfbp1	5	BP
GO:0072577	endothelial cell apoptotic process	0.033156859	Ramp2/Cd248/Xbp1/Rgcc	4	BP
GO:0007254	JNK cascade	0.033534624	Edar/Sfrp4/Fzd4/Sfrp1/Tf/Fzd10/Cbs/Fcer1a/Maged1	9	BP
GO:0006939	smooth muscle contraction	0.034043815	Nmu/Tacr3/Oxtr/Edn2/Sstr2/Agt	6	BP
GO:0014896	muscle hypertrophy	0.034043815	Pi16/Col14a1/Gata4/Klf15/Adk/Agt	6	BP
GO:0060740	prostate gland epithelium morphogenesis	0.034239228	Shh/Tp63/Sfrp1	3	BP
GO:0071827	plasma lipoprotein particle organization	0.034239228	Apob/Ces1d/Lpl	3	BP
GO:0042866	pyruvate biosynthetic process	0.034422099	Dhtkd1/Fbp1/Esrrb/Pklr/Aldob	5	BP
GO:0046209	nitric oxide metabolic process	0.034422099	Rgn/Ptgis/Cd34/Cps1/Agt	5	BP
GO:1903035	negative regulation of response to wounding	0.034422099	Cd34/Thbd/Cpb2/F2/Klk8	5	BP
GO:0090288	negative regulation of cellular response to growth	0.035471198	Dcn/Grem2/Tgfb3/Sfrp1/Xbp1/Mmrn2/Agt	7	BP

	factor stimulus				
GO:1904427	positive regulation of calcium ion transmembrane transport	0.035975349	Rgn/Snca/Trdn/F2/Kcne3	5	BP
GO:0001867	complement activation, lectin pathway	0.036001064	Masp1/Fcna	2	BP
GO:0006067	ethanol metabolic process	0.036001064	Adh5/Aldh1b1	2	BP
GO:0010359	regulation of anion channel activity	0.036001064	Abcb1a/Cftr	2	BP
GO:0021534	cell proliferation in hindbrain	0.036001064	Shh/Egf	2	BP
GO:0031987	locomotion involved in locomotory behavior	0.036001064	Gpr37/Fzd4	2	BP
GO:0032096	negative regulation of response to food	0.036001064	Gcg/Pyy	2	BP
GO:0032604	granulocyte macrophage colony-stimulating factor production	0.036001064	Isl1/Fcer1a	2	BP
GO:0032645	regulation of granulocyte macrophage colony-stimulating factor production	0.036001064	Isl1/Fcer1a	2	BP
GO:0033147	negative regulation of intracellular estrogen receptor signaling pathway	0.036001064	Tp63/Isl1	2	BP
GO:0043116	negative regulation of vascular permeability	0.036001064	Ramp2/Pde2a	2	BP
GO:0048143	astrocyte activation	0.036001064	Bace2/Agt	2	BP
GO:0051791	medium-chain fatty acid metabolic process	0.036001064	Ces1d/Cyp4a1	2	BP
GO:0060736	prostate gland growth	0.036001064	Shh/Prlr	2	BP
GO:0072512	trivalent inorganic cation transport	0.036001064	Tf/Rep15	2	BP
GO:0090494	dopamine uptake	0.036001064	Snca/Nat8l	2	BP
GO:1900119	positive regulation of execution phase of apoptosis	0.036001064	Ptgis/Igfbp3	2	BP
GO:2000095	regulation of Wnt signaling pathway, planar cell polarity pathway	0.036001064	Sfrp1/Gpc3	2	BP

GO:2000252	negative regulation of feeding behavior	0.036001064	Nmu/Lepr	2	BP
GO:0006970	response to osmotic stress	0.036669773	Sord/Aqp1/Tacr3/Abcb1a/Agtr1b/Agt	6	BP
GO:0008203	cholesterol metabolic process	0.036669773	Apob/Ces1d/Lepr/Npc1l1/Cftr/Hmgcs2	6	BP
GO:0002675	positive regulation of acute inflammatory response	0.036966221	Ffar2/Klk1/Fcer1a	3	BP
GO:0003401	axis elongation	0.036966221	Shh/Six4/Sfrp1	3	BP
GO:0032354	response to follicle-stimulating hormone	0.036966221	Srd5a1/Tf/Gata4	3	BP
GO:0035116	embryonic hindlimb morphogenesis	0.036966221	Shh/Tp63/Gpc3	3	BP
GO:0045745	positive regulation of G protein-coupled receptor signaling pathway	0.036966221	Chga/Mrap2/F2	3	BP
GO:0086091	regulation of heart rate by cardiac conduction	0.036966221	Kcnh2/Kcnj5/Kcne3	3	BP
GO:0140115	export across plasma membrane	0.036966221	Kcnh2/Fxyd1/Kcne3	3	BP
GO:0009247	glycolipid biosynthetic process	0.037046904	Ugt8/Dpm3/Gal3st1/B3galt1	4	BP
GO:0044060	regulation of endocrine process	0.037046904	Avpr1a/Bmp6/Pex5l/Agt	4	BP
GO:0061951	establishment of protein localization to plasma membrane	0.037046904	Amn/Trarg1/Anxa13/Krt18	4	BP
GO:0006942	regulation of striated muscle contraction	0.037571153	Chga/Ace2/Gata4/Fxyd1/Kcne3	5	BP
GO:0070252	actin-mediated cell contraction	0.037571153	Kcnh2/Kcnj5/Gata4/Fxyd1/Kcne3	5	BP
GO:0010466	negative regulation of peptidase activity	0.038351577	Serpina1/Serpina10/Aqp1/Snca/Spock1/Serpinf1/Spink3/Pzp/Cst6/Agt/Gpc3	11	BP
GO:0097327	response to antineoplastic agent	0.038561272	Srd5a1/Aqp1/Lepr/Serpinf1/Pck1/Abcb1a/Cps1/Agtr1b	8	BP
GO:0009408	response to heat	0.03902997	Cd34/Cpb2/Scara5/Cckar/Hspb7/Pklr/Cftr	7	BP
GO:0010717	regulation of epithelial to mesenchymal transition	0.039209711	Tgfb3/Sfrp1/Isl1/Rgcc/Dact3	5	BP
GO:0035773	insulin secretion	0.039209711	Gcg/Pim3/Lepr/Ptprn2/Cftr	5	BP

	involved in cellular response to glucose stimulus				
GO:0044272	sulfur compound biosynthetic process	0.039209711	Cth/Chst13/Snca/Cbs/Cdo1	5	BP
GO:0043271	negative regulation of ion transport	0.039658108	Kcnh2/Sfrp4/Snca/Trdn/Spink3/Kcne3/Slc18a1/Htr1b	8	BP
GO:1990823	response to leukemia inhibitory factor	0.039658108	Cth/Efhc2/Fzd4/Xbp1/Sstr1/Bcat2/PCOLCE2/Padi2	8	BP
GO:1990830	cellular response to leukemia inhibitory factor	0.039658108	Cth/Efhc2/Fzd4/Xbp1/Sstr1/Bcat2/PCOLCE2/Padi2	8	BP
GO:0010714	positive regulation of collagen metabolic process	0.039801128	Tgfb3/F2/Rgcc	3	BP
GO:0033238	regulation of cellular amine metabolic process	0.039801128	Gpr37/Snca/Tacr3	3	BP
GO:0050802	circadian sleep/wake cycle, sleep	0.039801128	Srd5a1/Nmu/Ptgds	3	BP
GO:0050892	intestinal absorption	0.039801128	Fabp2/Abcb1a/Npc1l1	3	BP
GO:0060512	prostate gland morphogenesis	0.039801128	Shh/Tp63/Sfrp1	3	BP
GO:0071870	cellular response to catecholamine stimulus	0.039801128	Srd5a1/Pklr/Adipoq	3	BP
GO:1904707	positive regulation of vascular associated smooth muscle cell proliferation	0.039801128	Xbp1/Htr1b/Agt	3	BP
GO:0071805	potassium ion transmembrane transport	0.040237982	Kcnh2/Kcnj5/Aqp1/Kcns1/Kcnv2/Fxyd1/Kcne3/Slc12a8/Lrrc26	9	BP
GO:0070372	regulation of ERK1 and ERK2 cascade	0.040440262	Rps6ka6/Alkal2/Ace2/Ccl24/Gcg/Egf/Fbln1/Xbp1/Gata4/Adipoq/Ccl11/Ros1/Trem2	13	BP
GO:0032368	regulation of lipid transport	0.04084627	Shh/Egf/Bmp6/Igfbp3/Adipoq/Agt	6	BP
GO:0032411	positive regulation of transporter activity	0.04084627	Rgn/Trdn/Abcb1a/Kcne3/Cftr/Lrrc26	6	BP
GO:2001057	reactive nitrogen species metabolic process	0.040891201	Rgn/Ptgis/Cd34/Cps1/Agt	5	BP
GO:0016486	peptide hormone processing	0.040954633	Pcsk5/Pcsk6	2	BP

GO:0034310	primary alcohol catabolic process	0.040954633	Adh5/Aldh1b1	2	BP
GO:0035112	genitalia morphogenesis	0.040954633	Shh/Tp63	2	BP
GO:0035641	locomotory exploration behavior	0.040954633	Slc4a10/Lsamp	2	BP
GO:0035815	positive regulation of renal sodium excretion	0.040954633	Avpr1a/Agt	2	BP
GO:0042953	lipoprotein transport	0.040954633	Apob/Pparg	2	BP
GO:0044241	lipid digestion	0.040954633	Aqp1/Npc1l1	2	BP
GO:0044872	lipoprotein localization	0.040954633	Apob/Pparg	2	BP
GO:0051386	regulation of neurotrophin TRK receptor signaling pathway	0.040954633	Dok5/Agt	2	BP
GO:0060442	branching involved in prostate gland morphogenesis	0.040954633	Shh/Sfrp1	2	BP
GO:0090493	catecholamine uptake	0.040954633	Snca/Nat8l	2	BP
GO:0098856	intestinal lipid absorption	0.040954633	Fabp2/Npc1l1	2	BP
GO:1900016	negative regulation of cytokine production involved in inflammatory response	0.040954633	F2/Apod	2	BP
GO:0003229	ventricular cardiac muscle tissue development	0.041188442	Zfpm2/Col14a1/Gata4/Isl1	4	BP
GO:0035272	exocrine system development	0.041188442	Edar/Shh/Tgfb3/Xbp1	4	BP
GO:0046850	regulation of bone remodeling	0.041188442	Sfrp1/Lepr/Tf/Syt7	4	BP
GO:0097006	regulation of plasma lipoprotein particle levels	0.041188442	Apob/Ces1d/Lpl/Adipoq	4	BP
GO:0035637	multicellular organismal signaling	0.04152788	Kcnh2/Ace2/Kcnj5/Trdn/Avpr1a/Kcne3/Agt	7	BP
GO:0032412	regulation of ion transmembrane transporter activity	0.041890677	Rgn/Kcns1/Fxyd3/Trdn/Abcb1a/Chrna4/Fxyd1/Kcne3/Cftr/Lrrc26	10	BP
GO:0051346	negative regulation of hydrolase activity	0.042023664	Rgn/Serpina1/Serpina10/Aqp1/Snca/Spock1/Lepr/Serpinf1/Spink3/Fzd10/Ptprn2/Ppp1r14c/Pzp/Cst6/Agt/Gpc3	16	BP
GO:0009132	nucleoside diphosphate metabolic	0.04230249	Dhtkd1/Fbp1/Esrrb/Pck1/Pklr/Aldob	6	BP

	process				
GO:0007595	lactation	0.042615778	Xbp1/Abcb1a/Prlr/Bcat2/Cdo1	5	BP
GO:0035115	embryonic forelimb morphogenesis	0.04274296	Crabp2/Shh/Tp63	3	BP
GO:0043001	Golgi to plasma membrane protein transport	0.04274296	Amn/Anxa13/Krt18	3	BP
GO:0048147	negative regulation of fibroblast proliferation	0.04274296	Sfrp1/Nupr1/Pparg	3	BP
GO:0071868	cellular response to monoamine stimulus	0.04274296	Srd5a1/Pklr/Adipoq	3	BP
GO:1903427	negative regulation of reactive oxygen species biosynthetic process	0.04274296	Rgn/Ptgis/Cd34	3	BP
GO:0030178	negative regulation of Wnt signaling pathway	0.042814894	Sfrp4/Shh/Sfrp1/Dkk2/Isl1/Dact3/Gpc3	7	BP
GO:0007626	locomotory behavior	0.042862841	Gpr37/Calb1/Fzd4/Slc4a10/Snca/Lepr/Chrna4/Lsamp/Fign/Etv5	10	BP
GO:0055081	anion homeostasis	0.043353629	Sfrp4/Xbp1/Cps1/Slc12a8	4	BP
GO:1901016	regulation of potassium ion transmembrane transporter activity	0.043353629	Kcns1/Fxyd1/Kcne3/Lrrc26	4	BP
GO:1902652	secondary alcohol metabolic process	0.043790998	Apob/Ces1d/Lepr/Npc1l1/Cftr/Hmgcs2	6	BP
GO:0014910	regulation of smooth muscle cell migration	0.044383578	Pcsk5/Xbp1/Igfbp3/Adipoq/Agt	5	BP
GO:0046031	ADP metabolic process	0.044383578	Dhtkd1/Fbp1/Esrrb/Pklr/Aldob	5	BP
GO:0050764	regulation of phagocytosis	0.044383578	Rab27a/Pparg/Syt7/Adipoq/Sftpa1	5	BP
GO:1901379	regulation of potassium ion transmembrane transport	0.044383578	Kcnh2/Kcns1/Fxyd1/Kcne3/Lrrc26	5	BP
GO:0071260	cellular response to mechanical stimulus	0.045311933	Aqp1/Bmp6/F2/Pde2a/Sftpa1/Agt	6	BP
GO:0060537	muscle tissue development	0.045577384	Dcn/Zfpm2/Pi16/Shh/Six4/Col14a1/Tcf21/Tp63/Nupr1/Gata4/Eya2/Isl1/Myom2/Meox2/Hlf/Agt	16	BP
GO:0031638	zymogen activation	0.045581723	Klk1b3/Klk1c9/Cpb2/Klk10	4	BP
GO:0045600	positive regulation of fat cell differen-	0.045581723	Sfrp1/Aamdc/Pparg/Xbp1	4	BP

	tiation				
GO:0006536	glutamate metabolic process	0.045790618	Aadat/Adhfe1/Oat	3	BP
GO:0030225	macrophage differentiation	0.045790618	Cebpe/Pf4/Adipoq	3	BP
GO:0030728	ovulation	0.045790618	Lepr/Edn2/Agt	3	BP
GO:0040019	positive regulation of embryonic development	0.045790618	Shh/Six4/Wnt2b	3	BP
GO:0048665	neuron fate specification	0.045790618	Shh/Isl1/Isl2	3	BP
GO:0060259	regulation of feeding behavior	0.045790618	Nmu/Tacr3/Lepr	3	BP
GO:0071825	protein-lipid complex subunit organization	0.045790618	Apob/Ces1d/Lpl	3	BP
GO:0001886	endothelial cell morphogenesis	0.046147323	Tnmd/Col23a1	2	BP
GO:0009312	oligosaccharide biosynthetic process	0.046147323	Fbp1/B3galt1	2	BP
GO:0030449	regulation of complement activation	0.046147323	Masp1/Cfh	2	BP
GO:0030730	sequestering of triglyceride	0.046147323	Pparg/Lpl	2	BP
GO:0032105	negative regulation of response to extracellular stimulus	0.046147323	Gcg/Pyy	2	BP
GO:0032108	negative regulation of response to nutrient levels	0.046147323	Gcg/Pyy	2	BP
GO:0042416	dopamine biosynthetic process	0.046147323	Gpr37/Snca	2	BP
GO:0045721	negative regulation of gluconeogenesis	0.046147323	Lepr/Adipoq	2	BP
GO:0050872	white fat cell differentiation	0.046147323	Pparg/Fabp4	2	BP
GO:0051873	killing by host of symbiont cells	0.046147323	Pf4/F2	2	BP
GO:0051923	sulfation	0.046147323	Sult1c2a/Chst4	2	BP
GO:0090051	negative regulation of cell migration involved in sprouting angiogenesis	0.046147323	Mmrn2/Meox2	2	BP
GO:2000194	regulation of female gonad development	0.046147323	Zfpm2/Nupr1	2	BP
GO:0060538	skeletal muscle	0.046662833	Dcn/Shh/Six4/Tcf21/Fgfrl1/Nupr1/Myom2/Meox2/Hlf	9	BP

	organ development				
GO:0034341	response to interferon-gamma	0.046829749	Cdc42ep2/Ccl24/Snca/Pparg/Slc30a8/Cfh/Ccl11	7	BP
GO:0050715	positive regulation of cytokine secretion	0.046829749	Fcna/Ffar2/Cd34/Xbp1/Lpl/Rgcc/Agt	7	BP
GO:0048512	circadian behavior	0.047872638	Srd5a1/Nmu/Lepr/Ptgds	4	BP
GO:0060393	regulation of pathway-restricted SMAD protein phosphorylation	0.047872638	Tgfb3/Xbp1/Bmp8a/Bmp6	4	BP
GO:1901616	organic hydroxy compound catabolic process	0.047872638	Adh5/Aldh1b1/Sord/Fah	4	BP
GO:0090263	positive regulation of canonical Wnt signaling pathway	0.048049267	Sfrp4/Egf/Sfrp1/Dkk2/Gpc3	5	BP
GO:0045017	glycerolipid biosynthetic process	0.048219702	Rgn/Dpm3/Pnpla3/Pck1/Ang/Lpl/Thrsp	7	BP
GO:0045860	positive regulation of protein kinase activity	0.048570378	Alkal2/Gprc5c/Fzd4/Gcg/Egf/Tgfb3/Snca/Tf/Fzd10/Ang/Prlr/Adipoq/Rgcc/Fcer1a/Maged1/Agt	16	BP
GO:0021522	spinal cord motor neuron differentiation	0.048942892	Shh/Isl1/Isl2	3	BP
GO:0022410	circadian sleep/wake cycle process	0.048942892	Srd5a1/Nmu/Ptgds	3	BP
GO:0034260	negative regulation of GTPase activity	0.048942892	Rgn/Fzd10/Ptprn2	3	BP
GO:0042311	vasodilation	0.048942892	Cps1/Cftr/Agt	3	BP
GO:0044275	cellular carbohydrate catabolic process	0.048942892	Sord/Avpr1a/G6pc	3	BP
GO:0045880	positive regulation of smoothened signaling pathway	0.048942892	Shh/Sfrp1/Gpc3	3	BP
GO:0061900	glial cell activation	0.048942892	Snca/Bace2/Agt	3	BP
GO:0017157	regulation of exocytosis	0.049019656	Rab27a/Rab27b/Snca/Chrna4/Sytl4/Syt7/Htr1b/Fcer1a/Cftr/Ms4a2	10	BP
GO:0042133	neurotransmitter metabolic process	0.04963567	Srd5a1/Rgn/Chdh/Ptgis/Cd34/Cps1/Agt	7	BP
GO:0031670	cellular response to nutrient	0.049947315	Sfrp1/Pparg/Xbp1/Abcb1a/Ltc4s	5	BP
GO:0030141	secretory granule	2.28711E-06	Tff3/Chga/Rab27a/Pcsk5/Klk11/Rab27b/Zg16/Klk1b3/Klk1c9/Pf4/Tgfb3/Snca/Clca1/Chgb/Slc30a8/Ptprn2/Sytl4/Klk1/Igfbp3/Klk9/Syt7/Klk8/Bace2/Klk10	24	CC
GO:0031012	extracellular matrix	1.15614E-05	Dcn/Fcgbp/Mmp10/Mfap5/Zg16/Shh/Col14a1/Fbln1/Tgfb3/Sfrp1/Serpinf1/Tf/Mmrn2/Efemp1/Ang/Lum/F2/Ccdc80/Emid1/Col23a1/Pcsk6/Gpc3	22	CC

GO:0062023	collagen-containing extra-cellular matrix	4.10974E-05	Col14a1/Fbln1/Tgfb3/Sfrp1/Serpinf1/Tf/Mmrn2/Efemp1/Ang/Lum/F2/Ccdc80/Pcsk6/Gpc3	14	CC
GO:0005833	hemoglobin com-plex	8.90466E-05	Hbb/Hba-a1/Hbb-b1/Hba-a2	4	CC
GO:0045177	apical part of cell	0.000190736	Rab27a/Edar/Rab27b/Amn/Adgrg2/Slc9a3r2/Cd34/Aqp1/Prom1/Fabp2/Tf/Abcb1a/Sytl4/Anxa13/Oxtr/Fxyd1/Klk1/Npc1l1/Slc6a19/Mgst1/Aqp8/Cftr	22	CC
GO:0016324	apical plasma membrane	0.000368055	Rab27a/Rab27b/Amn/Adgrg2/Slc9a3r2/Cd34/Aqp1/Prom1/Tf/Abcb1a/Sytl4/Anxa13/Oxtr/Fxyd1/Npc1l1/Slc6a19/Aqp8/Cftr	18	CC
GO:0005903	brush border	0.000619956	Scin/Ace2/Amn/Aqp1/Prom1/Abcb1a/Npc1l1/Slc6a19/Itln1/Myl6	10	CC
GO:0031526	brush border membrane	0.00086574	Ace2/Amn/Aqp1/Abcb1a/Npc1l1/Slc6a19/Itln1	7	CC
GO:0031838	haptoglobin-hemoglobin com-plex	0.00104193	Hbb/Hba-a1/Hba-a2	3	CC
GO:0030667	secretory granule membrane	0.002140128	Rab27a/Rab27b/Zg16/Snca/Clca1/Ptprn2/Sytl4	7	CC
GO:0098862	cluster of actin-based cell projec-tions	0.002364833	Scin/Ace2/Amn/Slc9a3r2/Aqp1/Prom1/Abcb1a/Npc1l1/Slc6a19/Itln1/Myl6	11	CC
GO:0042589	zymogen granule membrane	0.002965704	Rab27b/Zg16/Clca1	3	CC
GO:0005581	collagen trimer	0.003302569	Dcn/Fcna/Lum/Adipoq/Sftpa1/Col23a1	6	CC
GO:0043235	receptor complex	0.005726953	Gpr37/Ramp2/Gprc5c/Gpr20/Egf/Gpr119/Lepr/Tf/Chrna4/Il5ra/Ptprn2/Ramp1/Prlr/Itgbl1/Pex5l/Ros1/Itln1	17	CC
GO:0005604	basement mem-brane	0.006991951	Fbln1/Serpinf1/Tf/Mmrn2/Efemp1/Ang/Ccdc80	7	CC
GO:0031091	platelet alpha granule	0.007294273	Pf4/Snca/Igfbp3	3	CC
GO:0043679	axon terminus	0.007963086	Calb1/Aqp1/Nmu/Snca/Chrm1/Ptprn2/Cckar/Flrt3/Syt7/Slc18a1/Htr1b	11	CC
GO:0008076	voltage-gated potassium channel complex	0.008728762	Kcnh2/Kcnj5/Kcns1/Kcnv2/Kcne3/Lrrc26	6	CC
GO:0012506	vesicle membrane	0.013545311	Chga/Rab27a/Rab27b/Zg16/Trarg1/Fgfrl1/Snca/Apob/Clca1/Avpr1a/Slc30a8/Ptprn2/Sytl4/Syt7/Slc18a1/Cftr	16	CC
GO:0005640	nuclear outer membrane	0.014082935	Snca/Ltc4s/Gsta1	3	CC
GO:0042588	zymogen granule	0.014082935	Rab27b/Zg16/Clca1	3	CC
GO:0009897	external side of plasma membrane	0.01421491	Fcna/Cd248/Kcnj5/Cd34/Lepr/Ackr2/Tf/Ccr3/Chrna4/Il5ra/Prlr/F2/Tnfrsf18/Fcer1a/Ms4a2	15	CC
GO:0044306	neuron projection terminus	0.015426649	Calb1/Aqp1/Nmu/Snca/Chrm1/Ptprn2/Cckar/Flrt3/Syt7/Slc18a1/Htr1b	11	CC
GO:0034705	potassium channel complex	0.015510832	Kcnh2/Kcnj5/Kcns1/Kcnv2/Kcne3/Lrrc26	6	CC
GO:0030659	cytoplasmic vesi-	0.016210364	Chga/Rab27a/Rab27b/Zg16/Trarg1/Fgfrl1/Snca/Clca1/Avpr1a/Sl	15	CC

	cle membrane		c30a8/Ptprn2/Sytl4/Syt7/Slc18a1/Cftr		
GO:0030658	transport vesicle membrane	0.016654468	Chga/Rab27b/Fgfrl1/Snca/Slc30a8/Ptprn2/Sytl4/Syt7/Slc18a1	9	CC
GO:0030430	host cell cytoplasm	0.018329527	Pf4/Aqp1	2	CC
GO:0033655	host cell cytoplasm part	0.018329527	Pf4/Aqp1	2	CC
GO:0051286	cell tip	0.018329527	Tf/Pex5l	2	CC
GO:0043195	terminal bouton	0.018729535	Calb1/Nmu/Snca/Ptprn2/Cckar/Syt7/Slc18a1	7	CC
GO:0043230	extracellular organelle	0.02043877	Acy1/Sord/Egf/Pf4/Aqp1/Prom1	6	CC
GO:0042627	chylomicron	0.02208942	Apob/Lpl	2	CC
GO:0033646	host intracellular part	0.030459009	Pf4/Aqp1	2	CC
GO:0043656	host intracellular region	0.030459009	Pf4/Aqp1	2	CC
GO:0070062	extracellular exosome	0.032620682	Acy1/Sord/Egf/Aqp1/Prom1	5	CC
GO:0005791	rough endoplasmic reticulum	0.034520688	Tp63/Snca/Aldob/Nat8l/Ptgds/Sftpa1	6	CC
GO:0032809	neuronal cell body membrane	0.035621753	Aqp1/Tacr3/Kcne3	3	CC
GO:0044298	cell body membrane	0.038362042	Aqp1/Tacr3/Kcne3	3	CC
GO:0042734	presynaptic membrane	0.039168024	Pcdh8/Slc1a7/Chrm1/Chrna4/Flrt3/Syt7/Pde2a/Htr1b/Snph	9	CC
GO:0033643	host cell part	0.039870832	Pf4/Aqp1	2	CC
GO:0031983	vesicle lumen	0.044935204	Zg16/Apob	2	CC
GO:0098981	cholinergic synapse	0.044935204	Chrm1/Chrna4	2	CC
GO:0005902	microvillus	0.047434165	Prom1/Clca1/Fabp2/Oxtr/Cftr	5	CC
GO:1903561	extracellular vesicle	0.047434165	Acy1/Sord/Egf/Aqp1/Prom1	5	CC
GO:1901681	sulfur compound binding	1.68382E-10	Dhtkd1/Grem2/Slit1/Serpina10/Pf4/Cd34/Fgfrl1/Sfrp1/Gstm5/Pnpla3/Apob/Ang/Lpl/F2/Gstm1/Cbs/Cfh/Ccdc80/Mgst1/Ltc4s/PCOLCE2/Col23a1/Gsta1/Pcsk6/Reg4	25	MF
GO:0008528	G protein-coupled peptide receptor activity	1.06383E-07	Gpr37/Sstr5/Ramp2/Mc2r/Gpr182/Tacr3/Avpr1a/Ackr2/Ccr3/Ramp1/Prokr2/Sstr1/Oxtr/Cckar/Agtr1b/Sstr2	16	MF
GO:0001653	peptide receptor activity	2.47479E-07	Gpr37/Sstr5/Ramp2/Mc2r/Gpr182/Tacr3/Avpr1a/Ackr2/Ccr3/Ramp1/Prokr2/Sstr1/Oxtr/Cckar/Agtr1b/Sstr2	16	MF
GO:0042277	peptide binding	2.8684E-07	Gpr37/Sstr5/Ramp2/Pcsk5/Fzd4/Trhde/Gstm5/Pparg/Avpr1a/Lepr/Ang/Ramp1/Prlr/Sstr1/Oxtr/Cckar/Pex5l/Gstm1/Mgst1/Bace2/Ltc4s/Gsta1/Sstr2	23	MF
GO:0005539	glycosaminoglycan binding	5.32827E-07	Dcn/Grem2/Shh/Serpina10/Pf4/Fgfrl1/Sfrp1/Apob/Ang/Lpl/F2/Cfh/Ccdc80/PCOLCE2/Col23a1/Pcsk6/Trem2/Reg4	18	MF
GO:0033218	amide binding	7.88773E-07	Srd5a1/Gpr37/Sstr5/Ramp2/Pcsk5/Fzd4/Trhde/Gstm5/Pnpla3/Pparg/Avpr1a/Lepr/Ang/Ramp1/Prlr/Sstr1/Oxtr/Cckar/Pex5l/Gstm1	25	MF

			/Mgst1/Bace2/Ltc4s/Gsta1/Sstr2		
GO:0008201	heparin binding	1.23948E-06	Grem2/Serpina10/Pf4/Fgfrl1/Sfrp1/Apob/Ang/Lpl/F2/Cfh/Ccdc80/PCOLCE2/Col23a1/Pcsk6/Reg4	15	MF
GO:0004175	endopeptidase activity	3.18488E-06	Ad-am28/Pgc/Capn8/Pcsk5/Mmp10/Ace2/Klk11/Mcpt8l2/Ctsf/Masp1/Klk1b3/Klk1c9/Pga5/Sfrp1/Capn9/Klk1/F2/Cps1/Klk9/Klk8/Bace2/Klk10/Tpsg1/Mcpt10/Cfd/Casp14/Pcsk6	27	MF
GO:0043177	organic acid binding	8.11207E-06	Adh5/Hbb/Hba-a1/Crabp2/Thnsl2/Snca/Pparg/Pck1/Fabp2/Cyp4f1/Ugt1a1/Phyh/Cps1/Fabp4/Adipoq/Hba-a2/Ptgds	17	MF
GO:0005506	iron ion binding	1.10799E-05	Cyp4b1/Ptgis/Cyp2d5/Cyp8b1/Hba-a1/Cyp2d2/Snca/Cyp2c24/Tf/Cyp4a1/Cyp4f1/Phyh/Aox3/Cdo1/Hba-a2/Alox12e	16	MF
GO:0033293	monocarboxylic acid binding	1.84282E-05	Adh5/Crabp2/Snca/Pparg/Fabp2/Cyp4f1/Ugt1a1/Fabp4/Ptgds	9	MF
GO:0004252	serine-type endopeptidase activity	3.12632E-05	Pcsk5/Klk11/Mcpt8l2/Masp1/Klk1b3/Klk1c9/Klk1/F2/Klk9/Klk8/Klk10/Tpsg1/Mcpt10/Cfd/Pcsk6	15	MF
GO:0036041	long-chain fatty acid binding	3.29219E-05	Snca/Pparg/Fabp2/Cyp4f1/Fabp4	5	MF
GO:0017171	serine hydrolase activity	3.33511E-05	Pcsk5/Klk11/Mcpt8l2/Masp1/Klk1b3/Klk1c9/Klk1/F2/Klk9/Klk8/Klk10/Tpsg1/Mcpt10/Aadac/Cfd/Pcsk6	16	MF
GO:0005504	fatty acid binding	4.30136E-05	Adh5/Snca/Pparg/Fabp2/Cyp4f1/Fabp4/Ptgds	7	MF
GO:0043295	glutathione binding	4.38816E-05	Gstm5/Gstm1/Mgst1/Ltc4s/Gsta1	5	MF
GO:1900750	oligopeptide binding	5.74643E-05	Gstm5/Gstm1/Mgst1/Ltc4s/Gsta1	5	MF
GO:0020037	heme binding	6.59574E-05	Cyp4b1/Hbb/Ptgis/Cyp2d5/Cyp8b1/Hba-a1/Cyp2d2/Hbb-b1/Cyp2c24/Cyp4a1/Cyp4f1/Cbs/Hba-a2	13	MF
GO:0008236	serine-type peptidase activity	9.39632E-05	Pcsk5/Klk11/Mcpt8l2/Masp1/Klk1b3/Klk1c9/Klk1/F2/Klk9/Klk8/Klk10/Tpsg1/Mcpt10/Cfd/Pcsk6	15	MF
GO:0046906	tetrapyrrole binding	0.00010722	Cyp4b1/Hbb/Ptgis/Cyp2d5/Cyp8b1/Hba-a1/Cyp2d2/Hbb-b1/Cyp2c24/Cyp4a1/Cyp4f1/Cbs/Hba-a2	13	MF
GO:0004601	peroxidase activity	0.000113977	Hbb/Hba-a1/Prdx6/Mgst1/Hba-a2/Ltc4s/Gsta1	7	MF
GO:0004806	triglyceride lipase activity	0.000146599	Ces1e/Pnpla3/Ces1d/Lpl/Aadac	5	MF
GO:0016684	oxidoreductase activity, acting on peroxide as acceptor	0.00018537	Hbb/Hba-a1/Prdx6/Mgst1/Hba-a2/Ltc4s/Gsta1	7	MF
GO:0031406	carboxylic acid binding	0.000222198	Adh5/Crabp2/Thnsl2/Snca/Pparg/Pck1/Fabp2/Cyp4f1/Ugt1a1/Phyh/Cps1/Fabp4/Adipoq/Ptgds	14	MF
GO:0017046	peptide hormone binding	0.000232649	Ramp2/Avpr1a/Lepr/Ramp1/Prlr/Oxtr/Cckar	7	MF
GO:0072341	modified amino acid binding	0.000251402	Gstm5/Anxa13/Gstm1/Cps1/Syt7/Cbs/Mgst1/Ltc4s/Gsta1	9	MF
GO:0005344	oxygen carrier activity	0.000253364	Hbb/Hba-a1/Hbb-b1/Hba-a2	4	MF
GO:0019825	oxygen binding	0.000315485	Hbb/Hba-a1/Hbb-b1/Cbs/Hba-a2	5	MF
GO:0008188	neuropeptide re-	0.000468955	Sstr5/Mc2r/Tacr3/Prokr2/Sstr1/Sstr2	6	MF

	ceptor activity				
GO:0001664	G protein-coupled receptor binding	0.000562223	Ramp2/Ccl24/Gcg/Pf4/Tac4/Nmu/Sfrp1/Avpr1a/Ppy/Ramp1/Wnt2b/Mrap2/Pyy/Ccl11/Edn2/Agt	16	MF
GO:0016765	transferase activity, transferring alkyl or aryl (other than methyl) groups	0.000579364	Cth/Gstm5/Gstm1/Cbs/Mgst1/Ltc4s/Gsta1	7	MF
GO:0019842	vitamin binding	0.000648258	Calb1/Cth/Aadat/Dhtkd1/Crabp2/Thnsl2/Oat/Phyh/Cbs/Rbp2	10	MF
GO:0031489	myosin V binding	0.000671342	Rab27a/Rab27b/Mlph/Npc1l1	4	MF
GO:0004364	glutathione transferase activity	0.000919359	Gstm5/Gstm1/Mgst1/Ltc4s/Gsta1	5	MF
GO:0050542	icosanoid binding	0.001109202	Snca/Pparg/Cyp4f1	3	MF
GO:0050543	icosatetraenoic acid binding	0.001109202	Snca/Pparg/Cyp4f1	3	MF
GO:0004602	glutathione peroxidase activity	0.001203078	Prdx6/Mgst1/Ltc4s/Gsta1	4	MF
GO:0008235	metalloexopeptidase activity	0.001230962	Ace2/Trhde/Cpxm2/Cpb2/Cpn1/Cpq	6	MF
GO:0017147	Wnt-protein binding	0.001347373	Sfrp4/Fzd4/Egf/Sfrp1/Fzd10	5	MF
GO:0016782	transferase activity, transferring sulfur-containing groups	0.001350319	Chst13/Tst/Sult1c2a/Sult5a1/Gal3st1/Chst9/Chst4	7	MF
GO:0008146	sulfotransferase activity	0.001485618	Chst13/Sult1c2a/Sult5a1/Gal3st1/Chst9/Chst4	6	MF
GO:0030545	receptor regulator activity	0.001638167	Retnla/Grem2/Cdc42ep2/Ccl24/Gcg/Egf/Pf4/Tgfb3/Tac4/Efemp1/Dkk2/Ppy/Mrap2/Bmp8a/Bmp6/Flrt3/Pyy/Adipoq/Ccl11/Edn2/Agt	21	MF
GO:0061134	peptidase regulator activity	0.001840287	Serpina1/Serpina10/Fbln1/Snca/Spock1/Serpinf1/Spink3/Spink4/Pzp/PCOLCE2/Cst6/Agt/Gpc3	13	MF
GO:0031994	insulin-like growth factor I binding	0.001968447	Igfbp6/Igfbp3/Igfbp1	3	MF
GO:0086008	voltage-gated potassium channel activity involved in cardiac muscle cell action potential repolarization	0.001968447	Kcnh2/Kcnj5/Kcne3	3	MF
GO:0004180	carboxypeptidase activity	0.002126386	Ace2/Cpxm2/Cpb2/Cpn1/Cpq	5	MF
GO:0016769	transferase activity, transferring nitrogenous groups	0.002295224	Aadat/Oat/Bcat2/Gatm	4	MF
GO:0016209	antioxidant activity	0.002580134	Hbb/Hba-a1/Prdx6/Mgst1/Hba-a2/Ltc4s/Gsta1	7	MF
GO:0004181	metallocarboxypeptidase activity	0.002648508	Ace2/Cpxm2/Cpb2/Cpn1	4	MF

GO:0140104	molecular carrier activity	0.002895337	Hbb/Hba-a1/Hbb-b1/Tf/Hba-a2	5	MF
GO:0005520	insulin-like growth factor binding	0.003037345	Igfbp6/Igfbp3/Igfbp1/Esm1	4	MF
GO:0042562	hormone binding	0.0031952	Ramp2/Avpr1a/Lepr/Chrna4/Ramp1/Prlr/Oxtr/Cckar	8	MF
GO:0008509	anion transmem-brane transporter activity	0.003222832	Slc35a1/Slc38a11/Slc16a7/Slc1a7/Slc16a12/Slc26a7/Slc16a5/Slc4a10/Clca1/Ano4/Ankh/Slc18a1/Slc6a19/Slc12a8/Cftr	15	MF
GO:0048018	receptor ligand activity	0.003529318	Retnla/Grem2/Cdc42ep2/Ccl24/Gcg/Egf/Pf4/Tgfb3/Tac4/Efemp1/Ppy/Bmp8a/Bmp6/Flrt3/Pyy/Adipoq/Ccl11/Edn2/Agt	19	MF
GO:0004497	monooxygenase activity	0.00368018	Cyp4b1/Ptgis/Cyp2d5/Cyp8b1/Cyp2d2/Cmahp/Cyp2c24/Cyp4a1/Cyp4f1	9	MF
GO:0005501	retinoid binding	0.004433746	Crabp2/Ugt1a1/Ptgds/Rbp2	4	MF
GO:0052689	carboxylic ester hydrolase activity	0.004846838	Rgn/Ces1e/Ces2c/Ces2a/Pnpla3/Ces1d/Lpl/Prdx6/Aadac	9	MF
GO:0005217	intracellular lig-and-gated ion channel activity	0.004981178	Jph1/Aqp1/Pex5l/Cftr	4	MF
GO:0071855	neuropeptide re-ceptor binding	0.004981178	Tac4/Nmu/Mrap2/Edn2	4	MF
GO:0030414	peptidase inhibitor activity	0.005362151	Serpi-na1/Serpina10/Snca/Spock1/Serpinf1/Spink3/Spink4/Pzp/Cst6/Agt/Gpc3	11	MF
GO:0001968	fibronectin binding	0.005572153	Igfbp6/Fbln1/Igfbp3/Ccdc80	4	MF
GO:0030170	pyridoxal phos-phate binding	0.005901483	Cth/Aadat/Thnsl2/Oat/Cbs	5	MF
GO:0004867	serine-type endo-peptidase inhibitor activity	0.006009331	Serpina1/Serpina10/Serpinf1/Spink3/Spink4/Pzp/Agt	7	MF
GO:0070279	vitamin B6 binding	0.006389706	Cth/Aadat/Thnsl2/Oat/Cbs	5	MF
GO:0030246	carbohydrate bind-ing	0.007057377	Grifin/Zg16/Fcna/Fbp1/Cd34/Galnt5/Asgr1/Asgr2/Phyh/Aldob/Itln1/Sftpa1/Reg4	13	MF
GO:0015106	bicarbonate trans-membrane trans-porter activity	0.007741478	Slc26a7/Slc4a10/Cftr	3	MF
GO:0042813	Wnt-activated receptor activity	0.007741478	Fzd4/Egf/Fzd10	3	MF
GO:0004866	endopeptidase inhibitor activity	0.008205057	Serpi-na1/Serpina10/Snca/Spock1/Serpinf1/Spink3/Spink4/Pzp/Cst6/Agt	10	MF
GO:0019840	isoprenoid binding	0.008400145	Crabp2/Ugt1a1/Ptgds/Rbp2	4	MF
GO:0046915	transition metal ion transmembrane transporter activity	0.008400145	Slc39a8/Slc30a2/Tf/Slc30a8	4	MF
GO:1901567	fatty acid deriva-tive binding	0.008400145	Snca/Pnpla3/Pparg/Cyp4f1	4	MF
GO:0019955	cytokine binding	0.00889623	Grem2/Fzd4/Tgfb3/Lepr/Ackr2/Ccr3/Il5ra/Prlr	8	MF
GO:0008198	ferrous iron bind-	0.009229883	Snca/Tf/Phyh/Cdo1	4	MF

	ing				
GO:0016712	oxidoreductase activity, acting on paired donors, with incorporation or reduction of molecular oxygen, reduced flavin or flavoprotein as one donor, and incorporation of one atom of oxygen	0.009260889	Cyp4b1/Cyp2d5/Cyp2d2/Cyp2c24/Cyp4f1	5	MF
GO:0005216	ion channel activity	0.009469137	Jph1/Kcnh2/Slc26a7/Kcnj5/Aqp1/Kcns1/Fxyd3/Clca1/Ano4/Kcnv2/Chrna4/Fxyd1/Pex5l/Kcne3/Htr1b/Cftr/Lrrc26	17	MF
GO:0015103	inorganic anion transmembrane transporter activity	0.010105827	Slc26a7/Slc4a10/Clca1/Ano4/Ankh/Slc18a1/Slc12a8/Cftr	8	MF
GO:0070330	aromatase activity	0.010111218	Cyp4b1/Cyp2d5/Cyp2d2/Cyp4f1	4	MF
GO:0005385	zinc ion transmembrane transporter activity	0.010288834	Slc39a8/Slc30a2/Slc30a8	3	MF
GO:0015267	channel activity	0.0106383	Jph1/Kcnh2/Slc26a7/Kcnj5/Aqp1/Kcns1/Fxyd3/Clca1/Ano4/Kcnv2/Chrna4/Fxyd1/Pex5l/Kcne3/Aqp8/Htr1b/Cftr/Lrrc26	18	MF
GO:0022803	passive transmembrane transporter activity	0.0106383	Jph1/Kcnh2/Slc26a7/Kcnj5/Aqp1/Kcns1/Fxyd3/Clca1/Ano4/Kcnv2/Chrna4/Fxyd1/Pex5l/Kcne3/Aqp8/Htr1b/Cftr/Lrrc26	18	MF
GO:0061135	endopeptidase regulator activity	0.010829775	Serpina1/Serpina10/Snca/Spock1/Serpinf1/Spink3/Spink4/Pzp/Cst6/Agt	10	MF
GO:0015464	acetylcholine receptor activity	0.011723427	Hrh4/Chrm1/Chrna4	3	MF
GO:0016614	oxidoreductase activity, acting on CH-OH group of donors	0.011903384	Adh5/Chdh/Sord/Cryl1/Adhfe1/Hsd17b2/Cyp4a1/Me3	8	MF
GO:0004993	G protein-coupled serotonin receptor activity	0.012033242	Hrh4/Chrm1/Htr1b/Htr5b	4	MF
GO:0099589	serotonin receptor activity	0.012033242	Hrh4/Chrm1/Htr1b/Htr5b	4	MF
GO:0005496	steroid binding	0.012649355	Calb1/Esrrb/Prom1/Ugt1a1/Gstm1/Apod	6	MF
GO:0008514	organic anion transmembrane transporter activity	0.012766897	Slc35a1/Slc38a11/Slc16a7/Slc1a7/Slc16a12/Slc26a7/Slc16a5/Slc4a10/Slc6a19/Cftr	10	MF
GO:0046873	metal ion transmembrane transporter activity	0.012855559	Jph1/Kcnh2/Slc39a8/Slc30a2/Kcnj5/Aqp1/Slc4a10/Kcns1/Kcnv2/Tf/Slc30a8/Kcne3/Slc18a1/Slc6a19/Slc12a8/Htr1b/Lrrc26	17	MF
GO:0022839	ion gated channel activity	0.013170041	Jph1/Kcnh2/Kcnj5/Aqp1/Kcns1/Clca1/Ano4/Kcnv2/Chrna4/Pex5l/Kcne3/Htr1b/Cftr/Lrrc26	14	MF
GO:0008307	structural constituent of muscle	0.013267219	Jph1/Myom2/Myl6	3	MF

GO:0008483	transaminase activity	0.013267219	Aadat/Oat/Bcat2	3	MF
GO:0005249	voltage-gated potassium channel activity	0.013321966	Kcnh2/Kcnj5/Kcns1/Kcnv2/Kcne3/Lrrc26	6	MF
GO:0016705	oxidoreductase activity, acting on paired donors, with incorporation or reduction of molecular oxygen	0.014048031	Cyp4b1/Ptgis/Cyp2d5/Cyp8b1/Cyp2d2/Cmahp/Cyp2c24/Cyp4a1/Cyp4f1/Phyh	10	MF
GO:0005342	organic acid transmembrane transporter activity	0.014468518	Slc35a1/Slc38a11/Slc16a7/Slc1a7/Slc16a12/Slc26a7/Slc16a5/Slc6a19	8	MF
GO:0046943	carboxylic acid transmembrane transporter activity	0.014468518	Slc35a1/Slc38a11/Slc16a7/Slc1a7/Slc16a12/Slc26a7/Slc16a5/Slc6a19	8	MF
GO:0004190	aspartic-type endopeptidase activity	0.014921328	Pgc/Pga5/Bace2	3	MF
GO:0042923	neuropeptide binding	0.014921328	Sstr5/Sstr1/Sstr2	3	MF
GO:0015291	secondary active transmembrane transporter activity	0.014954064	Slc16a7/Slc9c1/Slc1a7/Slc2a10/Slc26a7/Slc4a10/Ankh/Slc18a1/Slc6a19/Slc12a8	10	MF
GO:0019838	growth factor binding	0.015026296	Igfbp6/Tgfb3/Fgfrl1/Igfbp3/Pzp/Igfbp1/Pcsk6/Esm1	8	MF
GO:0022836	gated channel activity	0.015937274	Jph1/Kcnh2/Kcnj5/Aqp1/Kcns1/Clca1/Ano4/Kcnv2/Chrna4/Pex5l/Kcne3/Htr1b/Cftr/Lrrc26	14	MF
GO:0070001	aspartic-type peptidase activity	0.01668663	Pgc/Pga5/Bace2	3	MF
GO:0017022	myosin binding	0.018455754	Rab27a/Rab27b/Fxyd1/Mlph/Npc1l1	5	MF
GO:0015293	symporter activity	0.018466647	Slc16a7/Slc1a7/Slc2a10/Slc4a10/Slc18a1/Slc6a19/Slc12a8	7	MF
GO:0008237	metallopeptidase activity	0.018848823	Adam28/Mmp10/Ace2/Acy1/Trhde/Cpxm2/Cpb2/Cpn1/Cpq	9	MF
GO:0016846	carbon-sulfur lyase activity	0.01909752	Cth/Ltc4s	2	MF
GO:0019763	immunoglobulin receptor activity	0.01909752	Fcer1a/Ms4a2	2	MF
GO:0019841	retinol binding	0.01909752	Crabp2/Rbp2	2	MF
GO:0099528	G protein-coupled neurotransmitter receptor activity	0.01914593	Hrh4/Chrm1/Htr1b/Htr5b	4	MF
GO:0005179	hormone activity	0.019217821	Retnla/Gcg/Ppy/Pyy/Adipoq/Edn2/Agt	7	MF
GO:0015108	chloride transmembrane transporter activity	0.019624133	Slc26a7/Clca1/Ano4/Slc18a1/Slc12a8/Cftr	6	MF
GO:0072509	divalent inorganic cation transmembrane transporter	0.020553177	Slc39a8/Slc30a2/Slc30a8	3	MF

	activity				
GO:0008238	exopeptidase activity	0.021470006	Ace2/Trhde/Cpxm2/Cpb2/Cpn1/Cpq	6	MF
GO:0099094	ligand-gated cation channel activity	0.021470006	Jph1/Kcnh2/Kcnj5/Aqp1/Chrna4/Pex5l	6	MF
GO:0005267	potassium channel activity	0.022436625	Kcnh2/Kcnj5/Aqp1/Kcns1/Kcnv2/Kcne3/Lrrc26	7	MF
GO:0001530	lipopolysaccharide binding	0.022655077	F2/Sftpa1/Trem2	3	MF
GO:0002162	dystroglycan binding	0.023007962	Agr3/Agr2	2	MF
GO:0005221	intracellular cyclic nucleotide activated cation channel activity	0.023007962	Aqp1/Pex5l	2	MF
GO:0043855	cyclic nucleotide-gated ion channel activity	0.023007962	Aqp1/Pex5l	2	MF
GO:0050308	sugar-phosphatase activity	0.023007962	Fbp1/G6pc	2	MF
GO:0070700	BMP receptor binding	0.023007962	Bmp8a/Bmp6	2	MF
GO:0016616	oxidoreductase activity, acting on the CH-OH group of donors, NAD or NADP as acceptor	0.023296172	Adh5/Sord/Cryl1/Adhfe1/Hsd17b2/Cyp4a1/Me3	7	MF
GO:0005251	delayed rectifier potassium channel activity	0.0248695	Kcnh2/Kcns1/Kcne3	3	MF
GO:0015276	ligand-gated ion channel activity	0.025082857	Jph1/Kcnh2/Kcnj5/Aqp1/Chrna4/Pex5l/Cftr	7	MF
GO:0022834	ligand-gated channel activity	0.025082857	Jph1/Kcnh2/Kcnj5/Aqp1/Chrna4/Pex5l/Cftr	7	MF
GO:0015079	potassium ion transmembrane transporter activity	0.026200264	Kcnh2/Kcnj5/Aqp1/Kcns1/Kcnv2/Kcne3/Slc12a8/Lrrc26	8	MF
GO:0019203	carbohydrate phosphatase activity	0.027215865	Fbp1/G6pc	2	MF
GO:0043121	neurotrophin binding	0.027215865	Pzp/Pcsk6	2	MF
GO:0051378	serotonin binding	0.027215865	Htr1b/Htr5b	2	MF
GO:0070405	ammonium ion binding	0.027996787	Gpr119/Chrna4/Aldob/Htr1b/Htr5b	5	MF
GO:0005326	neurotransmitter transmembrane transporter activity	0.028409505	Slc1a7/Aqp1/Slc18a1/Slc6a19	4	MF
GO:0008391	arachidonic acid monooxygenase	0.02963514	Cyp2c24/Cyp4a1/Cyp4f1	3	MF

	activity				
GO:0005324	long-chain fatty acid transporter activity	0.031706429	Fabp2/Fabp4	2	MF
GO:0015250	water channel activity	0.031706429	Aqp1/Aqp8	2	MF
GO:0016918	retinal binding	0.031706429	Crabp2/Rbp2	2	MF
GO:0042043	neurexin family protein binding	0.031706429	Sytl1/Sytl4	2	MF
GO:0016903	oxidoreductase activity, acting on the aldehyde or oxo group of do-nors	0.031995835	Adh5/Dhtkd1/Aldh1b1/Aox3	4	MF
GO:0016247	channel regulator activity	0.033164935	Kcns1/Fxyd3/Fxyd1/Tspan13/Kcne3/Cftr/Lrrc26	7	MF
GO:0008395	steroid hydrox-ylase activity	0.03583573	Cyp2d5/Cyp8b1/Cyp2d2/Cyp2c24	4	MF
GO:0005372	water transmem-brane transporter activity	0.036465356	Aqp1/Aqp8	2	MF
GO:0043176	amine binding	0.036465356	Htr1b/Htr5b	2	MF
GO:0004896	cytokine receptor activity	0.038520812	Lepr/Ackr2/Ccr3/Il5ra/Prlr	5	MF
GO:0016298	lipase activity	0.039113638	Ces1e/Pnpla3/Ces1d/Lpl/Prdx6/Aadac	6	MF
GO:0005154	epidermal growth factor receptor binding	0.040499113	Agr2/Egf/Efemp1	3	MF
GO:0050840	extracellular ma-trix binding	0.042073654	Dcn/Cd248/Shh/Bcam	4	MF
GO:0022843	voltage-gated cation channel activity	0.04282173	Kcnh2/Kcnj5/Kcns1/Kcnv2/Kcne3/Htr1b/Lrrc26	7	MF
GO:0008378	galactosyltransfer-ase activity	0.043488051	Ugt8/B3gnt6/B3galt1	3	MF
GO:0042165	neurotransmitter binding	0.044280649	Chrna4/Cbs/Htr1b/Htr5b	4	MF
GO:0008009	chemokine activity	0.046584043	Ccl24/Pf4/Ccl11	3	MF
GO:0001972	retinoic acid bind-ing	0.046733528	Crabp2/Ugt1a1	2	MF
GO:0004029	aldehyde dehydro-genase (NAD+) activity	0.046733528	Adh5/Aldh1b1	2	MF
GO:0004198	calcium-dependent cysteine-type en-dopeptidase activi-ty	0.046733528	Capn8/Capn9	2	MF
GO:0015377	cation:chloride symporter activity	0.046733528	Slc18a1/Slc12a8	2	MF

GO:0015144	carbohydrate transmembrane transporter activity	0.049785828	RGD1304770/Slc2a10/Aqp1	3	MF

ID represents the serial number of GO, Description represents the description information corresponding to GO, GeneID indicates the names of all the enriched genes in this GO term, which are separated by "/",Count indicates the number of all enriched genes on this GO term.

Support Table 9. KEGG enrichment analysis of 429 DE mRNAs down-regulated by EA.

ID	Description	pvalue	GeneID	Count	GeneSymbol	KEGGLink	Level_a	Level_b
rno05332	Graft-versus-host disease	5.58934E-23	414779/294228/406194/171528/414783/24493/360323/25408/294273/24973/414792/414789/24835/309603/368153/309607/24974/414270/56822/24494/25712/24750/414788/414819	24	RT1-CE2/RT1-S3/RT1-T18/Gzmb/RT1-CE4/Il1a/RT1-N2/Cd80/RT1-DMb/RT1-A1/RT1-CE10/RT1-CE15/Tnf/RT1-CE1/RT1-CE7/RT1-CE5/RT1-A2/RT1-CE14/Cd86/Il1b/Ifng/RT1-N3/RT1-T24-3/RT1-CE16	https://www.kegg.jp/kegg-bin/show_pathway?rno05332/414779%09red/294228%09red/406194%09red/171528%09red/414783%09red/24493%09red/360323%09red/25408%09red/294273%09red/24973%09red/414792%09red/414789%09red/24835%09red/309603%09red/368153%09red/309607%09red/24974%09red/414270%09red/56822%09red/24494%09red/25712%09red/24750%09red/414788%09red/414819%09red	Human Diseases	Immune disease
rno05169	Epstein-Barr virus infection	6.74885E-22	414779/24811/288774/294228/297989/25124/310553/24812/406194/414783/298693/360323/24223/294273/78963/64625/192281/156726/24973/414792/414789/24835/309603/368153/29513/494202/309607/246268/54287/24974/414270/29362	39	RT1-CE2/Tap1/Stat2/RT1-S3/Ddx58/Stat1/Tlr2/Tap2/RT1-T18/RT1-CE4/Isg15/RT1-N2/B2m/RT1-DMb/Apaf1/Bid/Oas1a/Runx3/RT1-A1/RT1-CE10/RT1-CE15/Tnf/RT1-CE1/RT1-	https://www.kegg.jp/kegg-bin/show_pathway?rno05169/414779%09red/24811%09red/288774%09red/294228%09red/297989%09red/25124%09red/310553%09red/24812%09red/406194%09red/414783%09red/298693%09red/360323%09red/	Human Diseases	Infectious disease: viral

			4/363938/304507/24750/414788/245920/414819/25326		CE7/Mapk13/Oas3/RT1-CE5/Oas1b/Eif2ak2/RT1-A2/RT1-CE14/Irf7/Oas2/Oas1i/RT1-N3/RT1-T24-3/Cxcl10/RT1-CE16/Jak3	24223%09red/294273%09red/78963%09red/64625%09red/192281%09red/156726%09red/24973%09red/414792%09red/414789%09red/24835%09red/309603%09red/368153%09red/29513%09red/494202%09red/309607%09red/246268%09red/54287%09red/24974%09red/414270%09red/293624%09red/363938%09red/304507%09red/24750%09red/414788%09red/245920%09red/414819%09red/25326%09red		
rno04940	Type I diabetes mellitus	1.81069E-21	414779/294228/406194/171528/414783/24493/360323/25408/294273/24973/414792/414789/24835/309603/368153/309607/24974/414270/56822/24494/25712/24750/414788/414819	24	RT1-CE2/RT1-S3/RT1-T18/Gzmb/RT1-CE4/Il1a/RT1-N2/Cd80/RT1-DMb/RT1-A1/RT1-CE10/RT1-CE15/Tnf/RT1-CE1/RT1-CE7/RT1-CE5/RT1-A2/RT1-CE14/Cd86/Il1b/Ifng/RT1-N3/RT1-T24-3/RT1-CE16	https://www.kegg.jp/kegg-bin/show_pathway?rno04940/414779%09red/294228%09red/406194%09red/171528%09red/414783%09red/24493%09red/360323%09red/25408%09red/294273%09red/24973%09red/414792%09red/414789%09red/24835%09red/309603%09red/368153%09red/309607%09red/24974%09red/414270%09red/56822%09red/24494%09red/25712%09red/24750%09red/414788%0	Human Diseases	Endocrine and metabolic disease

						9red/414819%09red		
rno05330	Allograft rejection	2.30246E-21	414779/294228/406194/171528/25325/414783/360323/25408/294273/24973/414792/414789/24835/309603/368153/309607/24974/414270/56822/25712/24750/414788/414819	23	RT1-CE2/RT1-S3/RT1-T18/Gzmb/Il10/RT1-CE4/RT1-N2/Cd80/RT1-DMb/RT1-A1/RT1-CE10/RT1-CE15/Tnf/RT1-CE1/RT1-CE7/RT1-CE5/RT1-A2/RT1-CE14/Cd86/Ifng/RT1-N3/RT1-T24-3/RT1-CE16	https://www.kegg.jp/kegg-bin/show_pathway?rno05330/414779%09red/294228%09red/406194%09red/171528%09red/25325%09red/414783%09red/360323%09red/25408%09red/294273%09red/24973%09red/414792%09red/414789%09red/24835%09red/309603%09red/368153%09red/309607%09red/24974%09red/414270%09red/56822%09red/25712%09red/24750%09red/414788%09red/414819%09red	Human Diseases	Immune disease
rno04612	Antigen processing and presentation	8.96869E-19	414779/25599/24811/294228/24812/406194/85483/414783/360323/24223/294273/24973/414792/414789/24835/309603/368153/309607/24974/414270/25712/24750/414788/414819	24	RT1-CE2/Cd74/Tap1/RT1-S3/Tap2/RT1-T18/Ciita/RT1-CE4/RT1-N2/B2m/RT1-DMb/RT1-A1/RT1-CE10/RT1-CE15/Tnf/RT1-CE1/RT1-CE7/RT1-CE5/RT1-A2/RT1-CE14/Ifng/RT1-N3/RT1-T24-3/RT1-CE16	https://www.kegg.jp/kegg-bin/show_pathway?rno04612/414779%09red/25599%09red/24811%09red/294228%09red/24812%09red/406194%09red/85483%09red/414783%09red/360323%09red/24223%09red/294273%09red/24973%09red/414792%09red/414789%09red/24835%09red/309603%09red/368153%09red/309607%09red/24974%09red/414270%09red/25712%09re	Organismal Systems	Immune system

						d/24750%09red/414788%09red/414819%09red		
rno05320	Autoimmune thyroid disease	2.98887E-17	414779/294228/406194/171528/25325/414783/360323/25408/294273/24973/414792/414789/309603/368153/309607/24974/414270/56822/24750/414788/414819	21	RT1-CE2/RT1-S3/RT1-T18/Gzmb/Il10/RT1-CE4/RT1-N2/Cd80/RT1-DMb/RT1-A1/RT1-CE10/RT1-CE15/RT1-CE1/RT1-CE7/RT1-CE5/RT1-A2/RT1-CE14/Cd86/RT1-N3/RT1-T24-3/RT1-CE16	https://www.kegg.jp/kegg-bin/show_pathway?rno05320/414779%09red/294228%09red/406194%09red/171528%09red/25325%09red/414783%09red/360323%09red/25408%09red/294273%09red/24973%09red/414792%09red/414789%09red/309603%09red/368153%09red/309607%09red/24974%09red/414270%09red/56822%09red/24750%09red/414788%09red/414819%09red	Human Diseases	Immune disease
rno05168	Herpes simplex virus 1 infection	6.23739E-16	414779/25599/24811/288774/294228/297989/25124/310553/24812/406194/24232/89829/414783/360323/24223/117058/294273/78963/64625/192281/24973/78971/414792/414789/24835/309603/368153/494202/309607/246268/54287/24974/414270/293624/24494/363938/304507/25712/24750/499801/414788/414819	42	RT1-CE2/Cd74/Tap1/Stat2/RT1-S3/Ddx58/Stat1/Tlr2/Tap2/RT1-T18/C3/Socs3/RT1-CE4/RT1-N2/B2m/Pou2f2/RT1-DMb/Apaf1/Bid/Oas1a/RT1-A1/Birc3/RT1-CE10/RT1-CE15/Tnf/RT1-CE1/RT1-CE7/Oas3/RT1-CE5/Oas1b/Eif2ak2/RT1-A2/RT1-CE14/Irf7/Il1b/Oas2/Oas1i/Ifng/RT1-N3/Ifih1/RT1	https://www.kegg.jp/kegg-bin/show_pathway?rno05168/414779%09red/25599%09red/24811%09red/288774%09red/294228%09red/297989%09red/25124%09red/310553%09red/24812%09red/406194%09red/24232%09red/89829%09red/414783%09red/360323%09red/24223%09red/117058%09red/294273%09red/78963%09red/64625%09red/192281%09red/24973%09red	Human Diseases	Infectious disease: viral

					-T24-3/RT1-CE16	/78971%09red/414792%09red/414789%09red/24835%09red/309603%09red/368153%09red/494202%09red/309607%09red/246268%09red/54287%09red/24974%09red/414270%09red/293624%09red/24494%09red/363938%09red/304507%09red/25712%09red/24750%09red/499801%09red/414788%09red/414819%09red		
rno05416	Viral myo-carditis	1.72645E-15	414779/294228/406194/414783/360323/25408/294273/64625/24973/414792/414789/309603/368153/309607/24974/414270/316758/56822/24750/414788/414819	21	RT1-CE2/RT1-S3/RT1-T18/RT1-CE4/RT1-N2/Cd80/RT1-DMb/Bid/RT1-A1/RT1-CE10/RT1-CE15/RT1-CE1/RT1-CE7/RT1-CE5/RT1-A2/RT1-CE14/Lama1/Cd86/RT1-N3/RT1-T24-3/RT1-CE16	https://www.kegg.jp/kegg-bin/show_pathway?rno05416/414779%09red/294228%09red/406194%09red/414783%09red/360323%09red/25408%09red/294273%09red/64625%09red/24973%09red/414792%09red/414789%09red/309603%09red/368153%09red/309607%09red/24974%09red/414270%09red/316758%09red/56822%09red/24750%09red/414788%09red/414819%09red	Human Dis-eases	Cardiovascu-lar disease
rno05164	Influenza A	1.60333E-14	361384/156435/288774/297989/25124/89829/85483/24493/81635/286918/65190/294273/7896	27	Dnajb1/Tmprss2/Stat2/Ddx58/Stat1/Socs3/Ciita/Il1a/Adar/Mx2/Rsad2/RT1-DMb/Apaf1/	https://www.kegg.jp/kegg-bin/show_pathway?rno05164/361384%09red/156435%09red/2887	Human Dis-eases	Infectious disease: viral

			3/64625/192281/24835/494202/246268/54287/287362/293624/24494/363938/304507/25712/499801/245920		Bid/Oas1a/Tnf/Oas3/Oas1b/Eif2ak2/Nlrp3/Irf7/Il1b/Oas2/Oas1i/Ifng/Ifih1/Cxcl10	74%09red/297989%09red/25124%09red/89829%09red/85483%09red/24493%09red/81635%09red/286918%09red/65190%09red/294273%09red/78963%09red/64625%09red/192281%09red/24835%09red/494202%09red/246268%09red/54287%09red/287362%09red/293624%09red/24494%09red/363938%09red/304507%09red/25712%09red/499801%09red/245920%09red		
rno04145	Phagosome	1.5713E-13	414779/24811/294228/310553/24812/406194/24232/304966/414783/29564/502902/360323/114553/294273/24973/414792/414789/500904/309603/368153/309607/24974/414270/293650/24750/414788/414819	27	RT1-CE2/Tap1/RT1-S3/Tlr2/Tap2/RT1-T18/C3/Fcgr3a/RT1-CE4/Dync1i1/Clec7a/RT1-N2/Ncf1/RT1-DMb/RT1-A1/RT1-CE10/RT1-CE15/Ncf4/RT1-CE1/RT1-CE7/RT1-CE5/RT1-A2/RT1-CE14/Tcirg1/RT1-N3/RT1-T24-3/RT1-CE16	https://www.kegg.jp/kegg-bin/show_pathway?rno04145/414779%09red/24811%09red/294228%09red/310553%09red/24812%09red/406194%09red/24232%09red/304966%09red/414783%09red/29564%09red/502902%09red/360323%09red/114553%09red/294273%09red/24973%09red/414792%09red/414789%09red/500904%09red/309603%09red/368153%09red/309607%09red/24974%09red/414270%09red/293650%09red/24750%09red	Cellular Processes	Transport and catabolism

						/414788%09red/414819%09red		
rno05163	Human cytomegalovirus infection	1.65928E-13	414779/24811/294228/24329/116637/24812/406194/25637/117029/414783/25542/360323/24223/89808/64625/24973/414792/414789/314638/24835/309603/368153/29513/309607/24974/414270/24494/117539/24750/414788/414819	31	RT1-CE2/Tap1/RT1-S3/Egfr/Ccl4/Tap2/RT1-T18/Ptger1/Ccr5/RT1-CE4/Ccl3/RT1-N2/B2m/Cx3cl1/Bid/RT1-A1/RT1-CE10/RT1-CE15/Creb3l3/Tnf/RT1-CE1/RT1-CE7/Mapk13/RT1-CE5/RT1-A2/RT1-CE14/Il1b/Il10ra/RT1-N3/RT1-T24-3/RT1-CE16	https://www.kegg.jp/kegg-bin/show_pathway?rno05163/414779%09red/24811%09red/294228%09red/24329%09red/116637%09red/24812%09red/406194%09red/25637%09red/117029%09red/414783%09red/25542%09red/360323%09red/24223%09red/89808%09red/64625%09red/24973%09red/414792%09red/414789%09red/314638%09red/24835%09red/309603%09red/368153%09red/29513%09red/309607%09red/24974%09red/414270%09red/24494%09red/117539%09red/24750%09red/414788%09red/414819%09red	Human Diseases	Infectious disease: viral
rno04060	Cytokine-cytokine receptor interaction	2.62376E-13	84475/500836/301291/116637/25610/25325/29373/298518/301289/117029/171081/24493/81811/25542/89808/24835/25069/171551/497942/365769/171333/60582/289747/353218/60665/24494/117539/25712/691516/245920/8150	32	Cxcr3/Il22/Il17f/Ccl4/Csf3/Il10/Bmp2/Csf3r/Il17a/Ccr5/Csf2rb/Il1a/Thpo/Ccl3/Cx3cl1/Tnf/Tnfrsf8/Cxcl3/Cxcl16/Il21/Il12rb1/Il1rn/Osm/Tnfsf9/Cxcl6/Il1b/Il10ra/Ifng/Il17c/Cxcl10/Cxcl1/Cxcl9	https://www.kegg.jp/kegg-bin/show_pathway?rno04060/84475%09red/500836%09red/301291%09red/116637%09red/25610%09red/25325%09red/29373%09red/298518%09red/301289%09red/117029%09red/171081%09red/24493%09red/8	Environmental Information Processing	Signaling molecules and interaction

			3/246759			1811%09red/25542%09red/89808%09red/24835%09red/25069%09red/171551%09red/497942%09red/365769%09red/171333%09red/60582%09red/289747%09red/353218%09red/60665%09red/24494%09red/117539%09red/25712%09red/691516%09red/245920%09red/81503%09red/246759%09red		
rno05167	Kaposi sarcoma-associated herpesvirus infection	1.10324E-12	414779/288774/294228/25124/406194/24232/117029/414783/360323/64625/24973/414792/414789/79426/309603/368153/29513/171551/309607/54287/24974/414270/293624/56822/24750/414788/414819/81503	28	RT1-CE2/Stat2/RT1-S3/Stat1/RT1-T18/C3/Ccr5/RT1-CE4/RT1-N2/Bid/RT1-A1/RT1-CE10/RT1-CE15/Zfp36/RT1-CE1/RT1-CE7/Mapk13/Cxcl3/RT1-CE5/Eif2ak2/RT1-A2/RT1-CE14/Irf7/Cd86/RT1-N3/RT1-T24-3/RT1-CE16/Cxcl1	https://www.kegg.jp/kegg-bin/show_pathway?rno05167/414779%09red/288774%09red/294228%09red/25124%09red/406194%09red/24232%09red/117029%09red/414783%09red/360323%09red/64625%09red/24973%09red/414792%09red/414789%09red/79426%09red/309603%09red/368153%09red/29513%09red/171551%09red/309607%09red/54287%09red/24974%09red/414270%09red/293624%09red/56822%09red/24750%09red/414788%09red/414819%09red/81503%09red	Human Diseases	Infectious disease: viral

rno04621	NOD-like receptor signaling pathway	3.19917E-12	288774/25124/310917/156117/114555/192281/78971/294329/24835/246240/29513/494202/171551/246268/287362/171164/315084/293624/24494/362050/363938/304507/291912/58923/81503	25	Stat2/Stat1/Gbp4/Casp12/Casp4/Oas1a/Birc3/Trpm2/Tnf/Ripk3/Mapk13/Oas3/Cxcl3/Oas1b/Nlrp3/Gbp2/Gsdmd/Irf7/Il1b/Gbp5/Oas2/Oas1i/Nod2/Mefv/Cxcl1	https://www.kegg.jp/kegg-bin/show_pathway?rno04621/288774%09red/25124%09red/310917%09red/156117%09red/114555%09red/192281%09red/78971%09red/294329%09red/24835%09red/246240%09red/29513%09red/494202%09red/171551%09red/246268%09red/287362%09red/171164%09red/315084%09red/293624%09red/24494%09red/362050%09red/363938%09red/304507%09red/291912%09red/58923%09red/81503%09red	Organismal Systems	Immune system
rno05152	Tuberculosis	1.24878E-10	25599/25124/310553/29469/24232/304966/24599/305354/25325/85483/24493/502902/294273/78963/64625/24835/29513/24494/117539/25712/293650/58936/291912	23	Cd74/Stat1/Tlr2/Lbp/C3/Fcgr3a/Nos2/Tlr1/Il10/Ciita/Il1a/Clec7a/RT1-DMb/Apaf1/Bid/Tnf/Mapk13/Il1b/Il10ra/Ifng/Tcirg1/Plk3/Nod2	https://www.kegg.jp/kegg-bin/show_pathway?rno05152/25599%09red/25124%09red/310553%09red/29469%09red/24232%09red/304966%09red/24599%09red/305354%09red/25325%09red/85483%09red/24493%09red/502902%09red/294273%09red/78963%09red/64625%09red/24835%09red/29513%09red/24494%09red/117539%09red/25712%09red/293650%09red/5893	Human Diseases	Infectious disease: bacterial

						6%09red/291912%09red		
rno05170	Human immunodeficiency virus 1 infection	2.49857E-10	414779/24811/315137/294228/310553/24812/406194/117029/414783/360323/24223/64625/24973/414792/414789/24835/309603/368153/29513/309607/311580/24974/414270/24750/414788/414819	26	RT1-CE2/Tap1/Apobec3/RT1-S3/Tlr2/Tap2/RT1-T18/Ccr5/RT1-CE4/RT1-N2/B2m/Bid/RT1-A1/RT1-CE10/RT1-CE15/Tnf/RT1-CE1/RT1-CE7/Mapk13/RT1-CE5/Samhd1/RT1-A2/RT1-CE14/RT1-N3/RT1-T24-3/RT1-CE16	https://www.kegg.jp/kegg-bin/show_pathway?rno05170/414779%09red/24811%09red/315137%09red/294228%09red/310553%09red/24812%09red/406194%09red/117029%09red/414783%09red/360323%09red/24223%09red/64625%09red/24973%09red/414792%09red/414789%09red/24835%09red/309603%09red/368153%09red/29513%09red/309607%09red/311580%09red/24974%09red/414270%09red/24750%09red/414788%09red/414819%09red	Human Diseases	Infectious disease: viral
rno05321	Inflammatory bowel disease	2.93112E-10	500836/301291/25124/310553/25325/301289/24493/294273/24835/365769/171333/24494/25712/291912	14	Il22/Il17f/Stat1/Tlr2/Il10/Il17a/Il1a/RT1-DMb/Tnf/Il21/Il12rb1/Il1b/Ifng/Nod2	https://www.kegg.jp/kegg-bin/show_pathway?rno05321/500836%09red/301291%09red/25124%09red/310553%09red/25325%09red/301289%09red/24493%09red/294273%09red/24835%09red/365769%09red/171333%09red/24494%09red/25712%09red/291912%09red	Human Diseases	Immune disease
rno05203	Viral carcinogenesis	2.04487E-09	414779/294228/406194/24232/117029/4	24	RT1-CE2/RT1-S3/RT1-	https://www.kegg.jp/kegg-bin/show_pat	Human Diseases	Cancer: overview

			14783/360323/492821/24973/414792/414789/314638/309603/368153/309607/54287/24974/414270/293624/114090/24750/414788/414819/25326		T18/C3/Ccr5/RT1-CE4/RT1-N2/Pmaip1/RT1-A1/RT1-CE10/RT1-CE15/Creb3l3/RT1-CE1/RT1-CE7/RT1-CE5/Eif2ak2/RT1-A2/RT1-CE14/Irf7/Egr2/RT1-N3/RT1-T24-3/RT1-CE16/Jak3	hway?rno05203/414779%09red/294228%09red/406194%09red/24232%09red/117029%09red/414783%09red/360323%09red/492821%09red/24973%09red/414792%09red/414789%09red/314638%09red/309603%09red/368153%09red/309607%09red/54287%09red/24974%09red/414270%09red/293624%09red/114090%09red/24750%09red/414788%09red/414819%09red/25326%09red		
rno05140	Leishmaniasis	2.49359E-09	25124/310553/24232/304966/24599/25325/24493/114553/294273/24835/500904/29513/24494/25712	14	Stat1/Tlr2/C3/Fcgr3a/Nos2/Il10/Il1a/Ncf1/RT1-DMb/Tnf/Ncf4/Mapk13/Il1b/Ifng	https://www.kegg.jp/kegg-bin/show_pathway?rno05140/25124%09red/310553%09red/24232%09red/304966%09red/24599%09red/25325%09red/24493%09red/114553%09red/294273%09red/24835%09red/500904%09red/29513%09red/24494%09red/25712%09red	Human Diseases	Infectious disease: parasitic
rno04668	TNF signaling pathway	2.84402E-09	89829/89808/78971/24508/314638/24835/246240/29513/171551/246208/60665/81687/24494/680611/245920/291912/81503	17	Socs3/Cx3cl1/Birc3/Irf1/Creb3l3/Tnf/Ripk3/Mapk13/Cxcl3/Ifi47/Cxcl6/Mmp9/Il1b/Bcl3/Cxcl10/Nod2/Cxcl1	https://www.kegg.jp/kegg-bin/show_pathway?rno04668/89829%09red/89808%09red/78971%09red/24508%09red/314638%09red/24835%09red/246240%09red	Environmental Information Processing	Signal transduction

						/29513%09red/171551%09red/246208%09red/60665%09red/81687%09red/24494%09red/680611%09red/245920%09red/291912%09red/81503%09red		
rno05162	Measles	4.25647E-09	288774/297989/25124/310553/24493/81635/286918/78963/64625/192281/494202/246268/54287/293624/24494/363938/304507/499801/25326	19	Stat2/Ddx58/Stat1/Tlr2/Il1a/Adar/Mx2/Apaf1/Bid/Oas1a/Oas3/Oas1b/Eif2ak2/Irf7/Il1b/Oas2/Oas1i/Ifih1/Jak3	https://www.kegg.jp/kegg-bin/show_pathway?rno05162/288774%09red/297989%09red/25124%09red/310553%09red/24493%09red/81635%09red/286918%09red/78963%09red/64625%09red/192281%09red/494202%09red/246268%09red/54287%09red/293624%09red/24494%09red/363938%09red/304507%09red/499801%09red/25326%09red	Human Diseases	Infectious disease: viral
rno04514	Cell adhesion molecules	1.50894E-08	414779/294228/301626/406194/414783/360323/25408/294273/24973/414792/414789/309603/368153/309607/24974/414270/56822/24750/414788/414819	20	RT1-CE2/RT1-S3/Pdcd1/RT1-T18/RT1-CE4/RT1-N2/Cd80/RT1-DMb/RT1-A1/RT1-CE10/RT1-CE15/RT1-CE1/RT1-CE7/RT1-CE5/RT1-A2/RT1-CE14/Cd86/RT1-N3/RT1-T24-3/RT1-CE16	https://www.kegg.jp/kegg-bin/show_pathway?rno04514/414779%09red/294228%09red/301626%09red/406194%09red/414783%09red/360323%09red/25408%09red/294273%09red/24973%09red/414792%09red/414789%09red/309603%09red/368153%09red/309607%09red/24974%09red/414270%09red/5	Environmental Information Processing	Signaling molecules and interaction

						6822%09red/24750%09red/414788%09red/414819%09red		
rno05160	Hepatitis C	2.02028E-08	288774/297989/24329/25124/89829/286918/65190/78963/64625/192281/24835/494202/246268/54287/293624/363938/304507/25712/245920	19	Stat2/Ddx58/Egfr/Stat1/Socs3/Mx2/Rsad2/Apaf1/Bid/Oas1a/Tnf/Oas3/Oas1b/Eif2ak2/Irf7/Oas2/Oas1i/Ifng/Cxcl10	https://www.kegg.jp/kegg-bin/show_pathway?rno05160/288774%09red/297989%09red/24329%09red/25124%09red/89829%09red/286918%09red/65190%09red/78963%09red/64625%09red/192281%09red/24835%09red/494202%09red/246268%09red/54287%09red/293624%09red/363938%09red/304507%09red/25712%09red/245920%09red	Human Diseases	Infectious disease: viral
rno05145	Toxoplasmosis	2.0691E-08	25124/310553/252971/24599/25325/117029/85483/294273/78971/24835/29513/303163/316758/303090/117539/25712	16	Stat1/Tlr2/Socs1/Nos2/Il10/Ccr5/Ciita/RT1-DMb/Birc3/Tnf/Mapk13/Igtp/Lama1/Irgm/Il10ra/Ifng	https://www.kegg.jp/kegg-bin/show_pathway?rno05145/25124%09red/310553%09red/252971%09red/24599%09red/25325%09red/117029%09red/85483%09red/294273%09red/78971%09red/24835%09red/29513%09red/303163%09red/316758%09red/303090%09red/117539%09red/25712%09red	Human Diseases	Infectious disease: parasitic
rno05166	Human T-cell leukemia virus 1 infection	2.20131E-08	414779/294228/406194/414783/360323/24223/294273/24973/414792/414789/79	24	RT1-CE2/RT1-S3/RT1-T18/RT1-CE4/RT1-N2/B2m/RT1	https://www.kegg.jp/kegg-bin/show_pathway?rno05166/414779%09red/294228	Human Diseases	Infectious disease: viral

			426/25335/314638/24835/309603/368153/309607/24974/414270/114090/24750/414788/414819/25326		-DMb/RT1-A1/RT1-CE10/RT1-CE15/Zfp36/Mmp7/Creb3l3/Tnf/RT1-CE1/RT1-CE7/RT1-CE5/RT1-A2/RT1-CE14/Egr2/RT1-N3/RT1-T24-3/RT1-CE16/Jak3	%09red/406194%09red/414783%09red/360323%09red/24223%09red/294273%09red/24973%09red/414792%09red/414789%09red/79426%09red/25335%09red/314638%09red/24835%09red/309603%09red/368153%09red/309607%09red/24974%09red/414270%09red/114090%09red/24750%09red/414788%09red/414819%09red/25326%09red		
rno05165	Human papillomavirus infection	2.59435E-08	414779/288774/294228/24329/25124/406194/304545/304549/414783/298693/360323/286918/24973/24508/414792/414789/314638/24835/309603/368153/309607/54287/24974/414270/316758/293650/24750/414788/414819	29	RT1-CE2/Stat2/RT1-S3/Egfr/Stat1/RT1-T18/Oasl/Oasl2/RT1-CE4/Isg15/RT1-N2/Mx2/RT1-A1/Irf1/RT1-CE10/RT1-CE15/Creb3l3/Tnf/RT1-CE1/RT1-CE7/RT1-CE5/Eif2ak2/RT1-A2/RT1-CE14/Lama1/Tcirg1/RT1-N3/RT1-T24-3/RT1-CE16	https://www.kegg.jp/kegg-bin/show_pathway?rno05165/414779%09red/288774%09red/294228%09red/24329%09red/25124%09red/406194%09red/304545%09red/304549%09red/414783%09red/298693%09red/360323%09red/286918%09red/24973%09red/24508%09red/414792%09red/414789%09red/314638%09red/24835%09red/309603%09red/368153%09red/309607%09red/54287%09red/24974%09red/414270%09red/316758%09red/293650%09red/24750%09r	Human Diseases	Infectious disease: viral

						ed/414788%09red/414819%09red		
rno05323	Rheumatoid arthritis	3.3366E-08	310553/301289/24493/25542/25408/294273/24835/171551/60665/56822/24494/25712/293650/81503	14	Tlr2/Il17a/Il1a/Ccl3/Cd80/RT1-DMb/Tnf/Cxcl3/Cxcl6/Cd86/Il1b/Ifng/Tcirg1/Cxcl1	https://www.kegg.jp/kegg-bin/show_pathway?rno05323/310553%09red/301289%09red/24493%09red/25542%09red/25408%09red/294273%09red/24835%09red/171551%09red/60665%09red/56822%09red/24494%09red/25712%09red/293650%09red/81503%09red	Human Dis-eases	Immune disease
rno04657	IL-17 signal-ing pathway	8.07617E-08	301291/171052/25610/301289/24835/29513/171551/60665/81687/24494/25712/691516/245920/81503	14	Il17f/Mmp13/Csf3/Il17a/Tnf/Mapk13/Cxcl3/Cxcl6/Mmp9/Il1b/Ifng/Il17c/Cxcl10/Cxcl1	https://www.kegg.jp/kegg-bin/show_pathway?rno04657/301291%09red/171052%09red/25610%09red/301289%09red/24835%09red/29513%09red/171551%09red/60665%09red/81687%09red/24494%09red/25712%09red/691516%09red/245920%09red/81503%09red	Organismal Systems	Immune system
rno04620	Toll-like receptor signaling pathway	9.2926E-08	25124/310553/116637/29469/305354/25542/25408/24835/29513/293624/56822/24494/245920/246759	14	Stat1/Tlr2/Ccl4/Lbp/Tlr1/Ccl3/Cd80/Tnf/Mapk13/Irf7/Cd86/Il1b/Cxcl10/Cxcl9	https://www.kegg.jp/kegg-bin/show_pathway?rno04620/25124%09red/310553%09red/116637%09red/29469%09red/305354%09red/25542%09red/25408%09red/24835%09red/29513%09red/293624%09red/56822%09red/24494	Organismal Systems	Immune system

						%09red/245920%09red/246759%09red		
rno04630	JAK-STAT signaling pathway	1.1218E-07	500836/288774/24329/25124/252971/25610/89829/25325/298518/171081/81811/307200/365769/171333/289747/117539/25712/25326	18	Il22/Stat2/Egfr/Stat1/Socs1/Csf3/Socs3/Il10/Csf3r/Csf2rb/Thpo/Socs6/Il21/Il12rb1/Osm/Il10ra/Ifng/Jak3	https://www.kegg.jp/kegg-bin/show_pathway?rno04630/500836%09red/288774%09red/24329%09red/25124%09red/252971%09red/25610%09red/89829%09red/25325%09red/298518%09red/171081%09red/81811%09red/307200%09red/365769%09red/171333%09red/289747%09red/117539%09red/25712%09red/25326%09red	Environmental Information Processing	Signal transduction
rno04061	Viral protein interaction with cytokine and cytokine receptor	2.23655E-07	84475/116637/25325/117029/25542/89808/24835/171551/60665/117539/245920/81503/246759	13	Cxcr3/Ccl4/Il10/Ccr5/Ccl3/Cx3cl1/Tnf/Cxcl3/Cxcl6/Il10ra/Cxcl10/Cxcl1/Cxcl9	https://www.kegg.jp/kegg-bin/show_pathway?rno04061/84475%09red/116637%09red/25325%09red/117029%09red/25542%09red/89808%09red/24835%09red/171551%09red/60665%09red/117539%09red/245920%09red/81503%09red/246759%09red	Environmental Information Processing	Signaling molecules and interaction
rno05133	Pertussis	2.93501E-07	24232/24599/25325/24231/24493/24508/24835/29513/287362/60665/24494/292060	12	C3/Nos2/Il10/C2/Il1a/Irf1/Tnf/Mapk13/Nlrp3/Cxcl6/Il1b/Irf8	https://www.kegg.jp/kegg-bin/show_pathway?rno05133/24232%09red/24599%09red/25325%09red/24231%09red/24493%09red/24508%09red/24835%09red/29513%09red/	Human Diseases	Infectious disease: bacterial

						287362%09red/60665%09red/24494%09red/292060%09red		
rno04218	Cellular senescence	1.20406E-06	414779/294228/406194/414783/24493/360323/24973/414792/414789/309603/368153/29513/309607/24974/414270/24750/414788/414819	18	RT1-CE2/RT1-S3/RT1-T18/RT1-CE4/Il1a/RT1-N2/RT1-A1/RT1-CE10/RT1-CE15/RT1-CE1/RT1-CE7/Mapk13/RT1-CE5/RT1-A2/RT1-CE14/RT1-N3/RT1-T24-3/RT1-CE16	https://www.kegg.jp/kegg-bin/show_pathway?rno04218/414779%09red/294228%09red/406194%09red/414783%09red/24493%09red/360323%09red/24973%09red/414792%09red/414789%09red/309603%09red/368153%09red/29513%09red/309607%09red/24974%09red/414270%09red/24750%09red/414788%09red/414819%09red	Cellular Processes	Cell growth and death
rno05171	Coronavirus disease - COVID-19	2.08547E-06	156435/288774/297989/24329/25124/310553/25610/24232/298693/24231/81635/286918/192281/24835/29513/494202/246268/54287/287362/24494/363938/304507/499801/245920	24	Tmprss2/Stat2/Ddx58/Egfr/Stat1/Tlr2/Csf3/C3/Isg15/C2/Adar/Mx2/Oas1a/Tnf/Mapk13/Oas3/Oas1b/Eif2ak2/Nlrp3/Il1b/Oas2/Oas1i/Ifih1/Cxcl10	https://www.kegg.jp/kegg-bin/show_pathway?rno05171/156435%09red/288774%09red/297989%09red/24329%09red/25124%09red/310553%09red/25610%09red/24232%09red/298693%09red/24231%09red/81635%09red/286918%09red/192281%09red/24835%09red/29513%09red/494202%09red/246268%09red/54287%09red/287362%09red/24494%09red/363938%09red/304507%09red/499801%09red/245920	Human Diseases	Infectious disease: viral

						%09red		
rno04380	Osteoclast differentia-tion	2.50198E-06	288774/25124/252971/304966/89829/114243/24493/114553/24835/500904/29513/292594/24494/25712	14	Stat2/Stat1/Socs1/Fcgr3a/Socs3/Nox1/Il1a/Ncf1/Tnf/Ncf4/Mapk13/Lilrb4/Il1b/Ifng	https://www.kegg.jp/kegg-bin/show_pathway?rno04380/288774%09red/25124%09red/252971%09red/304966%09red/89829%09red/114243%09red/24493%09red/114553%09red/24835%09red/500904%09red/29513%09red/292594%09red/24494%09red/25712%09red	Organismal Systems	Development and regenera-tion
rno04623	Cytosolic DNA-sensing pathway	9.66411E-06	297989/116637/81635/171091/100049583/246240/293624/24494/245920	9	Ddx58/Ccl4/Adar/Zbp1/Trex1/Ripk3/Irf7/Il1b/Cxcl10	https://www.kegg.jp/kegg-bin/show_pathway?rno04623/297989%09red/116637%09red/81635%09red/171091%09red/100049583%09red/246240%09red/293624%09red/24494%09red/245920%09red	Organismal Systems	Immune system
rno05161	Hepatitis B	1.19509E-05	288774/297989/25124/310553/156117/78963/64625/314638/24835/29513/81687/293624/114090/499801/25326	15	Stat2/Ddx58/Stat1/Tlr2/Casp12/Apaf1/Bid/Creb3l3/Tnf/Mapk13/Mmp9/Irf7/Egr2/Ifih1/Jak3	https://www.kegg.jp/kegg-bin/show_pathway?rno05161/288774%09red/297989%09red/25124%09red/310553%09red/156117%09red/78963%09red/64625%09red/314638%09red/24835%09red/29513%09red/81687%09red/293624%09red/114090%09red/499801%09red/25326%09red	Human Dis-eases	Infectious disease: viral
rno04062	Chemokine	1.75549E-05	84475/28877	16	Cxcr3/Stat2/S	https://www.k	Organismal	Immune

	signaling pathway		4/25124/116637/304109/117029/25542/114553/89808/171551/497942/60665/245920/81503/25326/246759		tat1/Ccl4/Tiam1/Ccr5/Ccl3/Ncf1/Cx3cl1/Cxcl3/Cxcl16/Cxcl6/Cxcl10/Cxcl1/Jak3/Cxcl9	egg.jp/kegg-bin/show_pathway?rno04062/84475%09red/288774%09red/25124%09red/116637%09red/304109%09red/117029%09red/25542%09red/114553%09red/89808%09red/171551%09red/497942%09red/60665%09red/245920%09red/81503%09red/25326%09red/246759%09red	Systems	system
rno05417	Lipid and atherosclero-sis	2.64214E-05	310553/29469/114243/25542/114553/117058/78963/64625/24835/500904/29513/171551/287362/81687/293624/24494/81503	17	Tlr2/Lbp/Nox1/Ccl3/Ncf1/Pou2f2/Apaf1/Bid/Tnf/Ncf4/Mapk13/Cxcl3/Nlrp3/Mmp9/Irf7/Il1b/Cxcl1	https://www.kegg.jp/kegg-bin/show_pathway?rno05417/310553%09red/29469%09red/114243%09red/25542%09red/114553%09red/117058%09red/78963%09red/64625%09red/24835%09red/500904%09red/29513%09red/171551%09red/287362%09red/81687%09red/293624%09red/24494%09red/81503%09red	Human Dis-eases	Cardiovascu-lar disease
rno04625	C-type lectin receptor signaling pathway	3.17883E-05	288774/25124/25325/502902/24508/24835/29513/287362/24494/680611/114090/58936	12	Stat2/Stat1/Il10/Clec7a/Irf1/Tnf/Mapk13/Nlrp3/Il1b/Bcl3/Egr2/Plk3	https://www.kegg.jp/kegg-bin/show_pathway?rno04625/288774%09red/25124%09red/25325%09red/502902%09red/24508%09red/24835%09red/29513%09red/287362%09red/24494%09red/680611%	Organismal Systems	Immune system

						09red/114090%09red/58936%09red		
rno04659	Th17 cell differentia-tion	7.06353E-05	500836/301291/25124/301289/294273/29513/365769/171333/24494/25712/25326	11	Il22/Il17f/Stat1/Il17a/RT1-DMb/Mapk13/Il21/Il12rb1/Il1b/Ifng/Jak3	https://www.kegg.jp/kegg-bin/show_pathway?rno04659/500836%09red/301291%09red/25124%09red/301289%09red/294273%09red/29513%09red/365769%09red/171333%09red/24494%09red/25712%09red/25326%09red	Organismal Systems	Immune system
rno04144	Endocytosis	8.06845E-05	414779/294228/24329/406194/117029/414783/360323/24973/414792/414789/687208/309603/368153/309607/24974/414270/24750/414788/414819	19	RT1-CE2/RT1-S3/Egfr/RT1-T18/Ccr5/RT1-CE4/RT1-N2/RT1-A1/RT1-CE10/RT1-CE15/Vps37d/RT1-CE1/RT1-CE7/RT1-CE5/RT1-A2/RT1-CE14/RT1-N3/RT1-T24-3/RT1-CE16	https://www.kegg.jp/kegg-bin/show_pathway?rno04144/414779%09red/294228%09red/24329%09red/406194%09red/117029%09red/414783%09red/360323%09red/24973%09red/414792%09red/414789%09red/687208%09red/309603%09red/368153%09red/309607%09red/24974%09red/414270%09red/24750%09red/414788%09red/414819%09red	Cellular Processes	Transport and catabolism
rno04622	RIG-I-like receptor signaling pathway	0.000155566	297989/298693/24835/29513/293624/303538/499801/245920	8	Ddx58/Isg15/Tnf/Mapk13/Irf7/Dhx58/Ifih1/Cxcl10	https://www.kegg.jp/kegg-bin/show_pathway?rno04622/297989%09red/298693%09red/24835%09red/29513%09red/293624%09red/303538%09red/499801%09red/245920%	Organismal Systems	Immune system

						09red		
rno05146	Amoebiasis	0.000171449	24611/310553/24599/25325/24835/171551/316758/24494/25712/81503	10	Gnal/Tlr2/Nos2/Il10/Tnf/Cxcl3/Lama1/Il1b/Ifng/Cxcl1	https://www.kegg.jp/kegg-bin/show_pathway?rno05146/24611%09red/310553%09red/24599%09red/25325%09red/24835%09red/171551%09red/316758%09red/24494%09red/25712%09red/81503%09red	Human Diseases	Infectious disease: parasitic
rno04217	Necroptosis	0.000306866	288774/25124/24493/171091/64625/78971/24835/246240/54287/287362/24494/25712/25326	13	Stat2/Stat1/Il1a/Zbp1/Bid/Birc3/Tnf/Ripk3/Eif2ak2/Nlrp3/Il1b/Ifng/Jak3	https://www.kegg.jp/kegg-bin/show_pathway?rno04217/288774%09red/25124%09red/24493%09red/171091%09red/64625%09red/78971%09red/24835%09red/246240%09red/54287%09red/287362%09red/24494%09red/25712%09red/25326%09red	Cellular Processes	Cell growth and death
rno05142	Chagas disease	0.000331574	24611/310553/24232/24599/25325/25542/24835/29513/24494/25712	10	Gnal/Tlr2/C3/Nos2/Il10/Ccl3/Tnf/Mapk13/Il1b/Ifng	https://www.kegg.jp/kegg-bin/show_pathway?rno05142/24611%09red/310553%09red/24232%09red/24599%09red/25325%09red/25542%09red/24835%09red/29513%09red/24494%09red/25712%09red	Human Diseases	Infectious disease: parasitic
rno05144	Malaria	0.000485982	310553/25610/25325/24934/24835/24494/25712	7	Tlr2/Csf3/Il10/Klrk1/Tnf/Il1b/Ifng	https://www.kegg.jp/kegg-bin/show_pathway?rno05144/310553%09red/25610%09red/25325	Human Diseases	Infectious disease: parasitic

						%09red/24934%09red/24835%09red/24494%09red/25712%09red		
rno05134	Legionellosis	0.00067451	310553/24232/78963/24835/171551/24494/81503	7	Tlr2/C3/Apaf1/Tnf/Cxcl3/Il1b/Cxcl1	https://www.kegg.jp/kegg-bin/show_pathway?rno05134/310553%09red/24232%09red/78963%09red/24835%09red/171551%09red/24494%09red/81503%09red	Human Diseases	Infectious disease: bacterial
rno04930	Type II diabetes mellitus	0.001514446	252971/89829/25059/25060/24835/25467	6	Socs1/Socs3/Hk2/Hk3/Tnf/Irs1	https://www.kegg.jp/kegg-bin/show_pathway?rno04930/252971%09red/89829%09red/25059%09red/25060%09red/24835%09red/25467%09red	Human Diseases	Endocrine and metabolic disease
rno04658	Th1 and Th2 cell differentiation	0.002013465	25124/84010/294273/156726/29513/171333/25712/25326	8	Stat1/Dll1/RT1-DMb/Runx3/Mapk13/Il12rb1/Ifng/Jak3	https://www.kegg.jp/kegg-bin/show_pathway?rno04658/25124%09red/84010%09red/294273%09red/156726%09red/29513%09red/171333%09red/25712%09red/25326%09red	Organismal Systems	Immune system
rno05340	Primary immunodeficiency	0.002131617	24811/24812/85483/24165/25326	5	Tap1/Tap2/Ciita/Ada/Jak3	https://www.kegg.jp/kegg-bin/show_pathway?rno05340/24811%09red/24812%09red/85483%09red/24165%09red/25326%09red	Human Diseases	Immune disease
rno04640	Hematopoietic cell lineage	0.00216195	25259/25610/298518/24493/81811/294273/24835/24494	8	Gp5/Csf3/Csf3r/Il1a/Thpo/RT1-DMb/Tnf/Il1b	https://www.kegg.jp/kegg-bin/show_pathway?rno04640/25259%09red/25610%09red/298518	Organismal Systems	Immune system

						%09red/24493%09red/81811%09red/294273%09red/24835%09red/24494%09red		
rno05143	African trypanosomiasis	0.003423015	25325/66029/24835/24494/25712	5	Il10/Ido1/Tnf/Il1b/Ifng	https://www.kegg.jp/kegg-bin/show_pathway?rno05143/25325%09red/66029%09red/24835%09red/24494%09red/25712%09red	Human Diseases	Infectious disease: parasitic
rno05322	Systemic lupus erythematosus	0.00353952	24232/304966/25325/24231/25408/294273/24835/56822/25712	9	C3/Fcgr3a/Il10/C2/Cd80/RT1-DMb/Tnf/Cd86/Ifng	https://www.kegg.jp/kegg-bin/show_pathway?rno05322/24232%09red/304966%09red/25325%09red/24231%09red/25408%09red/294273%09red/24835%09red/56822%09red/25712%09red	Human Diseases	Immune disease
rno04064	NF-kappa B signaling pathway	0.003683463	297989/116637/29469/78971/24835/171551/24494/81503	8	Ddx58/Ccl4/Lbp/Birc3/Tnf/Cxcl3/Il1b/Cxcl1	https://www.kegg.jp/kegg-bin/show_pathway?rno04064/297989%09red/116637%09red/29469%09red/78971%09red/24835%09red/171551%09red/24494%09red/81503%09red	Environmental Information Processing	Signal transduction
rno05235	PD-L1 expression and PD-1 checkpoint pathway in cancer	0.007900236	24329/25124/310553/301626/299206/29513/25712	7	Egfr/Stat1/Tlr2/Pdcd1/Batf/Mapk13/Ifng	https://www.kegg.jp/kegg-bin/show_pathway?rno05235/24329%09red/25124%09red/310553%09red/301626%09red/299206%09red/29513%09red/25712%09red	Human Diseases	Cancer: overview

rno04936	Alcoholic liver disease	0.008038832	29469/24232/301289/24231/24835/29513/171551/24494/81503	9	Lbp/C3/Il17a/C2/Tnf/Mapk13/Cxcl3/Il1b/Cxcl1	https://www.kegg.jp/kegg-bin/show_pathway?rno04936/29469%09red/24232%09red/301289%09red/24231%09red/24835%09red/29513%09red/171551%09red/24494%09red/81503%09red	Human Diseases	Endocrine and metabolic disease
rno04215	Apoptosis - multiple species	0.010845413	492821/78963/64625/78971	4	Pmaip1/Apaf1/Bid/Birc3	https://www.kegg.jp/kegg-bin/show_pathway?rno04215/492821%09red/78963%09red/64625%09red/78971%09red	Cellular Processes	Cell growth and death
rno04918	Thyroid hormone synthesis	0.011072069	79107/499879/266807/314638/24213/84396	6	Duox2/Duoxa2/Duox1/Creb3l3/Atp1a3/Atp1b4	https://www.kegg.jp/kegg-bin/show_pathway?rno04918/79107%09red/499879%09red/266807%09red/314638%09red/24213%09red/84396%09red	Organismal Systems	Endocrine system
rno04917	Prolactin signaling pathway	0.01255551	25124/252971/89829/24508/307200/29513	6	Stat1/Socs1/Socs3/Irf1/Socs6/Mapk13	https://www.kegg.jp/kegg-bin/show_pathway?rno04917/25124%09red/252971%09red/89829%09red/24508%09red/307200%09red/29513%09red	Organismal Systems	Endocrine system
rno04960	Aldosterone-regulated sodium reabsorption	0.015940154	64190/24213/25467/84396	4	Fxyd4/Atp1a3/Irs1/Atp1b4	https://www.kegg.jp/kegg-bin/show_pathway?rno04960/64190%09red/24213%09red/25467%09red/84396%09red	Organismal Systems	Excretory system
rno04210	Apoptosis	0.024114497	171528/156117/171081/492821/78963/64625/78971/2	8	Gzmb/Casp12/Csf2rb/Pmaip1/Apaf1/Bi	https://www.kegg.jp/kegg-bin/show_pathway?rno042	Cellular Processes	Cell growth and death

			4835		d/Birc3/Tnf	10/171528%09red/156117%09red/171081%09red/492821%09red/78963%09red/64625%09red/78971%09red/24835%09red		
rno04672	Intestinal immune network for IgA production	0.024133858	25325/25408/294273/56822	4	Il10/Cd80/RT1-DMb/Cd86	https://www.kegg.jp/kegg-bin/show_pathway?rno04672/25325%09red/25408%09red/294273%09red/56822%09red	Organismal Systems	Immune system
rno04977	Vitamin digestion and absorption	0.025543142	316559/312226/303333	3	Slc19a3/RGD1565367/Slc46a1	https://www.kegg.jp/kegg-bin/show_pathway?rno04977/316559%09red/312226%09red/303333%09red	Organismal Systems	Digestive system
rno05310	Asthma	0.028339578	25325/294273/24835	3	Il10/RT1-DMb/Tnf	https://www.kegg.jp/kegg-bin/show_pathway?rno05310/25325%09red/294273%09red/24835%09red	Human Diseases	Immune disease
rno04973	Carbohydrate digestion and absorption	0.03007559	25059/25060/24213/84396	4	Hk2/Hk3/Atp1a3/Atp1b4	https://www.kegg.jp/kegg-bin/show_pathway?rno04973/25059%09red/25060%09red/24213%09red/84396%09red	Organismal Systems	Digestive system
rno03050	Proteasome	0.032231441	689852/24967/24968/25712	4	Psmf1/Psmb9/Psmb8/Ifng	https://www.kegg.jp/kegg-bin/show_pathway?rno03050/689852%09red/24967%09red/24968%09red/25712%09red	Genetic Information Processing	Folding, sorting and degradation
rno00601	Glycosphingolipid biosynthesis - lacto and neolacto	0.037659742	116740/363040/308586	3	B3gnt5/St3gal4/Sec1	https://www.kegg.jp/kegg-bin/show_pathway?rno00601/116740%0	Metabolism	Glycan biosynthesis and metabolism

	series					9red/363040%09red/308586%09red		
rno01523	Antifolate resistance	0.037659742	24835/303333/24494	3	Tnf/Slc46a1/Il1b	https://www.kegg.jp/kegg-bin/show_pathway?rno01523/24835%09red/303333%09red/24494%09red	Human Diseases	Drug resistance: antineoplastic
rno05418	Fluid shear stress and atherosclerosis	0.041189259	114243/24493/114553/24835/29513/81687/24494/25712	8	Nox1/Il1a/Ncf1/Tnf/Mapk13/Mmp9/Il1b/Ifng	https://www.kegg.jp/kegg-bin/show_pathway?rno05418/114243%09red/24493%09red/114553%09red/24835%09red/29513%09red/81687%09red/24494%09red/25712%09red	Human Diseases	Cardiovascular disease
rno04650	Natural killer cell mediated cytotoxicity	0.042262014	171528/304966/24934/64625/24835/25712	6	Gzmb/Fcgr3a/Klrk1/Bid/Tnf/Ifng	https://www.kegg.jp/kegg-bin/show_pathway?rno04650/171528%09red/304966%09red/24934%09red/64625%09red/24835%09red/25712%09red	Organismal Systems	Immune system
rno04933	AGE-RAGE signaling pathway in diabetic complications	0.0458529	25124/114243/24493/24835/29513/24494	6	Stat1/Nox1/Il1a/Tnf/Mapk13/Il1b	https://www.kegg.jp/kegg-bin/show_pathway?rno04933/25124%09red/114243%09red/24493%09red/24835%09red/29513%09red/24494%09red	Human Diseases	Endocrine and metabolic disease
rno04613	Neutrophil extracellular trap formation	0.049595264	310553/24232/304966/502902/114553/114555/500904/29513/315084	9	Tlr2/C3/Fcgr3a/Clec7a/Ncf1/Casp4/Ncf4/Mapk13/Gsdmd	https://www.kegg.jp/kegg-bin/show_pathway?rno04613/310553%09red/24232%09red/304966%09red/502902%09red/114553%09red/114555%09red/500904%09red/29513%0	Organismal Systems	Immune system

						9red/315084%09red		
rno04974	Protein digestion and absorption	0.049628448	294279/24213/301012/84396/117522/116726	6	Col11a2/Atp1a3/Col7a1/Atp1b4/Xpnpep2/Slc7a9	https://www.kegg.jp/kegg-bin/show_pathway?rno04974/294279%09red/24213%09red/301012%09red/84396%09red/117522%09red/116726%09red	Organismal Systems	Digestive system
rno04972	Pancreatic secretion	0.053590092	29354/362053/24213/84396/29692/25298	6	Pla2g5/Clca4/Atp1a3/Atp1b4/Pla2g2a/Cck	https://www.kegg.jp/kegg-bin/show_pathway?rno04972/29354%09red/362053%09red/24213%09red/84396%09red/29692%09red/25298%09red	Organismal Systems	Digestive system
rno04978	Mineral absorption	0.058670189	24249/303333/24213/84396	4	S100g/Slc46a1/Atp1a3/Atp1b4	https://www.kegg.jp/kegg-bin/show_pathway?rno04978/24249%09red/303333%09red/24213%09red/84396%09red	Organismal Systems	Digestive system
rno04610	Complement and coagulation cascades	0.070627621	24232/29243/50692/24231/304917	5	C3/F10/Plaur/C2/Serpinc1	https://www.kegg.jp/kegg-bin/show_pathway?rno04610/24232%09red/29243%09red/50692%09red/24231%09red/304917%09red	Organismal Systems	Immune system
rno04066	HIF-1 signaling pathway	0.073739977	24329/24599/25059/25060/25438/25712	6	Egfr/Nos2/Hk2/Hk3/Eno3/Ifng	https://www.kegg.jp/kegg-bin/show_pathway?rno04066/24329%09red/24599%09red/25059%09red/25060%09red/25438%09red/25712%09red	Environmental Information Processing	Signal transduction
rno03250	Viral life cycle - HIV-1	0.078730543	315137/117029/286918/311580	4	Apobec3/Ccr5/Mx2/Samhd1	https://www.kegg.jp/kegg-bin/show_pathway?rno032	Genetic Information Processing	Information processing in viruses

						50/315137%09red/117029%09red/286918%09red/311580%09red		
rno04935	Growth hormone synthesis, secretion and action	0.078734039	25124/252971/89829/314638/29513/25467	6	Stat1/Socs1/Socs3/Creb3l3/Mapk13/Irs1	https://www.kegg.jp/kegg-bin/show_pathway?rno04935/25124%09red/252971%09red/89829%09red/314638%09red/29513%09red/25467%09red	Organismal Systems	Endocrine system
rno05230	Central carbon metabolism in cancer	0.101758472	24329/80878/25059/25060	4	Egfr/Slc16a3/Hk2/Hk3	https://www.kegg.jp/kegg-bin/show_pathway?rno05230/24329%09red/80878%09red/25059%09red/25060%09red	Human Diseases	Cancer: overview
rno04964	Proximal tubule bicarbonate reclamation	0.108996489	24213/84396	2	Atp1a3/Atp1b4	https://www.kegg.jp/kegg-bin/show_pathway?rno04964/24213%09red/84396%09red	Organismal Systems	Excretory system
rno04926	Relaxin signaling pathway	0.112554425	24329/171052/24599/314638/29513/81687	6	Egfr/Mmp13/Nos2/Creb3l3/Mapk13/Mmp9	https://www.kegg.jp/kegg-bin/show_pathway?rno04926/24329%09red/171052%09red/24599%09red/314638%09red/29513%09red/81687%09red	Organismal Systems	Endocrine system
rno04920	Adipocytokine signaling pathway	0.114311822	89829/24835/25467/171410	4	Socs3/Tnf/Irs1/Acsbg1	https://www.kegg.jp/kegg-bin/show_pathway?rno04920/89829%09red/24835%09red/25467%09red/171410%09red	Organismal Systems	Endocrine system
rno05150	Staphylococcus aureus infection	0.115397411	24232/304966/25325/24231/294273	5	C3/Fcgr3a/Il10/C2/RT1-DMb	https://www.kegg.jp/kegg-bin/show_pathway?rno05150/24232%09red/304966%	Human Diseases	Infectious disease: bacterial

						09red/25325%09red/24231%09red/294273%09red		
rno05132	Salmonella infection	0.120319626	310553/29564/114555/78971/24835/246240/29513/287362/315084/24494	10	Tlr2/Dync1i1/Casp4/Birc3/Tnf/Ripk3/Mapk13/Nlrp3/Gsdmd/Il1b	https://www.kegg.jp/kegg-bin/show_pathway?rno05132/310553%09red/29564%09red/114555%09red/78971%09red/24835%09red/246240%09red/29513%09red/287362%09red/315084%09red/24494%09red	Human Diseases	Infectious disease: bacterial
rno04932	Non-alcoholic fatty liver disease	0.121889131	89829/24493/64625/24835/29513/25467/24494	7	Socs3/Il1a/Bid/Tnf/Mapk13/Irs1/Il1b	https://www.kegg.jp/kegg-bin/show_pathway?rno04932/89829%09red/24493%09red/64625%09red/24835%09red/29513%09red/25467%09red/24494%09red	Human Diseases	Endocrine and metabolic disease
rno00592	alpha-Linolenic acid metabolism	0.12602593	29354/29692	2	Pla2g5/Pla2g2a	https://www.kegg.jp/kegg-bin/show_pathway?rno00592/29354%09red/29692%09red	Metabolism	Lipid metabolism
rno04660	T cell receptor signaling pathway	0.130202401	301626/25325/24835/29513/25712	5	Pdcd1/Il10/Tnf/Mapk13/Ifng	https://www.kegg.jp/kegg-bin/show_pathway?rno04660/301626%09red/25325%09red/24835%09red/29513%09red/25712%09red	Organismal Systems	Immune system
rno04068	FoxO signaling pathway	0.1318414	24329/25325/29513/171497/25467/58936	6	Egfr/Il10/Mapk13/Sgk2/Irs1/Plk3	https://www.kegg.jp/kegg-bin/show_pathway?rno04068/24329%09red/25325%09red/29513%09red/171497%09red/25467%09red/589	Environmental Information Processing	Signal transduction

						36%09red		
rno04928	Parathyroid hormone synthesis, secretion and action	0.134033016	24329/171052/54264/314638/54278	5	Egfr/Mmp13/Mafb/Creb3l3/Nr4a2	https://www.kegg.jp/kegg-bin/show_pathway?rno04928/24329%09red/171052%09red/54264%09red/314638%09red/54278%09red	Organismal Systems	Endocrine system
rno05135	Yersinia infection	0.135201508	25325/24835/29513/287362/24494/58923	6	Il10/Tnf/Mapk13/Nlrp3/Il1b/Mefv	https://www.kegg.jp/kegg-bin/show_pathway?rno05135/25325%09red/24835%09red/29513%09red/287362%09red/24494%09red/58923%09red	Human Diseases	Infectious disease: bacterial
rno00330	Arginine and proline metabolism	0.143435595	308652/24599/114027	3	Smox/Nos2/Dao	https://www.kegg.jp/kegg-bin/show_pathway?rno00330/308652%09red/24599%09red/114027%09red	Metabolism	Amino acid metabolism
rno05205	Proteoglycans in cancer	0.165272859	24329/310553/304109/50692/24835/29513/81687/303991	8	Egfr/Tlr2/Tiam1/Plaur/Tnf/Mapk13/Mmp9/Hoxd10	https://www.kegg.jp/kegg-bin/show_pathway?rno05205/24329%09red/310553%09red/304109%09red/50692%09red/24835%09red/29513%09red/81687%09red/303991%09red	Human Diseases	Cancer: overview
rno01524	Platinum drug resistance	0.165558818	492821/78963/64625/78971	4	Pmaip1/Apaf1/Bid/Birc3	https://www.kegg.jp/kegg-bin/show_pathway?rno01524/492821%09red/78963%09red/64625%09red/78971%09red	Human Diseases	Drug resistance: antineoplastic
rno02010	ABC transporters	0.168177186	24811/24812/289797	3	Tap1/Tap2/Abca13	https://www.kegg.jp/kegg-bin/show_pathway?rno02010/24811%09	Environmental Information Processing	Membrane transport

						red/24812%09red/289797%09red		
rno04911	Insulin secretion	0.175649498	314638/24213/84396/25298	4	Creb3l3/Atp1a3/Atp1b4/Cck	https://www.kegg.jp/kegg-bin/show_pathway?rno04911/314638%09red/24213%09red/84396%09red/25298%09red	Organismal Systems	Endocrine system
rno04151	PI3K-Akt signaling pathway	0.179396877	24329/310553/59325/25610/298518/314638/171497/316758/289747/25467/29183/25326	12	Egfr/Tlr2/Ereg/Csf3/Csf3r/Creb3l3/Sgk2/Lama1/Osm/Irs1/Areg/Jak3	https://www.kegg.jp/kegg-bin/show_pathway?rno04151/24329%09red/310553%09red/59325%09red/25610%09red/298518%09red/314638%09red/171497%09red/316758%09red/289747%09red/25467%09red/29183%09red/25326%09red	Environmental Information Processing	Signal transduction
rno04710	Circadian rhythm	0.180176058	299691/29657	2	Cry1/Arntl	https://www.kegg.jp/kegg-bin/show_pathway?rno04710/299691%09red/29657%09red	Organismal Systems	Environmental adaptation
rno04261	Adrenergic signaling in cardiomyocytes	0.182361504	24837/314638/29248/29513/24213/84396	6	Tnnt2/Creb3l3/Tnni3/Mapk13/Atp1a3/Atp1b4	https://www.kegg.jp/kegg-bin/show_pathway?rno04261/24837%09red/314638%09red/29248%09red/29513%09red/24213%09red/84396%09red	Organismal Systems	Circulatory system
rno00410	beta-Alanine metabolism	0.189523626	81632/308652	2	Abat/Smox	https://www.kegg.jp/kegg-bin/show_pathway?rno00410/81632%09red/308652%09red	Metabolism	Metabolism of other amino acids
rno04260	Cardiac muscle contraction	0.191165419	24837/29248/24213/84396	4	Tnnt2/Tnni3/Atp1a3/Atp1b4	https://www.kegg.jp/kegg-bin/show_pat	Organismal Systems	Circulatory system

						hway?rno04260/24837%09red/29248%09red/24213%09red/84396%09red		
rno05210	Colorectal cancer	0.196431886	24329/59325/492821/29183	4	Egfr/Ereg/Pmaip1/Areg	https://www.kegg.jp/kegg-bin/show_pathway?rno05210/24329%09red/59325%09red/492821%09red/29183%09red	Human Diseases	Cancer: specific types
rno00052	Galactose metabolism	0.198935305	25059/25060	2	Hk2/Hk3	https://www.kegg.jp/kegg-bin/show_pathway?rno00052/25059%09red/25060%09red	Metabolism	Carbohydrate metabolism
rno00500	Starch and sucrose metabolism	0.198935305	25059/25060	2	Hk2/Hk3	https://www.kegg.jp/kegg-bin/show_pathway?rno00500/25059%09red/25060%09red	Metabolism	Carbohydrate metabolism
rno05410	Hypertrophic cardiomyopathy	0.207096282	24837/29248/24835/316758	4	Tnnt2/Tnni3/Tnf/Lama1	https://www.kegg.jp/kegg-bin/show_pathway?rno05410/24837%09red/29248%09red/24835%09red/316758%09red	Human Diseases	Cardiovascular disease
rno05222	Small cell lung cancer	0.212490776	24599/78963/78971/316758	4	Nos2/Apaf1/Birc3/Lama1	https://www.kegg.jp/kegg-bin/show_pathway?rno05222/24599%09red/78963%09red/78971%09red/316758%09red	Human Diseases	Cancer: specific types
rno01522	Endocrine resistance	0.223395777	24329/84010/29513/81687	4	Egfr/Dll1/Mapk13/Mmp9	https://www.kegg.jp/kegg-bin/show_pathway?rno01522/24329%09red/84010%09red/29513%09red/81687%09red	Human Diseases	Drug resistance: antineoplastic
rno04350	TGF-beta signaling	0.223395777	29373/117275/24835/2571	4	Bmp2/Chrd/T	https://www.kegg.jp/kegg-	Environmental Infor-	Signal trans-

	pathway		2		nf/Ifng	bin/show_pathway?rno04350/29373%09red/117275%09red/24835%09red/25712%09red	mation Processing	duction
rno05414	Dilated cardiomyopathy	0.223395777	24837/29248/24835/316758	4	Tnnt2/Tnni3/Tnf/Lama1	https://www.kegg.jp/kegg-bin/show_pathway?rno05414/24837%09red/29248%09red/24835%09red/316758%09red	Human Diseases	Cardiovascular disease
rno05202	Transcriptional misregulation in cancer	0.23371317	156435/171528/78971/79237/81687/56822/304005	7	Tmprss2/Gzmb/Birc3/Hhex/Mmp9/Cd86/Nfkbiz	https://www.kegg.jp/kegg-bin/show_pathway?rno05202/156435%09red/171528%09red/78971%09red/79237%09red/81687%09red/56822%09red/304005%09red	Human Diseases	Cancer: overview
rno04925	Aldosterone synthesis and secretion	0.234444058	314638/24213/84396/54278	4	Creb3l3/Atp1a3/Atp1b4/Nr4a2	https://www.kegg.jp/kegg-bin/show_pathway?rno04925/314638%09red/24213%09red/84396%09red/54278%09red	Organismal Systems	Endocrine system
rno00051	Fructose and mannose metabolism	0.237040642	25059/25060	2	Hk2/Hk3	https://www.kegg.jp/kegg-bin/show_pathway?rno00051/25059%09red/25060%09red	Metabolism	Carbohydrate metabolism
rno01250	Biosynthesis of nucleotide sugars	0.246639698	25059/25060	2	Hk2/Hk3	https://www.kegg.jp/kegg-bin/show_pathway?rno01250/25059%09red/25060%09red	Metabolism	Global and overview maps
rno05215	Prostate cancer	0.251255384	156435/24329/314638/81687	4	Tmprss2/Egfr/Creb3l3/Mmp9	https://www.kegg.jp/kegg-bin/show_pathway?rno05215/156435%09red/24329%	Human Diseases	Cancer: specific types

						09red/314638%09red/81687%09red		
rno00010	Glycolysis / Gluconeo-genesis	0.255232585	25059/25060/25438	3	Hk2/Hk3/Eno3	https://www.kegg.jp/kegg-bin/show_pathway?rno00010/25059%09red/25060%09red/25438%09red	Metabolism	Carbohydrate metabolism
rno05020	Prion disease	0.271266093	156117/24493/114553/78963/314638/24835/500904/29513/24494	9	Casp12/Il1a/Ncf1/Apaf1/Creb3l3/Tnf/Ncf4/Mapk13/Il1b	https://www.kegg.jp/kegg-bin/show_pathway?rno05020/156117%09red/24493%09red/114553%09red/78963%09red/314638%09red/24835%09red/500904%09red/29513%09red/24494%09red	Human Dis-eases	Neurodegen-erative dis-ease
rno00591	Linoleic acid metabolism	0.275491267	29354/29692	2	Pla2g5/Pla2g2a	https://www.kegg.jp/kegg-bin/show_pathway?rno00591/29354%09red/29692%09red	Metabolism	Lipid metabo-lism
rno05219	Bladder can-cer	0.285104278	24329/81687	2	Egfr/Mmp9	https://www.kegg.jp/kegg-bin/show_pathway?rno05219/24329%09red/81687%09red	Human Dis-eases	Cancer: spe-cific types
rno04910	Insulin sig-naling path-way	0.288009072	252971/89829/25059/25060/25467	5	Socs1/Socs3/Hk2/Hk3/Irs1	https://www.kegg.jp/kegg-bin/show_pathway?rno04910/252971%09red/89829%09red/25059%09red/25060%09red/25467%09red	Organismal Systems	Endocrine system
rno04115	p53 signaling pathway	0.290325989	492821/78963/64625	3	Pmaip1/Apaf1/Bid	https://www.kegg.jp/kegg-bin/show_pathway?rno04115/492821%09red/78963%09red/64625%09red	Cellular Processes	Cell growth and death

rno04962	Vasopressin-regulated water reab-sorption	0.313846285	29564/314638	2	Dync1i1/Creb3l3	https://www.kegg.jp/kegg-bin/show_pathway?rno04962/29564%09red/314638%09red	Organismal Systems	Excretory system
rno04975	Fat digestion and absorp-tion	0.313846285	29354/29692	2	Pla2g5/Pla2g2a	https://www.kegg.jp/kegg-bin/show_pathway?rno04975/29354%09red/29692%09red	Organismal Systems	Digestive system
rno04931	Insulin re-sistance	0.314670287	89829/314638/24835/25467	4	Socs3/Creb3l3/Tnf/Irs1	https://www.kegg.jp/kegg-bin/show_pathway?rno04931/89829%09red/314638%09red/24835%09red/25467%09red	Human Dis-eases	Endocrine and metabolic disease
rno04120	Ubiquitin mediated proteolysis	0.318310576	301000/252971/89829/295704/78971	5	Uba7/Socs1/Socs3/Ube2l6/Birc3	https://www.kegg.jp/kegg-bin/show_pathway?rno04120/301000%09red/252971%09red/89829%09red/295704%09red/78971%09red	Genetic In-formation Processing	Folding, sorting and degradation
rno04024	cAMP signal-ing pathway	0.326267178	304109/60448/314638/29248/24213/366270/84396	7	Tiam1/Htr1f/Creb3l3/Tnni3/Atp1a3/Edn3/Atp1b4	https://www.kegg.jp/kegg-bin/show_pathway?rno04024/304109%09red/60448%09red/314638%09red/29248%09red/24213%09red/366270%09red/84396%09red	Environmen-tal Infor-mation Pro-cessing	Signal trans-duction
rno00603	Glycosphin-golipid bio-synthesis - globo and isoglobo series	0.341538732	308586	1	Sec1	https://www.kegg.jp/kegg-bin/show_pathway?rno00603/308586%09red	Metabolism	Glycan bio-synthesis and metabolism
rno00565	Ether lipid metabolism	0.342331933	29354/29692	2	Pla2g5/Pla2g2a	https://www.kegg.jp/kegg-bin/show_pathway?rno00565/29354%09red/29692%09red	Metabolism	Lipid metabo-lism

rno05030	Cocaine addiction	0.342331933	314638/116671	2	Creb3l3/Cdk5r1	https://www.kegg.jp/kegg-bin/show_pathway?rno05030/314638%09red/116671%09red	Human Diseases	Substance dependence
rno04970	Salivary secretion	0.347013437	24213/84396/25211	3	Atp1a3/Atp1b4/Lyz2	https://www.kegg.jp/kegg-bin/show_pathway?rno04970/24213%09red/84396%09red/25211%09red	Organismal Systems	Digestive system
rno00590	Arachidonic acid metabolism	0.354092637	29354/298423/29692	3	Pla2g5/Cyp4a3/Pla2g2a	https://www.kegg.jp/kegg-bin/show_pathway?rno00590/29354%09red/298423%09red/29692%09red	Metabolism	Lipid metabolism
rno04670	Leukocyte transendothelial migration	0.355783998	114553/500904/29513/81687	4	Ncf1/Ncf4/Mapk13/Mmp9	https://www.kegg.jp/kegg-bin/show_pathway?rno04670/114553%09red/500904%09red/29513%09red/81687%09red	Organismal Systems	Immune system
rno00071	Fatty acid degradation	0.361117844	298423/171410	2	Cyp4a3/Acsbg1	https://www.kegg.jp/kegg-bin/show_pathway?rno00071/298423%09red/171410%09red	Metabolism	Lipid metabolism
rno00061	Fatty acid biosynthesis	0.375081598	171410	1	Acsbg1	https://www.kegg.jp/kegg-bin/show_pathway?rno00061/171410%09red	Metabolism	Lipid metabolism
rno04012	ErbB signaling pathway	0.375266669	24329/59325/29183	3	Egfr/Ereg/Areg	https://www.kegg.jp/kegg-bin/show_pathway?rno04012/24329%09red/59325%09red/29183%09red	Environmental Information Processing	Signal transduction
rno00520	Amino sugar and nucleotide sugar metabolism	0.379702559	25059/25060	2	Hk2/Hk3	https://www.kegg.jp/kegg-bin/show_pathway?rno005	Metabolism	Carbohydrate metabolism

						20/25059%09red/25060%09red		
rno04512	ECM-receptor interaction	0.389309132	25259/25592/316758	3	Gp5/Agrn/Lama1	https://www.kegg.jp/kegg-bin/show_pathway?rno04512/25259%09red/25592%09red/316758%09red	Environmental Information Processing	Signaling molecules and interaction
rno00220	Arginine biosynthesis	0.391209429	24599	1	Nos2	https://www.kegg.jp/kegg-bin/show_pathway?rno00220/24599%09red	Metabolism	Amino acid metabolism
rno05010	Alzheimer disease	0.426003272	156117/24599/114243/24493/78963/64625/24835/54287/25467/24494/116671	11	Casp12/Nos2/Nox1/Il1a/Apaf1/Bid/Tnf/Eif2ak2/Irs1/Il1b/Cdk5r1	https://www.kegg.jp/kegg-bin/show_pathway?rno05010/156117%09red/24599%09red/114243%09red/24493%09red/78963%09red/64625%09red/24835%09red/54287%09red/25467%09red/24494%09red/116671%09red	Human Diseases	Neurodegenerative disease
rno04976	Bile secretion	0.437759605	297386/24213/84396	3	Slc4a5/Atp1a3/Atp1b4	https://www.kegg.jp/kegg-bin/show_pathway?rno04976/297386%09red/24213%09red/84396%09red	Organismal Systems	Digestive system
rno04728	Dopaminergic synapse	0.443346243	24611/29657/314638/29513	4	Gnal/Arntl/Creb3l3/Mapk13	https://www.kegg.jp/kegg-bin/show_pathway?rno04728/24611%09red/29657%09red/314638%09red/29513%09red	Organismal Systems	Nervous system
rno04666	Fc gamma R-mediated phagocytosis	0.444570151	25603/304966/114553	3	Marcks/Fcgr3a/Ncf1	https://www.kegg.jp/kegg-bin/show_pathway?rno04666/25603%09red/304966%09red/114553	Organismal Systems	Immune system

						%09red		
rno04961	Endocrine and other factor-regulated calcium reab-sorption	0.451516448	24213/84396	2	Atp1a3/Atp1b4	https://www.kegg.jp/kegg-bin/show_pathway?rno04961/24213%09red/84396%09red	Organismal Systems	Excretory system
rno00534	Glycosa-minoglycan biosynthesis - heparan sul-fate / heparin	0.465839755	364476	1	Hs6st3	https://www.kegg.jp/kegg-bin/show_pathway?rno00534/364476%09red	Metabolism	Glycan bio-synthesis and metabolism
rno05415	Diabetic cardiomyopa-thy	0.47525829	114553/29248/500904/29513/25467/81687	6	Ncf1/Tnni3/Ncf4/Mapk13/Irs1/Mmp9	https://www.kegg.jp/kegg-bin/show_pathway?rno05415/114553%09red/29248%09red/500904%09red/29513%09red/25467%09red/81687%09red	Human Dis-eases	Cardiovascu-lar disease
rno04330	Notch signal-ing pathway	0.477226438	498089/84010	2	Dtx3l/Dll1	https://www.kegg.jp/kegg-bin/show_pathway?rno04330/498089%09red/84010%09red	Environmen-tal Infor-mation Pro-cessing	Signal trans-duction
rno04270	Vascular smooth mus-cle contrac-tion	0.483093386	29354/298423/366270/29692	4	Pla2g5/Cyp4a3/Edn3/Pla2g2a	https://www.kegg.jp/kegg-bin/show_pathway?rno04270/29354%09red/298423%09red/366270%09red/29692%09red	Organismal Systems	Circulatory system
rno04010	MAPK sig-naling path-way	0.484481277	24329/59325/24493/311406/24835/29513/24494/29183	8	Egfr/Ereg/Il1a/Dusp2/Tnf/Mapk13/Il1b/Areg	https://www.kegg.jp/kegg-bin/show_pathway?rno04010/24329%09red/59325%09red/24493%09red/311406%09red/24835%09red/29513%09red/24494%09red/29183%09red	Environmen-tal Infor-mation Pro-cessing	Signal trans-duction
rno04744	Phototrans-duction	0.49307469	365901	1	Gnat2	https://www.kegg.jp/kegg-bin/show_pathway?rno047	Organismal Systems	Sensory system

						44/365901%09red		
rno04950	Maturity onset diabetes of the young	0.506169122	79237	1	Hhex	https://www.kegg.jp/kegg-bin/show_pathway?rno04950/79237%09red	Human Diseases	Endocrine and metabolic disease
rno04966	Collecting duct acid secretion	0.506169122	293650	1	Tcirg1	https://www.kegg.jp/kegg-bin/show_pathway?rno04966/293650%09red	Organismal Systems	Excretory system
rno04664	Fc epsilon RI signaling pathway	0.510344032	24835/29513	2	Tnf/Mapk13	https://www.kegg.jp/kegg-bin/show_pathway?rno04664/24835%09red/29513%09red	Organismal Systems	Immune system
rno00650	Butanoate metabolism	0.518926744	81632	1	Abat	https://www.kegg.jp/kegg-bin/show_pathway?rno00650/81632%09red	Metabolism	Carbohydrate metabolism
rno00640	Propanoate metabolism	0.555263914	81632	1	Abat	https://www.kegg.jp/kegg-bin/show_pathway?rno00640/81632%09red	Metabolism	Carbohydrate metabolism
rno04520	Adherens junction	0.557383641	24329/498281	2	Egfr/Nectin4	https://www.kegg.jp/kegg-bin/show_pathway?rno04520/24329%09red/498281%09red	Cellular Processes	Cellular community - eukaryotes
rno04924	Renin secretion	0.557383641	362053/366270	2	Clca4/Edn3	https://www.kegg.jp/kegg-bin/show_pathway?rno04924/362053%09red/366270%09red	Organismal Systems	Endocrine system
rno05223	Non-small cell lung cancer	0.564906392	24329/25326	2	Egfr/Jak3	https://www.kegg.jp/kegg-bin/show_pathway?rno05223/24329%09red/25326%09red	Human Diseases	Cancer: specific types
rno04750	Inflammatory mediator regulation of	0.572470404	298423/29513/24494	3	Cyp4a3/Mapk13/Il1b	https://www.kegg.jp/kegg-bin/show_pat	Organismal Systems	Sensory system

	TRP channels					hway?rno04750/298423%09red/29513%09red/24494%09red		
rno05022	Pathways of neurodegeneration - multiple diseases	0.57643393	301000/156117/24599/114243/24493/78963/64625/295704/24835/29513/24494/116671	12	Uba7/Casp12/Nos2/Nox1/Il1a/Apaf1/Bid/Ube2l6/Tnf/Mapk13/Il1b/Cdk5r1	https://www.kegg.jp/kegg-bin/show_pathway?rno05022/301000%09red/156117%09red/24599%09red/114243%09red/24493%09red/78963%09red/64625%09red/295704%09red/24835%09red/29513%09red/24494%09red/116671%09red	Human Diseases	Neurodegenerative disease
rno04971	Gastric acid secretion	0.579676035	24213/84396	2	Atp1a3/Atp1b4	https://www.kegg.jp/kegg-bin/show_pathway?rno04971/24213%09red/84396%09red	Organismal Systems	Digestive system
rno05212	Pancreatic cancer	0.579676035	24329/25124	2	Egfr/Stat1	https://www.kegg.jp/kegg-bin/show_pathway?rno05212/24329%09red/25124%09red	Human Diseases	Cancer: specific types
rno01200	Carbon metabolism	0.595797379	25059/25060/25438	3	Hk2/Hk3/Eno3	https://www.kegg.jp/kegg-bin/show_pathway?rno01200/25059%09red/25060%09red/25438%09red	Metabolism	Global and overview maps
rno00250	Alanine, aspartate and glutamate metabolism	0.609852886	81632	1	Abat	https://www.kegg.jp/kegg-bin/show_pathway?rno00250/81632%09red	Metabolism	Amino acid metabolism
rno04071	Sphingolipid signaling pathway	0.623775206	64625/24835/29513	3	Bid/Tnf/Mapk13	https://www.kegg.jp/kegg-bin/show_pathway?rno04071/64625%09red/24835%09red/29513%	Environmental Information Processing	Signal transduction

						09red		
rno04919	Thyroid hormone signaling pathway	0.623775206	25124/24213/84396	3	Stat1/Atp1a3/Atp1b4	https://www.kegg.jp/kegg-bin/show_pathway?rno04919/25124%09red/24213%09red/84396%09red	Organismal Systems	Endocrine system
rno01232	Nucleotide metabolism	0.628458957	24165/314004	2	Ada/Cmpk2	https://www.kegg.jp/kegg-bin/show_pathway?rno01232/24165%09red/314004%09red	Metabolism	Global and overview maps
rno05206	MicroRNAs in cancer	0.630809089	24329/252971/25603/303477/25467/81687/303991	7	Egfr/Socs1/Marcks/Igf2bp1/Irs1/Mmp9/Hoxd10	https://www.kegg.jp/kegg-bin/show_pathway?rno05206/24329%09red/252971%09red/25603%09red/303477%09red/25467%09red/81687%09red/303991%09red	Human Diseases	Cancer: overview
rno00260	Glycine, serine and threonine metabolism	0.639347901	114027	1	Dao	https://www.kegg.jp/kegg-bin/show_pathway?rno00260/114027%09red	Metabolism	Amino acid metabolism
rno04022	cGMP-PKG signaling pathway	0.646832763	314638/24213/25467/84396	4	Creb3l3/Atp1a3/Irs1/Atp1b4	https://www.kegg.jp/kegg-bin/show_pathway?rno04022/314638%09red/24213%09red/25467%09red/84396%09red	Environmental Information Processing	Signal transduction
rno05033	Nicotine addiction	0.648677558	65187	1	Gabrq	https://www.kegg.jp/kegg-bin/show_pathway?rno05033/65187%09red	Human Diseases	Substance dependence
rno03320	PPAR signaling pathway	0.654309866	298423/171410	2	Cyp4a3/Acsbg1	https://www.kegg.jp/kegg-bin/show_pathway?rno03320/298423%09red/171410%09red	Organismal Systems	Endocrine system

rno04146	Peroxisome	0.660545178	24599/114027	2	Nos2/Dao	https://www.kegg.jp/kegg-bin/show_pathway?rno04146/24599%09red/114027%09red	Cellular Processes	Transport and catabolism
rno00230	Purine metabolism	0.660681185	81744/24165/191569	3	Pde7a/Ada/Pde9a	https://www.kegg.jp/kegg-bin/show_pathway?rno00230/81744%09red/24165%09red/191569%09red	Metabolism	Nucleotide metabolism
rno03410	Base excision repair	0.675249078	289662	1	Apex2l1	https://www.kegg.jp/kegg-bin/show_pathway?rno03410/289662%09red	Genetic Information Processing	Replication and repair
rno04142	Lysosome	0.675680125	316519/65161/293650	3	Slc11a1/Litaf/Tcirg1	https://www.kegg.jp/kegg-bin/show_pathway?rno04142/316519%09red/65161%09red/293650%09red	Cellular Processes	Transport and catabolism
rno04211	Longevity regulating pathway	0.678712296	314638/25467	2	Creb3l3/Irs1	https://www.kegg.jp/kegg-bin/show_pathway?rno04211/314638%09red/25467%09red	Organismal Systems	Aging
rno04912	GnRH signaling pathway	0.678712296	24329/29513	2	Egfr/Mapk13	https://www.kegg.jp/kegg-bin/show_pathway?rno04912/24329%09red/29513%09red	Organismal Systems	Endocrine system
rno04915	Estrogen signaling pathway	0.680570494	24329/314638/81687	3	Egfr/Creb3l3/Mmp9	https://www.kegg.jp/kegg-bin/show_pathway?rno04915/24329%09red/314638%09red/81687%09red	Organismal Systems	Endocrine system
rno04727	GABAergic synapse	0.684590126	81632/65187	2	Abat/Gabrq	https://www.kegg.jp/kegg-bin/show_pathway?rno04727/81632%09red/65187%0	Organismal Systems	Nervous system

						9red		
rno04742	Taste transduction	0.684590126	24417/60448	2	Grm4/Htr1f	https://www.kegg.jp/kegg-bin/show_pathway?rno04742/24417%09red/60448%09red	Organismal Systems	Sensory system
rno05032	Morphine addiction	0.690379964	81744/65187	2	Pde7a/Gabrq	https://www.kegg.jp/kegg-bin/show_pathway?rno05032/81744%09red/65187%09red	Human Diseases	Substance dependence
rno04360	Axon guidance	0.690424981	310630/362049/303342/315711	4	Sema4a/Unc5c/Ssh2/Sema7a	https://www.kegg.jp/kegg-bin/show_pathway?rno04360/310630%09red/362049%09red/303342%09red/315711%09red	Organismal Systems	Development and regeneration
rno00564	Glycerophospholipid metabolism	0.72330717	29354/29692	2	Pla2g5/Pla2g2a	https://www.kegg.jp/kegg-bin/show_pathway?rno00564/29354%09red/29692%09red	Metabolism	Lipid metabolism
rno00380	Tryptophan metabolism	0.729723196	66029	1	Ido1	https://www.kegg.jp/kegg-bin/show_pathway?rno00380/66029%09red	Metabolism	Amino acid metabolism
rno04723	Retrograde endocannabinoid signaling	0.751480266	65187/29513/296757	3	Gabrq/Mapk13/Napepld	https://www.kegg.jp/kegg-bin/show_pathway?rno04723/65187%09red/29513%09red/296757%09red	Organismal Systems	Nervous system
rno00240	Pyrimidine metabolism	0.756659981	314004	1	Cmpk2	https://www.kegg.jp/kegg-bin/show_pathway?rno00240/314004%09red	Metabolism	Nucleotide metabolism
rno00280	Valine, leucine and isoleucine degradation	0.769108412	81632	1	Abat	https://www.kegg.jp/kegg-bin/show_pathway?rno00280/81632%09red	Metabolism	Amino acid metabolism

rno04923	Regulation of lipolysis in adipocytes	0.769108412	25467	1	Irs1	https://www.kegg.jp/kegg-bin/show_pathway?rno04923/25467%09red	Organismal Systems	Endocrine system
rno04390	Hippo signaling pathway	0.77829621	29373/78971/29183	3	Bmp2/Birc3/Areg	https://www.kegg.jp/kegg-bin/show_pathway?rno04390/29373%09red/78971%09red/29183%09red	Environmental Information Processing	Signal transduction
rno04370	VEGF signaling pathway	0.780922651	29513	1	Mapk13	https://www.kegg.jp/kegg-bin/show_pathway?rno04370/29513%09red	Environmental Information Processing	Signal transduction
rno05213	Endometrial cancer	0.786602069	24329	1	Egfr	https://www.kegg.jp/kegg-bin/show_pathway?rno05213/24329%09red	Human Diseases	Cancer: specific types
rno01212	Fatty acid metabolism	0.797524839	171410	1	Acsbg1	https://www.kegg.jp/kegg-bin/show_pathway?rno01212/171410%09red	Metabolism	Global and overview maps
rno05217	Basal cell carcinoma	0.802775632	29373	1	Bmp2	https://www.kegg.jp/kegg-bin/show_pathway?rno05217/29373%09red	Human Diseases	Cancer: specific types
rno04141	Protein processing in endoplasmic reticulum	0.812450365	361384/156117/54287	3	Dnajb1/Casp12/Eif2ak2	https://www.kegg.jp/kegg-bin/show_pathway?rno04141/361384%09red/156117%09red/54287%09red	Genetic Information Processing	Folding, sorting and degradation
rno04213	Longevity regulating pathway - multiple species	0.812873929	25467	1	Irs1	https://www.kegg.jp/kegg-bin/show_pathway?rno04213/25467%09red	Organismal Systems	Aging
rno04722	Neurotrophin signaling pathway	0.81977537	29513/25467	2	Mapk13/Irs1	https://www.kegg.jp/kegg-bin/show_pathway?rno04722/29513%09	Organismal Systems	Nervous system

						red/25467%09red		
rno04810	Regulation of actin cyto-skeleton	0.831481174	64350/24329/304109/303342	4	Itgad/Egfr/Tiam1/Ssh2	https://www.kegg.jp/kegg-bin/show_pathway?rno04810/64350%09red/24329%09red/304109%09red/303342%09red	Cellular Processes	Cell motility
rno04927	Cortisol synthesis and secretion	0.831551908	314638	1	Creb3l3	https://www.kegg.jp/kegg-bin/show_pathway?rno04927/314638%09red	Organismal Systems	Endocrine system
rno05031	Amphetamine addiction	0.831551908	314638	1	Creb3l3	https://www.kegg.jp/kegg-bin/show_pathway?rno05031/314638%09red	Human Dis-eases	Substance dependence
rno04152	AMPK sig-naling path-way	0.833692266	314638/25467	2	Creb3l3/Irs1	https://www.kegg.jp/kegg-bin/show_pathway?rno04152/314638%09red/25467%09red	Environmen-tal Infor-mation Pro-cessing	Signal trans-duction
rno04611	Platelet acti-vation	0.84028211	25259/29513	2	Gp5/Mapk13	https://www.kegg.jp/kegg-bin/show_pathway?rno04611/25259%09red/29513%09red	Organismal Systems	Immune system
rno04014	Ras signaling pathway	0.851236072	29354/24329/304109/29692	4	Pla2g5/Egfr/Tiam1/Pla2g2a	https://www.kegg.jp/kegg-bin/show_pathway?rno04014/29354%09red/24329%09red/304109%09red/29692%09red	Environmen-tal Infor-mation Pro-cessing	Signal trans-duction
rno05218	Melanoma	0.852309873	24329	1	Egfr	https://www.kegg.jp/kegg-bin/show_pathway?rno05218/24329%09red	Human Dis-eases	Cancer: spe-cific types
rno04080	Neuroactive ligand-receptor interaction	0.855806104	24417/24232/25637/60448/65187/366270/25298	7	Grm4/C3/Ptger1/Htr1f/Gabrq/Edn3/Cck	https://www.kegg.jp/kegg-bin/show_pathway?rno04080/24417%09	Environmen-tal Infor-mation Pro-cessing	Signaling molecules and interac-tion

						red/24232%09red/25637%09red/60448%09red/65187%09red/366270%09red/25298%09red		
rno05214	Glioma	0.856145114	24329	1	Egfr	https://www.kegg.jp/kegg-bin/show_pathway?rno05214/24329%09red	Human Diseases	Cancer: specific types
rno05208	Chemical carcinogenesis - reactive oxygen species	0.858124662	24329/114243/114553/29513	4	Egfr/Nox1/Ncf1/Mapk13	https://www.kegg.jp/kegg-bin/show_pathway?rno05208/24329%09red/114243%09red/114553%09red/29513%09red	Human Diseases	Cancer: overview
rno04020	Calcium signaling pathway	0.866887631	24611/24329/24599/25637	4	Gnal/Egfr/Nos2/Ptger1	https://www.kegg.jp/kegg-bin/show_pathway?rno04020/24611%09red/24329%09red/24599%09red/25637%09red	Environmental Information Processing	Signal transduction
rno04662	B cell receptor signaling pathway	0.867065951	292594	1	Lilrb4	https://www.kegg.jp/kegg-bin/show_pathway?rno04662/292594%09red	Organismal Systems	Immune system
rno05412	Arrhythmogenic right ventricular cardiomyopathy	0.867065951	316758	1	Lama1	https://www.kegg.jp/kegg-bin/show_pathway?rno05412/316758%09red	Human Diseases	Cardiovascular disease
rno01230	Biosynthesis of amino acids	0.870519564	25438	1	Eno3	https://www.kegg.jp/kegg-bin/show_pathway?rno01230/25438%09red	Metabolism	Global and overview maps
rno04721	Synaptic vesicle cycle	0.870519564	293650	1	Tcirg1	https://www.kegg.jp/kegg-bin/show_pathway?rno04721/293650%09red	Organismal Systems	Nervous system
rno01521	EGFR tyrosine kinase	0.877161057	24329	1	Egfr	https://www.kegg.jp/kegg-	Human Diseases	Drug resistance:

	inhibitor resistance					bin/show_pathway?rno01521/24329%09red		antineoplastic
rno03018	RNA degradation	0.88346329	25438	1	Eno3	https://www.kegg.jp/kegg-bin/show_pathway?rno03018/25438%09red	Genetic Information Processing	Folding, sorting and degradation
rno04550	Signaling pathways regulating pluripotency of stem cells	0.885054892	29513/25326	2	Mapk13/Jak3	https://www.kegg.jp/kegg-bin/show_pathway?rno04550/29513%09red/25326%09red	Cellular Processes	Cellular community - eukaryotes
rno04510	Focal adhesion	0.893340974	24329/78971/316758	3	Egfr/Birc3/Lama1	https://www.kegg.jp/kegg-bin/show_pathway?rno04510/24329%09red/78971%09red/316758%09red	Cellular Processes	Cellular community - eukaryotes
rno00830	Retinol metabolism	0.895118137	298423	1	Cyp4a3	https://www.kegg.jp/kegg-bin/show_pathway?rno00830/298423%09red	Metabolism	Metabolism of cofactors and vitamins
rno04540	Gap junction	0.897845729	24329	1	Egfr	https://www.kegg.jp/kegg-bin/show_pathway?rno04540/24329%09red	Cellular Processes	Cellular community - eukaryotes
rno05224	Breast cancer	0.898630034	24329/84010	2	Egfr/Dll1	https://www.kegg.jp/kegg-bin/show_pathway?rno05224/24329%09red/84010%09red	Human Diseases	Cancer: specific types
rno04072	Phospholipase D signaling pathway	0.902814143	24417/24329	2	Grm4/Egfr	https://www.kegg.jp/kegg-bin/show_pathway?rno04072/24417%09red/24329%09red	Environmental Information Processing	Signal transduction
rno04914	Progesterone-mediated oocyte maturation	0.910459912	29513	1	Mapk13	https://www.kegg.jp/kegg-bin/show_pathway?rno04914/29513%09red	Organismal Systems	Endocrine system

rno01240	Biosynthesis of cofactors	0.910704768	66029/314004	2	Ido1/Cmpk2	https://www.kegg.jp/kegg-bin/show_pathway?rno01240/66029%09red/314004%09red	Metabolism	Global and overview maps
rno04921	Oxytocin signaling pathway	0.912581874	24329/294329	2	Egfr/Trpm2	https://www.kegg.jp/kegg-bin/show_pathway?rno04921/24329%09red/294329%09red	Organismal Systems	Endocrine system
rno04015	Rap1 signaling pathway	0.91612416	24329/304109/29513	3	Egfr/Tiam1/Mapk13	https://www.kegg.jp/kegg-bin/show_pathway?rno04015/24329%09red/304109%09red/29513%09red	Environmental Information Processing	Signal transduction
rno04150	mTOR signaling pathway	0.917994362	24835/25467	2	Tnf/Irs1	https://www.kegg.jp/kegg-bin/show_pathway?rno04150/24835%09red/25467%09red	Environmental Information Processing	Signal transduction
rno04934	Cushing syndrome	0.917994362	24329/314638	2	Egfr/Creb3l3	https://www.kegg.jp/kegg-bin/show_pathway?rno04934/24329%09red/314638%09red	Human Diseases	Endocrine and metabolic disease
rno05014	Amyotrophic lateral sclerosis	0.920107339	156117/24599/78963/64625/24835/29513	6	Casp12/Nos2/Apaf1/Bid/Tnf/Mapk13	https://www.kegg.jp/kegg-bin/show_pathway?rno05014/156117%09red/24599%09red/78963%09red/64625%09red/24835%09red/29513%09red	Human Diseases	Neurodegenerative disease
rno05012	Parkinson disease	0.921702868	301000/24611/78963/295704	4	Uba7/Gnal/Apaf1/Ube2l6	https://www.kegg.jp/kegg-bin/show_pathway?rno05012/301000%09red/24611%09red/78963%09red/295704%09red	Human Diseases	Neurodegenerative disease
rno05231	Choline me-	0.92356586	24329	1	Egfr	https://www.k	Human Dis-	Cancer: over-

	tabolism in cancer					egg.jp/kegg-bin/show_pathway?rno05231/24329%09red	eases	view
rno04916	Melanogene-sis	0.925556333	314638	1	Creb3l3	https://www.kegg.jp/kegg-bin/show_pathway?rno04916/314638%09red	Organismal Systems	Endocrine system
rno03015	mRNA sur-veillance pathway	0.927495189	368070	1	Pnn	https://www.kegg.jp/kegg-bin/show_pathway?rno03015/368070%09red	Genetic In-formation Processing	Translation
rno04530	Tight junction	0.932407241	304109/63994	2	Tiam1/Arhgap17	https://www.kegg.jp/kegg-bin/show_pathway?rno04530/304109%09red/63994%09red	Cellular Processes	Cellular community - eukaryotes
rno04922	Glucagon signaling pathway	0.934760606	314638	1	Creb3l3	https://www.kegg.jp/kegg-bin/show_pathway?rno04922/314638%09red	Organismal Systems	Endocrine system
rno05207	Chemical carcinogene-sis - receptor activation	0.936869059	24329/84010/314638	3	Egfr/Dll1/Creb3l3	https://www.kegg.jp/kegg-bin/show_pathway?rno05207/24329%09red/84010%09red/314638%09red	Human Dis-eases	Cancer: over-view
rno04725	Cholinergic synapse	0.948565462	314638	1	Creb3l3	https://www.kegg.jp/kegg-bin/show_pathway?rno04725/314638%09red	Organismal Systems	Nervous system
rno04724	Glutama-tergic synapse	0.949907182	24417	1	Grm4	https://www.kegg.jp/kegg-bin/show_pathway?rno04724/24417%09red	Organismal Systems	Nervous system
rno03013	Nucleocyto-plasmic transport	0.952486967	368070	1	Pnn	https://www.kegg.jp/kegg-bin/show_pathway?rno03013/368070%09red	Genetic In-formation Processing	Translation

rno04114	Oocyte meiosis	0.957256362	29513	1	Mapk13	https://www.kegg.jp/kegg-bin/show_pathway?rno04114/29513%09red	Cellular Processes	Cell growth and death
rno04726	Serotonergic synapse	0.960517772	60448	1	Htr1f	https://www.kegg.jp/kegg-bin/show_pathway?rno04726/60448%09red	Organismal Systems	Nervous system
rno04371	Apelin signaling pathway	0.97274884	24599	1	Nos2	https://www.kegg.jp/kegg-bin/show_pathway?rno04371/24599%09red	Environmental Information Processing	Signal transduction
rno00190	Oxidative phosphorylation	0.97549061	293650	1	Tcirg1	https://www.kegg.jp/kegg-bin/show_pathway?rno00190/293650%09red	Metabolism	Energy metabolism
rno04140	Autophagy - animal	0.980177255	25467	1	Irs1	https://www.kegg.jp/kegg-bin/show_pathway?rno04140/25467%09red	Cellular Processes	Transport and catabolism
rno05226	Gastric cancer	0.981202144	24329	1	Egfr	https://www.kegg.jp/kegg-bin/show_pathway?rno05226/24329%09red	Human Diseases	Cancer: specific types
rno04714	Thermogenesis	0.985899314	314638/29513	2	Creb3l3/Mapk13	https://www.kegg.jp/kegg-bin/show_pathway?rno04714/314638%09red/29513%09red	Organismal Systems	Environmental adaptation
rno04310	Wnt signaling pathway	0.987041019	25335	1	Mmp7	https://www.kegg.jp/kegg-bin/show_pathway?rno04310/25335%09red	Environmental Information Processing	Signal transduction
rno05034	Alcoholism	0.990583231	314638	1	Creb3l3	https://www.kegg.jp/kegg-bin/show_pathway?rno05034/314638%09red	Human Diseases	Substance dependence
rno05225	Hepatocellu-	0.99107164	24329	1	Egfr	https://www.k	Human Dis-	Cancer: spe-

	lar carcinoma					egg.jp/kegg-bin/show_pathway?rno05225/24329%09red	eases	cific types
rno05016	Huntington disease	0.997290655	78963/314638	2	Apaf1/Creb3l3	https://www.kegg.jp/kegg-bin/show_pathway?rno05016/78963%09red/314638%09red	Human Diseases	Neurodegenerative disease

ID is the channel number, Description stands for the description of the path, GeneID represents kegg id of all the enriched genes in the pathway, which is divided by "/", Count indicates the number of all enriched genes in the pathway, GeneSymbol represents the gene symbols of all genes enriched in the pathway, which are divided by "/". GeneSymbol and geneID correspond to each other in order from left to right, KEGGLink is the link address of KEGG path graph, Level_a indicates the annotation of KEGG path classification a, which is the reference of hierarchical relationship, Level_b indicates KEGG path grade b annotation.

Support Table 10. KEGG enrichment analysis of 426 DE mRNAs up-regulated by EA.

ID	Description	pvalue	GeneID	Count	GeneSymbol	KEGGLink	Level_a	Level_b
rno04080	Neuroactive ligand-receptor interaction	7.22615E-06	25354/170704/282839/24952/29191/282829/63887/24808/25107/25229/24536/25590/24677/24684/25033/25342/24889/29251/287730/25075/81638/24324/54305/79247/24179	25	Sstr5/Hrh4/Mc2r/Gcg/Tac3/Tac4/Nmu/Tacr3/Avpr1a/Chrm1/Lepr/Chrna4/Ppy/Prlr/Sstr1/Oxtr/Cck-ar/F2/Pyy/Htr1b/Agtr1b/Edn2/Sstr2/Htr5b/Agt	https://www.kegg.jp/kegg-bin/show_pathway?rno04080/25354%09red/170704%09red/282839%09red/24952%09red/29191%09red/282829%09red/63887%09red/24808%09red/25107%09red/25229%09red/24536%09red/25590%09red/24677%09red/24684%09red/25033%09red/25342%09red/24889%09red/29251%09red/287730%09red/25075%09red/81638%09red/24324%09red/54305%09red/79247%09red/24179%09red	Environmental Information Processing	Signaling molecules and interaction
rno03320	PPAR signaling pathway	9.0587E-06	81924/25629/25664/362282/25598/50549/24539/79451/246253/361602/24450	11	Cyp8b1/Plin1/Pparg/Pck1/Fabp2/Cyp4a1/Lpl/Fabp4/Adi-poq/Me3/Hmgcs2	https://www.kegg.jp/kegg-bin/show_pathway?rno03320/81924%09red/25629%09red/25664%	Organismal Systems	Endocrine system

						09red/362282%09red/25598%09red/50549%09red/24539%09red/79451%09red/246253%09red/361602%09red/24450%09red		
rno04614	Renin-angiotensin system	1.71821E-05	302668/24594/292868/24523/100360872/81638/24179	7	Ace2/Klk1b3/Klk1c9/Klk1/Mcpt1l1/Agtr1b/Agt	https://www.kegg.jp/kegg-bin/show_pathway?rno04614/302668%09red/24594%09red/292868%09red/24523%09red/100360872%09red/81638%09red/24179%09red	Organismal Systems	Endocrine system
rno00983	Drug metabolism - other enzymes	0.000138041	29225/171118/246252/64352/113902/24861/291863/24423/171341/24421	10	Ces1e/Ces2c/Ces2a/Gstm5/Ces1d/Ugt1a1/LOC291863/Gstm1/Mgst1/Gsta1	https://www.kegg.jp/kegg-bin/show_pathway?rno00983/29225%09red/171118%09red/246252%09red/64352%09red/113902%09red/24861%09red/291863%09red/24423%09red/171341%09red/24421%09red	Metabolism	Xenobiotics biodegradation and metabolism
rno00480	Glutathione metabolism	0.00037758	113892/64352/114020/24423/94167/171341/59300/24421	8	Nat8f3/Gstm5/Nat8f5/Gstm1/Prdx6/Mgst1/Nat8f1/Gsta1	https://www.kegg.jp/kegg-bin/show_pathway?rno00480/113892%09red/64352%09red/114020%09red/24423%09red/94167%09red/171341%09red/59300%09red/24421%09red	Metabolism	Metabolism of other amino acids
rno01230	Biosynthesis of amino acids	0.000716132	287877/24962/300981/497840/24250/64203/24651/24190	8	Pycr1/Cth/Acy1/Cps1/Cbs/Bcat2/Pklr/Aldob	https://www.kegg.jp/kegg-bin/show_pathway?rno01230/287877%09red/24962%	Metabolism	Global and overview maps

						09red/300981%09red/497840%09red/24250%09red/64203%09red/24651%09red/24190%09red		
rno04610	Complement and coagulation cascades	0.00136559	64023/24648/83580/60327/113936/29251/155012/54249	8	Masp1/Serpina1/Thbd/F13a1/Cpb2/F2/Cfh/Cfd	https://www.kegg.jp/kegg-bin/show_pathway?rno04610/64023%09red/24648%09red/83580%09red/60327%09red/113936%09red/29251%09red/155012%09red/54249%09red	Organismal Systems	Immune system
rno00010	Glycolysis / Gluconeogenesis	0.001503705	100145871/298079/24362/362282/25634/24651/24190	7	Adh5/Aldh1b1/Fbp1/Pck1/G6pc/Pklr/Aldob	https://www.kegg.jp/kegg-bin/show_pathway?rno00010/100145871%09red/298079%09red/24362%09red/362282%09red/25634%09red/24651%09red/24190%09red	Metabolism	Carbohydrate metabolism
rno00982	Drug metabolism - cytochrome P450	0.002274181	100145871/64352/24861/24423/493909/171341/24421	7	Adh5/Gstm5/Ugt1a1/Gstm1/Aox3/Mgst1/Gsta1	https://www.kegg.jp/kegg-bin/show_pathway?rno00982/100145871%09red/64352%09red/24861%09red/24423%09red/493909%09red/171341%09red/24421%09red	Metabolism	Xenobiotics biodegradation and metabolism
rno05144	Malaria	0.002420284	24440/287167/25717/361619/100134871/360504	6	Hbb/Hba-a1/Tgfb3/Hbb-b1/LOC100134871/Hba-a2	https://www.kegg.jp/kegg-bin/show_pathway?rno05144/24440%09red/287167%09red/25717%09red/361619%09red/100134871%09red/360504%0	Human Diseases	Infectious disease: parasitic

						9red		
rno00980	Metabolism of xenobiotics by cytochrome P450	0.002864451	100145871/25315/64352/24861/24423/171341/24421	7	Adh5/Ephx1/Gstm5/Ugt1a1/Gstm1/Mgst1/Gsta1	https://www.kegg.jp/kegg-bin/show_pathway?rno00980/100145871%09red/25315%09red/64352%09red/24861%09red/24423%09red/171341%09red/24421%09red	Metabolism	Xenobiotics biodegradation and metabolism
rno05204	Chemical carcinogenesis - DNA adducts	0.002864451	25315/64352/499353/24861/24423/171341/24421	7	Ephx1/Gstm5/Cyp2c24/Ugt1a1/Gstm1/Mgst1/Gsta1	https://www.kegg.jp/kegg-bin/show_pathway?rno05204/25315%09red/64352%09red/499353%09red/24861%09red/24423%09red/171341%09red/24421%09red	Human Diseases	Cancer: overview
rno05143	African trypanosomiasis	0.003054996	24440/287167/361619/100134871/360504	5	Hbb/Hba-a1/Hbb-b1/LOC100134871/Hba-a2	https://www.kegg.jp/kegg-bin/show_pathway?rno05143/24440%09red/287167%09red/361619%09red/100134871%09red/360504%09red	Human Diseases	Infectious disease: parasitic
rno04024	cAMP signaling pathway	0.003176132	25354/282839/24952/292794/302813/25229/25033/25342/58971/25075/24255/24324/54305	13	Sstr5/Mc2r/Gcg/Ffar2/Gpr119/Chrm1/Sstr1/Oxtr/Fxyd1/Htr1b/Cftr/Edn2/Sstr2	https://www.kegg.jp/kegg-bin/show_pathway?rno04024/25354%09red/282839%09red/24952%09red/292794%09red/302813%09red/25229%09red/25033%09red/25342%09red/58971%09red/25075%09red/24255%09red/24324%09red/54305%09red	Environmental Information Processing	Signal transduction
rno04726	Serotonergic synapse	0.003706324	286963/25053/685451/29713/499353/25	9	Cyp2d5/Cyp2d2/Gng13/Kcnj5/Cyp2c24/	https://www.kegg.jp/kegg-bin/show_pat	Organismal Systems	Nervous system

			693/25075/303252/79247		Slc18a1/Htr1b/Alox12e/Htr5b	hway?rno04726/286963%09red/25053%09red/685451%09red/29713%09red/499353%09red/25693%09red/25075%09red/303252%09red/79247%09red		
rno00533	Glycosa-minoglycan biosynthesis - keratan sul-fate	0.004619577	64445/307838/362924	3	St3gal3/Chst4/St3gal1	https://www.kegg.jp/kegg-bin/show_pathway?rno00533/64445%09red/307838%09red/362924%09red	Metabolism	Glycan bio-synthesis and metabolism
rno00620	Pyruvate metabolism	0.005636798	100145871/298079/362282/24651/361602	5	Adh5/Aldh1b1/Pck1/Pklr/Me3	https://www.kegg.jp/kegg-bin/show_pathway?rno00620/100145871%09red/298079%09red/362282%09red/24651%09red/361602%09red	Metabolism	Carbohydrate metabolism
rno00270	Cysteine and methionine metabolism	0.008037649	24962/25274/24250/64203/81718	5	Cth/Tst/Cbs/Bcat2/Cdo1	https://www.kegg.jp/kegg-bin/show_pathway?rno00270/24962%09red/25274%09red/24250%09red/64203%09red/81718%09red	Metabolism	Amino acid metabolism
rno00380	Tryptophan metabolism	0.008037649	29416/361272/298079/368066/493909	5	Aadat/Dhtkd1/Aldh1b1/Inmt/Aox3	https://www.kegg.jp/kegg-bin/show_pathway?rno00380/29416%09red/361272%09red/298079%09red/368066%09red/493909%09red	Metabolism	Amino acid metabolism
rno04976	Bile secretion	0.009191631	117274/25315/25240/170913/24861/29172/24255	7	Nr0b2/Ephx1/Aqp1/Abcb1a/Ugt1a1/Aqp8/Cftr	https://www.kegg.jp/kegg-bin/show_pathway?rno04976/117274%09red/25315%09red/25240%09red/1709	Organismal Systems	Digestive system

						13%09red/24861%09red/29172%09red/24255%09red		
rno01210	2-Oxocarboxylic acid metabolism	0.009618968	29416/300981/64203	3	Aadat/Acy1/Bcat2	https://www.kegg.jp/kegg-bin/show_pathway?rno01210/29416%09red/300981%09red/64203%09red	Metabolism	Global and overview maps
rno04152	AMPK signaling pathway	0.012633143	24362/25664/24536/362282/25634/246253/295052/24255	8	Fbp1/Pparg/Lepr/Pck1/G6pc/Adipoq/Ccna1/Cftr	https://www.kegg.jp/kegg-bin/show_pathway?rno04152/24362%09red/25664%09red/24536%09red/362282%09red/25634%09red/246253%09red/295052%09red/24255%09red	Environmental Information Processing	Signal transduction
rno00260	Glycine, serine and threonine metabolism	0.015948552	24962/290551/24250/81660	4	Cth/Chdh/Cbs/Gatm	https://www.kegg.jp/kegg-bin/show_pathway?rno00260/24962%09red/290551%09red/24250%09red/81660%09red	Metabolism	Amino acid metabolism
rno00590	Arachidonic acid metabolism	0.016757197	25527/499353/50549/114097/25526/303252	6	Ptgis/Cyp2c24/Cyp4a1/Ltc4s/Ptgds/Alox12e	https://www.kegg.jp/kegg-bin/show_pathway?rno00590/25527%09red/499353%09red/50549%09red/114097%09red/25526%09red/303252%09red	Metabolism	Lipid metabolism
rno00140	Steroid hormone biosynthesis	0.017697093	24950/286963/25053/79243/499353/24861	6	Srd5a1/Cyp2d5/Cyp2d2/Hsd17b2/Cyp2c24/Ugt1a1	https://www.kegg.jp/kegg-bin/show_pathway?rno00140/24950%09red/286963%09red/25053%09red/79243%09red/499353%09red/24861%09red	Metabolism	Lipid metabolism
rno04060	Cytokine-	0.017857995	365581/2885	13	Edar/Ccl24/P	https://www.k	Environmen-	Signaling

	cytokine receptor interaction		93/360918/25717/24536/117027/114103/680931/25644/24684/500598/29397/287034		f4/Tgfb3/Lepr/Ccr3/Il5ra/Bmp8a/Bmp6/Prlr/Tnfrsf18/Ccl11/Tnfrsf17	egg.jp/kegg-bin/show_pathway?rno04060/365581%09red/288593%09red/360918%09red/25717%09red/24536%09red/117027%09red/114103%09red/680931%09red/25644%09red/24684%09red/500598%09red/29397%09red/287034%09red	tal Information Processing	molecules and interaction
rno04270	Vascular smooth muscle contraction	0.023551353	58966/25107/50549/58965/81638/685867/24324/24179	8	Ramp2/Avpr1a/Cyp4a1/Ramp1/Agtr1b/Myl6/Edn2/Agt	https://www.kegg.jp/kegg-bin/show_pathway?rno04270/58966%09red/25107%09red/50549%09red/58965%09red/81638%09red/685867%09red/24324%09red/24179%09red	Organismal Systems	Circulatory system
rno05310	Asthma	0.026471827	29397/25047/25316	3	Ccl11/Fcer1a/Ms4a2	https://www.kegg.jp/kegg-bin/show_pathway?rno05310/29397%09red/25047%09red/25316%09red	Human Diseases	Immune disease
rno04950	Maturity onset diabetes of the young	0.029243579	29458/25100/24651	3	Neurod1/Foxa3/Pklr	https://www.kegg.jp/kegg-bin/show_pathway?rno04950/29458%09red/25100%09red/24651%09red	Human Diseases	Endocrine and metabolic disease
rno01200	Carbon metabolism	0.029903744	100145871/25106/24362/497840/24651/24190/361602	7	Adh5/Rgn/Fbp1/Cps1/Pklr/Aldob/Me3	https://www.kegg.jp/kegg-bin/show_pathway?rno01200/100145871%09red/25106%09red/24362%09red/497840%09red/24651%09red/24190%09r	Metabolism	Global and overview maps

						ed/361602%09red		
rno04924	Renin secretion	0.034194859	25240/308015/81638/24324/24179	5	Aqp1/Clca1/Agtr1b/Edn2/Agt	https://www.kegg.jp/kegg-bin/show_pathway?rno04924/25240%09red/308015%09red/81638%09red/24324%09red/24179%09red	Organismal Systems	Endocrine system
rno00601	Glycosphingolipid biosynthesis - lacto and neolacto series	0.035228454	84597/64445/366064	3	Fut9/St3gal3/B3galt1	https://www.kegg.jp/kegg-bin/show_pathway?rno00601/84597%09red/64445%09red/366064%09red	Metabolism	Glycan biosynthesis and metabolism
rno05226	Gastric cancer	0.036114042	64558/29499/25313/25717/363913/170913/116466/445583	8	Fzd4/Shh/Egf/Tgfb3/Fzd10/Abcb1a/Wnt2b/Reg4	https://www.kegg.jp/kegg-bin/show_pathway?rno05226/64558%09red/29499%09red/25313%09red/25717%09red/363913%09red/170913%09red/116466%09red/445583%09red	Human Diseases	Cancer: specific types
rno00030	Pentose phosphate pathway	0.038439478	25106/24362/24190	3	Rgn/Fbp1/Aldob	https://www.kegg.jp/kegg-bin/show_pathway?rno00030/25106%09red/24362%09red/24190%09red	Metabolism	Carbohydrate metabolism
rno00053	Ascorbate and aldarate metabolism	0.038439478	25106/298079/24861	3	Rgn/Aldh1b1/Ugt1a1	https://www.kegg.jp/kegg-bin/show_pathway?rno00053/25106%09red/298079%09red/24861%09red	Metabolism	Carbohydrate metabolism
rno00330	Arginine and proline metabolism	0.038465455	287877/298079/64313/81660	4	Pycr1/Aldh1b1/Oat/Gatm	https://www.kegg.jp/kegg-bin/show_pathway?rno00330/287877%09red/298079%09red/64313%09red/816	Metabolism	Amino acid metabolism

						60%09red		
rno00512	Mucin type O-glycan biosynthesis	0.041794431	292325/83627/362924	3	B3gnt6/Galnt5/St3gal1	https://www.kegg.jp/kegg-bin/show_pathway?rno00512/292325%09red/83627%09red/362924%09red	Metabolism	Glycan biosynthesis and metabolism
rno04974	Protein digestion and absorption	0.044632161	302668/314981/60372/113936/63883/664630	6	Ace2/Col14a1/Pga5/Cpb2/Kcne3/Slc6a19	https://www.kegg.jp/kegg-bin/show_pathway?rno04974/302668%09red/314981%09red/60372%09red/113936%09red/63883%09red/664630%09red	Organismal Systems	Digestive system
rno00280	Valine, leucine and isoleucine degradation	0.051389783	298079/493909/64203/24450	4	Aldh1b1/Aox3/Bcat2/Hmgcs2	https://www.kegg.jp/kegg-bin/show_pathway?rno00280/298079%09red/493909%09red/64203%09red/24450%09red	Metabolism	Amino acid metabolism
rno00040	Pentose and glucuronate interconversions	0.052705728	24788/290277/24861	3	Sord/Cryl1/Ugt1a1	https://www.kegg.jp/kegg-bin/show_pathway?rno00040/24788%09red/290277%09red/24861%09red	Metabolism	Carbohydrate metabolism
rno05202	Transcriptional misregulation in cancer	0.055298229	25410/299138/60357/100912108/25664/24484/295052/303828/25243	9	Cebpe/Six4/Prom1/Nupr1/Pparg/Igfbp3/Ccna1/Etv5/Arnt2	https://www.kegg.jp/kegg-bin/show_pathway?rno05202/25410%09red/299138%09red/60357%09red/100912108%09red/25664%09red/24484%09red/295052%09red/303828%09red/25243%09red	Human Diseases	Cancer: overview
rno00603	Glycosphingolipid biosynthesis - globo and isoglobo	0.059677818	84597/362924	2	Fut9/St3gal1	https://www.kegg.jp/kegg-bin/show_pathway?rno00603/84597%09	Metabolism	Glycan biosynthesis and metabolism

	series					red/362924%09red		
rno04961	Endocrine and other factor-regulated calcium reab-sorption	0.060150258	83839/24594/292868/24523	4	Calb1/Klk1b3/Klk1c9/Klk1	https://www.kegg.jp/kegg-bin/show_pathway?rno04961/83839%09red/24594%09red/292868%09red/24523%09red	Organismal Systems	Excretory system
rno00051	Fructose and mannose metabolism	0.06066497	24788/24362/24190	3	Sord/Fbp1/Aldob	https://www.kegg.jp/kegg-bin/show_pathway?rno00051/24788%09red/24362%09red/24190%09red	Metabolism	Carbohydrate metabolism
rno00830	Retinol me-tabolism	0.064576925	100145871/499353/50549/24861/493909	5	Adh5/Cyp2c24/Cyp4a1/Ugt1a1/Aox3	https://www.kegg.jp/kegg-bin/show_pathway?rno00830/100145871%09red/499353%09red/50549%09red/24861%09red/493909%09red	Metabolism	Metabolism of cofactors and vitamins
rno00450	Selenocom-pound me-tabolism	0.066546215	24962/368066	2	Cth/Inmt	https://www.kegg.jp/kegg-bin/show_pathway?rno00450/24962%09red/368066%09red	Metabolism	Metabolism of other ami-no acids
rno00350	Tyrosine metabolism	0.069150368	100145871/29383/493909	3	Adh5/Fah/Aox3	https://www.kegg.jp/kegg-bin/show_pathway?rno00350/100145871%09red/29383%09red/493909%09red	Metabolism	Amino acid metabolism
rno05217	Basal cell carcinoma	0.069652078	64558/29499/363913/116466	4	Fzd4/Shh/Fzd10/Wnt2b	https://www.kegg.jp/kegg-bin/show_pathway?rno05217/64558%09red/29499%09red/363913%09red/116466%09red	Human Dis-eases	Cancer: spe-cific types
rno00310	Lysine deg-radation	0.072981261	29416/361272/298079/300723	4	Aadat/Dhtkd1/Aldh1b1/Hykk	https://www.kegg.jp/kegg-bin/show_pathway?rno003	Metabolism	Amino acid metabolism

						10/29416%09red/361272%09red/298079%09red/300723%09red		
rno00220	Arginine biosynthesis	0.081008924	300981/497840	2	Acy1/Cps1	https://www.kegg.jp/kegg-bin/show_pathway?rno00220/300981%09red/497840%09red	Metabolism	Amino acid metabolism
rno05225	Hepatocellular carcinoma	0.081077063	64558/25717/64352/363913/116466/24423/171341/24421	8	Fzd4/Tgfb3/Gstm5/Fzd10/Wnt2b/Gstm1/Mgst1/Gsta1	https://www.kegg.jp/kegg-bin/show_pathway?rno05225/64558%09red/25717%09red/64352%09red/363913%09red/116466%09red/24423%09red/171341%09red/24421%09red	Human Diseases	Cancer: specific types
rno04072	Phospholipase D signaling pathway	0.083090811	25313/25107/29251/25047/81638/25316/24179	7	Egf/Avpr1a/F2/Fcer1a/Agtr1b/Ms4a2/Agt	https://www.kegg.jp/kegg-bin/show_pathway?rno04072/25313%09red/25107%09red/29251%09red/25047%09red/81638%09red/25316%09red/24179%09red	Environmental Information Processing	Signal transduction
rno04350	TGF-beta signaling pathway	0.087043782	29139/289264/25717/680931/25644	5	Dcn/Grem2/Tgfb3/Bmp8a/Bmp6	https://www.kegg.jp/kegg-bin/show_pathway?rno04350/29139%09red/289264%09red/25717%09red/680931%09red/25644%09red	Environmental Information Processing	Signal transduction
rno04925	Aldosterone synthesis and secretion	0.09322566	282839/29713/81743/81638/24179	5	Mc2r/Kcnj5/Pde2a/Agtr1b/Agt	https://www.kegg.jp/kegg-bin/show_pathway?rno04925/282839%09red/29713%09red/81743%09red/81638%09red/24179%09red	Organismal Systems	Endocrine system

rno00770	Pantothenate and CoA biosynthesis	0.096332105	298079/64203	2	Aldh1b1/Bcat2	https://www.kegg.jp/kegg-bin/show_pathway?rno00770/298079%09red/64203%09red	Metabolism	Metabolism of cofactors and vitamins
rno04975	Fat digestion and absorption	0.097544535	54225/25598/432367	3	Apob/Fabp2/Npc1l1	https://www.kegg.jp/kegg-bin/show_pathway?rno04975/54225%09red/25598%09red/432367%09red	Organismal Systems	Digestive system
rno04390	Hippo signaling pathway	0.102353612	64558/305251/25717/363913/116466/680931/25644	7	Fzd4/Rassf6/Tgfb3/Fzd10/Wnt2b/Bmp8a/Bmp6	https://www.kegg.jp/kegg-bin/show_pathway?rno04390/64558%09red/305251%09red/25717%09red/363913%09red/116466%09red/680931%09red/25644%09red	Environmental Information Processing	Signal transduction
rno04934	Cushing syndrome	0.102353612	64558/282839/64455/363913/116466/81638/24179	7	Fzd4/Mc2r/Rasd1/Fzd10/Wnt2b/Agtr1b/Agt	https://www.kegg.jp/kegg-bin/show_pathway?rno04934/64558%09red/282839%09red/64455%09red/363913%09red/116466%09red/81638%09red/24179%09red	Human Diseases	Endocrine and metabolic disease
rno04964	Proximal tubule bicarbonate reclamation	0.104278525	25240/362282	2	Aqp1/Pck1	https://www.kegg.jp/kegg-bin/show_pathway?rno04964/25240%09red/362282%09red	Organismal Systems	Excretory system
rno04920	Adipocytokine signaling pathway	0.106421385	24536/362282/25634/246253	4	Lepr/Pck1/G6pc/Adipoq	https://www.kegg.jp/kegg-bin/show_pathway?rno04920/24536%09red/362282%09red/25634%09red/246253%09red	Organismal Systems	Endocrine system
rno00515	Mannose type	0.112395879	84597/64445	2	Fut9/St3gal3	https://www.k	Metabolism	Glycan bio-

	O-glycan biosynthesis					egg.jp/kegg-bin/show_pathway?rno00515/84597%09red/64445%09red		synthesis and metabolism
rno05207	Chemical carcinogenesis - receptor activation	0.119672134	317203/25315/25313/64352/25590/24861/24423/171341/24421	9	Rps6ka6/Ephx1/Egf/Gstm5/Chrna4/Ugt1a1/Gstm1/Mgst1/Gsta1	https://www.kegg.jp/kegg-bin/show_pathway?rno05207/317203%09red/25315%09red/25313%09red/64352%09red/25590%09red/24861%09red/24423%09red/171341%09red/24421%09red	Human Diseases	Cancer: overview
rno04310	Wnt signaling pathway	0.121037138	89803/64558/84402/287526/363913/295445/116466	7	Sfrp4/Fzd4/Sfrp1/Serpinf1/Fzd10/Dkk2/Wnt2b	https://www.kegg.jp/kegg-bin/show_pathway?rno04310/89803%09red/64558%09red/84402%09red/287526%09red/363913%09red/295445%09red/116466%09red	Environmental Information Processing	Signal transduction
rno04972	Pancreatic secretion	0.123700446	84590/308015/113936/24889/24255	5	Rab27b/Clca1/Cpb2/Cckar/Cftr	https://www.kegg.jp/kegg-bin/show_pathway?rno04972/84590%09red/308015%09red/113936%09red/24889%09red/24255%09red	Organismal Systems	Digestive system
rno00071	Fatty acid degradation	0.124209766	100145871/298079/50549	3	Adh5/Aldh1b1/Cyp4a1	https://www.kegg.jp/kegg-bin/show_pathway?rno00071/100145871%09red/298079%09red/50549%09red	Metabolism	Lipid metabolism
rno04977	Vitamin digestion and absorption	0.129090039	54225/24710	2	Apob/Rbp2	https://www.kegg.jp/kegg-bin/show_pathway?rno04977/54225%09red/24710%09red	Organismal Systems	Digestive system

rno04020	Calcium signaling pathway	0.14417841	25313/24808/59299/25107/25229/25342/24889/81638/79247	9	Egf/Tacr3/Trdn/Avpr1a/Chrm1/Oxtr/Cckar/Agtr1b/Htr5b	https://www.kegg.jp/kegg-bin/show_pathway?rno04020/25313%09red/24808%09red/59299%09red/25107%09red/25229%09red/25342%09red/24889%09red/81638%09red/79247%09red	Environmental Information Processing	Signal transduction
rno01524	Platinum drug resistance	0.154920597	64352/24423/171341/24421	4	Gstm5/Gstm1/Mgst1/Gsta1	https://www.kegg.jp/kegg-bin/show_pathway?rno01524/64352%09red/24423%09red/171341%09red/24421%09red	Human Diseases	Drug resistance: antineoplastic
rno00650	Butanoate metabolism	0.155094212	361637/24450	2	Acsm5/Hmgcs2	https://www.kegg.jp/kegg-bin/show_pathway?rno00650/361637%09red/24450%09red	Metabolism	Carbohydrate metabolism
rno04061	Viral protein interaction with cytokine and cytokine receptor	0.164512587	288593/360918/117027/29397	4	Ccl24/Pf4/Ccr3/Ccl11	https://www.kegg.jp/kegg-bin/show_pathway?rno04061/288593%09red/360918%09red/117027%09red/29397%09red	Environmental Information Processing	Signaling molecules and interaction
rno00561	Glycerolipid metabolism	0.209754187	298079/362972/24539	3	Aldh1b1/Pnpla3/Lpl	https://www.kegg.jp/kegg-bin/show_pathway?rno00561/298079%09red/362972%09red/24539%09red	Metabolism	Lipid metabolism
rno00750	Vitamin B6 metabolism	0.224439485	493909	1	Aox3	https://www.kegg.jp/kegg-bin/show_pathway?rno00750/493909%09red	Metabolism	Metabolism of cofactors and vitamins
rno00920	Sulfur metabolism	0.224439485	25274	1	Tst	https://www.kegg.jp/kegg-bin/show_pathway?rno00920/25274%09	Metabolism	Energy metabolism

						red		
rno00250	Alanine, aspartate and glutamate metabolism	0.228056652	497840/289727	2	Cps1/Nat8l	https://www.kegg.jp/kegg-bin/show_pathway?rno00250/497840%09red/289727%09red	Metabolism	Amino acid metabolism
rno05150	Staphylococcus aureus infection	0.242228838	64023/155012/294853/54249	4	Masp1/Cfh/Krt18/Cfd	https://www.kegg.jp/kegg-bin/show_pathway?rno05150/64023%09red/155012%09red/294853%09red/54249%09red	Human Diseases	Infectious disease: bacterial
rno04927	Cortisol synthesis and secretion	0.242946598	282839/81638/24179	3	Mc2r/Agtr1b/Agt	https://www.kegg.jp/kegg-bin/show_pathway?rno04927/282839%09red/81638%09red/24179%09red	Organismal Systems	Endocrine system
rno04933	AGE-RAGE signaling pathway in diabetic complications	0.247696733	25717/83580/81638/24179	4	Tgfb3/Thbd/Agtr1b/Agt	https://www.kegg.jp/kegg-bin/show_pathway?rno04933/25717%09red/83580%09red/81638%09red/24179%09red	Human Diseases	Endocrine and metabolic disease
rno05205	Proteoglycans in cancer	0.26337666	29139/64558/29499/363913/116466/81682/25236	7	Dcn/Fzd4/Shh/Fzd10/Wnt2b/Lum/Gpc3	https://www.kegg.jp/kegg-bin/show_pathway?rno05205/29139%09red/64558%09red/29499%09red/363913%09red/116466%09red/81682%09red/25236%09red	Human Diseases	Cancer: overview
rno04922	Glucagon signaling pathway	0.264256059	24952/24362/362282/25634	4	Gcg/Fbp1/Pck1/G6pc	https://www.kegg.jp/kegg-bin/show_pathway?rno04922/24952%09red/24362%09red/362282%09red/25634%09red	Organismal Systems	Endocrine system
rno00513	Various types of N-glycan	0.265454294	291770/64445	2	Chst9/St3gal3	https://www.kegg.jp/kegg-bin/show_pat	Metabolism	Glycan biosynthesis and

	biosynthesis					hway?rno00513/291770%09red/64445%09red		metabolism
rno04216	Ferroptosis	0.28418057	295455/24825	2	Slc39a8/Tf	https://www.kegg.jp/kegg-bin/show_pathway?rno04216/295455%09red/24825%09red	Cellular Processes	Cell growth and death
rno04550	Signaling pathways regulating pluripotency of stem cells	0.284498169	64558/299210/363913/116466/64444	5	Fzd4/Esrrb/Fzd10/Wnt2b/Isl1	https://www.kegg.jp/kegg-bin/show_pathway?rno04550/64558%09red/299210%09red/363913%09red/116466%09red/64444%09red	Cellular Processes	Cellular community - eukaryotes
rno04971	Gastric acid secretion	0.290641458	297910/24255/54305	3	Slc26a7/Cftr/Sstr2	https://www.kegg.jp/kegg-bin/show_pathway?rno04971/297910%09red/24255%09red/54305%09red	Organismal Systems	Digestive system
rno00514	Other types of O-glycan biosynthesis	0.293529703	83627/64445	2	Galnt5/St3gal3	https://www.kegg.jp/kegg-bin/show_pathway?rno00514/83627%09red/64445%09red	Metabolism	Glycan biosynthesis and metabolism
rno04931	Insulin resistance	0.297938812	317203/362282/25634/24179	4	Rps6ka6/Pck1/G6pc/Agt	https://www.kegg.jp/kegg-bin/show_pathway?rno04931/317203%09red/362282%09red/25634%09red/24179%09red	Human Diseases	Endocrine and metabolic disease
rno00604	Glycosphingolipid biosynthesis - ganglio series	0.31706947	362924	1	St3gal1	https://www.kegg.jp/kegg-bin/show_pathway?rno00604/362924%09red	Metabolism	Glycan biosynthesis and metabolism
rno05418	Fluid shear stress and atherosclerosis	0.328820544	83580/64352/24423/171341/24421	5	Thbd/Gstm5/Gstm1/Mgst1/Gsta1	https://www.kegg.jp/kegg-bin/show_pathway?rno05418/83580%09red/64352%09red/24423%	Human Diseases	Cardiovascular disease

						09red/171341%09red/24421%09red		
rno00565	Ether lipid metabolism	0.330712105	50555/683713	2	Ugt8/Gal3st1	https://www.kegg.jp/kegg-bin/show_pathway?rno00565/50555%09red/683713%09red	Metabolism	Lipid metabolism
rno04935	Growth hormone synthesis, secretion and action	0.332110878	25354/25033/24484/54305	4	Sstr5/Sstr1/Igfbp3/Sstr2	https://www.kegg.jp/kegg-bin/show_pathway?rno04935/25354%09red/25033%09red/24484%09red/54305%09red	Organismal Systems	Endocrine system
rno04930	Type II diabetes mellitus	0.349110527	24651/246253	2	Pklr/Adipoq	https://www.kegg.jp/kegg-bin/show_pathway?rno04930/24651%09red/246253%09red	Human Diseases	Endocrine and metabolic disease
rno04979	Cholesterol metabolism	0.349110527	54225/24539	2	Apob/Lpl	https://www.kegg.jp/kegg-bin/show_pathway?rno04979/54225%09red/24539%09red	Organismal Systems	Digestive system
rno00910	Nitrogen metabolism	0.350959082	497840	1	Cps1	https://www.kegg.jp/kegg-bin/show_pathway?rno00910/497840%09red	Metabolism	Energy metabolism
rno04911	Insulin secretion	0.359619291	24952/302813/24889	3	Gcg/Gpr119/Cckar	https://www.kegg.jp/kegg-bin/show_pathway?rno04911/24952%09red/302813%09red/24889%09red	Organismal Systems	Endocrine system
rno04630	JAK-STAT signaling pathway	0.363770647	25313/24536/114103/24684/493909	5	Egf/Lepr/Il5ra/Prlr/Aox3	https://www.kegg.jp/kegg-bin/show_pathway?rno04630/25313%09red/24536%09red/114103%09red/24684%09red/493909%09red	Environmental Information Processing	Signal transduction

rno00120	Primary bile acid biosynthesis	0.367270668	81924	1	Cyp8b1	https://www.kegg.jp/kegg-bin/show_pathway?rno00120/81924%09red	Metabolism	Lipid metabolism
rno04932	Non-alcoholic fatty liver disease	0.378793431	25664/24536/289754/24651/246253	5	Pparg/Lepr/Xbp1/Pklr/Adipoq	https://www.kegg.jp/kegg-bin/show_pathway?rno04932/25664%09red/24536%09red/289754%09red/24651%09red/246253%09red	Human Diseases	Endocrine and metabolic disease
rno05010	Alzheimer disease	0.391978012	291287/295455/64558/29219/25229/363913/289754/295445/116466/24539/288227	11	Tubal3/Slc39a8/Fzd4/Snca/Chrm1/Fzd10/Xbp1/Dkk2/Wnt2b/Lpl/Bace2	https://www.kegg.jp/kegg-bin/show_pathway?rno05010/291287%09red/295455%09red/64558%09red/29219%09red/25229%09red/363913%09red/289754%09red/295445%09red/116466%09red/24539%09red/288227%09red	Human Diseases	Neurodegenerative disease
rno00600	Sphingolipid metabolism	0.3942944	50555/683713	2	Ugt8/Gal3st1	https://www.kegg.jp/kegg-bin/show_pathway?rno00600/50555%09red/683713%09red	Metabolism	Lipid metabolism
rno00532	Glycosaminoglycan biosynthesis - chondroitin sulfate / dermatan sulfate	0.398679541	500257	1	Chst13	https://www.kegg.jp/kegg-bin/show_pathway?rno00532/500257%09red	Metabolism	Glycan biosynthesis and metabolism
rno05410	Hypertrophic cardiomyopathy	0.40065476	25717/305941/24179	3	Tgfb3/Sgcg/Agt	https://www.kegg.jp/kegg-bin/show_pathway?rno05410/25717%09red/305941%09red/24179%09red	Human Diseases	Cardiovascular disease
rno02010	ABC transporters	0.403165833	170913/24255	2	Abcb1a/Cftr	https://www.kegg.jp/kegg-bin/show_pathway?rno020	Environmental Information Processing	Membrane transport

						10/170913%09red/24255%09red		
rno05032	Morphine addiction	0.40743041	685451/29713/81743	3	Gng13/Kcnj5/Pde2a	https://www.kegg.jp/kegg-bin/show_pathway?rno05032/685451%09red/29713%09red/81743%09red	Human Diseases	Substance dependence
rno04530	Tight junction	0.408799849	291287/304388/54254/24255/685867	5	Tubal3/Cldn15/Gata4/Cftr/Myl6	https://www.kegg.jp/kegg-bin/show_pathway?rno04530/291287%09red/304388%09red/54254%09red/24255%09red/685867%09red	Cellular Processes	Cellular community - eukaryotes
rno04923	Regulation of lipolysis in adipocytes	0.411975531	25629/79451	2	Plin1/Fabp4	https://www.kegg.jp/kegg-bin/show_pathway?rno04923/25629%09red/79451%09red	Organismal Systems	Endocrine system
rno04978	Mineral absorption	0.420721007	24825/664630	2	Tf/Slc6a19	https://www.kegg.jp/kegg-bin/show_pathway?rno04978/24825%09red/664630%09red	Organismal Systems	Digestive system
rno05414	Dilated cardiomyopathy	0.420912285	25717/305941/24179	3	Tgfb3/Sgcg/Agt	https://www.kegg.jp/kegg-bin/show_pathway?rno05414/25717%09red/305941%09red/24179%09red	Human Diseases	Cardiovascular disease
rno00430	Taurine and hypotaurine metabolism	0.428535884	81718	1	Cdo1	https://www.kegg.jp/kegg-bin/show_pathway?rno00430/81718%09red	Metabolism	Metabolism of other amino acids
rno03430	Mismatch repair	0.428535884	296883	1	Rpa3	https://www.kegg.jp/kegg-bin/show_pathway?rno03430/296883%09red	Genetic Information Processing	Replication and repair
rno04913	Ovarian steroidogene-	0.429399936	79243/25644	2	Hsd17b2/Bmp6	https://www.kegg.jp/kegg-	Organismal Systems	Endocrine system

	sis					bin/show_pathway?rno04913/79243%09red/25644%09red		
rno04068	FoxO signaling pathway	0.434720641	25313/25717/362282/25634	4	Egf/Tgfb3/Pck1/G6pc	https://www.kegg.jp/kegg-bin/show_pathway?rno04068/25313%09red/25717%09red/362282%09red/25634%09red	Environmental Information Processing	Signal transduction
rno04713	Circadian entrainment	0.447556675	685451/29713/64455	3	Gng13/Kcnj5/Rasd1	https://www.kegg.jp/kegg-bin/show_pathway?rno04713/685451%09red/29713%09red/64455%09red	Organismal Systems	Environmental adaptation
rno04916	Melanogenesis	0.45414315	64558/363913/116466	3	Fzd4/Fzd10/Wnt2b	https://www.kegg.jp/kegg-bin/show_pathway?rno04916/64558%09red/363913%09red/116466%09red	Organismal Systems	Endocrine system
rno04910	Insulin signaling pathway	0.462615654	24362/362282/25634/24651	4	Fbp1/Pck1/G6pc/Pklr	https://www.kegg.jp/kegg-bin/show_pathway?rno04910/24362%09red/362282%09red/25634%09red/24651%09red	Organismal Systems	Endocrine system
rno00340	Histidine metabolism	0.47057553	298079	1	Aldh1b1	https://www.kegg.jp/kegg-bin/show_pathway?rno00340/298079%09red	Metabolism	Amino acid metabolism
rno00900	Terpenoid backbone biosynthesis	0.47057553	24450	1	Hmgcs2	https://www.kegg.jp/kegg-bin/show_pathway?rno00900/24450%09red	Metabolism	Metabolism of terpenoids and polyketides
rno04062	Chemokine signaling pathway	0.482653776	288593/685451/360918/117027/29397	5	Ccl24/Gng13/Pf4/Ccr3/Ccl11	https://www.kegg.jp/kegg-bin/show_pathway?rno04062/288593%09red/685451%09red/3609	Organismal Systems	Immune system

						18%09red/117027%09red/29397%09red		
rno04664	Fc epsilon RI signaling pathway	0.496205244	25047/25316	2	Fcer1a/Ms4a2	https://www.kegg.jp/kegg-bin/show_pathway?rno04664/25047%09red/25316%09red	Organismal Systems	Immune system
rno04392	Hippo signaling pathway - multiple species	0.509535327	305251	1	Rassf6	https://www.kegg.jp/kegg-bin/show_pathway?rno04392/305251%09red	Environmental Information Processing	Signal transduction
rno05224	Breast cancer	0.511535318	64558/25313/363913/116466	4	Fzd4/Egf/Fzd10/Wnt2b	https://www.kegg.jp/kegg-bin/show_pathway?rno05224/64558%09red/25313%09red/363913%09red/116466%09red	Human Diseases	Cancer: specific types
rno05221	Acute myeloid leukemia	0.512121245	25410/295052	2	Cebpe/Ccna1	https://www.kegg.jp/kegg-bin/show_pathway?rno05221/25410%09red/295052%09red	Human Diseases	Cancer: specific types
rno01523	Antifolate resistance	0.521876833	303252	1	Alox12e	https://www.kegg.jp/kegg-bin/show_pathway?rno01523/303252%09red	Human Diseases	Drug resistance: antineoplastic
rno05211	Renal cell carcinoma	0.527704856	25717/25243	2	Tgfb3/Arnt2	https://www.kegg.jp/kegg-bin/show_pathway?rno05211/25717%09red/25243%09red	Human Diseases	Cancer: specific types
rno05208	Chemical carcinogenesis - reactive oxygen species	0.536840719	25315/25313/64352/24423/171341/24421	6	Ephx1/Egf/Gstm5/Gstm1/Mgst1/Gsta1	https://www.kegg.jp/kegg-bin/show_pathway?rno05208/25315%09red/25313%09red/64352%09red/24423%09red/171341%09red/24421%09red	Human Diseases	Cancer: overview
rno04725	Cholinergic	0.542463287	685451/2522	3	Gng13/Chrm	https://www.k	Organismal	Nervous

	synapse		9/25590		1/Chrna4	egg.jp/kegg-bin/show_pathway?rno04725/685451%09red/25229%09red/25590%09red	Systems	system
rno01240	Biosynthesis of cofactors	0.543009048	25106/298079/24861/64203	4	Rgn/Aldh1b1/Ugt1a1/Bcat2	https://www.kegg.jp/kegg-bin/show_pathway?rno01240/25106%09red/298079%09red/24861%09red/64203%09red	Metabolism	Global and overview maps
rno00020	Citrate cycle (TCA cycle)	0.545639968	362282	1	Pck1	https://www.kegg.jp/kegg-bin/show_pathway?rno00020/362282%09red	Metabolism	Carbohydrate metabolism
rno00410	beta-Alanine metabolism	0.545639968	298079	1	Aldh1b1	https://www.kegg.jp/kegg-bin/show_pathway?rno00410/298079%09red	Metabolism	Metabolism of other amino acids
rno00640	Propanoate metabolism	0.545639968	314800	1	Acss3	https://www.kegg.jp/kegg-bin/show_pathway?rno00640/314800%09red	Metabolism	Carbohydrate metabolism
rno04066	HIF-1 signaling pathway	0.548457306	25313/24825/24190	3	Egf/Tf/Aldob	https://www.kegg.jp/kegg-bin/show_pathway?rno04066/25313%09red/24825%09red/24190%09red	Environmental Information Processing	Signal transduction
rno04918	Thyroid hormone synthesis	0.550443774	24210/29403	2	Asgr1/Asgr2	https://www.kegg.jp/kegg-bin/show_pathway?rno04918/24210%09red/29403%09red	Organismal Systems	Endocrine system
rno04750	Inflammatory mediator regulation of TRP channels	0.554405418	499353/50549/303252	3	Cyp2c24/Cyp4a1/Alox12e	https://www.kegg.jp/kegg-bin/show_pathway?rno04750/499353%09red/50549%09red/303252%09red	Organismal Systems	Sensory system

rno00052	Galactose metabolism	0.557076832	25634	1	G6pc	https://www.kegg.jp/kegg-bin/show_pathway?rno00052/25634%09red	Metabolism	Carbohydrate metabolism
rno00500	Starch and sucrose metabolism	0.557076832	25634	1	G6pc	https://www.kegg.jp/kegg-bin/show_pathway?rno00500/25634%09red	Metabolism	Carbohydrate metabolism
rno04150	mTOR signaling pathway	0.563409409	317203/64558/363913/116466	4	Rps6ka6/Fzd4/Fzd10/Wnt2b	https://www.kegg.jp/kegg-bin/show_pathway?rno04150/317203%09red/64558%09red/363913%09red/116466%09red	Environmental Information Processing	Signal transduction
rno05212	Pancreatic cancer	0.565172173	25313/25717	2	Egf/Tgfb3	https://www.kegg.jp/kegg-bin/show_pathway?rno05212/25313%09red/25717%09red	Human Diseases	Cancer: specific types
rno04130	SNARE interactions in vesicular transport	0.579097825	29631	1	Bet1	https://www.kegg.jp/kegg-bin/show_pathway?rno04130/29631%09red	Genetic Information Processing	Folding, sorting and degradation
rno04721	Synaptic vesicle cycle	0.586611151	366432/25693	2	Slc1a7/Slc18a1	https://www.kegg.jp/kegg-bin/show_pathway?rno04721/366432%09red/25693%09red	Organismal Systems	Nervous system
rno03030	DNA replication	0.589696081	296883	1	Rpa3	https://www.kegg.jp/kegg-bin/show_pathway?rno03030/296883%09red	Genetic Information Processing	Replication and repair
rno00760	Nicotinate and nicotinamide metabolism	0.610102122	493909	1	Aox3	https://www.kegg.jp/kegg-bin/show_pathway?rno00760/493909%09red	Metabolism	Metabolism of cofactors and vitamins
rno01250	Biosynthesis of nucleotide sugars	0.610102122	361245	1	Cmahp	https://www.kegg.jp/kegg-bin/show_pathway?rno012	Metabolism	Global and overview maps

						50/361245%09red		
rno04960	Aldosterone-regulated sodium reab-sorption	0.619923007	116501	1	Slc9a3r2	https://www.kegg.jp/kegg-bin/show_pathway?rno04960/116501%09red	Organismal Systems	Excretory system
rno05216	Thyroid cancer	0.619923007	25664	1	Pparg	https://www.kegg.jp/kegg-bin/show_pathway?rno05216/25664%09red	Human Dis-eases	Cancer: spe-cific types
rno00591	Linoleic acid metabolism	0.63883204	499353	1	Cyp2c24	https://www.kegg.jp/kegg-bin/show_pathway?rno00591/499353%09red	Metabolism	Lipid metabo-lism
rno05033	Nicotine addiction	0.63883204	25590	1	Chrna4	https://www.kegg.jp/kegg-bin/show_pathway?rno05033/25590%09red	Human Dis-eases	Substance dependence
rno04146	Peroxisome	0.646200305	286937/114209	2	Pex5l/Phyh	https://www.kegg.jp/kegg-bin/show_pathway?rno04146/286937%09red/114209%09red	Cellular Processes	Transport and catabolism
rno04540	Gap junction	0.646200305	291287/25313	2	Tubal3/Egf	https://www.kegg.jp/kegg-bin/show_pathway?rno04540/291287%09red/25313%09red	Cellular Processes	Cellular community - eukaryotes
rno03440	Homologous recombina-tion	0.647932335	296883	1	Rpa3	https://www.kegg.jp/kegg-bin/show_pathway?rno03440/296883%09red	Genetic In-formation Processing	Replication and repair
rno05219	Bladder can-cer	0.647932335	25313	1	Egf	https://www.kegg.jp/kegg-bin/show_pathway?rno05219/25313%09red	Human Dis-eases	Cancer: spe-cific types
rno04728	Dopaminer-gic synapse	0.648062232	685451/29713/25693	3	Gng13/Kcnj5/Slc18a1	https://www.kegg.jp/kegg-bin/show_pathway?rno04728/685451%0	Organismal Systems	Nervous system

						9red/29713%09red/25693%09red		
rno05210	Colorectal cancer	0.658486028	25313/25717	2	Egf/Tgfb3	https://www.kegg.jp/kegg-bin/show_pathway?rno05210/25313%09red/25717%09red	Human Diseases	Cancer: specific types
rno04810	Regulation of actin cytoskeleton	0.663020632	298975/25313/25229/29251/494222	5	Scin/Egf/Chrm1/F2/Pfn4	https://www.kegg.jp/kegg-bin/show_pathway?rno04810/298975%09red/25313%09red/25229%09red/29251%09red/494222%09red	Cellular Processes	Cell motility
rno04936	Alcoholic liver disease	0.663066401	100145871/298079/246253	3	Adh5/Aldh1b1/Adipoq	https://www.kegg.jp/kegg-bin/show_pathway?rno04936/100145871%09red/298079%09red/246253%09red	Human Diseases	Endocrine and metabolic disease
rno04211	Longevity regulating pathway	0.6645003	25664/246253	2	Pparg/Adipoq	https://www.kegg.jp/kegg-bin/show_pathway?rno04211/25664%09red/246253%09red	Organismal Systems	Aging
rno04640	Hematopoietic cell lineage	0.6645003	305081/114103	2	Cd34/Il5ra	https://www.kegg.jp/kegg-bin/show_pathway?rno04640/305081%09red/114103%09red	Organismal Systems	Immune system
rno00860	Porphyrin metabolism	0.665453725	24861	1	Ugt1a1	https://www.kegg.jp/kegg-bin/show_pathway?rno00860/24861%09red	Metabolism	Metabolism of cofactors and vitamins
rno04672	Intestinal immune network for IgA production	0.665453725	287034	1	Tnfrsf17	https://www.kegg.jp/kegg-bin/show_pathway?rno04672/287034%09red	Organismal Systems	Immune system
rno04742	Taste transduction	0.670429413	685451/25075	2	Gng13/Htr1b	https://www.kegg.jp/kegg-bin/show_pat	Organismal Systems	Sensory system

						hway?rno04742/685451%09red/25075%09red		
rno04371	Apelin signaling pathway	0.672810194	685451/25629/81638	3	Gng13/Plin1/Agtr1b	https://www.kegg.jp/kegg-bin/show_pathway?rno04371/685451%09red/25629%09red/81638%09red	Environmental Information Processing	Signal transduction
rno05012	Parkinson disease	0.676074474	117549/291287/295455/29219/289754/25693	6	Gpr37/Tubal3/Slc39a8/Snca/Xbp1/Slc18a1	https://www.kegg.jp/kegg-bin/show_pathway?rno05012/117549%09red/291287%09red/295455%09red/29219%09red/289754%09red/25693%09red	Human Diseases	Neurodegenerative disease
rno04914	Progesterone-mediated oocyte maturation	0.676273835	317203/295052	2	Rps6ka6/Ccna1	https://www.kegg.jp/kegg-bin/show_pathway?rno04914/317203%09red/295052%09red	Organismal Systems	Endocrine system
rno03420	Nucleotide excision repair	0.682106825	296883	1	Rpa3	https://www.kegg.jp/kegg-bin/show_pathway?rno03420/296883%09red	Genetic Information Processing	Replication and repair
rno04218	Cellular senescence	0.686954094	25717/54254/24484/295052	4	Tgfb3/Gata4/Igfbp3/Ccna1	https://www.kegg.jp/kegg-bin/show_pathway?rno04218/25717%09red/54254%09red/24484%09red/295052%09red	Cellular Processes	Cell growth and death
rno04973	Carbohydrate digestion and absorption	0.69012124	25634	1	G6pc	https://www.kegg.jp/kegg-bin/show_pathway?rno04973/25634%09red	Organismal Systems	Digestive system
rno05171	Coronavirus disease - COVID-19	0.691256152	302668/64023/25347/60327/29251/81638/54249	7	Ace2/Masp1/Rpl39/F13a1/F2/Agtr1b/Cfd	https://www.kegg.jp/kegg-bin/show_pathway?rno05171/302668%09red/64023%	Human Diseases	Infectious disease: viral

						09red/25347%09red/60327%09red/29251%09red/81638%09red/54249%09red		
rno05030	Cocaine addiction	0.697934482	25693	1	Slc18a1	https://www.kegg.jp/kegg-bin/show_pathway?rno05030/25693%09red	Human Diseases	Substance dependence
rno04714	Thermogenesis	0.714420066	317203/24952/25629/25664/680931	5	Rps6ka6/Gcg/Plin1/Pparg/Bmp8a	https://www.kegg.jp/kegg-bin/show_pathway?rno04714/317203%09red/24952%09red/25629%09red/25664%09red/680931%09red	Organismal Systems	Environmental adaptation
rno05215	Prostate cancer	0.714858571	25313/303828	2	Egf/Etv5	https://www.kegg.jp/kegg-bin/show_pathway?rno05215/25313%09red/303828%09red	Human Diseases	Cancer: specific types
rno00510	N-Glycan biosynthesis	0.720216828	502017	1	Dpm3	https://www.kegg.jp/kegg-bin/show_pathway?rno00510/502017%09red	Metabolism	Glycan biosynthesis and metabolism
rno00520	Amino sugar and nucleotide sugar metabolism	0.727274421	361245	1	Cmahp	https://www.kegg.jp/kegg-bin/show_pathway?rno00520/361245%09red	Metabolism	Carbohydrate metabolism
rno03460	Fanconi anemia pathway	0.727274421	296883	1	Rpa3	https://www.kegg.jp/kegg-bin/show_pathway?rno03460/296883%09red	Genetic Information Processing	Replication and repair
rno04340	Hedgehog signaling pathway	0.73415476	29499	1	Shh	https://www.kegg.jp/kegg-bin/show_pathway?rno04340/29499%09red	Environmental Information Processing	Signal transduction
rno04921	Oxytocin signaling pathway	0.751272681	29713/25342/685867	3	Kcnj5/Oxtr/Myl6	https://www.kegg.jp/kegg-bin/show_pathway?rno049	Organismal Systems	Endocrine system

						21/29713%09red/25342%09red/685867%09red		
rno05213	Endometrial cancer	0.777712759	25313	1	Egf	https://www.kegg.jp/kegg-bin/show_pathway?rno05213/25313%09red	Human Diseases	Cancer: specific types
rno04724	Glutamatergic synapse	0.784667037	366432/685451	2	Slc1a7/Gng13	https://www.kegg.jp/kegg-bin/show_pathway?rno04724/366432%09red/685451%09red	Organismal Systems	Nervous system
rno05321	Inflammatory bowel disease	0.788797513	25717	1	Tgfb3	https://www.kegg.jp/kegg-bin/show_pathway?rno05321/25717%09red	Human Diseases	Immune disease
rno05415	Diabetic cardiomyopathy	0.789919434	25717/100360872/81638/24179	4	Tgfb3/Mcpt11/Agtr1b/Agt	https://www.kegg.jp/kegg-bin/show_pathway?rno05415/25717%09red/100360872%09red/81638%09red/24179%09red	Human Diseases	Cardiovascular disease
rno04929	GnRH secretion	0.804401172	29713	1	Kcnj5	https://www.kegg.jp/kegg-bin/show_pathway?rno04929/29713%09red	Organismal Systems	Endocrine system
rno04022	cGMP-PKG signaling pathway	0.807727937	54254/81743/81638	3	Gata4/Pde2a/Agtr1b	https://www.kegg.jp/kegg-bin/show_pathway?rno04022/54254%09red/81743%09red/81638%09red	Environmental Information Processing	Signal transduction
rno04722	Neurotrophin signaling pathway	0.808119162	317203/84469	2	Rps6ka6/Maged1	https://www.kegg.jp/kegg-bin/show_pathway?rno04722/317203%09red/84469%09red	Organismal Systems	Nervous system
rno04514	Cell adhesion molecules	0.81087042	304388/303926/305081	3	Cldn15/Igsf11/Cd34	https://www.kegg.jp/kegg-bin/show_pathway?rno045	Environmental Information Processing	Signaling molecules and interaction

						14/304388%09red/303926%09red/305081%09red		
rno04380	Osteoclast differentia-tion	0.811800158	25664/301227	2	Pparg/Trem2	https://www.kegg.jp/kegg-bin/show_pathway?rno04380/25664%09red/301227%09red	Organismal Systems	Development and regenera-tion
rno04720	Long-term potentiation	0.818856797	317203	1	Rps6ka6	https://www.kegg.jp/kegg-bin/show_pathway?rno04720/317203%09red	Organismal Systems	Nervous system
rno04071	Sphingolipid signaling pathway	0.822469238	25047/25316	2	Fcer1a/Ms4a2	https://www.kegg.jp/kegg-bin/show_pathway?rno04071/25047%09red/25316%09red	Environmen-tal Infor-mation Pro-cessing	Signal trans-duction
rno05031	Amphetamine addiction	0.823434939	25693	1	Slc18a1	https://www.kegg.jp/kegg-bin/show_pathway?rno05031/25693%09red	Human Dis-eases	Substance dependence
rno04940	Type I diabe-tes mellitus	0.827897879	29714	1	Ptprn2	https://www.kegg.jp/kegg-bin/show_pathway?rno04940/29714%09red	Human Dis-eases	Endocrine and metabolic disease
rno04110	Cell cycle	0.832594628	25717/295052	2	Tgfb3/Ccna1	https://www.kegg.jp/kegg-bin/show_pathway?rno04110/25717%09red/295052%09red	Cellular Processes	Cell growth and death
rno05140	Leishmania-sis	0.836489624	25717	1	Tgfb3	https://www.kegg.jp/kegg-bin/show_pathway?rno05140/25717%09red	Human Dis-eases	Infectious disease: para-sitic
rno04115	p53 signaling pathway	0.844654268	24484	1	Igfbp3	https://www.kegg.jp/kegg-bin/show_pathway?rno04115/24484%09red	Cellular Processes	Cell growth and death
rno05133	Pertussis	0.844654268	24773	1	Sftpa1	https://www.kegg.jp/kegg-	Human Dis-	Infectious disease: bac-

						bin/show_pathway?rno05133/24773%09red	eases	terial
rno05218	Melanoma	0.844654268	25313	1	Egf	https://www.kegg.jp/kegg-bin/show_pathway?rno05218/25313%09red	Human Diseases	Cancer: specific types
rno05223	Non-small cell lung cancer	0.844654268	25313	1	Egf	https://www.kegg.jp/kegg-bin/show_pathway?rno05223/25313%09red	Human Diseases	Cancer: specific types
rno00230	Purine metabolism	0.845287248	81743/25368	2	Pde2a/Adk	https://www.kegg.jp/kegg-bin/show_pathway?rno00230/81743%09red/25368%09red	Metabolism	Nucleotide metabolism
rno05214	Glioma	0.848583075	25313	1	Egf	https://www.kegg.jp/kegg-bin/show_pathway?rno05214/25313%09red	Human Diseases	Cancer: specific types
rno04917	Prolactin signaling pathway	0.852412952	24684	1	Prlr	https://www.kegg.jp/kegg-bin/show_pathway?rno04917/24684%09red	Organismal Systems	Endocrine system
rno05022	Pathways of neurodegeneration - multiple diseases	0.853110365	117549/291287/64558/29219/25229/363913/289754/295445/116466	9	Gpr37/Tubal3/Fzd4/Snca/Chrm1/Fzd10/Xbp1/Dkk2/Wnt2b	https://www.kegg.jp/kegg-bin/show_pathway?rno05022/117549%09red/291287%09red/64558%09red/29219%09red/25229%09red/363913%09red/289754%09red/295445%09red/116466%09red	Human Diseases	Neurodegenerative disease
rno04915	Estrogen signaling pathway	0.857103391	29713/294853	2	Kcnj5/Krt18	https://www.kegg.jp/kegg-bin/show_pathway?rno04915/29713%09red/294853%09red	Organismal Systems	Endocrine system

rno05412	Arrhythmogenic right ventricular cardiomyopathy	0.859785775	305941	1	Sgcg	https://www.kegg.jp/kegg-bin/show_pathway?rno05412/305941%09red	Human Diseases	Cardiovascular disease
rno04210	Apoptosis	0.859926364	291287/361704	2	Tubal3/Ctsf	https://www.kegg.jp/kegg-bin/show_pathway?rno04210/291287%09red/361704%09red	Cellular Processes	Cell growth and death
rno05220	Chronic myeloid leukemia	0.863333498	25717	1	Tgfb3	https://www.kegg.jp/kegg-bin/show_pathway?rno05220/25717%09red	Human Diseases	Cancer: specific types
rno04151	PI3K-Akt signaling pathway	0.869877357	685451/25313/25229/362282/25634/24684	6	Gng13/Egf/Chrm1/Pck1/G6pc/Prlr	https://www.kegg.jp/kegg-bin/show_pathway?rno04151/685451%09red/25313%09red/25229%09red/362282%09red/25634%09red/24684%09red	Environmental Information Processing	Signal transduction
rno01521	EGFR tyrosine kinase inhibitor resistance	0.870163061	25313	1	Egf	https://www.kegg.jp/kegg-bin/show_pathway?rno01521/25313%09red	Human Diseases	Drug resistance: antineoplastic
rno01232	Nucleotide metabolism	0.876652782	25368	1	Adk	https://www.kegg.jp/kegg-bin/show_pathway?rno01232/25368%09red	Metabolism	Global and overview maps
rno04012	ErbB signaling pathway	0.885786764	25313	1	Egf	https://www.kegg.jp/kegg-bin/show_pathway?rno04012/25313%09red	Environmental Information Processing	Signal transduction
rno05416	Viral myocarditis	0.885786764	305941	1	Sgcg	https://www.kegg.jp/kegg-bin/show_pathway?rno05416/305941%09red	Human Diseases	Cardiovascular disease
rno05323	Rheumatoid arthritis	0.888679219	25717	1	Tgfb3	https://www.kegg.jp/kegg-bin/show_pat	Human Diseases	Immune disease

						hway?rno05323/25717%09red		
rno04261	Adrenergic signaling in cardiomyocytes	0.89004668	81638/24179	2	Agtr1b/Agt	https://www.kegg.jp/kegg-bin/show_pathway?rno04261/81638%09red/24179%09red	Organismal Systems	Circulatory system
rno04260	Cardiac muscle contraction	0.894247162	59299	1	Trdn	https://www.kegg.jp/kegg-bin/show_pathway?rno04260/59299%09red	Organismal Systems	Circulatory system
rno04723	Retrograde endocannabinoid signaling	0.896580219	685451/29713	2	Gng13/Kcnj5	https://www.kegg.jp/kegg-bin/show_pathway?rno04723/685451%09red/29713%09red	Organismal Systems	Nervous system
rno05235	PD-L1 expression and PD-1 checkpoint pathway in cancer	0.896926266	25313	1	Egf	https://www.kegg.jp/kegg-bin/show_pathway?rno05235/25313%09red	Human Diseases	Cancer: overview
rno05417	Lipid and atherosclerosis	0.90004613	54225/25664/289754	3	Apob/Pparg/Xbp1	https://www.kegg.jp/kegg-bin/show_pathway?rno05417/54225%09red/25664%09red/289754%09red	Human Diseases	Cardiovascular disease
rno04727	GABAergic synapse	0.902083442	685451	1	Gng13	https://www.kegg.jp/kegg-bin/show_pathway?rno04727/685451%09red	Organismal Systems	Nervous system
rno04657	IL-17 signaling pathway	0.904564866	29397	1	Ccl11	https://www.kegg.jp/kegg-bin/show_pathway?rno04657/29397%09red	Organismal Systems	Immune system
rno05161	Hepatitis B	0.908580686	25717/295052	2	Tgfb3/Ccna1	https://www.kegg.jp/kegg-bin/show_pathway?rno05161/25717%09red/295052%09red	Human Diseases	Infectious disease: viral

rno05160	Hepatitis C	0.910449999	304388/25313	2	Cldn15/Egf	https://www.kegg.jp/kegg-bin/show_pathway?rno05160/304388%09red/25313%09red	Human Diseases	Infectious disease: viral
rno04666	Fc gamma R-mediated phagocytosis	0.911639724	298975	1	Scin	https://www.kegg.jp/kegg-bin/show_pathway?rno04666/298975%09red	Organismal Systems	Immune system
rno05146	Amoebiasis	0.913879985	25717	1	Tgfb3	https://www.kegg.jp/kegg-bin/show_pathway?rno05146/25717%09red	Human Diseases	Infectious disease: parasitic
rno04064	NF-kappa B signaling pathway	0.918192272	365581	1	Edar	https://www.kegg.jp/kegg-bin/show_pathway?rno04064/365581%09red	Environmental Information Processing	Signal transduction
rno05231	Choline metabolism in cancer	0.918192272	25313	1	Egf	https://www.kegg.jp/kegg-bin/show_pathway?rno05231/25313%09red	Human Diseases	Cancer: overview
rno05203	Viral carcinogenesis	0.919561563	298975/117027/295052	3	Scin/Ccr3/Ccna1	https://www.kegg.jp/kegg-bin/show_pathway?rno05203/298975%09red/117027%09red/295052%09red	Human Diseases	Cancer: overview
rno05142	Chagas disease	0.929881225	25717	1	Tgfb3	https://www.kegg.jp/kegg-bin/show_pathway?rno05142/25717%09red	Human Diseases	Infectious disease: parasitic
rno05034	Alcoholism	0.939835479	685451/25693	2	Gng13/Slc18a1	https://www.kegg.jp/kegg-bin/show_pathway?rno05034/685451%09red/25693%09red	Human Diseases	Substance dependence
rno05145	Toxoplasmosis	0.941432592	25717	1	Tgfb3	https://www.kegg.jp/kegg-bin/show_pathway?rno051	Human Diseases	Infectious disease: parasitic

						45/25717%09red		
rno04360	Axon guidance	0.944732684	65047/29499	2	Slit1/Shh	https://www.kegg.jp/kegg-bin/show_pathway?rno04360/65047%09red/29499%09red	Organismal Systems	Development and regeneration
rno05165	Human papillomavirus infection	0.945597623	64558/25313/363913/116466/295052	5	Fzd4/Egf/Fzd10/Wnt2b/Ccna1	https://www.kegg.jp/kegg-bin/show_pathway?rno05165/64558%09red/25313%09red/363913%09red/116466%09red/295052%09red	Human Diseases	Infectious disease: viral
rno04145	Phagosome	0.949250474	291287/24773	2	Tubal3/Sftpa1	https://www.kegg.jp/kegg-bin/show_pathway?rno04145/291287%09red/24773%09red	Cellular Processes	Transport and catabolism
rno04670	Leukocyte transendothelial migration	0.949812453	304388	1	Cldn15	https://www.kegg.jp/kegg-bin/show_pathway?rno04670/304388%09red	Organismal Systems	Immune system
rno04114	Oocyte meiosis	0.953543272	317203	1	Rps6ka6	https://www.kegg.jp/kegg-bin/show_pathway?rno04114/317203%09red	Cellular Processes	Cell growth and death
rno04919	Thyroid hormone signaling pathway	0.958091627	54254	1	Gata4	https://www.kegg.jp/kegg-bin/show_pathway?rno04919/54254%09red	Organismal Systems	Endocrine system
rno04611	Platelet activation	0.960196692	29251	1	F2	https://www.kegg.jp/kegg-bin/show_pathway?rno04611/29251%09red	Organismal Systems	Immune system
rno04926	Relaxin signaling pathway	0.962196467	685451	1	Gng13	https://www.kegg.jp/kegg-bin/show_pathway?rno04926/685451%09red	Organismal Systems	Endocrine system

rno04142	Lysosome	0.967615207	361704	1	Ctsf	https://www.kegg.jp/kegg-bin/show_pathway?rno04142/361704%09red	Cellular Processes	Transport and catabolism
rno05135	Yersinia infection	0.968439922	317203	1	Rps6ka6	https://www.kegg.jp/kegg-bin/show_pathway?rno05135/317203%09red	Human Diseases	Infectious disease: bacterial
rno04015	Rap1 signaling pathway	0.972332454	25313/494222	2	Egf/Pfn4	https://www.kegg.jp/kegg-bin/show_pathway?rno04015/25313%09red/494222%09red	Environmental Information Processing	Signal transduction
rno05167	Kaposi sarcoma-associated herpesvirus infection	0.974097536	685451/117027	2	Gng13/Ccr3	https://www.kegg.jp/kegg-bin/show_pathway?rno05167/685451%09red/117027%09red	Human Diseases	Infectious disease: viral
rno05017	Spinocerebellar ataxia	0.975619845	289754	1	Xbp1	https://www.kegg.jp/kegg-bin/show_pathway?rno05017/289754%09red	Human Diseases	Neurodegenerative disease
rno05206	MicroRNAs in cancer	0.979478321	314930/246334/170913	3	Zfpm2/Tp63/Abcb1a	https://www.kegg.jp/kegg-bin/show_pathway?rno05206/314930%09red/246334%09red/170913%09red	Human Diseases	Cancer: overview
rno04010	MAPK signaling pathway	0.979886299	317203/25313/25717	3	Rps6ka6/Egf/Tgfb3	https://www.kegg.jp/kegg-bin/show_pathway?rno04010/317203%09red/25313%09red/25717%09red	Environmental Information Processing	Signal transduction
rno04014	Ras signaling pathway	0.981415876	685451/25313	2	Gng13/Egf	https://www.kegg.jp/kegg-bin/show_pathway?rno04014/685451%09red/25313%09red	Environmental Information Processing	Signal transduction
rno05014	Amyotrophic lateral sclero-	0.985114028	291287/289754/305843/49	4	Tubal3/Xbp1/A	https://www.kegg.jp/kegg-	Human Diseases	Neurodegenerative dis-

	sis		4222		ng/Pfn4	bin/show_pathway?rno05014/291287%09red/289754%09red/305843%09red/494222%09red		ease
rno04141	Protein processing in endoplasmic reticulum	0.986550335	289754	1	Xbp1	https://www.kegg.jp/kegg-bin/show_pathway?rno04141/289754%09red	Genetic Information Processing	Folding, sorting and degradation
rno05163	Human cytomegalovirus infection	0.987301729	685451/117027	2	Gng13/Ccr3	https://www.kegg.jp/kegg-bin/show_pathway?rno05163/685451%09red/117027%09red	Human Diseases	Infectious disease: viral
rno05166	Human T-cell leukemia virus 1 infection	0.987584787	25717/295052	2	Tgfb3/Ccna1	https://www.kegg.jp/kegg-bin/show_pathway?rno05166/25717%09red/295052%09red	Human Diseases	Infectious disease: viral
rno05132	Salmonella infection	0.988910688	291287/494222	2	Tubal3/Pfn4	https://www.kegg.jp/kegg-bin/show_pathway?rno05132/291287%09red/494222%09red	Human Diseases	Infectious disease: bacterial
rno05152	Tuberculosis	0.989347765	25717	1	Tgfb3	https://www.kegg.jp/kegg-bin/show_pathway?rno05152/25717%09red	Human Diseases	Infectious disease: bacterial
rno04510	Focal adhesion	0.994136489	25313	1	Egf	https://www.kegg.jp/kegg-bin/show_pathway?rno04510/25313%09red	Cellular Processes	Cellular community - eukaryotes
rno05016	Huntington disease	0.996719682	291287/25664	2	Tubal3/Pparg	https://www.kegg.jp/kegg-bin/show_pathway?rno05016/291287%09red/25664%09red	Human Diseases	Neurodegenerative disease
rno05169	Epstein-Barr virus infection	0.99701997	295052	1	Ccna1	https://www.kegg.jp/kegg-bin/show_pathway?rno051	Human Diseases	Infectious disease: viral

						69/295052%09red		
rno03010	Ribosome	0.997171389	25347	1	Rpl39	https://www.kegg.jp/kegg-bin/show_pathway?rno03010/25347%09red	Genetic Information Processing	Translation
rno05170	Human immunodeficiency virus 1 infection	0.997581203	685451	1	Gng13	https://www.kegg.jp/kegg-bin/show_pathway?rno05170/685451%09red	Human Diseases	Infectious disease: viral
rno05020	Prion disease	0.999127816	291287	1	Tubal3	https://www.kegg.jp/kegg-bin/show_pathway?rno05020/291287%09red	Human Diseases	Neurodegenerative disease

ID is the channel number, Description stands for the description of the path, GeneID represents kegg id of all the enriched genes in the pathway, which is divided by "/", Count indicates the number of all enriched genes in the pathway, GeneSymbol represents the gene symbols of all genes enriched in the pathway, which are divided by "/". GeneSymbol and geneID correspond to each other in order from left to right, KEGGLink is the link address of KEGG path graph, Level_a indicates the annotation of KEGG path classification a, which is the reference of hierarchical relationship, Level_b indicates KEGG path grade b annotation.

Support Table 11. CeRNA composed of DE mRNAs,DE lncRNAs,DE miRNAs between the control group and the model group.

lncRNA	lncRNA_regulation	lncRNA_cor	lncRNA_pvalue	miRNA	miRNA_regulation	miRNA_cor	miRNA_pvalue	mRNA	mRNA_regulation	mRNA_cor	mRNA_pvalue
NONRATG013093.2	Up	-0.9428571	0.01666667	rno-miR-139-3p	Down	-0.9428571	0.01666667	Bid	Up	1	0.002777778
NONRATG018580.2	Up	-0.9428571	0.01666667	rno-miR-139-3p	Down	-0.9428571	0.01666667	Bid	Up	1	0.002777778
NONRATG026519.1	Up	-0.8804063	0.02059873	rno-miR-139-3p	Down	-0.9428571	0.01666667	Bid	Up	0.8804063	0.02059873
NONRATG021211.2	Up	-0.9428571	0.01666667	rno-miR-139-3p	Down	-0.9428571	0.01666667	Bid	Up	0.8857143	0.03333333
NONRATG019132.2	Up	-0.9428571	0.01666667	rno-miR-139-3p	Down	-0.9428571	0.01666667	Bid	Up	0.8857143	0.03333333
NONRATG021786.2	Up	-0.8857143	0.03333333	rno-miR-139-3p	Down	-0.9428571	0.01666667	Bid	Up	0.9428571	0.01666667
NONRATG012798.2	Up	-0.9428571	0.01666667	rno-miR-139-3p	Down	-0.9428571	0.01666667	Bid	Up	1	0.002777778
NONRATG018111.2	Up	-0.8451543	0.03410942	rno-miR-378b	Down	-1	0.002777778	Slc4a5	Up	0.8451543	0.03410942
NONRATG001963.2	Up	-0.9428571	0.01666667	rno-miR-378b	Down	-1	0.002777778	Slc4a5	Up	0.9428571	0.01666667
NONRATG008034.2	Up	-0.8857143	0.03333333	rno-miR-378b	Down	-1	0.002777778	Slc4a5	Up	0.8857143	0.03333333
NONRATG019132.2	Up	-0.8857143	0.03333333	rno-miR-378b	Down	-1	0.002777778	Slc4a5	Up	0.8857143	0.03333333
NONRATG021786.2	Up	-0.9428571	0.01666667	rno-miR-378b	Down	-1	0.002777778	Slc4a5	Up	0.9428571	0.01666667

NON-RATG026519.1	Up	-0.8804063	0.02059873	rno-miR-378b	Down	-1	0.002777778	Slc4a5	Up	0.8804063	0.02059873
NON-RATG021441.2	Up	-0.9428571	0.01666667	rno-miR-378b	Down	-1	0.002777778	Slc4a5	Up	0.9428571	0.01666667
NON-RATG014311.2	Up	-0.8857143	0.03333333	rno-miR-378b	Down	-1	0.002777778	Slc4a5	Up	0.8857143	0.03333333

LncRNA_regulation indicates the up-and-down regulation of lncRNA in differential analysis, LncRNA_cor indicates the correlation value between lncRNA and miRNA, LncRNA_*p*value indicates the *p* value of the correlation between lncRNA and miRNA, The rest is the same as it means.

Support Table 11 .CeRNA composed of DE mRNAs,DE lncRNAs,DE miRNAs between the model group and the electroacupuncture group.

lncRNA	lncRNA_regulation	lncRNA_cor	lncRNA_pvalue	miRNA	miR-NA_regulation	miR-NA_cor	miR-NA_pvalue	mRNA	mRNA_regulation	mRNA_cor	mRNA_pvalue
NON-RATG013093.2	Down	-0.9428571	0.01666667	rno-miR-139-3p	Up	-0.9428571	0.01666667	Bid	Down	0.8285714	0.05833333
NON-RATG018580.2	Down	-0.9428571	0.01666667	rno-miR-139-3p	Up	-0.9428571	0.01666667	Bid	Down	0.8285714	0.05833333
NON-RATG026519.1	Down	-0.8804063	0.02059873	rno-miR-139-3p	Up	-0.9428571	0.01666667	Bid	Down	0.8804063	0.02059873
NON-RATG021211.2	Down	-0.8857143	0.03333333	rno-miR-139-3p	Up	-0.9428571	0.01666667	Bid	Down	0.9428571	0.01666667
NON-RATG019132.2	Down	-0.8857143	0.03333333	rno-miR-139-3p	Up	-0.9428571	0.01666667	Bid	Down	0.7714286	0.1027778
NON-RATG021786.2	Down	-0.8857143	0.03333333	rno-miR-139-3p	Up	-0.9428571	0.01666667	Bid	Down	0.9428571	0.01666667
NON-RATG012798.2	Down	-0.9428571	0.01666667	rno-miR-139-3p	Up	-0.9428571	0.01666667	Bid	Down	1	0.002777778
NON-RATG018111.2	Down	-0.8451543	0.03410942	rno-miR-378b	Up	-0.9428571	0.01666667	Slc4a5	Down	0.8451543	0.03410942
NON-RATG001963.2	Down	-0.8857143	0.03333333	rno-miR-378b	Up	-0.9428571	0.01666667	Slc4a5	Down	0.9428571	0.01666667
NON-RATG026519.1	Down	-0.8804063	0.02059873	rno-miR-378b	Up	-0.9428571	0.01666667	Slc4a5	Down	0.8804063	0.02059873
NON-RATG008034.2	Down	-1	0.002777778	rno-miR-378b	Up	-0.9428571	0.01666667	Slc4a5	Down	0.9428571	0.01666667
NON-RATG021441.2	Down	-0.9428571	0.01666667	rno-miR-378b	Up	-0.9428571	0.01666667	Slc4a5	Down	0.8285714	0.05833333
NON-RATG014311.2	Down	-0.9428571	0.01666667	rno-miR-378b	Up	-0.942857	0.01666667	Slc4a5	Down	0.8857143	0.03333333

						1					
NON-RATG021786.2	Down	-0.9428571	0.01666667	rno-miR-378b	Up	-0.9428571	0.01666667	Slc4a5	Down	0.8857143	0.03333333

LncRNA_regulation indicates the up-and-down regulation of lncRNA in differential analysis, LncRNA_cor indicates the correlation value between lncRNA and miRNA, LncRNA_*p*value indicates the *p* value of the correlation between lncRNA and miRNA, The rest is the same as it means.

Support Table 13. KEGG enrichment analysis of *Bid* and *Slc4a5* in ceRNA regulated by EA.

ID	Description	GeneRatio	BgRatio	pvalue	p.adjust	qvalue	geneID	Count	geneSymbol	keggLink	level_a
rno04932	Non-alcoholic fatty liver disease	2/17	161/8940	0.036721804	0.333652927	0.31396368	64625/24536	2	Bid/Lepr	https://www.kegg.jp/kegg-bin/show_pathway?rno04932/64625%09red/24536%09red	Human Diseases
rno04215	Apoptosis - multiple species	1/17	34/8940	0.062777577	0.333652927	0.31396368	64625	1	Bid	https://www.kegg.jp/kegg-bin/show_pathway?rno04215/64625%09red	Cellular Processes
rno04115	p53 signaling pathway	1/17	73/8940	0.130215297	0.333652927	0.31396368	64625	1	Bid	https://www.kegg.jp/kegg-bin/show_pathway?rno04115/64625%09red	Cellular Processes
rno01524	Platinum drug resistance	1/17	83/8940	0.146756181	0.333652927	0.31396368	64625	1	Bid	https://www.kegg.jp/kegg-bin/show_pathway?rno01524/64625%09red	Human Diseases
rno05416	Viral myocarditis	1/17	85/8940	0.15002863	0.333652927	0.31396368	64625	1	Bid	https://www.kegg.jp/kegg-bin/show_pathway?rno05416/64625%09red	Human Diseases
rno04976	Bile secretion	1/17	94/8940	0.164609015	0.338149332	0.318194746	297386	1	Slc4a5	https://www.kegg.jp/kegg-bin/show_pathway?rno04976/297386%09red	Organismal Systems
rno04650	Natural killer cell mediated	1/17	99/8940	0.17260718	0.338149332	0.318194746	64625	1	Bid	https://www.kegg.jp/kegg-	Organismal Systems

	cytotoxicity									bin/show_pathway?rno04650/64625%09red	
rno04071	Sphingolipid signaling pathway	1/17	124/8940	0.211528454	0.363112595	0.341684898	64625	1	Bid	https://www.kegg.jp/kegg-bin/show_pathway?rno04071/64625%09red	Environ-mental Information Processing
rno04210	Apoptosis	1/17	136/8940	0.229592375	0.378827419	0.356472372	64625	1	Bid	https://www.kegg.jp/kegg-bin/show_pathway?rno04210/64625%09red	Cellular Processes
rno05162	Measles	1/17	144/8940	0.241417816	0.383830014	0.361179758	64625	1	Bid	https://www.kegg.jp/kegg-bin/show_pathway?rno05162/64625%09red	Human Diseases
rno05161	Hepatitis B	1/17	157/8940	0.260270493	0.383830014	0.361179758	64625	1	Bid	https://www.kegg.jp/kegg-bin/show_pathway?rno05161/64625%09red	Human Diseases
rno05160	Hepatitis C	1/17	158/8940	0.261702282	0.383830014	0.361179758	64625	1	Bid	https://www.kegg.jp/kegg-bin/show_pathway?rno05160/64625%09red	Human Diseases
rno04217	Necroptosis	1/17	164/8940	0.270238391	0.387444574	0.364581018	64625	1	Bid	https://www.kegg.jp/kegg-bin/show_pathway?rno04217/64625%09red	Cellular Processes
rno05164	Influenza A	1/17	169/8940	0.27728077	0.387444574	0.364581018	64625	1	Bid	https://www.kegg.jp/kegg-bin/show_pathway?rno05164/64625%09red	Human Diseases
rno05152	Tuberculosis	1/17	177/8940	0.288415684	0.387444574	0.364581018	64625	1	Bid	https://www.kegg.jp/kegg-bin/show_pa	Human Diseases

										thway?rno05152/64625%09red	
rno05417	Lipid and atherosclero-sis	1/17	209/8940	0.331361109	0.420573715	0.395755171	64625	1	Bid	https://www.kegg.jp/kegg-bin/show_pathway?rno05417/64625%09red	Human Diseases
rno05167	Kaposi sarcoma-associated herpesvirus infection	1/17	216/8940	0.340424423	0.423924753	0.39890846	64625	1	Bid	https://www.kegg.jp/kegg-bin/show_pathway?rno05167/64625%09red	Human Diseases
rno05169	Epstein-Barr virus infec-tion	1/17	226/8940	0.353171623	0.428059568	0.402799275	64625	1	Bid	https://www.kegg.jp/kegg-bin/show_pathway?rno05169/64625%09red	Human Diseases
rno05170	Human immunode-ficiency virus 1 infection	1/17	234/8940	0.363202058	0.428059568	0.402799275	64625	1	Bid	https://www.kegg.jp/kegg-bin/show_pathway?rno05170/64625%09red	Human Diseases
rno05163	Human cytomegalo-virus infec-tion	1/17	248/8940	0.380403947	0.440467729	0.414475215	64625	1	Bid	https://www.kegg.jp/kegg-bin/show_pathway?rno05163/64625%09red	Human Diseases
rno05014	Amyo-trophic lateral sclerosis	1/17	370/8940	0.512863629	0.537285706	0.505579851	64625	1	Bid	https://www.kegg.jp/kegg-bin/show_pathway?rno05014/64625%09red	Human Diseases
rno05168	Herpes simplex virus 1 infection	1/17	384/8940	0.52622899	0.540978147	0.509054397	64625	1	Bid	https://www.kegg.jp/kegg-bin/show_pathway?rno05168/64625%09red	Human Diseases
rno05010	Alzheimer disease	1/17	391/8940	0.532781508	0.540978147	0.509054397	64625	1	Bid	https://www.kegg.jp/kegg-bin/show_pathway?rno05	Human Diseases

										010/64625%09red	
rno05022	Pathways of neurodegen-eration - multiple diseases	1/17	477/8940	0.606628285	0.606628285	0.570830444	64625	1	Bid	https://www.kegg.jp/kegg-bin/show_pathway?rno05022/64625%09red	Human Diseases
